# Burden of disease in the Belt and Road countries from 1990 to 2021: analysis of estimates from the Global Burden of Disease 2021

**DOI:** 10.1186/s41256-025-00403-3

**Published:** 2025-05-01

**Authors:** Youyou Wu, Peishan Ning, Zhenzhen Rao, Li Li, David C. Schwebel, Peixia Cheng, Yanhong Fu, Ruotong Li, Jie Li, Wanhui Wang, Guoqing Hu

**Affiliations:** 1 Department of Epidemiology and Health Statistics, Hunan Provincial Key Laboratory of Clinical Epidemiology, Xiangya School of Public Health, Central South University, Changsha, 410078 China; 2 Department of Psychology, University of Alabama at Birmingham, Birmingham, AL 35294 USA; 3 Department of Child, Adolescent and Women’s Health, School of Public Health, Capital Medical University, Beijing, 100069 China; 4 National Clinical Research Center for Geriatric Disorders, Xiangya Hospital, Central South University, Changsha, 410008 China

**Keywords:** The Belt and Road initiative, Burden of disease, Health disparity, Projection, Sustainable Development Goals

## Abstract

**Background:**

As a newly emerging collaborative platform to boost regional growth and prosperity, the Belt and Road Initiative (BRI) has great potential to promote global health development. However, the overall health status of BRI countries remains unclear. We analyzed the health patterns and its geographical distribution in 149 BRI countries from 1990 to 2021.

**Methods:**

Using the Global Burden of Disease 2021 (GBD 2021) online database, we examined time trends, country and income variations in death rate and disability-adjusted life years (DALY) rate, and compared the trends and projected 2030 values for ten key health-related Sustainable Development Goals (SDGs) indicators among the 149 BRI countries.

**Results:**

The number of deaths and DALYs of BRI countries represented 62.9–66.0% of global deaths and 64.8–66.8% of global DALYs between 1990 and 2021, and both the overall age-standardized death rate and DALY rate continued to be higher in BRI countries than in non-BRI countries throughout the time period studied. Great variations existed across the 149 BRI countries for both level of and changes in age-standardized death rate and DALY rate. The 2030 targets for six health-related SDGs indicators will not be reached in over 70% of BRI countries according to the previous changing speed trajectory.

**Conclusions:**

Our findings demonstrate that BRI countries face a heavy burden of disease that varies across countries, although health outcomes have improved since 1990. Progress toward 2030 targets for six key health-related SDGs indicators in most BRI countries was slow. These findings support calls for more health collaborations, aid programs, and other health service to reduce health disparities across the BRI countries.

**Supplementary Information:**

The online version contains supplementary material available at 10.1186/s41256-025-00403-3.

## Introduction

For many reasons, disparities in health across countries persist as a global challenge [1]. Coordinated efforts initiated by international and regional organizations such as the United Nations (UN) and the World Health Organization (WHO) are critical to reduce health disparities and achieve the Sustainable Development Goals (SDGs) [2]. Nevertheless, despite steady increases in global development assistance to improve health over the past several decades [3–5], large health disparities continue to exist across countries [6] and warrant new global and regional cooperation.

The Belt and Road Initiative (BRI) launched by China and also known as the Silk Road Economic Belt and the 21st Century Maritime Silk Road, represents a new cooperation initiative to promote higher-quality development under multilateralism [7]. By early 2022, the BRI included 149 countries, representing approximately 64% of the world’s population. Seventy-four of the participating countries are classified as low-income and lower middle-income [8]. Given the large populations involved and the shared health challenges, the BRI countries have excellent joint potential to promote global efforts, address health disparities and improve global public health outcomes.

Reflecting this objective, between 2000 and 2017, the annual health assistance from China to BRI countries (typically, African and Asian countries) rose from 0.02 billion to 0.88 billion [9]. In particular, the BRI recently established a regional resilient health system to address major health threats through strengthened collaboration, improved medical supply chains and digital infrastructure, and enhanced health financing [10]. Further, some BRI countries have signed bilateral and multilateral agreements to support global and regional health research, health personnel training, and health system reform [7, 11].

To maximize the potential of using the BRI network as a strategy to promote global health and reduce health disparities, we require a systematic understanding of the burden of disease in the BRI countries. Previous studies reported the prevalence of major infectious diseases including HIV/AIDS [12], malaria [13], and tuberculosis [14], and non-communicable diseases like cancer [15] and glaucoma [16] for specific BRI countries. Additionally, some research compared health systems between China and selected BRI countries (Myanmar, Cambodia, and five Lancang-Mekong countries) [17–19]. These studies focused on specific diseases or a limited number of BRI countries, and are therefore quite valuable but lack a comprehensive perspective.

Additionally, several studies have examined the progress of non-communicable disease-related SDGs indicators toward 2030 goals, including chronic diseases mortality rates, suicide mortality rate, and road traffic accidents within BRI countries [20–22]. Again, these analyses are valuable but the assessments have not been examined systematically. A comprehensive and rigorous evaluation of this progress in the BRI countries would form the basis for understanding and following the potential of BRI to improve global health and reduce global health disparities.

Thus, this study used data from the Global Burden of Disease 2021 (GBD 2021) to compare health disparities between BRI and non-BRI countries, examine trends and variations in key health indicators by income level and region within BRI countries, and project ten key SDGs indicators of all BRI countries by 2030.

## Methods

### Data sources

The GBD 2021 synthesizes multiple data sources including censuses, household surveys, civil registration, vital statistics, and other sources, to estimate multiple health outcome indicators for each country or territory in each year by sex and age group. GBD 2021 estimates can be accessed through the Global Health Data Exchange (GHDx), which provides information on mortality and disability of 371 diseases and injuries from 204 countries and territories from 1990 to 2021 (https://vizhub.healthdata.org/gbd-results/) [23]. Details of GBD data extraction and methodology have been reported previously [24].

Health outcome indicators for each of the 149 BRI countries were obtained from the GBD 2021 online estimates. We extracted country-specific data on deaths, disability-adjusted life years (DALYs), and corresponding age-standardized rates. Additionally, we acquired data on 10 key disease-specific health indicators outlined in the SDGs with specific 2030 development targets. Non-BRI countries were defined as the 55 countries and regions not included in the BRI countries by the GBD 2021; their data were also extracted to compare with the results of BRI countries.

### Included countries

This study included 149 BRI countries that signed cooperation documents as part of the initiative by early 2022, recognizing some had joined only fairly recently. We use the term “China” to refer only to mainland China. To assess the impact of economic development on health, we grouped the BRI countries into four categories according to the World Bank’s 2021 per capita Gross National Income (GNI): (1) low-income countries (LICs), with a per capita GNI of $1085 and lower; (2) lower-middle-income countries (LMICs), with a per capita GNI between $1086 and $4255; (3) upper-middle-income countries (UMICs), with a per capita GNI between $4256 and $13,205; and (4) high-income countries (HICs), with a per capita GNI higher than $13,205 (https://datahelpdesk.worldbank.org/knowledgebase/articles/906519) [25].

Venezuela, the Cook Islands, and Niue lacked income data and were therefore excluded from the World Bank country classification, so we included a total of 26 LICs, 48 LMICs, 38 UMICs, and 34 HICs in data analysis.

In addition, we compared the burden of disease between BRI and non-BRI countries. Non-BRI countries included the 55 nations and regions not participating in the BRI among the 204 countries and regions included in GBD 2021.

### Measures of disease burden

DALYs were calculated by the sum of the years of life lost (YLLs) and years of healthy life lost due to disability (YLDs). YLLs were calculated by the longest possible life expectancy for a person subtracting the age at death. Prevalence counts multiplied by disability weights were used to calculate YLDs [26].

We determined 10 key health-related SDGs indicators to include using two criteria: (1) having an explicit and quantifiable target by 2030 [27]; and (2) data were available in the free-access GBD 2021 estimates. The operational definitions and 2030 targets for the 10 indicators are listed in Table 1 [28].

**Table 1:** Operational definitions and 2030 targets for the 10 included SDGs indicators

Operational definition of health indicator	2030 target
Maternal deaths per 100,000 livebirths in females aged 10–54 years	< 70 deaths
Probability of dying before age 5 years per 1000 live births	≤ 25 deaths
Probability of dying during the first 28 days of life per 1000 live births	≤ 12 deaths
Age-standardized HIV incidence rate per 1000 population	≤ 0.005
Age-standardized rate of tuberculosis cases (/100,000 population)	≤ 0.5
Age-standardized malaria incidence rate (/1000 population)	≤ 0.005
Age-standardized overall prevalence of 15 neglected tropical diseases (NTDs)* (%)	≤ 0.5%
Age-standardized mortality rate due to cardiovascular disease, cancer, diabetes, and chronic respiratory disease in population aged 30–70 years (/100,000 population)	reduce by 1/3^†^
Age-standardized mortality rate due to self-harm (/100,000 population)	reduce by 1/3^†^
Age-standardized mortality rate due to road injuries (/100,000 population)	reduce by 1/2^†^

^*^The 15 neglected tropical diseases include leprosy, chagas disease, leishmaniasis, African trypanosomiasis, schistosomiasis, cysticercosis, cystic echinococcosis, lymphatic filariasis, onchocerciasis, trachoma, dengue, guinea worm disease, rabies, intestinal nematode infections, and food-borne trematodiases

^†^2030 values were estimated using 2015 data for each country as a baseline

### Statistical analysis

We first plotted line chart to compare the number of deaths, number of DALYs, death rate (/100,000 population), and DALY rate (/100,000 population) between BRI countries and non-BRI countries during 1990–2021. Since GBD 2021 does not provide the aggregate estimates of BRI countries as a single entity, we used country-specific data to calculate the overall number of deaths, DALYs, and corresponding age-standardized rates of the 149 BRI countries and 55 non-BRI countries, respectively. GBD 2021 uses the GBD-estimated world population as a reference population to calculate age-standardized death rates and age-standardized DALY rates [29].

We plotted line chart of the age-standardized death rate and DALY rate for 2021, and calculated the estimated annual percentage change (EAPC) in age-standardized death rate and DALY rate between 1990 and 2021 to demonstrate geographic variations across the 149 BRI countries. We also plotted line charts to demonstrate the number of deaths, number of DALYs, death rate, and DALY rate across the four country types by income. We reported the names and age-standardized death rate and DALY rate for the top five diseases in each BRI country in 1990 and in 2021.

Because data for four indicators (under-five mortality rate, neonatal mortality rate, NTDs prevalence rate, and NCD mortality rate) were unavailable in the GHDx online dataset, we calculated their values according to the SDGs definitions.

Following previous research [30–32], we adopted a log-linear regression model to examine changes in the age-standardized death rate and DALY rate, and 10 key health-related SDGs indicators during 1990–2021. Log-linear regression models assume that the natural logarithm of age-standardized death rate can be represented as a formula (1).

1 $${\text{Ln}}\left( p \right) = \alpha + \beta x + \varepsilon$$

where $p$ represents the age-standardized rate, $\beta$ represents the changing speed of age-standardized rate over year, *x* denotes the calendar year, and $\varepsilon$ is the error term. When the model and $\beta$ are statistically significant, we calculate EAPC to quantify the change in the age-standardized rate over the study period through formula (2).

2 $${\text{EAPC}} = 100 \times \left[ {\exp \left( \beta \right) - 1} \right]$$

where “EAPC > 0” indicates that the age-standardized rate increases significantly, and “EAPC ≤ 0” suggests that the age-standardized rate decreases significantly or does not change significantly [30].

Given the impact of the COVID-19 pandemic on both fatal and non-fatal outcome estimations in 2020–2021, we decided to use data from 1990 to 2019 to project the estimates by 2030 [24]. Following previous research [33], we used an adjusted GBD method that assigned more weight to recent years to calculate the weighted mean of annual rate of change and project the NCD mortality values by 2030.

For the NCD mortality value $\left(V\right)$, we first transformed the value using the natural logarithm and calculated the annual change rate for every location $\left(l\right)$ and each year ($year$=1991, 1992, …, 2019) using the following formula (3):

3 $$R_{l,year} = \frac{{\ln \left( {V_{l,year} } \right) - \ln \left( {V_{l,year - 1} } \right)}}{{\ln \left( {V_{l,year - 1} } \right)}}$$

Then, we computed the weighted mean of the annual change rate over time based on formula (4), where ${weight}_{year}$ was determined by the parameter $\omega$, scaled to sum to 1, and then assigned greater weight to more recent years:

4 $$Weight_{year} = \frac{{\left( {year - 1990} \right)^{\omega } }}{{\mathop \sum \nolimits_{t = 1991}^{2019} \left( {t - 1990} \right)^{\omega } }}$$

where $year$ was the selected year, and $\omega$ was determined through a validity test [28].

Finally, we applied formula (5) to compute the weighted mean annual rate of change of NCD mortality rate for each country to project the attainment by 2030.

5 $$W_{l} = \mathop \sum \limits_{t = 1991}^{2019} R_{l,year} \times Weight_{year}$$

We obtained the 2030 projection data for road traffic mortality rate and suicide mortality rate from GBD foresight visualization (https://vizhub.healthdata.org/gbd-foresight/) [34], and the remaining seven indicators from the health-related SDGs visualizations (https://vizhub.healthdata.org/sdg/) [35], which were provided by the GBD 2021 study group.

All statistical analyses were performed with R version 4.4.1, and the charts were created with R and Microsoft Excel.

## Results

### Deaths and DALYs

**Fig. 1:**
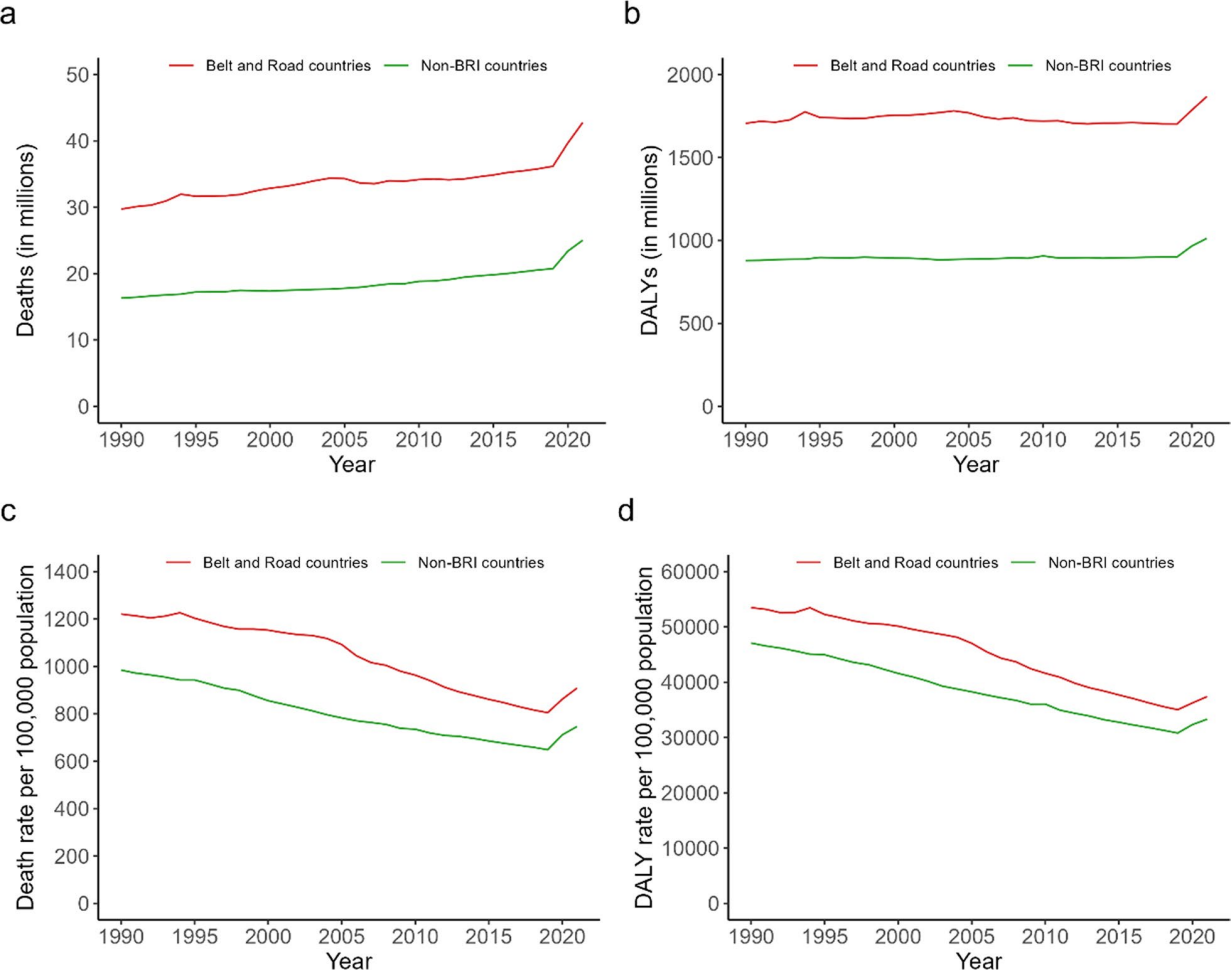
Number and age-standardized rate of deaths and DALYs for 149 BRI countries and for non-BRI countries, 1990–2021 (**a** number of deaths; **b** number of DALYs; **c** death rate; **d** DALY rate). *Note* DALY: disability-adjusted life year

Between 1990 and 2021, the number of deaths and DALYs in the BRI countries accounted for 62.9–66.0% and 64.8–66.8% of global deaths and DALYs, respectively (Online Appendix Table 2). Both the number and rate of deaths and of DALYs were higher in BRI countries than in non-BRI countries during the study time period (Fig. 1a-d). The number of deaths and DALYs increased by 43.9% and 9.5% for BRI countries between 1990 and 2021, respectively, percentages that were similar to 53.3% and 15.3% in non-BRI countries (Fig. 1a, b). In contrast, the age-standardized death rate and DALY rate significantly decreased by 1.45% (95% CI − 1.61% to − 1.30%) and by 1.50% (95% CI − 1.62% to − 1.38%) in BRI countries, and significantly dropped by 1.34%, (95% CI − 1.48% to − 1.20%) and 1.41% (95% CI − 1.49% to − 1.33%) in non-BRI countries (Fig. 1c, d).

### Deaths and DALYs by country income

**Fig. 2:**
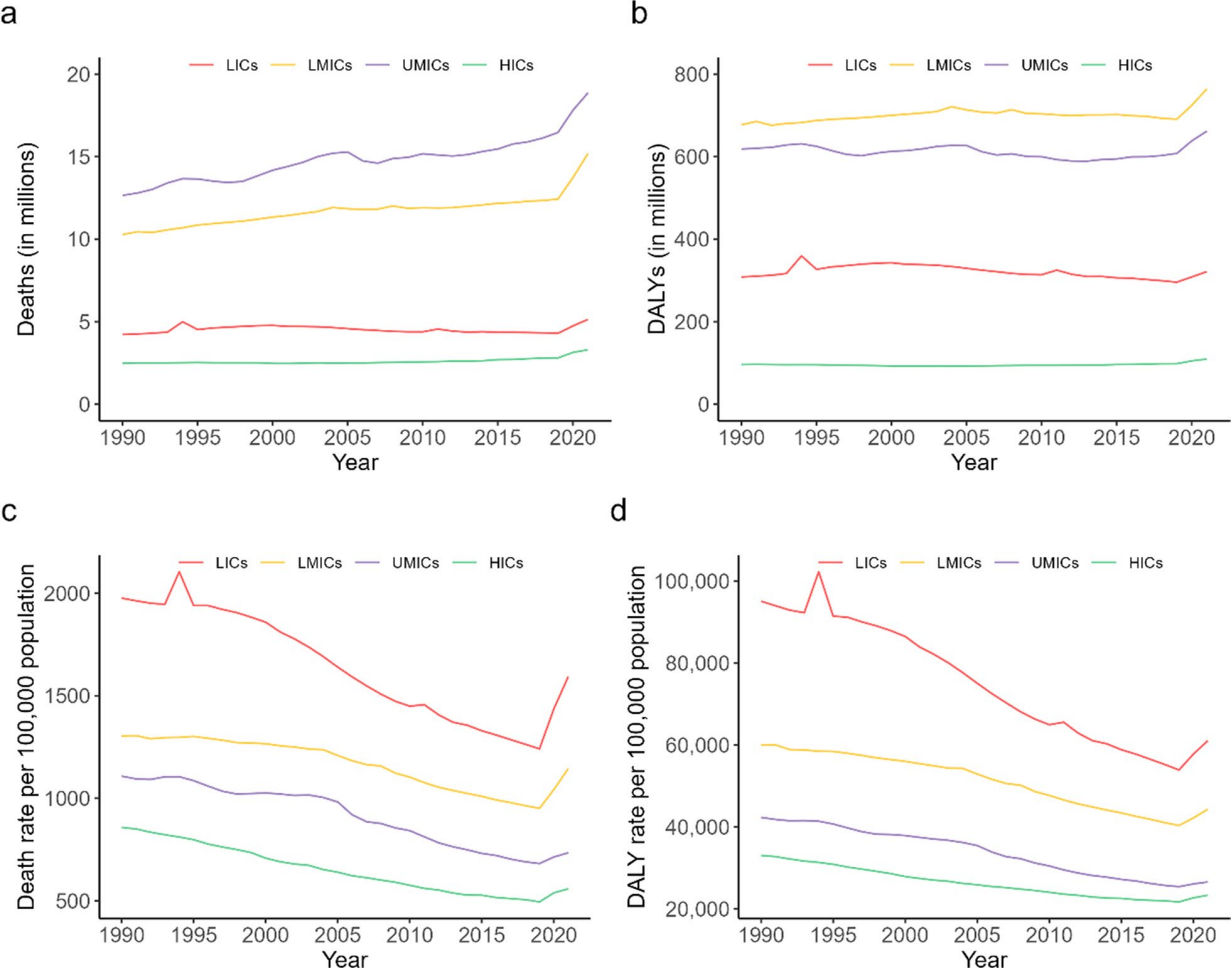
Number and age-standardized rate of deaths and DALYs for different income countries, 1990–2021 (**a** number of deaths; **b** number of DALYs; **c** death rate; **d** DALY rate). *Note* a. DALY, disability-adjusted life year; b. LICs, low-income countries; LMICs, lower middle-income countries; UMICs, upper middle-income countries; HICs, high-income countries

Between 1990 and 2021, the number of deaths in the BRI countries increased from 29.7 million to 42.8 million (Online Appendix Table 3), and the number of DALYs increased from 1705.6 million to 1867.7 million (Online Appendix Table 4). UMICs and LMICs consistently had the greatest number of deaths and DALYs, while HICs had the smallest numbers, respectively. The largest increases occurred in UMICs (49.10% increase in deaths number), and HICs (13.90% increase in DALYs number) (Fig. 2a, b).

Between 1990 and 2021, the age-standardized death rate in the BRI countries decreased from 1220.8 to 908.6 per 100,000 population (Online Appendix Table 3), and the age-standardized DALY rate decreased from 53,487.4 to 37,407.7 per 100,000 population (Online Appendix Table 4). LICs consistently had the highest age-standardized death and DALY rate, while HICs had the lowest. The greatest EAPC decrease occurred in HICs for age-standardized death rate (− 1.83%, 95% CI − 1.97% to − 1.69%) and in LICs for age-standardized DALY rate (− 2.03%, 95% CI − 2.20% to − 1.86%) (Fig. 2c, d).

### Geographic variations in deaths and DALYs

**Fig. 3:**
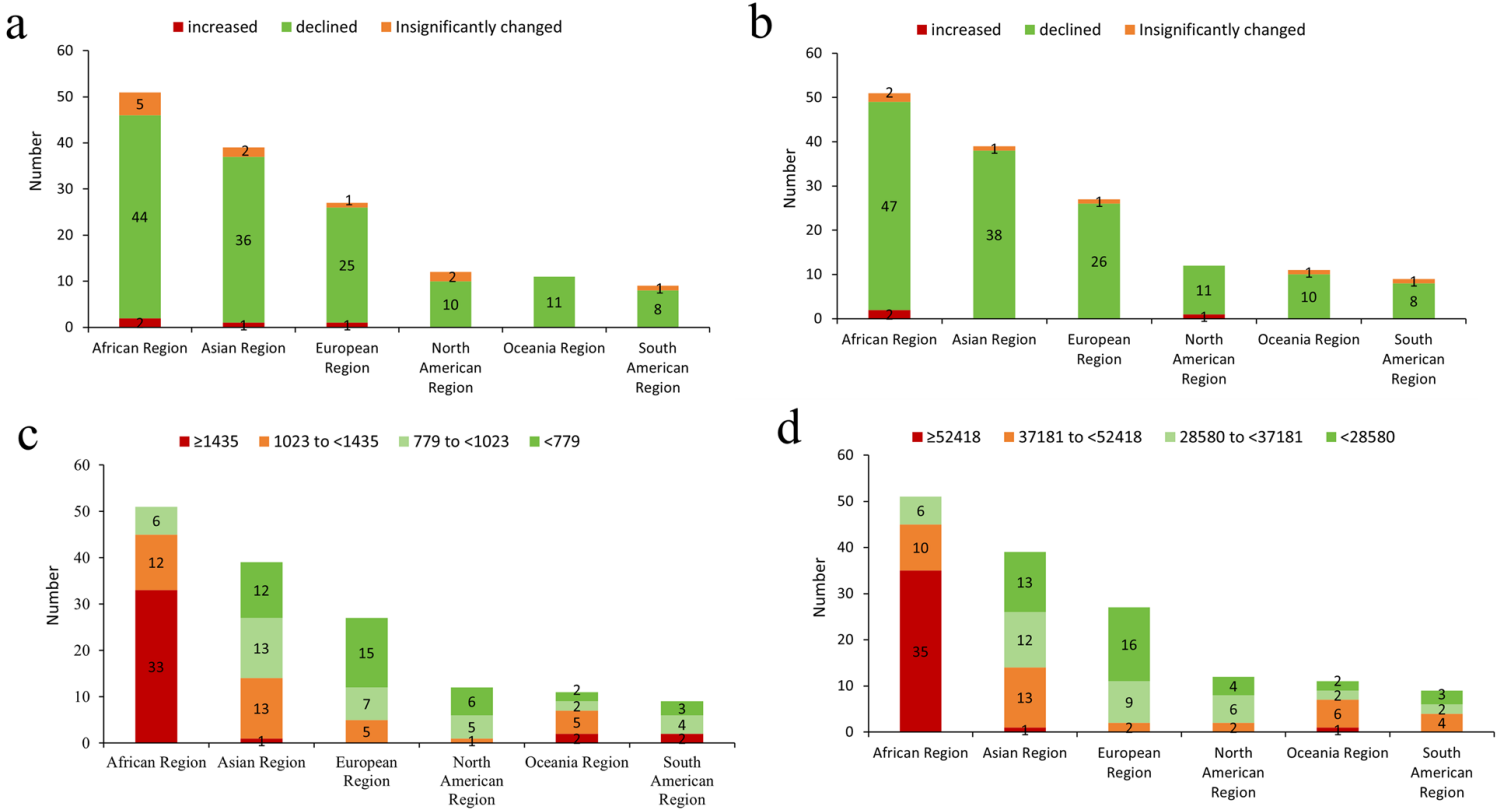
Number of age-standardized death and DALY rates by geographical region between 1990 and 2021 in 149 BRI countries (**a** EAPC in death rate; **b** EAPC in DALY rate; **c** age-standardized death rate in 2021; **d** age-standardized DALY rate in 2021). *Notes* **a**. The four categories of age-standardized death and DALY rates in the panels **c** and **d** were determined based on quintiles; **b**. BRI: Belt and Road Initiative; c. DALYs: disability-adjusted life year

Among the 149 BRI countries, the age-standardized death rate declined between 1990 and 2021 in 134 countries (89.9%), did not change significantly in 11 countries (7.4%; mostly from African countries), and increased in four countries (2.7%; Montenegro, Libya, United Arab Emirates, and Lesotho) (Fig. 3a and Online Appendix Table 5). The fastest declines occurred in Rwanda (− 3.92%, 95% CI − 4.96% to − 2.87%), the Republic of Korea (− 3.29%, 95% CI − 3.41% to − 3.17%) and Maldives (− 3.21%, 95% CI − 3.42% to − 2.99%). The greatest increases occurred in Lesotho (2.08%, 95% CI 1.39% to 2.77%), Libya (0.83%, 95% CI 0.62% to 1.05%), and Montenegro (0.71%, 95% CI 0.39% to 1.03%) (Online Appendix Table 5).

The change in age-standardized DALY rate between 1990 and 2021 was relatively consistent across geography (Fig. 3b). The largest decreases occurred in Rwanda (-4.30%, 95% CI -5.48% to -3.11%), Ethiopia (-3.34%, 95% CI -3.60% to -3.08%), and Burundi (-3.08%, 95% CI -3.55% to -2.60%). Three countries experienced DALY rate increases: Lesotho (1.62%, 95% CI 0.92% to 2.33%), Libya (0.39%, 95% CI 0.16% to 0.61%), and Dominica (0.20%, 95% CI 0.02% to 0.38%) (Online Appendix Table 6).

In 2021, the age-standardized death rate varied greatly across the 149 BRI countries. Most African countries had extremely high rates (highest in Lesotho, 3,336 per 100,000 population), while some European countries and a few Asian countries had comparatively low rates (lowest in Singapore, 292 per 100,000 population) (Fig. 3c and Online Appendix Table 5). The age-standardized DALY rate across the 149 BRI countries showed similar variations across geography, with the highest rate in Lesotho (117,885 per 100,000 population) and the lowest in Singapore (14,980 per 100,000 population) (Fig. 3d and Online Appendix Table 6).

The five leading causes of age-standardized death rate and DALY rate were generally similar across the 149 BRI countries in both 1990 and 2021 (Online Appendix Table 7, 8). In 2021, stroke was the leading cause of death and the leading cause of DALYs in 148 countries and 113 countries, respectively. Ischemic heart disease, COVID-19, lower respiratory infections, diabetes mellitus, and chronic kidney disease were the leading cause of death in 139, 138, 64, 43, and 37 countries in 2021, respectively. COVID-19, ischemic heart disease, neonatal disorders, diabetes mellitus, lower respiratory infections were the leading cause of death in 133, 109, 91, 56, and 51 countries in 2021, respectively.

### Changes in ten health-related SDGs indicators

**Fig. 4:**
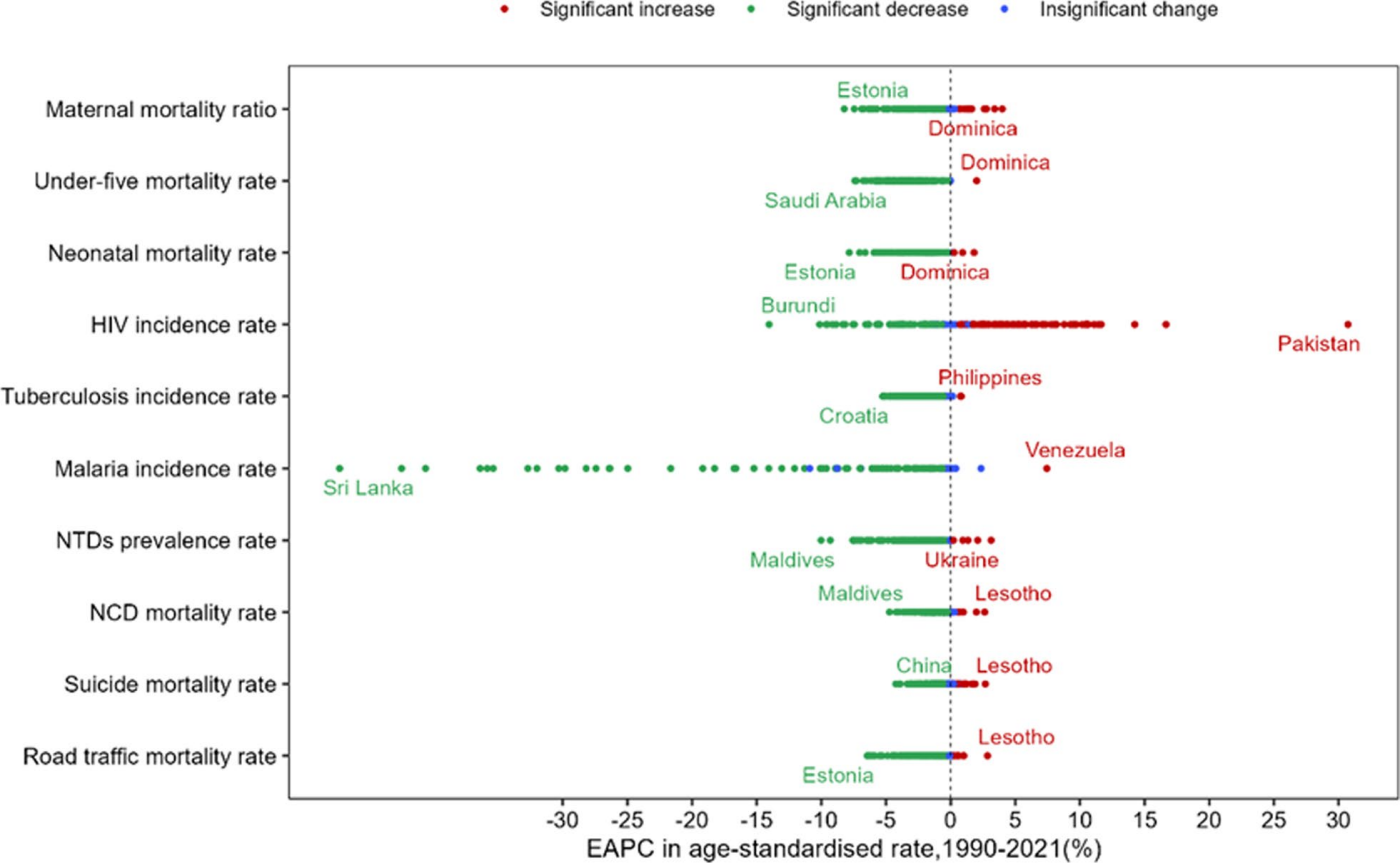
The estimated annual percentage change (EAPC) for 10 key health-related SDGs indicators specifying a concrete target in 149 BRI countries from 1990 to 2021. *Notes* **a**. Data points denote the BRI countries having the extreme high and low values of EAPC for each key health-related SDGs indicator; **b** SDGs: Sustainable Development Goals; **c** BRI: Belt and Road initiative

The EAPC for each of the ten health-related SDGs indicators varied largely across the 149 BRI countries between 1990 and 2021 (Fig. 4). Fifteen countries experienced significant increases between 1990 and 2021 in maternal mortality ratio (mostly African and North American countries). Corresponding increases for the other nine health-related SDGs outcomes were: 1 country for under-five mortality rate (Dominica), 3 for neonatal mortality rate (Dominica, Brunei Darussalam and Zimbabwe), 78 for HIV incidence rate (mostly Asian and European countries), 2 for tuberculosis incidence rate (Lesotho and Philippines), 1 for malaria incidence rate (Venezuela), 5 for NTDs prevalence rate (mostly European countries), 7 for NCD mortality rate (mostly African countries), 21 for suicide mortality rate (mostly African countries), and 7 for road traffic mortality rate (mostly African countries). Accordingly, 121, 147, 143, 58, 143, 78, 136, 130, 108, and 130 countries, respectively, witnessed significant decreases in the ten health indicators (Online Appendix Tables 9, 10, 11, 12, 13, 14, 15, 16, 17 and 18).

### Target achievement of ten health-related SDGs indicators

**Fig. 5:**
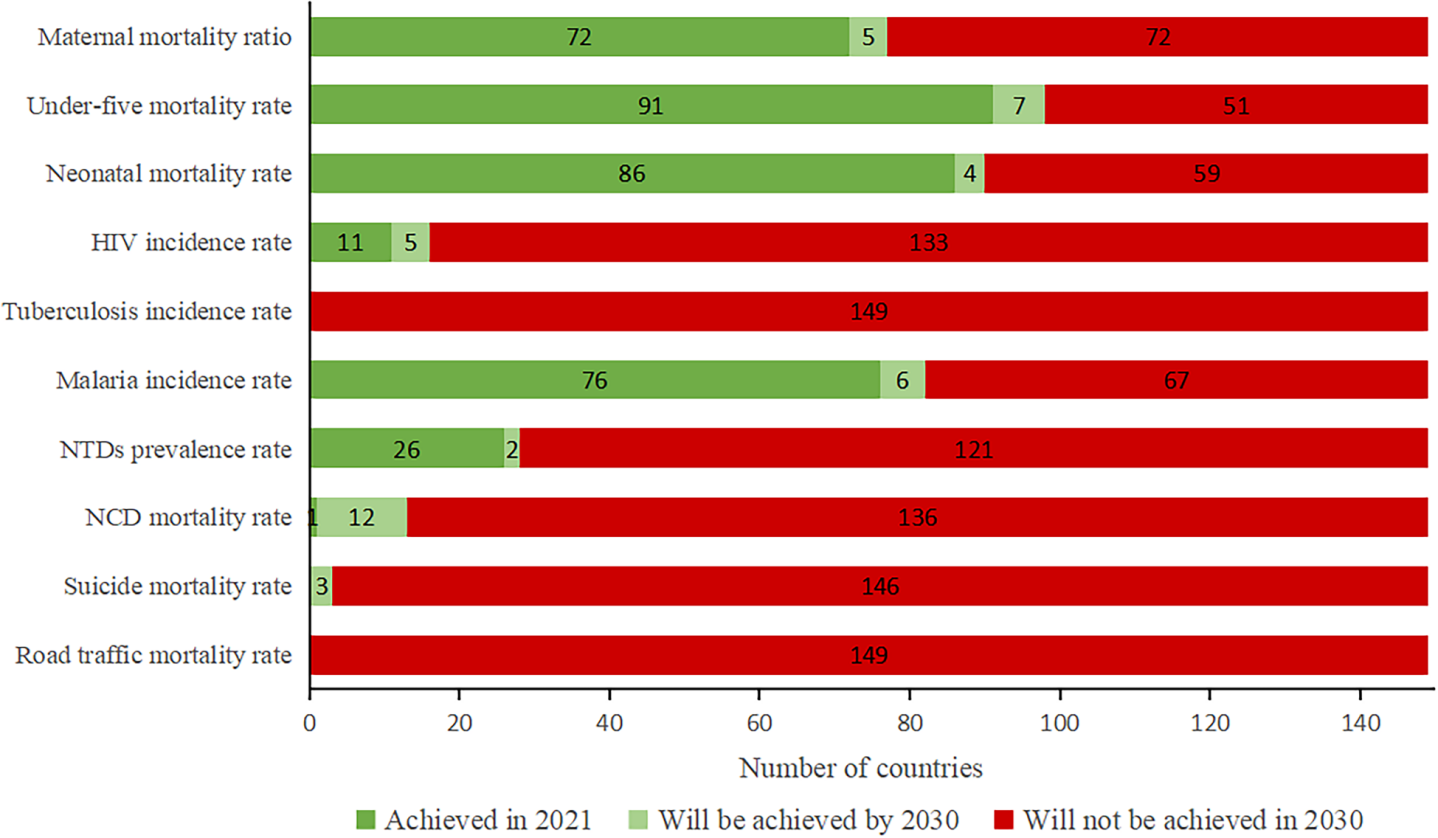
Number among 149 BRI countries that have achieved the specified targets for the 10 key health-related SDGs in 2021, or are projected to achieve the targets by 2030. *Note* BRI: Belt and Road initiative

Based on GBD 2021 estimates and our projections, four health-related SDGs indicators were either achieved in 2021 or are projected to reach their target by 2030 in over half the BRI countries—most from UMICs and HICs in Asia and Europe: 98 countries (66%) for under-five mortality rate, 90 countries (61%) for neonatal mortality rate, 82 countries for malaria incidence rate (55%), and 77 countries (52%) for maternal mortality ratio (Fig. 5 and Online Appendix Tables 19, 20, 21, 22, 23, 24, 25, 26, 27 and 28). In contrast, we projected that the 2030 targets will not be reached in over 70% of BRI countries for the remaining six health-related SDGs indicators. Notably, no BRI countries are projected to achieve the SDGs targets for tuberculosis incidence rate or road traffic mortality rate by 2030.

## Discussion

### Principal findings

This study characterized the burden of disease in the 149 BRI countries between 1990 and 2021. We generated four key findings. First, the number of deaths and DALYs in the BRI countries represented 62.9–66.0% and 64.8–66.8% of global deaths and DALYs between 1990 and 2021, and both the overall age-standardized death rate and DALY rate remained higher in BRI countries than in non-BRI countries throughout the study time period. Similar to non-BRI countries, BRI countries witnessed substantial decreases in age-standardized death rate and DALY rate. Second, great variations existed across the 149 BRI countries both for level of and changes in age-standardized death rate and DALY rate, LICs consistently had the highest age-standardized death rate and DALY rate, but had the greatest decrease in age-standardized DALY rate between 1990 and 2021. Rates were highest in African countries, and were lowest in several European and Asian countries. With a few exceptions, most BRI countries had similar leading causes of death and DALYs, e.g., stroke, COVID-19, and ischemic heart disease in 2021. Third, the 149 BRI countries demonstrated varying changes in each of the 10 health-related SDGs indicators from 1990 to 2021. Most countries experienced significant decreases in the ten indicators, but some countries witnessed substantial increases in a few indicators, such as HIV incidence rate and suicide mortality rate. Last, we project the 2030 targets for 6 health-related SDGs indicators will not be reached in over 70% of the BRI countries. No BRI countries will achieve the 2030 targets for tuberculosis incidence rate or road traffic mortality rate.

### Interpretation of findings

#### Disease burden gaps between BRI countries and non-BRI countries

The heavier disease burden in BRI countries compared to non-BRI countries is likely explained by lagging social and economic development in many BRI countries. Ninety percent of the world’s LICs and LMICs participate in the BRI. Seven of ten countries with the greatest number of children globally who do not receive the routine three doses of diphtheria–tetanus–pertussis-containing vaccines (DTP) are from the BRI countries [34]. Despite the BRI's primary aim to promote economic development in LIC and LMIC countries, many BRI countries still have regions with substantial populations of impoverished individuals lacking proper sanitation, safe water, and adequate health investments, medical facilities, or health professionals [36, 37]. Consequently, many BRI countries suffer from high mortality and morbidity rates for many diseases [38, 39].

The substantial decreases in overall age-standardized death rates and DALY rates in BRI countries between 1990 and 2019 was associated with increased governmental investment and with health development assistance from developed country governments and international aid organizations [40]. GBD 2019 estimates suggest the Socio-Demographic Index (SDI) improved substantially in many BRI countries between 1990 and 2019 [29]. However, these improvements should be couched, obviously, in the large increases of overall age-standardized death rates and DALY rates in BRI countries during 2019 to 2021, likely due to the impact of COVID-19 pandemic [24], which affect BRI countries most dramatically where the health infrastructure was limited and vaccination rate was low.

Large disparities across countries in key health indicators, including age-standardized death and DALY rate, are likely attributable to poor access to adequate health care, political conflict [41] and/or extreme weather patterns [42] in certain African countries. The slow progress in health-related indicators we observed in several countries in North America and Oceania may be ascribed to the high prevalence of chronic non-communicable diseases and associated risk factors such as hypertension, hyperlipidemia, and smoking [43]. In contrast, the rapid decline in age-standardized DALY rate in low-income countries demonstrates significant health progress in many locations, likely the result of prevention efforts made by these countries and outside aid agencies [24, 40].

#### Leading cause of death rate and DALY rate

The top five leading causes of age-standardized death rate and DALY rate in BRI countries gradually shifted from communicable diseases to chronic non-communicable diseases over the past few decades [44]. COVID-19 became the third and first leading cause of death rate and DALY rate in 2021. The increased risk of ischemic heart disease and diabetes mellitus between 1990 and 2021 may be related to increased exposure to hypertension, high fasting plasma glucose and high BMI [45]. The rise of HIV/AIDS threat during 1990–2021 was primarily due to the HIV/AIDS morbidity and mortality increase in Eastern Europe and Central Asia, as well as the highest global HIV/AIDS prevalence rates in sub-Saharan Africa. Most countries in these regions participate in the BRI [46].

#### Past progress and future projections of health-related SDGs indicators

The varying change patterns among the 10 included health-related SDGs indicators between 1990 and 2021 can be ascribed to inconsistent exposures to major risk factors [47] and disparities in socio-economic development [48] and health care services [40]. Our findings generally match projections from the GBD 2017 study regarding achievement of 2030 health-related SDGs indicator targets [28]. Broad anticipated achievement of four health-related indicators (under-five mortality rate, neonatal mortality rate, malaria incidence rate, and maternal mortality ratio) likely reflects combined efforts of individual governments and international organizations [49]. However, it is notable that our estimates suggest six indicators will not be achieved in over 70% of BRI countries by 2030. That result likely reflects multiple converging factors: (1) the persistent presence of common risk factors, such as weak law enforcement leading to high rates of impaired driving, speeding, and failure to wear helmets and use seatbelts, which contribute to road traffic injuries [50]; (2) synergistic effects between different health indicators, for instance, HIV infections increase the risk of developing active tuberculosis and other infectious diseases [51]; (3) inadequate health service in many BRI countries, in sub-Saharan Africa, for example, at least one-sixth of the population lives far from medical facilities [52]; and (4) a high burden of infectious disease in some BRI countries, especially low-income African and Southeast Asian countries [53].

### Policy implications

Our findings have several implications for policymaking. First, our study comprehensively presents the quantitative burden of diseases in the 149 BRI countries as a whole for the first time, providing basic evidence to use the BRI infrastructure to implement a long-term health action plan that promotes health and progress toward health-related SDGs across the BRI network. Strengthening the capacities of national health care systems should be a priority for all low- and lower middle-income countries, and could be supported by the more economically prosperous countries in the BRI [54]. Efforts might include developing, generalizing and implementing effective and easy-to-implement health-promoting interventions and training of health personnel [55, 56]. For example, the BRI might establish education or training programs to alleviate the shortage of health-related human resources in low- and middle-income BRI countries, and especially in rural parts of those countries.

Second, to reduce health gaps between BRI countries and non-BRI countries and across the 149 BRI countries, it is critically urgent to initiate and extend existing health collaborations and aid programs within less prosperous BRI countries. A series of actions could reduce health inequities across BRI countries, including building collaborative health initiatives between wealthy and poor BRI countries, building health infrastructure in lower income BRI countries, raising health financing, and offering health assistance for vulnerable countries [57].

Last, to achieve health indicator targets by 2030, it is important for BRI countries to adopt proven interventions such as TB Control Programs [58] and HIV prevention programs [59]. The BRI network can and should assist with these initiatives.

### Study limitations

This study has some limitations. First, our findings rely on the quality of GBD 2021 database. Although the GBD study group synthesized multiple data sources and used sophisticated models to address the challenges of poor raw data quality in some locations, their estimates cannot fully overcome this challenge. Because the GBD 2021 study group calculated the 95% uncertainty intervals (UIs) through 1000 repeated draws, they therefore do not provide the 95% UIs of estimates for specific purposes [24, 29]. As a result, we cannot present the 95% UIs for several indicators for specific analytic purposes. Second, we provide only the general burden of disease in BRI countries and did not further analyze the data by gender, or other health-related indicators without specific targets. Third, the projection for NCD mortality rate was derived using the weighted mean of annual rate of change, which methodologically do not allow for the calculation of 95% confidence intervals (95% CIs). The estimates for other seven health-related SDGs indicators provided by the GBD 2021 do not give the 95% CIs or 95% UIs [35]. The projection methodology relied solely on historical trajectories and did not incorporate demographic factors, health risk factors, or socioeconomic data into the models. Therefore, our projected findings might be sensitive to key factors such as health policy. Last, although some countries joined the BRI only in recent years, we included them in full analysis examining trends during 1990–2021. Similarly, although country classifications by income might change in particular countries between 1990 and 2021 according to the World Bank data, we used 2021 data to define income categories, following previous research [60]. Such strategies could impact our results.

## Conclusions

In conclusion, we sought to address two research questions. First, we examined changes in major health indicators and gaps across the 149 BRI countries and between those 149 BRI countries and 55 non-BRI countries. This analysis found that the overall death and DALYs burden in the 149 BRI countries decreased substantially during 1990–2021 but remained higher than that of non-BRI countries. Further, large variations existed across the 149 BRI countries in the level and changes of primary health outcomes. Our second research goal was to project whether the 2030 targets of 10 key health-related SDGs indicators will be achieved in the BRI countries. We found that over 70% of BRI countries will not reach the 2030 targets for six SDGs indicators based on progress over the past three decades.

Our results highlight the need for government officials and policymakers across the BRI network to capitalize on the BRI economic infrastructure to accomplish a series of actions that promote the overall health of BRI countries, reduce health inequities across the 149 countries, narrow the gaps in comparison to non-BRI countries, and accelerate the speed of health-related development in the BRI member states. These strategies might include establishing long-term health collaboration and aid programs and training health-related human resources in low-income and middle-income BRI countries.

## Supplementary Information

Additional file 1.

## Data Availability

Datasets used in the study are available from the Global Health Data Exchange (GHDx): http://ghdx.healthdata.org/gbd-results-tool.
