# Supplementary material for: Burden of disease in the Belt and Road countries from 1990 to 2021: analysis of estimates from the Global Burden of Disease 2021
Source: Glob Health Res Policy. 2025 May 1;10:20. doi: 10.1186/s41256-025-00403-3 (PMC12046647; doi:10.1186/s41256-025-00403-3)
Supplement: Supplementary file 1 — Additional file 1. [file 41256_2025_403_MOESM1_ESM.docx]

Appendix: supplementary results to “Burden of disease in the Belt and Road countries from 1990 to 2021: Analysis of estimates from the Global Burden of Disease 2021”

Tables

[Supplementary Table 1: The basic information of 149 BRI countries 1](#_Toc194952450)

[Supplementary Table 2: The number of Deaths and DALYs, 1990–2021 4](#_Toc194952451)

[Supplementary Table 3: Deaths and age-standardized death rate in 1990 and in 2021 5](#_Toc194952452)

[Supplementary Table 4: DALYs, and age-standardized DALY rate in 1990 and in 2021 5](#_Toc194952453)

[Supplementary Table 5: Age-standardized death rate between 1990 and 2021 6](#_Toc194952454)

[Supplementary Table 6 : Age-standardized DALY rate between 1990 and 2021 12](#_Toc194952455)

[Supplementary Table 7: Top five causes of age-standardized mortality in 1990 and in 2021 18](#_Toc194952456)

[Supplementary Table 8: Top five causes of age-standardized DALY rate in 1990 and in 2021 48](#_Toc194952457)

[Supplementary Table 9: Maternal mortality ratio between 1990 and 2021 77](#_Toc194952458)

[Supplementary Table 10: Under-five mortality rate between 1990 and 2021 83](#_Toc194952459)

[Supplementary Table 11: Neonatal mortality rate between 1990 and 2021 89](#_Toc194952460)

[Supplementary Table 12: HIV incidence rate between 1990 and 2021 95](#_Toc194952461)

[Supplementary Table 13: Tuberculosis incidence rate between 1990 and 2021 101](#_Toc194952462)

[Supplementary Table 14: Malaria incidence rate between 1990 and 2021 107](#_Toc194952463)

[Supplementary Table 15: NTDs prevalence rate between 1990 and 2021 114](#_Toc194952464)

[Supplementary Table 16: NCD mortality rate between 1990 and 2021 120](#_Toc194952465)

[Supplementary Table 17: Suicide mortality rate between 1990 and 2021 126](#_Toc194952466)

[Supplementary Table 18: Road traffic mortality rate between 1990 and 2021 132](#_Toc194952467)

[Supplementary Table 19. Achievement of SDGs for maternal mortality ratio 138](#_Toc194952468)

[Supplementary Table 20. Achievement of SDGs for under-five mortality rate 144](#_Toc194952469)

[Supplementary Table 21. Achievement of SDGs for neonatal mortality rate 150](#_Toc194952470)

[Supplementary Table 22. Achievement of SDGs for HIV incidence rate 156](#_Toc194952471)

[Supplementary Table 23. Achievement of SDGs for tuberculosis incidence rate 162](#_Toc194952472)

[Supplementary Table 24. Achievement of SDGs for malaria incidence rate 168](#_Toc194952473)

[Supplementary Table 25. Achievement of SDGs for NTDs prevalence 174](#_Toc194952474)

[Supplementary Table 26. Achievement of SDGs for NCD mortality rate 180](#_Toc194952475)

[Supplementary Table 27. Achievement of SDGs for suicide mortality rate 186](#_Toc194952476)

[Supplementary Table 28. Achievement of SDGs for road traffic mortality rate 192](#_Toc194952477)

| Supplementary Table 1: The basic information of 149 BRI countries | | |
| --- | --- | --- |
| Country | Region | Country income |
| China | Asia | Upper middle income |
| Kyrgyzstan | Asia | Lower middle income |
| Qatar | Asia | High income |
| Sri Lanka | Asia | Lower middle income |
| Kazakhstan | Asia | Upper middle income |
| Belarus | Europe | Upper middle income |
| Ukraine | Europe | Lower middle income |
| Georgia | Asia | Upper middle income |
| Armenia | Asia | Upper middle income |
| North Macedonia | Europe | Upper middle income |
| Hungary | Europe | High income |
| Uzbekistan | Asia | Lower middle income |
| Tajikistan | Asia | Lower middle income |
| South Korea | Asia | High income |
| Turkey | Asia | Upper middle income |
| Bulgaria | Europe | Upper middle income |
| Czechia | Europe | High income |
| Poland | Europe | High income |
| Serbia | Europe | Upper middle income |
| Slovakia | Europe | High income |
| Azerbaijan | Asia | Upper middle income |
| Iraq | Asia | Upper middle income |
| Saudi Arabia | Asia | High income |
| Egypt | Africa | Lower middle income |
| Iran | Asia | Lower middle income |
| Afghanistan | Asia | Low income |
| Laos | Asia | Lower middle income |
| Cambodia | Asia | Lower middle income |
| Bangladesh | Asia | Lower middle income |
| Madagascar | Africa | Low income |
| New Zealand | Oceania | High income |
| Russia | Europe | Upper middle income |
| Albania | Europe | Upper middle income |
| Bosnia and Herzegovina | Europe | Upper middle income |
| Croatia | Europe | High income |
| Malaysia | Asia | Upper middle income |
| Mongolia | Asia | Lower middle income |
| Montenegro | Europe | Upper middle income |
| Myanmar | Asia | Lower middle income |
| Nepal | Asia | Lower middle income |
| Pakistan | Asia | Lower middle income |
| Romania | Europe | High income |
| Singapore | Asia | High income |
| Timor-Leste | Asia | Lower middle income |
| Thailand | Asia | Upper middle income |
| Lebanon | Asia | Lower middle income |
| Brunei | Asia | High income |
| Vietnam | Asia | Lower middle income |
| Panama | North America | High income |
| Morocco | Africa | Lower middle income |
| Latvia | Europe | High income |
| Moldova | Europe | Upper middle income |
| Estonia | Europe | High income |
| Lithuania | Europe | High income |
| Slovenia | Europe | High income |
| Maldives | Asia | Upper middle income |
| Cuba | North America | Upper middle income |
| Austria | Europe | High income |
| Trinidad and Tobago | North America | High income |
| Oman | Asia | High income |
| Antigua and Barbuda | North America | High income |
| Bolivia | South America | Lower middle income |
| Papua New Guinea | Oceania | Lower middle income |
| Bahrain | Asia | High income |
| Kuwait | Asia | High income |
| Sudan | Africa | Low income |
| Libya | Africa | Upper middle income |
| Tunisia | Africa | Lower middle income |
| Dominica | North America | Upper middle income |
| Niue | Oceania | / |
| Rwanda | Africa | Low income |
| Guyana | South America | Upper middle income |
| United Arab Emirates | Asia | High income |
| Uruguay | South America | High income |
| Greece | Europe | High income |
| Senegal | Africa | Lower middle income |
| Costa Rica | North America | Upper middle income |
| Algeria | Africa | Lower middle income |
| Angola | Africa | Lower middle income |
| Burundi | Africa | Low income |
| Côte d'Ivoire | Africa | Lower middle income |
| Cape Verde | Africa | Lower middle income |
| Cameroon | Africa | Lower middle income |
| Chad | Africa | Low income |
| Republic of the Congo | Africa | Lower middle income |
| Djibouti | Africa | Lower middle income |
| Ethiopia | Africa | Low income |
| Gabon | Africa | Upper middle income |
| Gambia | Africa | Low income |
| Ghana | Africa | Lower middle income |
| Guinea | Africa | Low income |
| Kenya | Africa | Lower middle income |
| Mauritania | Africa | Lower middle income |
| Mozambique | Africa | Low income |
| Namibia | Africa | Upper middle income |
| Nigeria | Africa | Lower middle income |
| Seychelles | Africa | High income |
| Sierra Leone | Africa | Low income |
| Somalia | Africa | Low income |
| South Sudan | Africa | Low income |
| Togo | Africa | Low income |
| Uganda | Africa | Low income |
| Tanzania | Africa | Lower middle income |
| Zambia | Africa | Low income |
| Zimbabwe | Africa | Lower middle income |
| Venezuela | South America | / |
| Grenada | North America | Upper middle income |
| Suriname | South America | Upper middle income |
| Samoa | Oceania | Lower middle income |
| El Salvador | North America | Lower middle income |
| Chile | South America | High income |
| Dominican Republic | North America | Upper middle income |
| Malta | Europe | High income |
| Vanuatu | Oceania | Lower middle income |
| Fiji | Oceania | Upper middle income |
| Cook Islands | Oceania | / |
| Micronesia | Oceania | Lower middle income |
| Tonga | Oceania | Upper middle income |
| Indonesia | Asia | Lower middle income |
| Philippines | Asia | Lower middle income |
| South Africa | Africa | Upper middle income |
| Portugal | Europe | High income |
| Ecuador | South America | Upper middle income |
| Barbados | North America | High income |
| Italy | Europe | High income |
| Luxembourg | Europe | High income |
| Jamaica | North America | Upper middle income |
| Cyprus | Europe | High income |
| Equatorial Guinea | Africa | Upper middle income |
| Liberia | Africa | Low income |
| Peru | South America | Upper middle income |
| Yemen | Asia | Low income |
| Benin | Africa | Lower middle income |
| Comoros | Africa | Lower middle income |
| Lesotho | Africa | Lower middle income |
| Mali | Africa | Low income |
| Solomon Islands | Oceania | Lower middle income |
| Kiribati | Oceania | Lower middle income |
| Democratic Republic of the Congo | Africa | Low income |
| Botswana | Africa | Upper middle income |
| Central African Republic | Africa | Low income |
| Guinea-Bissau | Africa | Low income |
| Eritrea | Africa | Low income |
| Burkina Faso | Africa | Low income |
| Sao Tome and Principe | Africa | Lower middle income |
| Nicaragua | North America | Lower middle income |
| Syria | Asia | Low income |
| Argentina | South America | Upper middle income |
| Niger | Africa | Low income |

Note: BRI countries: the Belt and Road countries.

| Supplementary Table 2: The number of Deaths and DALYs, 1990–2021 | | | | | | | | |
| --- | --- | --- | --- | --- | --- | --- | --- | --- |
| Year |  | Number of Deaths (Millions) | | |  | Number of DALYs (Millions) | | |
|  |  | BRI countries | Global | Proportion (%) |  | BRI countries | Global | Proportion (%) |
| 1990 |  | 29.7 | 46.1 | 64.5 |  | 1705.6 | 2584.3 | 66.0 |
| 1991 |  | 30.1 | 46.5 | 64.7 |  | 1717.9 | 2598.0 | 66.1 |
| 1992 |  | 30.3 | 47.0 | 64.6 |  | 1712.5 | 2597.1 | 65.9 |
| 1993 |  | 30.9 | 47.7 | 64.8 |  | 1727.2 | 2613.7 | 66.1 |
| 1994 |  | 32.0 | 48.9 | 65.4 |  | 1775.0 | 2662.2 | 66.7 |
| 1995 |  | 31.7 | 48.9 | 64.8 |  | 1741.5 | 2638.8 | 66.0 |
| 1996 |  | 31.7 | 49.0 | 64.7 |  | 1738.8 | 2633.8 | 66.0 |
| 1997 |  | 31.7 | 49.0 | 64.7 |  | 1734.2 | 2628.4 | 66.0 |
| 1998 |  | 31.9 | 49.4 | 64.6 |  | 1735.8 | 2635.0 | 65.9 |
| 1999 |  | 32.5 | 49.9 | 65.1 |  | 1748.8 | 2644.6 | 66.1 |
| 2000 |  | 32.9 | 50.2 | 65.4 |  | 1754.9 | 2648.1 | 66.3 |
| 2001 |  | 33.1 | 50.6 | 65.5 |  | 1755.0 | 2647.9 | 66.3 |
| 2002 |  | 33.5 | 51.1 | 65.6 |  | 1761.4 | 2650.9 | 66.4 |
| 2003 |  | 34.0 | 51.6 | 65.9 |  | 1770.3 | 2653.3 | 66.7 |
| 2004 |  | 34.4 | 52.0 | 66.0 |  | 1781.1 | 2666.5 | 66.8 |
| 2005 |  | 34.3 | 52.1 | 65.9 |  | 1768.9 | 2656.6 | 66.6 |
| 2006 |  | 33.7 | 51.6 | 65.3 |  | 1744.5 | 2633.1 | 66.3 |
| 2007 |  | 33.5 | 51.7 | 64.8 |  | 1731.4 | 2622.6 | 66.0 |
| 2008 |  | 34.0 | 52.4 | 64.8 |  | 1739.0 | 2633.8 | 66.0 |
| 2009 |  | 33.9 | 52.4 | 64.7 |  | 1721.8 | 2614.3 | 65.9 |
| 2010 |  | 34.2 | 53.0 | 64.5 |  | 1719.4 | 2626.4 | 65.5 |
| 2011 |  | 34.3 | 53.2 | 64.5 |  | 1721.3 | 2615.8 | 65.8 |
| 2012 |  | 34.1 | 53.2 | 64.1 |  | 1707.0 | 2601.8 | 65.6 |
| 2013 |  | 34.3 | 53.7 | 63.8 |  | 1702.6 | 2598.6 | 65.5 |
| 2014 |  | 34.6 | 54.3 | 63.7 |  | 1707.5 | 2600.3 | 65.7 |
| 2015 |  | 34.9 | 54.7 | 63.8 |  | 1707.8 | 2602.7 | 65.6 |
| 2016 |  | 35.3 | 55.3 | 63.8 |  | 1710.3 | 2606.5 | 65.6 |
| 2017 |  | 35.5 | 55.8 | 63.6 |  | 1706.6 | 2605.4 | 65.5 |
| 2018 |  | 35.8 | 56.3 | 63.5 |  | 1702.6 | 2603.1 | 65.4 |
| 2019 |  | 36.2 | 56.9 | 63.6 |  | 1702.1 | 2602.9 | 65.4 |
| 2020 |  | 39.7 | 63.1 | 62.9 |  | 1786.7 | 2753.6 | 64.9 |
| 2021 |  | 42.8 | 67.8 | 63.1 |  | 1867.7 | 2880.6 | 64.8 |

BRI countries: the Belt and Road countries.

The proportion was calculated as the value for BRI countries divided by the global value *100%.

Supplementary Table 3: The number of deaths and age-standardized death rate in 1990 and in 2021 for the Belt and Road countries

| Country income | 1990 | |  | 2021 | |  | 1990-2021 |
| --- | --- | --- | --- | --- | --- | --- | --- |
|  | Number of deaths  (in million) | ASDR  (per 100,000 persons) |  | Number of deaths  (in million) | ASDR  (per 100,000 persons) |  | EAPC (95% CI) (%) |
| All BRI countries | 29.7 | 1220.8 |  | 42.8 | 908.6 |  | -1.45 (-1.61 to -1.30) |
| Low income | 4.2 | 1975.8 |  | 5.1 | 1593.9 |  | -1.58 (-1.82 to -1.35) |
| Lower middle income | 10.3 | 1303.4 |  | 15.2 | 1144.9 |  | -1.03 (-1.20 to -0.86) |
| Upper middle income | 12.7 | 1107.7 |  | 18.9 | 734.3 |  | -1.74 (-1.90 to -1.57) |
| High income | 2.5 | 858.0 |  | 3.3 | 558.5 |  | -1.83 (-1.97 to -1.69) |

Supplementary Table 4: The number of DALYs, and age-standardized DALY rate in 1990 and in 2021 for the Belt and Road countries

| Country income | 1990 | |  | 2021 | |  | 1990-2021 |
| --- | --- | --- | --- | --- | --- | --- | --- |
|  | DALYs  (in million) | Age-standardized DALY Rate (per 100,000 persons) |  | DALYs  (in million) | Age-standardized DALY rate (per 100,000 persons) |  | EAPC (95% CI) (%) |
| All BRI countries | 1705.6 | 53487.4 |  | 1867.7 | 37407.7 |  | -1.50 (-1.62 to -1.38) |
| Low income | 308.4 | 95049.9 |  | 321.4 | 61019.5 |  | -2.03 (-2.20 to -1.86) |
| Lower middle income | 677.1 | 60020.2 |  | 764.1 | 44290.4 |  | -1.35 (-1.47 to -1.24) |
| Upper middle income | 618.3 | 42278.1 |  | 661.8 | 26536.9 |  | -1.83 (-1.95 to -1.71) |
| High income | 96.2 | 33022.2 |  | 109.6 | 23306.2 |  | -1.40 (-1.51 to -1.29) |

BRI countries: the Belt and Road countries; ASDR: age-standardized death rate; EAPC: the estimated annual percentage cha

| Supplementary Table 5: The age-standardized death rate (/100,000 persons) in 149 BRI countries between 1990 and 2021 | | | | |
| --- | --- | --- | --- | --- |
| Country |  | 1990 | 2021 | EAPC during 1990-2021 (95% CI) (%) |
| Afghanistan |  | 1999.8 | 2030.7 | -0.94 (-1.25 to -0.63) |
| Albania |  | 852.4 | 754 | -0.82 (-1.12 to -0.51) |
| Algeria |  | 1046.2 | 957.1 | -0.85 (-1.10 to -0.59) |
| Angola |  | 2128.4 | 1699.8 | -1.76 (-2.04 to -1.49) |
| Antigua and Barbuda |  | 774.5 | 764 | -0.55 (-0.74 to -0.36) |
| Argentina |  | 830.9 | 676 | -0.82 (-0.92 to -0.72) |
| Armenia |  | 906.5 | 749.9 | -1.19 (-1.48 to -0.91) |
| Austria |  | 711.0 | 436.1 | -1.78 (-1.91 to -1.65) |
| Azerbaijan |  | 1051.9 | 1047.5 | -0.71 (-0.93 to -0.49) |
| Bahrain |  | 1351.5 | 1022.6 | -1.84 (-2.19 to -1.48) |
| Bangladesh |  | 1442.3 | 878.8 | -2.12 (-2.36 to -1.87) |
| Barbados |  | 783.6 | 677.1 | -0.68 (-0.77 to -0.59) |
| Belarus |  | 912.3 | 1032.2 | -0.84 (-1.21 to -0.47) |
| Benin |  | 1607.7 | 1394.9 | -1.03 (-1.17 to -0.88) |
| Bolivia (Plurinational State of) |  | 1342.0 | 1445.3 | -1.06 (-1.56 to -0.55) |
| Bosnia and Herzegovina |  | 795.6 | 756.5 | -1.17 (-1.60 to -0.74) |
| Botswana |  | 1477.1 | 1899 | -1.32 (-2.06 to -0.58) |
| Brunei Darussalam |  | 1003.9 | 670.2 | -1.01 (-1.23 to -0.78) |
| Bulgaria |  | 1224.2 | 1217.9 | -0.99 (-1.27 to -0.70) |
| Burkina Faso |  | 1892.4 | 1593.9 | -1.23 (-1.41 to -1.05) |
| Burundi |  | 2263.0 | 1521 | -2.70 (-3.15 to -2.25) |
| Cabo Verde |  | 847.4 | 838.5 | -0.59 (-0.87 to -0.30) |
| Cambodia |  | 1650.4 | 1172.5 | -1.63 (-1.77 to -1.48) |
| Cameroon |  | 1503.1 | 1641.3 | -0.52 (-0.84 to -0.19) |
| Central African Republic |  | 2467.1 | 2551.5 | -0.60 (-0.86 to -0.34) |
| Chad |  | 1741.3 | 1677.4 | -0.61 (-0.83 to -0.39) |
| Chile |  | 784.5 | 529.2 | -1.51 (-1.66 to -1.36) |
| China |  | 1198.2 | 644.7 | -2.14 (-2.26 to -2.01) |
| Comoros |  | 1568.8 | 1224.3 | -1.44 (-1.63 to -1.25) |
| Congo |  | 2023.8 | 1688.1 | -1.59 (-1.90 to -1.28) |
| Cook Islands |  | 1104.0 | 673.4 | -1.60 (-1.73 to -1.47) |
| Costa Rica |  | 595.1 | 563.5 | -0.92 (-1.17 to -0.67) |
| Côte d'Ivoire |  | 1659.8 | 1419.2 | -1.31 (-1.65 to -0.98) |
| Croatia |  | 1003.0 | 670.6 | -1.87 (-2.05 to -1.69) |
| Cuba |  | 725.9 | 827.7 | -0.74 (-1.11 to -0.37) |
| Cyprus |  | 1194.5 | 530.4 | -2.89 (-3.11 to -2.68) |
| Czechia |  | 984.0 | 628.1 | -1.91 (-2.10 to -1.71) |
| Democratic Republic of the Congo |  | 1796.7 | 1641 | -1.05 (-1.27 to -0.83) |
| Djibouti |  | 1383.2 | 1448.6 | -0.55 (-0.77 to -0.33) |
| Dominica |  | 922.4 | 1023 | -0.18 (-0.39 to 0.03) |
| Dominican Republic |  | 792.0 | 731.3 | -0.16 (-0.31 to -0.01) |
| Ecuador |  | 804.3 | 793.8 | -0.53 (-0.84 to -0.23) |
| Egypt |  | 1618.8 | 1421.8 | -0.57 (-0.75 to -0.39) |
| El Salvador |  | 829.0 | 792.9 | -0.61 (-0.87 to -0.35) |
| Equatorial Guinea |  | 2125.0 | 1615.5 | -1.77 (-2.19 to -1.35) |
| Eritrea |  | 2489.8 | 1684.3 | -1.59 (-1.77 to -1.40) |
| Estonia |  | 1016.8 | 646.1 | -2.49 (-2.74 to -2.24) |
| Ethiopia |  | 2499.9 | 1375 | -3.18 (-3.55 to -2.82) |
| Fiji |  | 1276.2 | 1454.6 | -0.35 (-0.53 to -0.16) |
| Gabon |  | 1561.9 | 1532.2 | -0.75 (-1.00 to -0.51) |
| Gambia |  | 1465.7 | 1526.4 | -0.34 (-0.52 to -0.16) |
| Georgia |  | 972.7 | 978.5 | -0.73 (-1.00 to -0.45) |
| Ghana |  | 1474.9 | 1394.1 | -0.64 (-0.83 to -0.45) |
| Greece |  | 670.1 | 508.9 | -1.37 (-1.51 to -1.23) |
| Grenada |  | 943.4 | 1102.7 | -0.38 (-0.65 to -0.11) |
| Guinea |  | 1776.7 | 1650.3 | -0.69 (-0.86 to -0.52) |
| Guinea-Bissau |  | 2244.4 | 2005.1 | -0.90 (-1.12 to -0.69) |
| Guyana |  | 1358.8 | 1446.5 | -0.46 (-0.70 to -0.23) |
| Hungary |  | 1088.6 | 778.5 | -1.74 (-1.92 to -1.56) |
| Indonesia |  | 1221.2 | 1118.8 | -0.60 (-0.73 to -0.47) |
| Iran (Islamic Republic of) |  | 1000.3 | 809.9 | -1.48 (-1.77 to -1.18) |
| Iraq |  | 1020.5 | 1092.1 | -0.52 (-0.84 to -0.19) |
| Italy |  | 643.8 | 401.1 | -1.75 (-1.91 to -1.60) |
| Jamaica |  | 667.9 | 754.3 | -0.14 (-0.43 to 0.15) |
| Kazakhstan |  | 1047.7 | 1165.5 | -1.01 (-1.42 to -0.60) |
| Kenya |  | 1277.2 | 1496.9 | -0.67 (-1.13 to -0.21) |
| Kiribati |  | 1694.5 | 1419 | -0.54 (-0.59 to -0.50) |
| Kuwait |  | 673.5 | 463.2 | -1.27 (-1.67 to -0.86) |
| Kyrgyzstan |  | 1077.1 | 866.7 | -1.20 (-1.44 to -0.95) |
| Lao People's Democratic Republic |  | 1954.8 | 1146.3 | -2.20 (-2.33 to -2.07) |
| Latvia |  | 1019.2 | 840.5 | -1.65 (-1.92 to -1.39) |
| Lebanon |  | 995.1 | 776.2 | -1.55 (-1.91 to -1.18) |
| Lesotho |  | 1387.9 | 3336.2 | 2.08 (1.39 to 2.77) |
| Liberia |  | 1940.9 | 1435.4 | -1.53 (-1.76 to -1.30) |
| Libya |  | 756.9 | 964.7 | 0.83 (0.62 to 1.05) |
| Lithuania |  | 913.5 | 794.3 | -1.16 (-1.40 to -0.92) |
| Luxembourg |  | 746.8 | 398.3 | -2.10 (-2.20 to -2.00) |
| Madagascar |  | 1714.9 | 1606.9 | -0.81 (-1.01 to -0.61) |
| Malaysia |  | 842.1 | 878.6 | -0.40 (-0.58 to -0.22) |
| Maldives |  | 1177.3 | 520.6 | -3.21 (-3.42 to -2.99) |
| Mali |  | 1930.5 | 1720.7 | -1.15 (-1.38 to -0.91) |
| Malta |  | 690.4 | 392.9 | -1.97 (-2.05 to -1.89) |
| Mauritania |  | 1483.7 | 1073 | -1.52 (-1.75 to -1.28) |
| Micronesia (Federated States of) |  | 1512.3 | 1245.4 | -0.64 (-0.68 to -0.61) |
| Mongolia |  | 1355.9 | 1013.7 | -1.57 (-1.77 to -1.38) |
| Montenegro |  | 723.5 | 1147.3 | 0.71 (0.39 to 1.03) |
| Morocco |  | 1035.2 | 950.6 | -0.55 (-0.74 to -0.36) |
| Mozambique |  | 1895.8 | 2116.1 | -0.06 (-0.27 to 0.15) |
| Myanmar |  | 1798.9 | 1156.4 | -2.02 (-2.25 to -1.80) |
| Namibia |  | 1430.9 | 1961.5 | -0.47 (-1.10 to 0.16) |
| Nepal |  | 1539.4 | 1192.9 | -1.46 (-1.81 to -1.10) |
| New Zealand |  | 700.8 | 399 | -1.87 (-1.96 to -1.79) |
| Nicaragua |  | 660.1 | 811.8 | -0.37 (-0.73 to -0.02) |
| Niger |  | 2014.8 | 1454.5 | -1.66 (-1.87 to -1.46) |
| Nigeria |  | 1631.5 | 1319.4 | -1.31 (-1.51 to -1.12) |
| Niue |  | 1104.6 | 1117.9 | -0.32 (-0.44 to -0.20) |
| North Macedonia |  | 991.5 | 1243.8 | -0.26 (-0.59 to 0.07) |
| Oman |  | 1021.4 | 939.1 | -0.66 (-0.96 to -0.37) |
| Pakistan |  | 1248.4 | 1318.5 | -0.55 (-0.76 to -0.33) |
| Panama |  | 596.1 | 535.9 | -0.62 (-0.78 to -0.46) |
| Papua New Guinea |  | 1450.5 | 1497 | -0.53 (-0.70 to -0.35) |
| Peru |  | 837.0 | 945.1 | -1.36 (-2.04 to -0.67) |
| Philippines |  | 1014.8 | 1158.2 | -0.10 (-0.31 to 0.11) |
| Poland |  | 955.1 | 713.7 | -1.65 (-1.87 to -1.43) |
| Portugal |  | 827.4 | 441.4 | -2.43 (-2.58 to -2.28) |
| Qatar |  | 1244.7 | 675.2 | -2.68 (-3.31 to -2.05) |
| Republic of Korea |  | 943.8 | 374.3 | -3.29 (-3.41 to -3.17) |
| Republic of Moldova |  | 1129.2 | 871.4 | -1.73 (-1.97 to -1.49) |
| Romania |  | 1069.4 | 886.7 | -1.54 (-1.78 to -1.29) |
| Russian Federation |  | 1031.1 | 1048.6 | -1.25 (-1.70 to -0.79) |
| Rwanda |  | 2249.4 | 1398 | -3.92 (-4.96 to -2.87) |
| Samoa |  | 1127.2 | 987.9 | -0.38 (-0.47 to -0.29) |
| Sao Tome and Principe |  | 1227.8 | 1054.7 | -0.86 (-0.97 to -0.74) |
| Saudi Arabia |  | 1100.1 | 858.3 | -0.98 (-1.07 to -0.90) |
| Senegal |  | 1499.6 | 1335.4 | -1.19 (-1.42 to -0.96) |
| Serbia |  | 1195.5 | 893.4 | -1.88 (-2.16 to -1.60) |
| Seychelles |  | 948.2 | 831.7 | -0.61 (-0.71 to -0.50) |
| Sierra Leone |  | 1831.0 | 1486.3 | -1.02 (-1.22 to -0.82) |
| Singapore |  | 694.7 | 292.4 | -2.96 (-3.02 to -2.89) |
| Slovakia |  | 968.1 | 785.8 | -1.29 (-1.48 to -1.09) |
| Slovenia |  | 759.8 | 471.1 | -2.08 (-2.25 to -1.91) |
| Solomon Islands |  | 1639.2 | 1321.3 | -0.70 (-0.72 to -0.67) |
| Somalia |  | 2332.5 | 2552.2 | -0.43 (-0.90 to 0.04) |
| South Africa |  | 1072.1 | 1633.6 | 0.51 (-0.27 to 1.28) |
| South Sudan |  | 1882.2 | 1977.6 | -0.41 (-0.66 to -0.15) |
| Sri Lanka |  | 1007.6 | 661.1 | -1.50 (-1.73 to -1.27) |
| Sudan |  | 1480.5 | 1163.9 | -1.47 (-1.71 to -1.22) |
| Suriname |  | 940.8 | 906.5 | -0.68 (-0.90 to -0.47) |
| Syrian Arab Republic |  | 1032.8 | 1005.1 | 0.15 (-0.31 to 0.61) |
| Tajikistan |  | 1088.4 | 1036.2 | -1.36 (-1.70 to -1.01) |
| Thailand |  | 858.0 | 621 | -1.75 (-1.95 to -1.55) |
| Timor-Leste |  | 1401.3 | 1118.9 | -1.17 (-1.44 to -0.90) |
| Togo |  | 1527.8 | 1455.7 | -0.63 (-0.86 to -0.39) |
| Tonga |  | 928.0 | 829.8 | -0.28 (-0.40 to -0.16) |
| Trinidad and Tobago |  | 1062.2 | 927.2 | -1.41 (-1.70 to -1.11) |
| Tunisia |  | 818.6 | 867.4 | -0.90 (-1.16 to -0.65) |
| Turkey |  | 1042.8 | 772.9 | -1.39 (-1.83 to -0.95) |
| Uganda |  | 2135.6 | 1603.9 | -2.42 (-2.78 to -2.05) |
| Ukraine |  | 994.7 | 1004.8 | -1.05 (-1.43 to -0.67) |
| United Arab Emirates |  | 1034.5 | 820.2 | 0.59 (0.08 to 1.11) |
| United Republic of Tanzania |  | 1671.5 | 1447 | -1.72 (-2.06 to -1.37) |
| Uruguay |  | 802.7 | 691.3 | -0.96 (-1.09 to -0.83) |
| Uzbekistan |  | 909.8 | 851.9 | -0.53 (-0.73 to -0.33) |
| Vanuatu |  | 1461.2 | 1380.3 | -0.56 (-0.66 to -0.46) |
| Venezuela (Bolivarian Republic of) |  | 786.2 | 982.4 | -0.18 (-0.59 to 0.24) |
| Viet Nam |  | 978.8 | 801.1 | -0.68 (-0.74 to -0.62) |
| Yemen |  | 1490.3 | 1347.3 | -1.09 (-1.40 to -0.77) |
| Zambia |  | 1994.8 | 2141.6 | -1.80 (-2.36 to -1.23) |
| Zimbabwe |  | 1380.4 | 2592.1 | 0.28 (-0.54 to 1.11) |

| Supplementary Table 6 : The age-standardized DALY rate (/100,000 persons) in 149 BRI countries between 1990 and 2021 | | | | |
| --- | --- | --- | --- | --- |
| Country |  | 1990 | 2021 | EAPC during 1990-2021 (95% CI) (%) |
| Afghanistan |  | 91400.6 | 73497.1 | -1.39 (-1.62 to -1.17) |
| Albania |  | 33912.5 | 26709.8 | -1.21 (-1.41 to -1.01) |
| Algeria |  | 41532.5 | 32233.0 | -1.26 (-1.43 to -1.08) |
| Angola |  | 101933.2 | 62523.2 | -2.36 (-2.57 to -2.14) |
| Antigua and Barbuda |  | 30325.5 | 28434.1 | -0.53 (-0.64 to -0.41) |
| Argentina |  | 33868.3 | 27299.2 | -0.84 (-0.90 to -0.78) |
| Armenia |  | 37341.0 | 27883.2 | -1.25 (-1.56 to -0.93) |
| Austria |  | 27328.4 | 19792.2 | -1.17 (-1.25 to -1.09) |
| Azerbaijan |  | 46678.9 | 37180.8 | -1.45 (-1.64 to -1.25) |
| Bahrain |  | 41328.1 | 31694.8 | -1.59 (-1.79 to -1.38) |
| Bangladesh |  | 68997.9 | 34955.0 | -2.45 (-2.59 to -2.31) |
| Barbados |  | 30817.6 | 27401.1 | -0.54 (-0.61 to -0.48) |
| Belarus |  | 35389.7 | 35135.3 | -1.02 (-1.34 to -0.69) |
| Benin |  | 79233.8 | 56512.6 | -1.36 (-1.45 to -1.28) |
| Bolivia (Plurinational State of) |  | 61144.7 | 47797.5 | -1.67 (-2.01 to -1.32) |
| Bosnia and Herzegovina |  | 31223.9 | 27306.4 | -1.40 (-1.86 to -0.94) |
| Botswana |  | 59973.4 | 66909.9 | -1.43 (-2.23 to -0.62) |
| Brunei Darussalam |  | 34151.2 | 25369.4 | -0.87 (-1.02 to -0.72) |
| Bulgaria |  | 38833.0 | 37902.7 | -0.90 (-1.11 to -0.69) |
| Burkina Faso |  | 95692.5 | 64495.1 | -1.72 (-1.86 to -1.58) |
| Burundi |  | 101888.0 | 58181.1 | -3.08 (-3.55 to -2.60) |
| Cabo Verde |  | 41500.5 | 31702.5 | -1.31 (-1.48 to -1.14) |
| Cambodia |  | 72529.8 | 41850.6 | -2.26 (-2.40 to -2.11) |
| Cameroon |  | 70768.0 | 62046.0 | -0.91 (-1.22 to -0.60) |
| Central African Republic |  | 111960.1 | 97933.1 | -0.93 (-1.18 to -0.68) |
| Chad |  | 87787.5 | 70276.7 | -1.03 (-1.21 to -0.85) |
| Chile |  | 32096.2 | 24000.2 | -1.06 (-1.16 to -0.95) |
| China |  | 43085.4 | 22717.2 | -2.27 (-2.35 to -2.19) |
| Comoros |  | 70277.0 | 46742.7 | -1.71 (-1.82 to -1.60) |
| Congo |  | 82812.3 | 60064.7 | -1.92 (-2.25 to -1.59) |
| Cook Islands |  | 40033.9 | 26698.3 | -1.26 (-1.41 to -1.10) |
| Costa Rica |  | 26580.5 | 26196.8 | -0.49 (-0.64 to -0.33) |
| Côte d'Ivoire |  | 79714.3 | 56654.0 | -1.69 (-2.03 to -1.36) |
| Croatia |  | 33300.1 | 24312.2 | -1.51 (-1.67 to -1.35) |
| Cuba |  | 29168.1 | 29355.1 | -0.68 (-0.92 to -0.43) |
| Cyprus |  | 31966.5 | 19917.5 | -1.73 (-1.82 to -1.63) |
| Czechia |  | 34221.3 | 24095.0 | -1.41 (-1.55 to -1.26) |
| Democratic Republic of the Congo |  | 86260.1 | 61019.6 | -1.64 (-1.86 to -1.41) |
| Djibouti |  | 60898.9 | 51618.9 | -1.00 (-1.18 to -0.81) |
| Dominica |  | 33518.6 | 38176.2 | 0.20 (0.02 to 0.38) |
| Dominican Republic |  | 39322.0 | 32631.6 | -0.53 (-0.60 to -0.45) |
| Ecuador |  | 36472.5 | 30937.1 | -0.85 (-1.04 to -0.67) |
| Egypt |  | 58005.0 | 42611.7 | -1.15 (-1.31 to -0.99) |
| El Salvador |  | 43416.9 | 33566.4 | -0.95 (-1.16 to -0.75) |
| Equatorial Guinea |  | 95454.9 | 60681.0 | -2.14 (-2.45 to -1.84) |
| Eritrea |  | 113949.6 | 60040.7 | -2.12 (-2.32 to -1.91) |
| Estonia |  | 38032.3 | 25489.0 | -2.14 (-2.35 to -1.92) |
| Ethiopia |  | 113351.6 | 51937.1 | -3.34 (-3.60 to -3.08) |
| Fiji |  | 45705.5 | 47287.9 | -0.38 (-0.50 to -0.26) |
| Gabon |  | 64432.2 | 54377.2 | -1.00 (-1.21 to -0.79) |
| Gambia |  | 67863.0 | 55745.1 | -0.95 (-1.06 to -0.84) |
| Georgia |  | 38967.6 | 33970.8 | -1.07 (-1.24 to -0.89) |
| Ghana |  | 67534.5 | 51315.3 | -1.12 (-1.29 to -0.94) |
| Greece |  | 25509.9 | 22056.3 | -0.73 (-0.80 to -0.65) |
| Grenada |  | 37930.3 | 37995.6 | -0.49 (-0.64 to -0.33) |
| Guinea |  | 92067.5 | 64783.2 | -1.32 (-1.43 to -1.22) |
| Guinea-Bissau |  | 103335.4 | 71653.1 | -1.60 (-1.76 to -1.43) |
| Guyana |  | 55292.0 | 52417.6 | -0.54 (-0.67 to -0.41) |
| Hungary |  | 39184.0 | 28445.7 | -1.58 (-1.73 to -1.44) |
| Indonesia |  | 52858.1 | 38208.1 | -1.27 (-1.39 to -1.16) |
| Iran (Islamic Republic of) |  | 47155.9 | 30373.2 | -1.63 (-1.80 to -1.47) |
| Iraq |  | 47098.2 | 38955.0 | -0.88 (-1.10 to -0.67) |
| Italy |  | 26023.3 | 18913.3 | -1.23 (-1.33 to -1.12) |
| Jamaica |  | 29498.0 | 30363.1 | -0.26 (-0.44 to -0.09) |
| Kazakhstan |  | 42682.8 | 38408.1 | -1.39 (-1.78 to -1.01) |
| Kenya |  | 59153.9 | 53177.5 | -1.38 (-1.87 to -0.88) |
| Kiribati |  | 67714.0 | 52225.3 | -0.81 (-0.85 to -0.76) |
| Kuwait |  | 30984.9 | 21885.0 | -0.94 (-1.21 to -0.67) |
| Kyrgyzstan |  | 46134.2 | 32961.8 | -1.54 (-1.74 to -1.34) |
| Lao People's Democratic Republic |  | 87951.8 | 42470.3 | -2.69 (-2.79 to -2.59) |
| Latvia |  | 38695.2 | 30935.7 | -1.63 (-1.86 to -1.40) |
| Lebanon |  | 41332.8 | 29648.7 | -1.37 (-1.64 to -1.11) |
| Lesotho |  | 60562.7 | 117884.9 | 1.62 (0.92 to 2.33) |
| Liberia |  | 107830.1 | 58621.7 | -2.36 (-2.60 to -2.11) |
| Libya |  | 34953.9 | 37448.4 | 0.39 (0.16 to 0.61) |
| Lithuania |  | 35517.2 | 29981.2 | -1.15 (-1.36 to -0.94) |
| Luxembourg |  | 28382.1 | 18908.2 | -1.45 (-1.54 to -1.35) |
| Madagascar |  | 77772.0 | 58531.0 | -1.25 (-1.35 to -1.14) |
| Malaysia |  | 33400.1 | 30958.4 | -0.57 (-0.70 to -0.44) |
| Maldives |  | 48868.2 | 22079.8 | -2.86 (-3.07 to -2.64) |
| Mali |  | 99430.7 | 68253.6 | -1.66 (-1.79 to -1.52) |
| Malta |  | 25550.5 | 19260.8 | -0.99 (-1.04 to -0.94) |
| Mauritania |  | 63648.2 | 40013.8 | -1.77 (-1.89 to -1.66) |
| Micronesia (Federated States of) |  | 55132.6 | 44027.9 | -0.75 (-0.79 to -0.70) |
| Mongolia |  | 56692.2 | 37496.9 | -1.75 (-1.87 to -1.63) |
| Montenegro |  | 28433.7 | 32582.7 | -0.17 (-0.35 to 0.02) |
| Morocco |  | 47415.6 | 34266.4 | -1.25 (-1.37 to -1.13) |
| Mozambique |  | 96752.8 | 78703.9 | -0.84 (-1.02 to -0.66) |
| Myanmar |  | 79486.9 | 43311.5 | -2.39 (-2.62 to -2.16) |
| Namibia |  | 57974.3 | 65915.7 | -0.66 (-1.29 to -0.01) |
| Nepal |  | 72466.6 | 42967.3 | -2.08 (-2.31 to -1.85) |
| New Zealand |  | 29378.9 | 20402.3 | -1.18 (-1.26 to -1.10) |
| Nicaragua |  | 37031.6 | 32141.5 | -1.03 (-1.26 to -0.81) |
| Niger |  | 108666.5 | 59453.9 | -2.43 (-2.61 to -2.26) |
| Nigeria |  | 84740.8 | 57660.9 | -1.59 (-1.76 to -1.43) |
| Niue |  | 39765.1 | 45301.1 | -0.20 (-0.40 to 0.01) |
| North Macedonia |  | 37387.5 | 35282.0 | -0.89 (-1.07 to -0.71) |
| Oman |  | 40615.8 | 32139.3 | -1.01 (-1.18 to -0.85) |
| Pakistan |  | 59253.8 | 51687.1 | -0.81 (-0.93 to -0.69) |
| Panama |  | 28858.9 | 25769.5 | -0.49 (-0.58 to -0.40) |
| Papua New Guinea |  | 60268.5 | 54479.7 | -0.69 (-0.80 to -0.58) |
| Peru |  | 43203.7 | 33992.0 | -1.72 (-2.15 to -1.28) |
| Philippines |  | 43485.8 | 41088.9 | -0.48 (-0.62 to -0.34) |
| Poland |  | 35793.5 | 26825.1 | -1.37 (-1.53 to -1.21) |
| Portugal |  | 31261.6 | 20692.0 | -1.61 (-1.71 to -1.50) |
| Qatar |  | 38186.2 | 24637.7 | -1.82 (-2.14 to -1.49) |
| Republic of Korea |  | 33347.1 | 17522.0 | -2.21 (-2.32 to -2.11) |
| Republic of Moldova |  | 42159.4 | 32865.1 | -1.37 (-1.54 to -1.21) |
| Romania |  | 39325.0 | 31042.9 | -1.43 (-1.62 to -1.24) |
| Russian Federation |  | 39713.3 | 37550.7 | -1.20 (-1.59 to -0.81) |
| Rwanda |  | 98814.6 | 50628.8 | -4.30 (-5.48 to -3.11) |
| Samoa |  | 41235.5 | 35900.2 | -0.35 (-0.46 to -0.23) |
| Sao Tome and Principe |  | 56176.1 | 36735.2 | -1.77 (-1.90 to -1.65) |
| Saudi Arabia |  | 44313.1 | 31951.1 | -1.08 (-1.15 to -1.01) |
| Senegal |  | 70520.3 | 48560.9 | -1.77 (-1.92 to -1.61) |
| Serbia |  | 38258.5 | 28723.9 | -1.58 (-1.78 to -1.37) |
| Seychelles |  | 35654.4 | 30671.6 | -0.56 (-0.63 to -0.49) |
| Sierra Leone |  | 95756.2 | 63192.8 | -1.65 (-1.85 to -1.45) |
| Singapore |  | 25972.2 | 14980.9 | -1.85 (-1.91 to -1.79) |
| Slovakia |  | 35263.8 | 28066.8 | -1.16 (-1.30 to -1.03) |
| Slovenia |  | 29862.4 | 20262.8 | -1.60 (-1.70 to -1.50) |
| Solomon Islands |  | 59384.1 | 46721.6 | -0.77 (-0.81 to -0.74) |
| Somalia |  | 105297.2 | 90159.2 | -0.91 (-1.38 to -0.44) |
| South Africa |  | 54882.7 | 62840.0 | -0.05 (-0.79 to 0.71) |
| South Sudan |  | 92468.3 | 82213.8 | -0.65 (-0.85 to -0.44) |
| Sri Lanka |  | 39138.4 | 26148.4 | -1.53 (-1.77 to -1.28) |
| Sudan |  | 72331.9 | 43799.4 | -1.99 (-2.13 to -1.85) |
| Suriname |  | 42864.4 | 38368.8 | -0.72 (-0.86 to -0.58) |
| Syrian Arab Republic |  | 41518.0 | 34692.7 | 0.51 (-0.26 to 1.30) |
| Tajikistan |  | 48040.4 | 39103.5 | -1.69 (-1.99 to -1.40) |
| Thailand |  | 36845.0 | 27341.3 | -1.48 (-1.66 to -1.30) |
| Timor-Leste |  | 67836.5 | 41367.5 | -2.05 (-2.33 to -1.76) |
| Togo |  | 72215.8 | 55671.4 | -1.11 (-1.34 to -0.87) |
| Tonga |  | 35720.4 | 31205.4 | -0.39 (-0.50 to -0.28) |
| Trinidad and Tobago |  | 39749.5 | 36653.7 | -0.87 (-1.05 to -0.70) |
| Tunisia |  | 36316.9 | 31707.4 | -1.08 (-1.26 to -0.90) |
| Turkey |  | 45335.8 | 28509.3 | -1.84 (-2.10 to -1.57) |
| Uganda |  | 104171.3 | 61279.8 | -2.81 (-3.10 to -2.52) |
| Ukraine |  | 37445.4 | 36128.0 | -0.91 (-1.19 to -0.63) |
| United Arab Emirates |  | 37626.7 | 27669.2 | -0.42 (-0.65 to -0.18) |
| United Republic of Tanzania |  | 81657.0 | 54679.3 | -2.21 (-2.51 to -1.91) |
| Uruguay |  | 32163.3 | 27866.3 | -0.79 (-0.88 to -0.70) |
| Uzbekistan |  | 40581.5 | 32884.8 | -0.96 (-1.10 to -0.82) |
| Vanuatu |  | 52672.9 | 47256.9 | -0.62 (-0.70 to -0.54) |
| Venezuela (Bolivarian Republic of) |  | 35068.9 | 38861.3 | -0.13 (-0.41 to 0.15) |
| Viet Nam |  | 39579.3 | 28579.6 | -1.05 (-1.13 to -0.97) |
| Yemen |  | 70508.6 | 51473.7 | -1.46 (-1.72 to -1.20) |
| Zambia |  | 93190.5 | 72861.9 | -2.38 (-2.91 to -1.85) |
| Zimbabwe |  | 59725.9 | 86148.0 | -0.39 (-1.23 to 0.47) |

| Supplementary Table 7: Top five causes of age-standardized mortality (/100,000 persons) across 149 BRI countries in 1990 and in 2021 | | | | | | |
| --- | --- | --- | --- | --- | --- | --- |
| Country | Rank | 1990 | |  | 2021 | |
|  |  | Cause | Mortality (95% UI) |  | Cause | Mortality (95% UI) |
| Afghanistan | 1 | Ischemic heart disease | 361.4 (280.0, 444.6) |  | COVID-19 | 420.1 (328.6, 494.1) |
|  | 2 | Stroke | 255.8 (190.7, 321.1) |  | Ischemic heart disease | 280.0 (221.4, 346.2) |
|  | 3 | Tuberculosis | 141.9 (96.8, 264.8) |  | Stroke | 183.9 (137.6, 228.4) |
|  | 4 | Lower respiratory infections | 131.0 (108.9, 155.0) |  | Conflict and terrorism | 111.1 (104.0, 118.6) |
|  | 5 | Hypertensive heart disease | 100.0 (49.0, 155.6) |  | Hypertensive heart disease | 78.3 (43.8, 115.0) |
| Albania | 1 | Stroke | 201.1 (178.0, 222.2) |  | Ischemic heart disease | 158.3 (134.2, 185.5) |
|  | 2 | Ischemic heart disease | 174.2 (153.5, 192.2) |  | Stroke | 150.7 (122.8, 181.8) |
|  | 3 | Lower respiratory infections | 60.7 (52.0, 67.5) |  | COVID-19 | 114.6 (51.5, 172.3) |
|  | 4 | Chronic obstructive pulmonary disease | 40.5 (34.7, 47.3) |  | Tracheal, bronchus, and lung cancer | 24.2 (16.9, 33.0) |
|  | 5 | Tracheal, bronchus, and lung cancer | 29.1 (23.5, 36.1) |  | Alzheimer's disease and other dementias | 20.8 (4.9, 56.6) |
| Algeria | 1 | Ischemic heart disease | 310.6 (267.5, 359.9) |  | Ischemic heart disease | 212.0 (172.7, 251.7) |
|  | 2 | Stroke | 170.9 (139.1, 206.8) |  | COVID-19 | 183.8 (143.1, 220.8) |
|  | 3 | Hypertensive heart disease | 66.0 (44.2, 87.7) |  | Stroke | 109.2 (86.8, 134.4) |
|  | 4 | Road injuries | 49.5 (42.8, 55.9) |  | Hypertensive heart disease | 50.7 (33.3, 67.5) |
|  | 5 | Neonatal disorders | 47.3 (40.5, 56.5) |  | Chronic kidney disease | 37.3 (30.6, 45.5) |
| Angola | 1 | Diarrheal diseases | 274.7 (186.7, 383.8) |  | COVID-19 | 294.2 (215.9, 371.1) |
|  | 2 | Tuberculosis | 205.1 (129.4, 286.1) |  | Stroke | 137.6 (109.0, 168.7) |
|  | 3 | Stroke | 183.8 (149.3, 221.8) |  | Ischemic heart disease | 125.5 (99.6, 156.4) |
|  | 4 | Lower respiratory infections | 170.0 (141.9, 201.2) |  | Lower respiratory infections | 88.5 (72.0, 109.0) |
|  | 5 | Ischemic heart disease | 126.9 (102.5, 155.5) |  | Tuberculosis | 77.0 (53.2, 103.3) |
| Antigua and Barbuda | 1 | Ischemic heart disease | 127.2 (118.7, 134.9) |  | COVID-19 | 119.4 (119.4, 119.4) |
|  | 2 | Stroke | 122.5 (113.7, 130.3) |  | Stroke | 72.0 (67.0, 77.1) |
|  | 3 | Diabetes mellitus | 68.0 (62.9, 73.0) |  | Ischemic heart disease | 71.4 (66.6, 77.9) |
|  | 4 | Hypertensive heart disease | 36.9 (33.8, 39.7) |  | Diabetes mellitus | 59.0 (54.2, 62.5) |
|  | 5 | Lower respiratory infections | 36.7 (34.0, 39.4) |  | Chronic kidney disease | 47.1 (43.0, 50.5) |
| Argentina | 1 | Ischemic heart disease | 158.8 (149.3, 164.6) |  | COVID-19 | 129.3 (129.2, 129.3) |
|  | 2 | Stroke | 111.7 (105.2, 116.5) |  | Ischemic heart disease | 60.8 (55.9, 64.1) |
|  | 3 | Tracheal, bronchus, and lung cancer | 30.5 (28.5, 32.5) |  | Lower respiratory infections | 52.3 (46.5, 56.3) |
|  | 4 | Chronic kidney disease | 30.4 (28.6, 31.9) |  | Stroke | 39.8 (36.4, 42.5) |
|  | 5 | Lower respiratory infections | 28.2 (26.3, 29.5) |  | Chronic kidney disease | 26.3 (24.0, 28.1) |
| Armenia | 1 | Ischemic heart disease | 307.4 (286.8, 323.8) |  | Ischemic heart disease | 209.8 (186.4, 233.4) |
|  | 2 | Stroke | 118.7 (110.6, 124.1) |  | COVID-19 | 120.8 (120.8, 120.9) |
|  | 3 | Chronic obstructive pulmonary disease | 43.3 (40.5, 45.8) |  | Stroke | 65.3 (57.3, 72.9) |
|  | 4 | Tracheal, bronchus, and lung cancer | 35.9 (33.6, 38.0) |  | Tracheal, bronchus, and lung cancer | 27.4 (24.4, 30.8) |
|  | 5 | Lower respiratory infections | 32.9 (30.1, 36.4) |  | Lower respiratory infections | 22.3 (19.4, 25.7) |
| Austria | 1 | Ischemic heart disease | 163.5 (150.0, 169.7) |  | Ischemic heart disease | 68.1 (59.0, 73.2) |
|  | 2 | Stroke | 88.0 (80.3, 93.2) |  | COVID-19 | 48.8 (46.4, 51.1) |
|  | 3 | Cardiomyopathy and myocarditis | 40.0 (35.9, 42.9) |  | Alzheimer's disease and other dementias | 24.4 (6.3, 61.9) |
|  | 4 | Tracheal, bronchus, and lung cancer | 29.4 (27.8, 30.7) |  | Tracheal, bronchus, and lung cancer | 22.9 (20.9, 24.9) |
|  | 5 | Alzheimer's disease and other dementias | 25.4 (6.2, 67.7) |  | Stroke | 21.4 (18.3, 23.2) |
| Azerbaijan | 1 | Ischemic heart disease | 362.3 (337.1, 388.2) |  | Ischemic heart disease | 306.1 (270.8, 343.5) |
|  | 2 | Stroke | 127.2 (111.2, 142.6) |  | COVID-19 | 217.7 (172.9, 252.9) |
|  | 3 | Lower respiratory infections | 98.5 (85.7, 111.4) |  | Stroke | 101.3 (85.3, 120.1) |
|  | 4 | Cirrhosis and other chronic liver diseases | 33.7 (29.3, 38.0) |  | Cirrhosis and other chronic liver diseases | 27.3 (20.6, 35.5) |
|  | 5 | Neonatal disorders | 29.8 (25.1, 34.9) |  | Lower respiratory infections | 24.6 (20.9, 29.1) |
| Bahrain | 1 | Ischemic heart disease | 408.1 (381.8, 433.0) |  | COVID-19 | 172.7 (139.9, 200.2) |
|  | 2 | Stroke | 160.0 (143.6, 175.1) |  | Ischemic heart disease | 161.6 (140.6, 184.4) |
|  | 3 | Diabetes mellitus | 107.1 (93.7, 120.1) |  | Diabetes mellitus | 126.7 (104.1, 146.0) |
|  | 4 | Chronic obstructive pulmonary disease | 63.9 (56.9, 71.2) |  | Stroke | 81.5 (70.7, 94.5) |
|  | 5 | Chronic kidney disease | 41.7 (31.3, 55.7) |  | Chronic kidney disease | 52.5 (42.1, 63.4) |
| Bangladesh | 1 | Stroke | 203.4 (177.0, 231.7) |  | Stroke | 149.0 (122.6, 179.4) |
|  | 2 | Tuberculosis | 127.7 (96.2, 155.7) |  | Ischemic heart disease | 107.5 (86.2, 131.7) |
|  | 3 | Diarrheal diseases | 122.6 (78.5, 165.3) |  | COVID-19 | 104.5 (89.5, 126.9) |
|  | 4 | Ischemic heart disease | 119.1 (101.7, 138.5) |  | Chronic obstructive pulmonary disease | 59.6 (46.2, 77.2) |
|  | 5 | Chronic obstructive pulmonary disease | 101.9 (77.9, 125.5) |  | Diabetes mellitus | 36.0 (29.2, 43.7) |
| Barbados | 1 | Ischemic heart disease | 120.8 (112.9, 126.5) |  | Stroke | 71.6 (58.8, 86.3) |
|  | 2 | Stroke | 116.3 (108.8, 122.1) |  | Ischemic heart disease | 62.5 (51.4, 74.6) |
|  | 3 | Diabetes mellitus | 77.1 (72.6, 81.8) |  | Diabetes mellitus | 61.2 (48.4, 74.2) |
|  | 4 | Lower respiratory infections | 29.3 (27.3, 30.9) |  | COVID-19 | 56.9 (56.9, 57.0) |
|  | 5 | Neonatal disorders | 20.4 (17.3, 23.9) |  | Chronic kidney disease | 29.3 (23.3, 36.0) |
| Belarus | 1 | Ischemic heart disease | 331.1 (309.3, 343.5) |  | Ischemic heart disease | 341.5 (292.0, 394.6) |
|  | 2 | Stroke | 133.4 (124.0, 141.5) |  | COVID-19 | 194.7 (151.7, 251.7) |
|  | 3 | Chronic obstructive pulmonary disease | 50.3 (47.1, 53.4) |  | Stroke | 97.8 (81.9, 114.7) |
|  | 4 | Tracheal, bronchus, and lung cancer | 31.9 (29.5, 34.4) |  | Tracheal, bronchus, and lung cancer | 21.6 (16.8, 26.4) |
|  | 5 | Stomach cancer | 31.3 (28.6, 34.2) |  | Alzheimer's disease and other dementias | 20.6 (5.0, 56.4) |
| Benin | 1 | Stroke | 193.7 (168.0, 224.2) |  | COVID-19 | 196.7 (175.5, 217.6) |
|  | 2 | Diarrheal diseases | 188.9 (119.1, 260.0) |  | Stroke | 149.8 (127.9, 175.5) |
|  | 3 | Lower respiratory infections | 164.0 (140.8, 186.8) |  | Malaria | 131.4 (56.3, 255.1) |
|  | 4 | Malaria | 89.1 (44.9, 142.2) |  | Lower respiratory infections | 85.1 (68.1, 103.9) |
|  | 5 | Tuberculosis | 81.6 (68.3, 105.5) |  | Ischemic heart disease | 77.6 (65.2, 93.1) |
| Bolivia  (Plurinational State of) | 1 | Lower respiratory infections | 162.0 (139.9, 186.9) |  | COVID-19 | 490.9 (420.8, 541.2) |
|  | 2 | Ischemic heart disease | 142.7 (113.8, 184.2) |  | Ischemic heart disease | 84.6 (62.4, 121.7) |
|  | 3 | Stroke | 125.2 (97.3, 164.3) |  | Stroke | 67.5 (49.8, 89.4) |
|  | 4 | Tuberculosis | 95.6 (69.3, 120.2) |  | Lower respiratory infections | 60.2 (48.8, 75.3) |
|  | 5 | Stomach cancer | 51.2 (42.5, 61.8) |  | Chronic kidney disease | 58.2 (45.7, 75.3) |
| Bosnia and Herzegovina | 1 | Ischemic heart disease | 200.1 (181.5, 217.0) |  | COVID-19 | 155.7 (155.2, 156.4) |
|  | 2 | Stroke | 163.9 (147.8, 180.5) |  | Ischemic heart disease | 138.3 (114.1, 162.5) |
|  | 3 | Tracheal, bronchus, and lung cancer | 35.5 (31.5, 39.6) |  | Stroke | 110.1 (87.8, 130.1) |
|  | 4 | Chronic obstructive pulmonary disease | 30.9 (27.4, 34.5) |  | Tracheal, bronchus, and lung cancer | 35.9 (27.9, 45.3) |
|  | 5 | Neonatal disorders | 22.8 (21.0, 24.5) |  | Diabetes mellitus | 31.0 (24.9, 38.3) |
| Botswana | 1 | Stroke | 187.1 (144.5, 230.1) |  | COVID-19 | 582.8 (424.0, 710.1) |
|  | 2 | Tuberculosis | 136.9 (84.6, 202.9) |  | HIV/AIDS | 229.4 (185.1, 294.4) |
|  | 3 | Lower respiratory infections | 130.1 (96.2, 169.3) |  | Stroke | 106.2 (85.6, 133.5) |
|  | 4 | Diarrheal diseases | 111.5 (66.3, 173.3) |  | Ischemic heart disease | 78.6 (62.1, 98.7) |
|  | 5 | Ischemic heart disease | 101.4 (75.7, 127.4) |  | Lower respiratory infections | 68.0 (51.9, 87.5) |
| Brunei Darussalam | 1 | Ischemic heart disease | 149.6 (132.7, 166.5) |  | Ischemic heart disease | 84.5 (74.3, 95.9) |
|  | 2 | Stroke | 145.4 (127.7, 164.5) |  | Stroke | 71.1 (60.5, 81.7) |
|  | 3 | Diabetes mellitus | 84.8 (72.9, 97.4) |  | Diabetes mellitus | 51.5 (44.8, 58.9) |
|  | 4 | Chronic obstructive pulmonary disease | 63.8 (53.8, 75.9) |  | Chronic kidney disease | 36.3 (30.6, 42.3) |
|  | 5 | Lower respiratory infections | 51.3 (44.5, 58.9) |  | COVID-19 | 36.0 (34.6, 47.2) |
| Bulgaria | 1 | Ischemic heart disease | 404.0 (387.8, 416.7) |  | COVID-19 | 258.4 (172.5, 340.0) |
|  | 2 | Stroke | 305.2 (292.2, 316.5) |  | Ischemic heart disease | 204.4 (180.5, 230.3) |
|  | 3 | Lower respiratory infections | 46.3 (43.9, 48.3) |  | Stroke | 192.1 (168.4, 215.7) |
|  | 4 | Hypertensive heart disease | 39.4 (35.4, 43.3) |  | Hypertensive heart disease | 103.4 (89.6, 116.4) |
|  | 5 | Chronic obstructive pulmonary disease | 31.4 (28.1, 35.0) |  | Tracheal, bronchus, and lung cancer | 33.7 (28.2, 39.5) |
| Burkina Faso | 1 | Diarrheal diseases | 206.2 (135.6, 293.6) |  | COVID-19 | 219.2 (178.3, 248.9) |
|  | 2 | Lower respiratory infections | 193.6 (163.3, 222.7) |  | Malaria | 173.1 (75.4, 318.5) |
|  | 3 | Malaria | 189.8 (95.3, 349.2) |  | Stroke | 107.8 (90.1, 130.0) |
|  | 4 | HIV/AIDS | 136.4 (86.3, 207.0) |  | Lower respiratory infections | 105.9 (85.7, 126.4) |
|  | 5 | Stroke | 119.6 (101.8, 141.1) |  | Ischemic heart disease | 97.6 (75.5, 124.1) |
| Burundi | 1 | Tuberculosis | 286.4 (219.5, 364.4) |  | COVID-19 | 183.9 (166.7, 199.3) |
|  | 2 | Stroke | 259.0 (195.4, 319.7) |  | Stroke | 140.1 (110.7, 175.4) |
|  | 3 | Lower respiratory infections | 178.3 (144.3, 214.3) |  | Tuberculosis | 120.7 (87.0, 160.5) |
|  | 4 | Malaria | 167.6 (80.6, 282.1) |  | Lower respiratory infections | 92.6 (73.4, 117.2) |
|  | 5 | Diarrheal diseases | 145.3 (84.3, 204.5) |  | Malaria | 90.3 (37.9, 193.0) |
| Cabo Verde | 1 | Stroke | 98.5 (81.6, 114.7) |  | Ischemic heart disease | 121.5 (100.5, 142.3) |
|  | 2 | Ischemic heart disease | 71.8 (59.6, 82.9) |  | Stroke | 105.5 (86.7, 127.3) |
|  | 3 | Diarrheal diseases | 62.5 (41.6, 82.2) |  | Lower respiratory infections | 61.6 (50.3, 73.6) |
|  | 4 | Lower respiratory infections | 59.2 (51.0, 69.7) |  | COVID-19 | 54.9 (54.4, 59.4) |
|  | 5 | Chronic obstructive pulmonary disease | 37.5 (30.0, 44.1) |  | Diabetes mellitus | 35.8 (29.0, 43.0) |
| Cambodia | 1 | Stroke | 237.6 (201.5, 277.5) |  | Stroke | 185.2 (148.0, 220.1) |
|  | 2 | Lower respiratory infections | 208.4 (181.1, 235.9) |  | Ischemic heart disease | 111.5 (89.2, 134.6) |
|  | 3 | Tuberculosis | 170.6 (113.9, 219.5) |  | Lower respiratory infections | 93.1 (72.8, 111.2) |
|  | 4 | Ischemic heart disease | 115.7 (97.9, 135.1) |  | COVID-19 | 87.3 (29.2, 172.0) |
|  | 5 | Diarrheal diseases | 93.3 (57.8, 143.1) |  | Tuberculosis | 54.1 (35.8, 77.1) |
| Cameroon | 1 | Stroke | 160.6 (133.4, 196.8) |  | COVID-19 | 296.1 (259.3, 334.9) |
|  | 2 | Lower respiratory infections | 140.9 (115.1, 171.6) |  | Stroke | 149.2 (115.5, 193.2) |
|  | 3 | Malaria | 114.0 (58.0, 193.7) |  | Malaria | 116.4 (44.6, 244.7) |
|  | 4 | Diarrheal diseases | 112.0 (70.3, 165.4) |  | Ischemic heart disease | 103.3 (79.9, 137.1) |
|  | 5 | Tuberculosis | 82.0 (60.6, 103.4) |  | Lower respiratory infections | 87.9 (65.3, 116.4) |
| Central African Republic | 1 | Tuberculosis | 350.1 (224.3, 448.5) |  | COVID-19 | 342.1 (192.5, 523.5) |
|  | 2 | Lower respiratory infections | 224.8 (187.7, 269.3) |  | Tuberculosis | 265.0 (173.7, 356.8) |
|  | 3 | Stroke | 224.7 (171.3, 284.4) |  | Stroke | 191.3 (139.3, 250.2) |
|  | 4 | Diarrheal diseases | 188.1 (126.4, 264.7) |  | Lower respiratory infections | 152.5 (116.1, 193.8) |
|  | 5 | Ischemic heart disease | 166.0 (127.2, 223.3) |  | Ischemic heart disease | 150.3 (109.4, 210.6) |
| Chad | 1 | Diarrheal diseases | 307.3 (190.4, 435.9) |  | COVID-19 | 174.5 (127.1, 207.7) |
|  | 2 | Lower respiratory infections | 174.9 (143.0, 211.8) |  | Stroke | 162.4 (128.4, 201.4) |
|  | 3 | Stroke | 155.7 (125.8, 183.2) |  | Diarrheal diseases | 152.5 (100.4, 222.6) |
|  | 4 | Tuberculosis | 127.9 (93.8, 171.3) |  | Lower respiratory infections | 132.7 (107.5, 161.3) |
|  | 5 | Ischemic heart disease | 87.9 (70.4, 109.9) |  | Ischemic heart disease | 104.3 (80.3, 131.3) |
| Chile | 1 | Ischemic heart disease | 118.5 (111.1, 122.9) |  | COVID-19 | 109.0 (104.7, 113.2) |
|  | 2 | Stroke | 96.5 (90.7, 101.3) |  | Ischemic heart disease | 39.1 (35.8, 41.3) |
|  | 3 | Lower respiratory infections | 72.4 (66.9, 76.4) |  | Stroke | 37.8 (33.9, 40.6) |
|  | 4 | Cirrhosis and other chronic liver diseases | 38.5 (37.1, 39.9) |  | Chronic kidney disease | 19.8 (17.5, 21.3) |
|  | 5 | Stomach cancer | 34.2 (31.6, 36.7) |  | Alzheimer's disease and other dementias | 19.4 (4.9, 50.0) |
| China | 1 | Stroke | 242.2 (213.8, 272.7) |  | Stroke | 138.0 (116.7, 160.3) |
|  | 2 | Chronic obstructive pulmonary disease | 231.8 (199.0, 257.4) |  | Ischemic heart disease | 110.9 (92.4, 128.6) |
|  | 3 | Ischemic heart disease | 94.1 (84.0, 105.9) |  | Chronic obstructive pulmonary disease | 73.2 (59.7, 86.9) |
|  | 4 | Lower respiratory infections | 60.7 (53.0, 66.7) |  | Tracheal, bronchus, and lung cancer | 39.0 (31.4, 47.1) |
|  | 5 | Stomach cancer | 46.0 (38.9, 54.4) |  | Alzheimer's disease and other dementias | 30.8 (7.9, 82.4) |
| Comoros | 1 | Stroke | 182.9 (148.9, 224.0) |  | COVID-19 | 160.7 (146.0, 173.1) |
|  | 2 | Tuberculosis | 178.1 (125.4, 239.4) |  | Stroke | 112.8 (87.3, 139.8) |
|  | 3 | Lower respiratory infections | 146.5 (114.9, 173.5) |  | Lower respiratory infections | 79.5 (60.5, 101.8) |
|  | 4 | Diarrheal diseases | 77.6 (46.5, 113.3) |  | Ischemic heart disease | 73.2 (55.3, 95.1) |
|  | 5 | Ischemic heart disease | 77.3 (60.0, 98.6) |  | Tuberculosis | 68.0 (48.2, 94.0) |
| Congo | 1 | Stroke | 214.0 (172.3, 253.9) |  | COVID-19 | 241.8 (169.3, 310.9) |
|  | 2 | Ischemic heart disease | 186.7 (149.0, 224.1) |  | Ischemic heart disease | 158.9 (127.7, 189.8) |
|  | 3 | Tuberculosis | 161.8 (108.4, 212.8) |  | Stroke | 146.7 (115.9, 180.2) |
|  | 4 | Lower respiratory infections | 141.6 (113.4, 168.6) |  | HIV/AIDS | 88.1 (66.4, 125.2) |
|  | 5 | Diarrheal diseases | 113.4 (70.5, 165.4) |  | Lower respiratory infections | 86.2 (64.6, 107.2) |
| Cook Islands | 1 | Ischemic heart disease | 160.3 (141.4, 183.7) |  | Diabetes mellitus | 113.0 (93.5, 134.1) |
|  | 2 | Diabetes mellitus | 148.4 (128.2, 172.1) |  | Ischemic heart disease | 107.2 (89.0, 128.1) |
|  | 3 | Stroke | 132.1 (115.5, 149.0) |  | Stroke | 63.3 (52.5, 75.0) |
|  | 4 | Hypertensive heart disease | 108.6 (88.4, 132.8) |  | Hypertensive heart disease | 56.2 (44.9, 69.7) |
|  | 5 | Lower respiratory infections | 83.9 (73.1, 95.3) |  | Lower respiratory infections | 40.1 (34.1, 47.6) |
| Costa Rica | 1 | Ischemic heart disease | 124.8 (114.4, 131.2) |  | COVID-19 | 93.3 (62.4, 128.2) |
|  | 2 | Stroke | 46.8 (42.7, 49.6) |  | Ischemic heart disease | 55.1 (48.2, 61.1) |
|  | 3 | Stomach cancer | 36.1 (33.4, 38.6) |  | Chronic kidney disease | 29.0 (25.2, 32.3) |
|  | 4 | Chronic obstructive pulmonary disease | 25.6 (23.2, 27.4) |  | Stroke | 27.3 (23.5, 30.6) |
|  | 5 | Lower respiratory infections | 21.7 (20.0, 22.9) |  | Chronic obstructive pulmonary disease | 19.7 (16.6, 22.1) |
| Croatia | 1 | Ischemic heart disease | 287.4 (271.4, 300.7) |  | Ischemic heart disease | 130.5 (114.9, 143.1) |
|  | 2 | Stroke | 195.3 (183.7, 205.7) |  | COVID-19 | 93.6 (60.4, 128.1) |
|  | 3 | Tracheal, bronchus, and lung cancer | 42.7 (38.5, 47.0) |  | Stroke | 66.0 (57.7, 74.3) |
|  | 4 | Hypertensive heart disease | 30.2 (27.2, 33.5) |  | Tracheal, bronchus, and lung cancer | 33.7 (29.5, 38.1) |
|  | 5 | Cirrhosis and other chronic liver diseases | 27.3 (26.2, 28.4) |  | Colon and rectum cancer | 24.3 (20.8, 28.0) |
| Cuba | 1 | Ischemic heart disease | 214.2 (203.0, 220.2) |  | COVID-19 | 231.8 (191.4, 274.5) |
|  | 2 | Stroke | 78.6 (74.6, 81.6) |  | Ischemic heart disease | 104.8 (91.3, 117.3) |
|  | 3 | Lower respiratory infections | 35.9 (33.5, 37.5) |  | Stroke | 54.6 (47.9, 61.2) |
|  | 4 | Tracheal, bronchus, and lung cancer | 33.6 (31.7, 35.5) |  | Lower respiratory infections | 33.2 (29.0, 37.2) |
|  | 5 | Diabetes mellitus | 22.3 (21.1, 23.3) |  | Tracheal, bronchus, and lung cancer | 31.9 (27.7, 36.6) |
| Cyprus | 1 | Ischemic heart disease | 271.5 (245.0, 299.1) |  | Ischemic heart disease | 87.0 (75.8, 98.1) |
|  | 2 | Stroke | 177.3 (152.3, 204.0) |  | Stroke | 47.8 (40.1, 55.4) |
|  | 3 | Diabetes mellitus | 79.1 (67.8, 89.9) |  | COVID-19 | 30.6 (23.4, 42.0) |
|  | 4 | Chronic obstructive pulmonary disease | 60.3 (42.4, 73.7) |  | Diabetes mellitus | 29.1 (24.8, 33.8) |
|  | 5 | Chronic kidney disease | 41.3 (34.1, 51.5) |  | Chronic obstructive pulmonary disease | 27.0 (22.7, 31.6) |
| Czechia | 1 | Ischemic heart disease | 296.0 (282.6, 305.5) |  | Ischemic heart disease | 126.7 (110.4, 139.6) |
|  | 2 | Stroke | 195.1 (182.2, 205.8) |  | COVID-19 | 115.2 (76.9, 157.0) |
|  | 3 | Tracheal, bronchus, and lung cancer | 46.9 (43.9, 50.2) |  | Stroke | 40.6 (35.6, 45.5) |
|  | 4 | Colon and rectum cancer | 34.6 (31.1, 38.4) |  | Tracheal, bronchus, and lung cancer | 27.7 (24.3, 31.2) |
|  | 5 | Falls | 26.2 (24.3, 28.0) |  | Alzheimer's disease and other dementias | 20.4 (5.0, 53.6) |
| Côte d'Ivoire | 1 | Stroke | 164.4 (138.9, 198.2) |  | COVID-19 | 177.4 (153.1, 206.7) |
|  | 2 | Malaria | 156.6 (71.0, 277.8) |  | Stroke | 141.9 (114.3, 175.8) |
|  | 3 | Lower respiratory infections | 155.0 (132.7, 181.3) |  | Malaria | 122.7 (47.8, 243.3) |
|  | 4 | HIV/AIDS | 133.1 (78.2, 238.4) |  | Ischemic heart disease | 119.9 (98.8, 152.7) |
|  | 5 | Ischemic heart disease | 118.1 (97.3, 141.6) |  | Lower respiratory infections | 90.1 (70.4, 112.5) |
| Democratic Republic of the Congo | 1 | Tuberculosis | 180.8 (119.2, 259.1) |  | COVID-19 | 257.1 (219.9, 309.7) |
|  | 2 | Lower respiratory infections | 169.1 (131.8, 222.7) |  | Stroke | 138.6 (100.9, 185.5) |
|  | 3 | Stroke | 155.5 (119.9, 200.4) |  | Ischemic heart disease | 112.3 (83.3, 149.1) |
|  | 4 | Ischemic heart disease | 130.6 (99.0, 169.5) |  | Lower respiratory infections | 112.2 (79.3, 154.8) |
|  | 5 | Malaria | 122.9 (66.6, 199.1) |  | Tuberculosis | 107.5 (71.8, 170.9) |
| Djibouti | 1 | Stroke | 161.0 (122.8, 210.1) |  | COVID-19 | 250.2 (166.7, 334.0) |
|  | 2 | Tuberculosis | 128.1 (88.3, 171.8) |  | Stroke | 120.7 (92.0, 157.2) |
|  | 3 | Diarrheal diseases | 118.9 (75.5, 166.8) |  | Ischemic heart disease | 91.5 (67.7, 118.9) |
|  | 4 | Lower respiratory infections | 117.8 (95.4, 146.0) |  | Lower respiratory infections | 78.5 (58.4, 103.5) |
|  | 5 | Ischemic heart disease | 69.6 (50.2, 92.4) |  | HIV/AIDS | 71.3 (47.5, 105.5) |
| Dominica | 1 | Ischemic heart disease | 151.5 (140.1, 162.3) |  | COVID-19 | 145.2 (62.2, 259.4) |
|  | 2 | Stroke | 126.0 (115.3, 136.2) |  | Stroke | 93.2 (82.5, 106.6) |
|  | 3 | Diabetes mellitus | 72.7 (66.6, 78.6) |  | Ischemic heart disease | 92.2 (81.5, 107.8) |
|  | 4 | Hypertensive heart disease | 57.5 (48.8, 66.9) |  | Diabetes mellitus | 74.6 (65.0, 87.0) |
|  | 5 | Lower respiratory infections | 40.9 (36.1, 46.3) |  | Chronic kidney disease | 50.3 (40.5, 61.8) |
| Dominican Republic | 1 | Ischemic heart disease | 150.4 (133.6, 167.9) |  | Ischemic heart disease | 144.7 (116.7, 177.9) |
|  | 2 | Stroke | 92.8 (79.9, 105.2) |  | Stroke | 72.9 (57.5, 91.7) |
|  | 3 | Neonatal disorders | 52.2 (46.1, 58.5) |  | COVID-19 | 59.1 (14.5, 134.3) |
|  | 4 | Cirrhosis and other chronic liver diseases | 36.6 (30.7, 41.5) |  | Neonatal disorders | 30.2 (24.3, 37.5) |
|  | 5 | Lower respiratory infections | 34.7 (30.5, 39.3) |  | Diabetes mellitus | 28.9 (22.8, 36.1) |
| Ecuador | 1 | Ischemic heart disease | 99.9 (94.7, 103.3) |  | COVID-19 | 174.8 (130.8, 211.2) |
|  | 2 | Stroke | 73.6 (69.8, 76.6) |  | Ischemic heart disease | 77.5 (64.1, 93.1) |
|  | 3 | Lower respiratory infections | 70.8 (66.5, 73.8) |  | Chronic kidney disease | 42.1 (32.2, 56.0) |
|  | 4 | Stomach cancer | 31.4 (29.7, 33.2) |  | Stroke | 40.8 (33.4, 49.2) |
|  | 5 | Tuberculosis | 30.4 (29.2, 31.6) |  | Lower respiratory infections | 31.1 (26.4, 36.6) |
| Egypt | 1 | Ischemic heart disease | 379.2 (343.4, 420.1) |  | Ischemic heart disease | 347.7 (297.3, 402.1) |
|  | 2 | Stroke | 272.6 (234.3, 310.1) |  | COVID-19 | 217.8 (144.6, 282.2) |
|  | 3 | Cirrhosis and other chronic liver diseases | 226.4 (186.2, 248.7) |  | Stroke | 174.1 (146.1, 208.2) |
|  | 4 | Hypertensive heart disease | 97.9 (78.6, 141.4) |  | Cirrhosis and other chronic liver diseases | 93.7 (77.9, 111.5) |
|  | 5 | Lower respiratory infections | 91.2 (80.4, 103.4) |  | Chronic kidney disease | 71.7 (59.7, 86.6) |
| El Salvador | 1 | Ischemic heart disease | 118.0 (108.5, 125.5) |  | COVID-19 | 121.2 (100.8, 141.1) |
|  | 2 | Stroke | 61.1 (56.0, 66.3) |  | Ischemic heart disease | 94.7 (78.4, 113.9) |
|  | 3 | Interpersonal violence | 53.5 (50.1, 57.0) |  | Chronic kidney disease | 69.8 (52.2, 86.8) |
|  | 4 | Lower respiratory infections | 47.8 (42.1, 53.1) |  | Interpersonal violence | 52.6 (43.2, 63.1) |
|  | 5 | Diarrheal diseases | 38.9 (29.9, 49.8) |  | Diabetes mellitus | 42.5 (33.7, 52.1) |
| Equatorial Guinea | 1 | Tuberculosis | 247.9 (167.3, 334.2) |  | COVID-19 | 260.0 (141.2, 379.1) |
|  | 2 | Diarrheal diseases | 229.0 (145.6, 347.0) |  | HIV/AIDS | 165.3 (101.7, 299.9) |
|  | 3 | Stroke | 207.9 (163.5, 257.1) |  | Ischemic heart disease | 141.8 (103.0, 188.4) |
|  | 4 | Lower respiratory infections | 174.8 (140.8, 212.3) |  | Stroke | 106.2 (71.9, 148.3) |
|  | 5 | Ischemic heart disease | 159.3 (124.1, 197.6) |  | Lower respiratory infections | 78.9 (58.9, 103.2) |
| Eritrea | 1 | Conflict and terrorism | 281.1 (231.2, 340.0) |  | COVID-19 | 202.0 (167.6, 230.2) |
|  | 2 | Tuberculosis | 280.4 (195.9, 371.3) |  | Stroke | 156.8 (124.6, 192.7) |
|  | 3 | Diarrheal diseases | 249.4 (141.4, 371.8) |  | Tuberculosis | 143.3 (95.2, 220.0) |
|  | 4 | Stroke | 234.5 (189.0, 283.8) |  | Lower respiratory infections | 136.7 (86.6, 207.2) |
|  | 5 | Lower respiratory infections | 210.0 (145.0, 296.1) |  | Ischemic heart disease | 90.9 (69.2, 117.1) |
| Estonia | 1 | Ischemic heart disease | 369.0 (347.4, 383.3) |  | Ischemic heart disease | 92.1 (80.7, 102.4) |
|  | 2 | Stroke | 170.4 (159.3, 179.3) |  | COVID-19 | 91.6 (52.3, 136.9) |
|  | 3 | Tracheal, bronchus, and lung cancer | 40.0 (37.5, 42.5) |  | Hypertensive heart disease | 68.6 (58.4, 77.0) |
|  | 4 | Road injuries | 26.9 (25.6, 28.1) |  | Stroke | 35.8 (31.0, 40.2) |
|  | 5 | Stomach cancer | 25.7 (23.7, 28.0) |  | Tracheal, bronchus, and lung cancer | 24.1 (20.3, 27.3) |
| Ethiopia | 1 | Tuberculosis | 348.3 (272.8, 413.5) |  | COVID-19 | 282.8 (258.7, 306.7) |
|  | 2 | Diarrheal diseases | 241.9 (124.5, 389.2) |  | Stroke | 81.3 (66.2, 96.0) |
|  | 3 | Lower respiratory infections | 218.1 (181.8, 259.0) |  | Lower respiratory infections | 71.6 (61.9, 82.9) |
|  | 4 | Stroke | 168.2 (141.2, 206.4) |  | Diarrheal diseases | 63.5 (37.1, 89.8) |
|  | 5 | Neonatal disorders | 100.0 (88.2, 117.5) |  | Tuberculosis | 62.0 (52.0, 74.1) |
| Fiji | 1 | Ischemic heart disease | 291.1 (256.3, 329.6) |  | Ischemic heart disease | 266.8 (217.0, 322.3) |
|  | 2 | Diabetes mellitus | 193.1 (164.2, 225.8) |  | Diabetes mellitus | 266.1 (213.7, 329.4) |
|  | 3 | Stroke | 163.3 (141.7, 189.0) |  | COVID-19 | 179.8 (108.9, 357.0) |
|  | 4 | Asthma | 73.7 (61.2, 89.5) |  | Stroke | 129.4 (105.5, 158.8) |
|  | 5 | Chronic obstructive pulmonary disease | 61.1 (51.2, 72.5) |  | Chronic kidney disease | 48.0 (36.4, 61.9) |
| Gabon | 1 | Stroke | 159.1 (127.5, 192.2) |  | COVID-19 | 239.5 (177.5, 284.6) |
|  | 2 | Ischemic heart disease | 135.3 (108.1, 162.1) |  | Ischemic heart disease | 126.8 (99.3, 155.2) |
|  | 3 | Lower respiratory infections | 118.4 (91.7, 141.9) |  | Stroke | 116.8 (89.3, 146.6) |
|  | 4 | Tuberculosis | 93.0 (54.7, 131.6) |  | Lower respiratory infections | 76.5 (55.5, 100.0) |
|  | 5 | Diarrheal diseases | 82.6 (46.5, 135.6) |  | Diabetes mellitus | 75.1 (57.6, 96.8) |
| Gambia | 1 | Stroke | 166.3 (135.9, 203.6) |  | COVID-19 | 217.5 (180.6, 256.0) |
|  | 2 | Lower respiratory infections | 163.5 (137.0, 194.3) |  | Stroke | 171.8 (132.8, 211.5) |
|  | 3 | Ischemic heart disease | 112.6 (90.3, 140.9) |  | Ischemic heart disease | 140.0 (108.6, 171.8) |
|  | 4 | Diarrheal diseases | 110.4 (74.8, 158.3) |  | Lower respiratory infections | 114.6 (88.0, 141.6) |
|  | 5 | Malaria | 96.1 (34.6, 182.0) |  | HIV/AIDS | 68.0 (43.5, 104.2) |
| Georgia | 1 | Ischemic heart disease | 358.6 (337.6, 375.0) |  | COVID-19 | 209.3 (151.0, 268.6) |
|  | 2 | Stroke | 192.1 (177.0, 204.3) |  | Stroke | 158.7 (141.1, 176.1) |
|  | 3 | Lower respiratory infections | 42.4 (38.1, 47.3) |  | Ischemic heart disease | 124.2 (111.0, 136.5) |
|  | 4 | Neonatal disorders | 35.8 (31.0, 40.9) |  | Hypertensive heart disease | 44.8 (39.1, 50.2) |
|  | 5 | Road injuries | 26.5 (24.8, 28.4) |  | Tracheal, bronchus, and lung cancer | 25.1 (21.7, 28.8) |
| Ghana | 1 | Stroke | 205.3 (172.9, 242.3) |  | COVID-19 | 199.3 (160.3, 239.8) |
|  | 2 | Ischemic heart disease | 142.8 (119.3, 169.6) |  | Stroke | 185.6 (149.0, 226.6) |
|  | 3 | Lower respiratory infections | 119.0 (98.5, 145.4) |  | Ischemic heart disease | 97.6 (78.4, 118.2) |
|  | 4 | Diarrheal diseases | 109.7 (70.5, 173.4) |  | Lower respiratory infections | 79.7 (63.3, 98.1) |
|  | 5 | Tuberculosis | 105.4 (82.8, 136.5) |  | Malaria | 53.7 (23.5, 106.2) |
| Greece | 1 | Stroke | 155.4 (141.5, 163.1) |  | Ischemic heart disease | 72.9 (65.2, 76.9) |
|  | 2 | Ischemic heart disease | 142.0 (132.2, 147.9) |  | COVID-19 | 58.6 (58.5, 58.6) |
|  | 3 | Tracheal, bronchus, and lung cancer | 41.4 (38.8, 44.0) |  | Stroke | 51.5 (44.9, 55.3) |
|  | 4 | Alzheimer's disease and other dementias | 24.8 (6.2, 66.6) |  | Tracheal, bronchus, and lung cancer | 36.3 (33.7, 38.8) |
|  | 5 | Chronic kidney disease | 21.3 (19.4, 22.7) |  | Alzheimer's disease and other dementias | 24.2 (6.1, 61.8) |
| Grenada | 1 | Stroke | 160.8 (147.9, 174.4) |  | COVID-19 | 224.8 (195.3, 350.8) |
|  | 2 | Ischemic heart disease | 144.0 (131.7, 156.4) |  | Ischemic heart disease | 95.4 (83.9, 105.8) |
|  | 3 | Diabetes mellitus | 85.3 (77.9, 92.5) |  | Stroke | 88.5 (78.1, 97.1) |
|  | 4 | Lower respiratory infections | 58.4 (53.0, 64.0) |  | Diabetes mellitus | 83.1 (73.2, 92.2) |
|  | 5 | Chronic kidney disease | 35.7 (32.5, 39.2) |  | Chronic kidney disease | 58.3 (51.0, 64.9) |
| Guinea | 1 | Diarrheal diseases | 255.8 (142.0, 421.1) |  | COVID-19 | 268.4 (183.2, 321.0) |
|  | 2 | Lower respiratory infections | 206.3 (171.0, 245.6) |  | Stroke | 159.0 (127.2, 194.8) |
|  | 3 | Stroke | 163.4 (133.4, 192.7) |  | Lower respiratory infections | 120.2 (97.3, 151.6) |
|  | 4 | Ischemic heart disease | 92.1 (73.8, 112.2) |  | Ischemic heart disease | 110.4 (85.9, 140.2) |
|  | 5 | Tuberculosis | 90.0 (73.2, 115.3) |  | Malaria | 90.2 (35.3, 187.9) |
| Guinea-Bissau | 1 | Stroke | 259.7 (210.5, 314.9) |  | COVID-19 | 309.2 (165.1, 437.7) |
|  | 2 | Lower respiratory infections | 214.7 (179.9, 253.0) |  | Stroke | 223.9 (178.8, 268.3) |
|  | 3 | Tuberculosis | 210.1 (156.8, 263.3) |  | Ischemic heart disease | 156.9 (123.3, 192.4) |
|  | 4 | Diarrheal diseases | 208.1 (140.2, 288.8) |  | Lower respiratory infections | 123.8 (98.2, 147.7) |
|  | 5 | Ischemic heart disease | 150.6 (121.0, 186.5) |  | Tuberculosis | 99.9 (71.6, 126.0) |
| Guyana | 1 | Stroke | 268.1 (245.9, 293.3) |  | COVID-19 | 294.6 (158.3, 524.2) |
|  | 2 | Ischemic heart disease | 230.6 (209.2, 251.9) |  | Ischemic heart disease | 143.2 (115.5, 177.3) |
|  | 3 | Hypertensive heart disease | 85.0 (77.0, 92.1) |  | Stroke | 136.7 (110.7, 167.0) |
|  | 4 | Diabetes mellitus | 79.8 (72.1, 87.3) |  | Diabetes mellitus | 85.7 (68.0, 106.2) |
|  | 5 | Neonatal disorders | 57.7 (50.6, 66.0) |  | Hypertensive heart disease | 63.5 (50.8, 77.3) |
| Hungary | 1 | Ischemic heart disease | 262.8 (250.2, 271.3) |  | Ischemic heart disease | 155.4 (137.2, 170.6) |
|  | 2 | Stroke | 188.8 (178.2, 197.8) |  | COVID-19 | 154.5 (104.9, 205.8) |
|  | 3 | Tracheal, bronchus, and lung cancer | 48.4 (44.0, 52.8) |  | Stroke | 55.3 (47.8, 62.6) |
|  | 4 | Cirrhosis and other chronic liver diseases | 42.1 (40.1, 44.4) |  | Tracheal, bronchus, and lung cancer | 44.1 (37.8, 50.6) |
|  | 5 | Chronic obstructive pulmonary disease | 34.9 (32.8, 37.1) |  | Chronic obstructive pulmonary disease | 29.2 (25.6, 33.3) |
| Indonesia | 1 | Stroke | 206.2 (174.0, 242.0) |  | Stroke | 217.7 (182.0, 245.4) |
|  | 2 | Diarrheal diseases | 193.0 (109.4, 299.1) |  | Ischemic heart disease | 143.3 (119.3, 163.3) |
|  | 3 | Tuberculosis | 105.8 (75.1, 124.8) |  | COVID-19 | 120.2 (64.1, 229.5) |
|  | 4 | Ischemic heart disease | 103.0 (85.9, 119.9) |  | Chronic obstructive pulmonary disease | 49.5 (40.9, 58.2) |
|  | 5 | Cirrhosis and other chronic liver diseases | 61.2 (45.7, 86.1) |  | Cirrhosis and other chronic liver diseases | 44.8 (36.5, 52.5) |
| Iran (Islamic Republic of) | 1 | Ischemic heart disease | 259.9 (238.1, 276.2) |  | COVID-19 | 223.2 (214.2, 235.3) |
|  | 2 | Stroke | 124.7 (111.6, 135.1) |  | Ischemic heart disease | 146.1 (130.7, 157.0) |
|  | 3 | Exposure to forces of nature | 69.2 (62.9, 76.1) |  | Stroke | 63.9 (56.8, 69.7) |
|  | 4 | Road injuries | 57.3 (53.2, 62.3) |  | Hypertensive heart disease | 28.9 (24.2, 34.9) |
|  | 5 | Neonatal disorders | 56.9 (48.6, 67.4) |  | Alzheimer's disease and other dementias | 25.2 (6.3, 66.3) |
| Iraq | 1 | Ischemic heart disease | 249.7 (213.9, 290.9) |  | Ischemic heart disease | 254.8 (204.2, 296.7) |
|  | 2 | Stroke | 198.6 (167.7, 231.4) |  | COVID-19 | 173.0 (123.4, 218.0) |
|  | 3 | Neonatal disorders | 53.7 (47.9, 60.9) |  | Stroke | 170.0 (136.9, 201.3) |
|  | 4 | Diabetes mellitus | 40.5 (33.5, 49.8) |  | Diabetes mellitus | 48.5 (38.5, 58.2) |
|  | 5 | Hypertensive heart disease | 37.4 (23.9, 50.5) |  | Chronic kidney disease | 43.4 (30.5, 54.8) |
| Italy | 1 | Ischemic heart disease | 108.5 (97.9, 113.9) |  | Ischemic heart disease | 44.3 (37.6, 47.8) |
|  | 2 | Stroke | 87.5 (77.5, 92.6) |  | COVID-19 | 39.0 (37.0, 41.2) |
|  | 3 | Tracheal, bronchus, and lung cancer | 36.5 (35.1, 37.6) |  | Stroke | 30.7 (25.3, 33.6) |
|  | 4 | Alzheimer's disease and other dementias | 31.5 (8.1, 81.3) |  | Alzheimer's disease and other dementias | 29.7 (8.1, 72.9) |
|  | 5 | Cardiomyopathy and myocarditis | 28.9 (24.8, 31.0) |  | Tracheal, bronchus, and lung cancer | 24.2 (22.2, 25.7) |
| Jamaica | 1 | Stroke | 120.8 (113.8, 126.3) |  | COVID-19 | 106.8 (64.4, 151.6) |
|  | 2 | Ischemic heart disease | 71.4 (66.4, 74.7) |  | Stroke | 87.3 (69.6, 107.7) |
|  | 3 | Diabetes mellitus | 67.8 (64.5, 71.4) |  | Diabetes mellitus | 67.7 (53.1, 85.0) |
|  | 4 | Hypertensive heart disease | 42.4 (39.2, 44.9) |  | Ischemic heart disease | 52.4 (42.1, 66.2) |
|  | 5 | Neonatal disorders | 32.1 (27.2, 37.3) |  | Hypertensive heart disease | 30.8 (24.4, 38.7) |
| Kazakhstan | 1 | Ischemic heart disease | 298.7 (276.2, 320.0) |  | COVID-19 | 241.5 (191.4, 283.2) |
|  | 2 | Stroke | 181.2 (165.1, 195.8) |  | Ischemic heart disease | 236.0 (212.0, 258.9) |
|  | 3 | Chronic obstructive pulmonary disease | 45.2 (40.4, 50.0) |  | Stroke | 163.0 (144.2, 183.1) |
|  | 4 | Tracheal, bronchus, and lung cancer | 42.0 (38.8, 45.4) |  | Chronic obstructive pulmonary disease | 46.7 (40.3, 54.1) |
|  | 5 | Lower respiratory infections | 37.4 (34.5, 40.7) |  | Cardiomyopathy and myocarditis | 41.8 (34.1, 49.2) |
| Kenya | 1 | Tuberculosis | 140.6 (81.6, 225.2) |  | COVID-19 | 301.2 (269.7, 330.3) |
|  | 2 | Diarrheal diseases | 132.4 (82.7, 195.3) |  | Stroke | 100.9 (76.6, 126.5) |
|  | 3 | Lower respiratory infections | 112.7 (93.3, 138.2) |  | Tuberculosis | 96.2 (56.5, 134.6) |
|  | 4 | HIV/AIDS | 105.6 (66.5, 166.5) |  | Lower respiratory infections | 85.2 (66.0, 107.3) |
|  | 5 | Stroke | 101.6 (77.5, 124.4) |  | HIV/AIDS | 81.6 (68.0, 101.4) |
| Kiribati | 1 | Stroke | 204.8 (166.8, 242.1) |  | Ischemic heart disease | 202.9 (169.3, 243.4) |
|  | 2 | Diarrheal diseases | 193.7 (109.1, 281.7) |  | Stroke | 186.0 (155.5, 223.8) |
|  | 3 | Ischemic heart disease | 189.6 (154.8, 225.4) |  | Diabetes mellitus | 184.9 (143.7, 232.5) |
|  | 4 | Tuberculosis | 172.4 (141.1, 206.1) |  | Tuberculosis | 102.5 (80.4, 137.3) |
|  | 5 | Diabetes mellitus | 142.1 (118.1, 167.4) |  | Diarrheal diseases | 85.7 (43.9, 129.2) |
| Kuwait | 1 | Ischemic heart disease | 198.3 (179.2, 213.0) |  | Ischemic heart disease | 109.1 (90.4, 131.4) |
|  | 2 | Conflict and terrorism | 66.2 (60.2, 72.8) |  | COVID-19 | 59.4 (54.6, 75.4) |
|  | 3 | Stroke | 50.6 (44.9, 54.7) |  | Stroke | 34.0 (28.0, 40.8) |
|  | 4 | Hypertensive heart disease | 41.8 (36.1, 45.7) |  | Diabetes mellitus | 25.3 (20.8, 30.3) |
|  | 5 | Chronic kidney disease | 30.7 (26.6, 34.3) |  | Lower respiratory infections | 24.6 (20.2, 28.7) |
| Kyrgyzstan | 1 | Ischemic heart disease | 264.4 (242.4, 284.7) |  | Ischemic heart disease | 274.4 (234.2, 313.7) |
|  | 2 | Stroke | 199.2 (182.5, 214.3) |  | COVID-19 | 105.4 (71.7, 144.6) |
|  | 3 | Chronic obstructive pulmonary disease | 95.5 (88.0, 103.1) |  | Stroke | 93.1 (79.4, 107.4) |
|  | 4 | Lower respiratory infections | 64.9 (59.4, 71.1) |  | Cirrhosis and other chronic liver diseases | 33.2 (27.5, 38.8) |
|  | 5 | Cirrhosis and other chronic liver diseases | 35.6 (32.9, 38.3) |  | Chronic obstructive pulmonary disease | 28.2 (23.9, 32.7) |
| Lao People's Democratic Republic | 1 | Stroke | 297.3 (239.4, 363.3) |  | Stroke | 183.7 (148.5, 223.8) |
|  | 2 | Ischemic heart disease | 235.3 (187.6, 286.5) |  | Ischemic heart disease | 176.5 (141.7, 213.3) |
|  | 3 | Lower respiratory infections | 175.9 (147.6, 211.5) |  | COVID-19 | 80.0 (31.1, 160.5) |
|  | 4 | Diarrheal diseases | 158.4 (104.8, 257.2) |  | Lower respiratory infections | 62.7 (48.3, 77.5) |
|  | 5 | Tuberculosis | 152.6 (94.0, 204.8) |  | Chronic obstructive pulmonary disease | 56.4 (41.3, 72.8) |
| Latvia | 1 | Ischemic heart disease | 343.1 (322.5, 356.7) |  | Ischemic heart disease | 160.3 (141.8, 177.1) |
|  | 2 | Stroke | 199.9 (187.4, 209.5) |  | COVID-19 | 140.4 (90.5, 192.3) |
|  | 3 | Road injuries | 36.4 (35.1, 37.6) |  | Stroke | 106.1 (93.4, 117.2) |
|  | 4 | Tracheal, bronchus, and lung cancer | 35.4 (32.9, 38.2) |  | Cardiomyopathy and myocarditis | 27.9 (23.9, 32.0) |
|  | 5 | Self-harm | 25.1 (23.9, 26.3) |  | Tracheal, bronchus, and lung cancer | 23.3 (19.8, 26.6) |
| Lebanon | 1 | Ischemic heart disease | 235.3 (199.0, 287.0) |  | COVID-19 | 213.8 (198.4, 231.3) |
|  | 2 | Stroke | 141.9 (114.3, 177.0) |  | Ischemic heart disease | 92.1 (77.7, 108.0) |
|  | 3 | Interpersonal violence | 45.9 (43.1, 49.0) |  | Stroke | 44.5 (36.8, 52.4) |
|  | 4 | Diabetes mellitus | 37.8 (31.8, 45.5) |  | Chronic kidney disease | 31.9 (26.3, 38.5) |
|  | 5 | Chronic kidney disease | 32.3 (24.7, 42.7) |  | Diabetes mellitus | 29.6 (21.7, 37.0) |
| Lesotho | 1 | Diarrheal diseases | 209.5 (130.9, 300.8) |  | COVID-19 | 894.6 (725.9, 1045.7) |
|  | 2 | Tuberculosis | 153.5 (100.1, 219.0) |  | HIV/AIDS | 422.9 (353.0, 506.6) |
|  | 3 | Stroke | 148.7 (117.6, 182.2) |  | Tuberculosis | 213.4 (143.1, 274.3) |
|  | 4 | Lower respiratory infections | 109.1 (89.3, 133.5) |  | Stroke | 213.3 (165.0, 271.2) |
|  | 5 | Neonatal disorders | 64.2 (56.3, 73.6) |  | Lower respiratory infections | 118.5 (96.3, 141.0) |
| Liberia | 1 | Diarrheal diseases | 176.8 (127.3, 234.8) |  | COVID-19 | 169.2 (142.0, 198.0) |
|  | 2 | Stroke | 167.4 (143.4, 193.9) |  | Stroke | 148.6 (118.1, 187.6) |
|  | 3 | Lower respiratory infections | 167.0 (138.2, 201.1) |  | Malaria | 136.3 (44.0, 281.0) |
|  | 4 | Malaria | 165.0 (63.8, 313.9) |  | Ischemic heart disease | 114.2 (90.1, 145.7) |
|  | 5 | Conflict and terrorism | 145.9 (138.8, 153.4) |  | Lower respiratory infections | 74.6 (57.4, 95.9) |
| Libya | 1 | Ischemic heart disease | 162.1 (128.6, 199.6) |  | Ischemic heart disease | 178.5 (140.3, 228.1) |
|  | 2 | Stroke | 82.2 (63.9, 107.0) |  | COVID-19 | 153.3 (118.8, 190.1) |
|  | 3 | Road injuries | 46.6 (37.8, 55.5) |  | Stroke | 79.1 (58.8, 105.1) |
|  | 4 | Hypertensive heart disease | 31.3 (19.4, 44.8) |  | Chronic kidney disease | 46.9 (31.3, 61.1) |
|  | 5 | Alzheimer's disease and other dementias | 30.8 (8.0, 76.8) |  | Road injuries | 40.2 (31.7, 50.5) |
| Lithuania | 1 | Ischemic heart disease | 365.3 (343.2, 379.0) |  | Ischemic heart disease | 197.1 (176.6, 215.9) |
|  | 2 | Stroke | 97.7 (91.3, 103.1) |  | COVID-19 | 121.9 (75.6, 172.5) |
|  | 3 | Tracheal, bronchus, and lung cancer | 33.6 (31.4, 35.4) |  | Stroke | 68.2 (60.4, 75.4) |
|  | 4 | Road injuries | 29.8 (28.8, 30.7) |  | Tracheal, bronchus, and lung cancer | 23.6 (20.0, 27.2) |
|  | 5 | Chronic obstructive pulmonary disease | 28.7 (26.7, 30.5) |  | Alzheimer's disease and other dementias | 21.0 (5.2, 56.8) |
| Luxembourg | 1 | Ischemic heart disease | 153.8 (144.6, 160.4) |  | Ischemic heart disease | 45.7 (40.6, 50.2) |
|  | 2 | Stroke | 125.5 (117.3, 131.3) |  | COVID-19 | 36.6 (28.4, 44.5) |
|  | 3 | Tracheal, bronchus, and lung cancer | 42.3 (39.6, 44.9) |  | Tracheal, bronchus, and lung cancer | 26.4 (23.6, 29.3) |
|  | 4 | Colon and rectum cancer | 26.7 (24.8, 28.4) |  | Stroke | 26.1 (22.5, 28.9) |
|  | 5 | Chronic obstructive pulmonary disease | 25.9 (24.3, 27.3) |  | Chronic obstructive pulmonary disease | 18.6 (16.4, 20.8) |
| Madagascar | 1 | Stroke | 251.5 (219.2, 283.8) |  | COVID-19 | 257.0 (211.0, 285.4) |
|  | 2 | Tuberculosis | 162.9 (125.8, 201.9) |  | Stroke | 203.9 (154.1, 256.2) |
|  | 3 | Lower respiratory infections | 153.5 (139.0, 168.6) |  | Ischemic heart disease | 100.0 (75.0, 125.9) |
|  | 4 | Diarrheal diseases | 150.7 (113.0, 209.9) |  | Lower respiratory infections | 90.6 (70.8, 108.4) |
|  | 5 | Protein-energy malnutrition | 86.9 (76.1, 97.9) |  | Tuberculosis | 83.0 (59.4, 118.7) |
| Malaysia | 1 | Ischemic heart disease | 177.5 (162.8, 189.8) |  | Ischemic heart disease | 149.7 (138.2, 159.7) |
|  | 2 | Stroke | 140.2 (127.3, 151.8) |  | COVID-19 | 127.4 (122.0, 156.5) |
|  | 3 | Lower respiratory infections | 56.2 (48.4, 69.7) |  | Stroke | 94.3 (84.5, 104.8) |
|  | 4 | Chronic obstructive pulmonary disease | 41.7 (35.8, 48.1) |  | Lower respiratory infections | 75.9 (62.1, 86.6) |
|  | 5 | Road injuries | 29.3 (27.2, 31.7) |  | Chronic kidney disease | 29.2 (24.4, 33.0) |
| Maldives | 1 | Stroke | 226.8 (200.4, 251.0) |  | Stroke | 80.1 (66.6, 92.8) |
|  | 2 | Ischemic heart disease | 168.0 (147.8, 188.2) |  | Ischemic heart disease | 79.4 (66.2, 93.8) |
|  | 3 | Chronic obstructive pulmonary disease | 91.8 (62.3, 111.2) |  | COVID-19 | 66.5 (64.9, 87.3) |
|  | 4 | Diarrheal diseases | 74.1 (50.6, 104.6) |  | Chronic obstructive pulmonary disease | 35.0 (29.1, 42.5) |
|  | 5 | Tuberculosis | 67.5 (45.6, 97.7) |  | Chronic kidney disease | 28.9 (23.4, 34.5) |
| Mali | 1 | Diarrheal diseases | 221.4 (143.9, 306.0) |  | COVID-19 | 347.6 (315.6, 382.4) |
|  | 2 | Malaria | 161.9 (82.5, 298.8) |  | Stroke | 116.1 (94.1, 142.3) |
|  | 3 | Stroke | 147.6 (118.1, 173.1) |  | Malaria | 91.3 (39.4, 195.2) |
|  | 4 | Tuberculosis | 133.1 (114.5, 152.8) |  | Neonatal disorders | 76.6 (64.6, 90.6) |
|  | 5 | Neonatal disorders | 120.6 (106.6, 134.9) |  | Ischemic heart disease | 71.7 (56.2, 91.0) |
| Malta | 1 | Ischemic heart disease | 207.7 (192.1, 219.0) |  | Ischemic heart disease | 71.2 (61.3, 78.5) |
|  | 2 | Stroke | 86.6 (79.3, 92.0) |  | COVID-19 | 25.3 (22.7, 28.5) |
|  | 3 | Tracheal, bronchus, and lung cancer | 27.6 (25.4, 30.1) |  | Alzheimer's disease and other dementias | 24.0 (6.2, 59.1) |
|  | 4 | Diabetes mellitus | 25.4 (23.3, 27.5) |  | Stroke | 23.6 (19.7, 26.3) |
|  | 5 | Alzheimer's disease and other dementias | 24.7 (6.1, 65.7) |  | Tracheal, bronchus, and lung cancer | 18.9 (16.7, 21.3) |
| Mauritania | 1 | Stroke | 194.0 (158.3, 241.6) |  | Stroke | 131.1 (101.1, 171.4) |
|  | 2 | Ischemic heart disease | 136.7 (109.2, 169.8) |  | COVID-19 | 118.9 (81.4, 149.1) |
|  | 3 | Lower respiratory infections | 133.2 (111.6, 155.1) |  | Ischemic heart disease | 112.2 (85.3, 144.3) |
|  | 4 | Diarrheal diseases | 112.0 (70.5, 151.8) |  | Lower respiratory infections | 75.5 (58.7, 94.1) |
|  | 5 | Tuberculosis | 84.3 (63.6, 106.6) |  | Chronic kidney disease | 40.4 (27.5, 56.5) |
| Micronesia  (Federated States of) | 1 | Ischemic heart disease | 268.0 (220.5, 324.3) |  | Ischemic heart disease | 266.4 (213.7, 330.4) |
|  | 2 | Stroke | 264.4 (214.3, 312.1) |  | Stroke | 196.2 (158.1, 242.6) |
|  | 3 | Chronic obstructive pulmonary disease | 115.4 (85.1, 149.4) |  | Diabetes mellitus | 117.8 (91.3, 150.8) |
|  | 4 | Lower respiratory infections | 88.9 (73.9, 105.5) |  | Chronic obstructive pulmonary disease | 67.1 (53.3, 86.2) |
|  | 5 | Diabetes mellitus | 87.9 (70.8, 109.7) |  | Lower respiratory infections | 57.7 (46.5, 72.4) |
| Mongolia | 1 | Ischemic heart disease | 324.8 (286.6, 365.7) |  | Ischemic heart disease | 219.6 (190.3, 247.4) |
|  | 2 | Stroke | 214.9 (183.4, 249.3) |  | Stroke | 134.1 (109.1, 159.6) |
|  | 3 | Lower respiratory infections | 101.5 (87.7, 115.3) |  | COVID-19 | 116.6 (59.5, 193.7) |
|  | 4 | Liver cancer | 60.4 (42.2, 84.1) |  | Liver cancer | 80.9 (62.1, 102.6) |
|  | 5 | Cirrhosis and other chronic liver diseases | 57.7 (49.1, 69.7) |  | Stomach cancer | 37.4 (29.4, 45.9) |
| Montenegro | 1 | Stroke | 184.2 (162.2, 206.1) |  | COVID-19 | 271.4 (224.6, 316.1) |
|  | 2 | Ischemic heart disease | 164.6 (142.8, 183.4) |  | Stroke | 225.8 (196.2, 259.4) |
|  | 3 | Tracheal, bronchus, and lung cancer | 43.5 (36.1, 51.9) |  | Ischemic heart disease | 212.8 (186.7, 239.2) |
|  | 4 | Cardiomyopathy and myocarditis | 24.9 (17.1, 33.8) |  | Tracheal, bronchus, and lung cancer | 48.4 (38.0, 60.0) |
|  | 5 | Alzheimer's disease and other dementias | 20.6 (4.9, 55.8) |  | Cardiomyopathy and myocarditis | 28.3 (18.0, 37.3) |
| Morocco | 1 | Ischemic heart disease | 296.2 (254.4, 342.6) |  | Ischemic heart disease | 267.2 (210.8, 310.9) |
|  | 2 | Stroke | 151.9 (117.8, 189.2) |  | Stroke | 126.7 (99.4, 158.2) |
|  | 3 | Hypertensive heart disease | 53.9 (32.3, 75.5) |  | COVID-19 | 110.7 (87.6, 138.4) |
|  | 4 | Lower respiratory infections | 47.9 (40.4, 57.2) |  | Hypertensive heart disease | 48.9 (31.2, 67.3) |
|  | 5 | Neonatal disorders | 47.5 (40.7, 53.9) |  | Chronic kidney disease | 40.0 (30.3, 51.3) |
| Mozambique | 1 | Tuberculosis | 223.5 (167.6, 308.5) |  | COVID-19 | 409.1 (316.0, 484.2) |
|  | 2 | Malaria | 214.9 (119.6, 376.9) |  | Stroke | 222.4 (173.0, 271.4) |
|  | 3 | Stroke | 206.1 (181.9, 235.1) |  | HIV/AIDS | 167.1 (148.7, 194.2) |
|  | 4 | Diarrheal diseases | 156.8 (93.5, 222.9) |  | Tuberculosis | 138.1 (96.7, 180.2) |
|  | 5 | Lower respiratory infections | 136.1 (115.0, 161.2) |  | Malaria | 115.0 (34.0, 264.0) |
| Myanmar | 1 | Stroke | 289.9 (239.1, 348.0) |  | Stroke | 181.1 (147.6, 222.2) |
|  | 2 | Ischemic heart disease | 193.3 (157.0, 238.0) |  | Ischemic heart disease | 138.2 (113.0, 171.6) |
|  | 3 | Tuberculosis | 160.9 (126.0, 201.7) |  | Chronic obstructive pulmonary disease | 104.5 (82.5, 126.4) |
|  | 4 | Chronic obstructive pulmonary disease | 135.5 (99.6, 173.1) |  | COVID-19 | 99.5 (38.5, 200.7) |
|  | 5 | Lower respiratory infections | 132.7 (110.1, 159.9) |  | Diabetes mellitus | 57.1 (46.7, 70.8) |
| Namibia | 1 | Stroke | 190.8 (161.0, 220.5) |  | COVID-19 | 540.6 (453.0, 641.9) |
|  | 2 | Diarrheal diseases | 139.0 (86.7, 205.8) |  | Stroke | 154.7 (120.2, 190.1) |
|  | 3 | Tuberculosis | 137.3 (89.3, 184.3) |  | HIV/AIDS | 138.9 (114.2, 178.4) |
|  | 4 | Lower respiratory infections | 125.3 (95.5, 163.6) |  | Ischemic heart disease | 104.2 (81.4, 129.1) |
|  | 5 | Ischemic heart disease | 104.5 (84.8, 124.6) |  | Lower respiratory infections | 82.8 (61.7, 109.5) |
| Nepal | 1 | Chronic obstructive pulmonary disease | 176.1 (121.7, 222.6) |  | COVID-19 | 218.7 (205.2, 247.5) |
|  | 2 | Diarrheal diseases | 144.7 (103.9, 210.7) |  | Chronic obstructive pulmonary disease | 146.1 (116.7, 182.5) |
|  | 3 | Tuberculosis | 144.5 (102.4, 192.4) |  | Ischemic heart disease | 136.5 (111.7, 169.9) |
|  | 4 | Ischemic heart disease | 130.1 (102.8, 160.8) |  | Stroke | 86.4 (69.9, 108.3) |
|  | 5 | Stroke | 126.9 (103.7, 153.5) |  | Neonatal disorders | 33.5 (25.5, 43.3) |
| New Zealand | 1 | Ischemic heart disease | 185.4 (172.4, 193.6) |  | Ischemic heart disease | 61.0 (52.8, 65.4) |
|  | 2 | Stroke | 64.5 (58.0, 69.0) |  | Stroke | 28.7 (24.2, 31.5) |
|  | 3 | Tracheal, bronchus, and lung cancer | 35.8 (33.3, 38.4) |  | Alzheimer's disease and other dementias | 24.8 (6.6, 64.0) |
|  | 4 | Chronic obstructive pulmonary disease | 32.7 (30.3, 34.2) |  | Tracheal, bronchus, and lung cancer | 23.3 (21.1, 25.5) |
|  | 5 | Colon and rectum cancer | 30.3 (27.3, 33.4) |  | Chronic obstructive pulmonary disease | 22.0 (19.2, 23.8) |
| Nicaragua | 1 | Ischemic heart disease | 89.2 (80.6, 96.2) |  | COVID-19 | 274.0 (257.4, 296.3) |
|  | 2 | Stroke | 61.9 (55.8, 67.6) |  | Ischemic heart disease | 81.9 (70.2, 95.0) |
|  | 3 | Diarrheal diseases | 44.5 (33.7, 56.8) |  | Chronic kidney disease | 55.3 (44.7, 66.5) |
|  | 4 | Lower respiratory infections | 40.2 (35.9, 44.4) |  | Stroke | 33.5 (28.3, 40.9) |
|  | 5 | Neonatal disorders | 35.2 (30.5, 39.7) |  | Diabetes mellitus | 30.2 (25.5, 36.4) |
| Niger | 1 | Diarrheal diseases | 335.4 (219.9, 461.2) |  | COVID-19 | 174.2 (131.7, 207.1) |
|  | 2 | Lower respiratory infections | 244.9 (206.7, 292.7) |  | Malaria | 140.0 (44.8, 273.2) |
|  | 3 | Malaria | 166.0 (62.5, 340.3) |  | Stroke | 138.1 (109.6, 172.6) |
|  | 4 | Stroke | 154.9 (120.4, 187.2) |  | Lower respiratory infections | 101.3 (80.1, 127.3) |
|  | 5 | Tuberculosis | 147.7 (107.3, 210.2) |  | Diarrheal diseases | 100.0 (60.1, 151.9) |
| Nigeria | 1 | Diarrheal diseases | 196.1 (132.4, 267.2) |  | COVID-19 | 158.6 (141.9, 178.8) |
|  | 2 | Stroke | 170.0 (141.8, 203.4) |  | Malaria | 112.2 (36.4, 242.7) |
|  | 3 | Lower respiratory infections | 149.4 (128.1, 173.6) |  | Ischemic heart disease | 109.5 (90.7, 129.2) |
|  | 4 | Malaria | 143.0 (71.0, 251.5) |  | Stroke | 102.5 (85.8, 124.5) |
|  | 5 | Tuberculosis | 110.8 (85.1, 148.4) |  | Lower respiratory infections | 77.5 (62.0, 94.4) |
| Niue | 1 | Ischemic heart disease | 241.3 (204.6, 282.9) |  | Ischemic heart disease | 243.5 (206.5, 279.7) |
|  | 2 | Stroke | 179.5 (150.9, 210.8) |  | Stroke | 133.9 (112.9, 154.9) |
|  | 3 | Diabetes mellitus | 83.5 (69.0, 100.9) |  | Diabetes mellitus | 122.5 (94.1, 155.9) |
|  | 4 | Chronic obstructive pulmonary disease | 74.4 (62.9, 88.2) |  | Lower respiratory infections | 66.2 (58.6, 75.6) |
|  | 5 | Lower respiratory infections | 59.9 (49.8, 71.0) |  | Chronic kidney disease | 56.1 (33.1, 86.2) |
| North Macedonia | 1 | Stroke | 282.5 (257.1, 309.2) |  | Stroke | 277.5 (235.0, 321.9) |
|  | 2 | Ischemic heart disease | 208.6 (187.1, 232.7) |  | COVID-19 | 271.1 (199.4, 319.9) |
|  | 3 | Hypertensive heart disease | 39.7 (30.9, 49.4) |  | Ischemic heart disease | 195.9 (170.1, 224.3) |
|  | 4 | Neonatal disorders | 36.7 (33.7, 40.2) |  | Hypertensive heart disease | 45.8 (32.9, 59.1) |
|  | 5 | Tracheal, bronchus, and lung cancer | 30.1 (25.3, 35.1) |  | Diabetes mellitus | 33.3 (26.6, 40.1) |
| Oman | 1 | Ischemic heart disease | 301.2 (238.0, 376.6) |  | COVID-19 | 213.9 (201.9, 224.8) |
|  | 2 | Stroke | 126.4 (97.0, 159.6) |  | Ischemic heart disease | 179.3 (150.3, 211.7) |
|  | 3 | Road injuries | 94.4 (73.6, 119.4) |  | Stroke | 74.2 (60.0, 87.0) |
|  | 4 | Hypertensive heart disease | 49.8 (34.8, 72.6) |  | Diabetes mellitus | 47.1 (37.7, 57.1) |
|  | 5 | Lower respiratory infections | 42.4 (33.6, 54.0) |  | Hypertensive heart disease | 43.9 (32.5, 55.2) |
| Pakistan | 1 | Ischemic heart disease | 140.7 (111.6, 162.5) |  | COVID-19 | 207.7 (176.3, 254.0) |
|  | 2 | Diarrheal diseases | 133.6 (83.5, 219.3) |  | Ischemic heart disease | 183.4 (154.6, 226.8) |
|  | 3 | Tuberculosis | 109.5 (64.7, 154.2) |  | Stroke | 98.0 (81.1, 119.4) |
|  | 4 | Stroke | 106.6 (86.9, 123.7) |  | Chronic obstructive pulmonary disease | 73.8 (60.0, 94.6) |
|  | 5 | Chronic obstructive pulmonary disease | 84.0 (64.4, 98.3) |  | Neonatal disorders | 69.2 (57.4, 83.5) |
| Panama | 1 | Ischemic heart disease | 105.3 (97.3, 110.9) |  | COVID-19 | 75.5 (74.7, 86.9) |
|  | 2 | Stroke | 71.0 (65.7, 74.6) |  | Ischemic heart disease | 54.5 (43.0, 64.9) |
|  | 3 | Lower respiratory infections | 24.8 (23.0, 26.3) |  | Stroke | 43.6 (34.0, 51.9) |
|  | 4 | Road injuries | 22.2 (21.3, 23.2) |  | Chronic kidney disease | 30.7 (24.3, 36.8) |
|  | 5 | Diabetes mellitus | 20.9 (19.5, 22.2) |  | Diabetes mellitus | 28.0 (22.4, 33.5) |
| Papua New Guinea | 1 | Stroke | 217.7 (169.4, 269.9) |  | COVID-19 | 207.8 (45.4, 409.5) |
|  | 2 | Chronic obstructive pulmonary disease | 188.5 (140.8, 243.2) |  | Stroke | 173.3 (134.6, 217.4) |
|  | 3 | Ischemic heart disease | 136.5 (103.0, 179.1) |  | Chronic obstructive pulmonary disease | 156.8 (123.6, 197.4) |
|  | 4 | Diarrheal diseases | 101.7 (61.9, 141.6) |  | Ischemic heart disease | 144.4 (109.8, 186.3) |
|  | 5 | Lower respiratory infections | 101.0 (85.8, 120.7) |  | Diabetes mellitus | 90.0 (70.2, 113.5) |
| Peru | 1 | Lower respiratory infections | 115.3 (102.3, 127.8) |  | COVID-19 | 342.5 (339.8, 346.5) |
|  | 2 | Ischemic heart disease | 79.0 (69.2, 89.6) |  | Lower respiratory infections | 60.1 (46.8, 75.5) |
|  | 3 | Stroke | 62.4 (54.1, 71.6) |  | Ischemic heart disease | 44.8 (34.9, 55.9) |
|  | 4 | Tuberculosis | 48.7 (41.1, 58.3) |  | Stroke | 31.4 (24.5, 39.6) |
|  | 5 | Neonatal disorders | 37.7 (33.4, 43.1) |  | Chronic kidney disease | 30.9 (22.1, 39.7) |
| Philippines | 1 | Ischemic heart disease | 174.1 (159.0, 186.4) |  | COVID-19 | 227.4 (225.2, 231.4) |
|  | 2 | Stroke | 133.6 (123.3, 144.6) |  | Ischemic heart disease | 150.4 (129.2, 171.9) |
|  | 3 | Lower respiratory infections | 85.9 (78.6, 98.7) |  | Stroke | 114.8 (98.7, 131.2) |
|  | 4 | Tuberculosis | 57.4 (46.4, 70.3) |  | Lower respiratory infections | 77.8 (62.2, 89.6) |
|  | 5 | Chronic obstructive pulmonary disease | 46.6 (41.9, 51.3) |  | Chronic kidney disease | 41.7 (35.0, 48.1) |
| Poland | 1 | Ischemic heart disease | 269.8 (255.7, 277.0) |  | COVID-19 | 114.0 (82.9, 151.7) |
|  | 2 | Stroke | 168.0 (157.8, 174.0) |  | Ischemic heart disease | 113.3 (100.9, 122.6) |
|  | 3 | Tracheal, bronchus, and lung cancer | 45.3 (44.1, 46.5) |  | Stroke | 58.0 (51.3, 63.3) |
|  | 4 | Chronic obstructive pulmonary disease | 25.2 (24.1, 25.9) |  | Tracheal, bronchus, and lung cancer | 41.6 (37.8, 45.3) |
|  | 5 | Road injuries | 23.5 (22.8, 24.2) |  | Colon and rectum cancer | 24.3 (21.9, 26.5) |
| Portugal | 1 | Stroke | 225.5 (208.9, 235.0) |  | Stroke | 44.3 (38.1, 48.2) |
|  | 2 | Ischemic heart disease | 116.9 (109.2, 121.4) |  | COVID-19 | 44.0 (42.2, 45.9) |
|  | 3 | Road injuries | 31.1 (30.3, 31.8) |  | Ischemic heart disease | 36.8 (32.4, 39.4) |
|  | 4 | Stomach cancer | 30.3 (28.1, 32.1) |  | Alzheimer's disease and other dementias | 24.8 (6.5, 62.1) |
|  | 5 | Chronic obstructive pulmonary disease | 28.3 (26.4, 30.1) |  | Tracheal, bronchus, and lung cancer | 19.8 (18.1, 21.5) |
| Qatar | 1 | Ischemic heart disease | 384.9 (334.5, 437.7) |  | Ischemic heart disease | 123.2 (97.9, 147.9) |
|  | 2 | Stroke | 158.1 (135.1, 184.1) |  | Diabetes mellitus | 77.1 (61.0, 95.9) |
|  | 3 | Diabetes mellitus | 85.7 (69.9, 105.3) |  | COVID-19 | 66.2 (48.6, 80.2) |
|  | 4 | Road injuries | 58.9 (50.1, 69.8) |  | Stroke | 54.8 (43.6, 66.7) |
|  | 5 | Cirrhosis and other chronic liver diseases | 46.2 (36.4, 59.1) |  | Chronic kidney disease | 39.9 (32.4, 49.2) |
| Republic of Korea | 1 | Stroke | 235.3 (212.7, 253.6) |  | Stroke | 41.2 (33.8, 46.8) |
|  | 2 | Ischemic heart disease | 60.1 (51.8, 68.9) |  | Alzheimer's disease and other dementias | 29.0 (7.9, 69.7) |
|  | 3 | Stomach cancer | 55.4 (44.1, 62.4) |  | Ischemic heart disease | 28.3 (22.9, 32.6) |
|  | 4 | Cirrhosis and other chronic liver diseases | 49.8 (41.5, 53.9) |  | Tracheal, bronchus, and lung cancer | 23.9 (19.7, 28.2) |
|  | 5 | Asthma | 38.5 (27.5, 47.2) |  | Self-harm | 18.1 (11.1, 19.7) |
| Republic of Moldova | 1 | Ischemic heart disease | 404.2 (382.5, 417.0) |  | Ischemic heart disease | 230.5 (209.7, 252.5) |
|  | 2 | Stroke | 177.8 (168.6, 185.2) |  | COVID-19 | 133.9 (129.4, 140.3) |
|  | 3 | Cirrhosis and other chronic liver diseases | 69.2 (66.9, 71.7) |  | Stroke | 85.2 (76.5, 94.2) |
|  | 4 | Chronic obstructive pulmonary disease | 45.4 (42.7, 48.2) |  | Cirrhosis and other chronic liver diseases | 48.1 (43.2, 53.4) |
|  | 5 | Tracheal, bronchus, and lung cancer | 30.0 (27.8, 32.1) |  | Hypertensive heart disease | 31.4 (27.8, 35.5) |
| Romania | 1 | Ischemic heart disease | 278.0 (264.2, 287.4) |  | Ischemic heart disease | 154.2 (140.0, 169.1) |
|  | 2 | Stroke | 235.5 (222.6, 245.2) |  | COVID-19 | 141.9 (88.7, 203.6) |
|  | 3 | Chronic obstructive pulmonary disease | 54.8 (51.3, 57.8) |  | Stroke | 128.3 (113.9, 142.7) |
|  | 4 | Lower respiratory infections | 45.6 (43.8, 47.2) |  | Hypertensive heart disease | 36.1 (32.1, 40.6) |
|  | 5 | Hypertensive heart disease | 43.0 (40.2, 45.4) |  | Cirrhosis and other chronic liver diseases | 31.8 (28.1, 35.3) |
| Russian Federation | 1 | Ischemic heart disease | 311.3 (294.2, 318.7) |  | COVID-19 | 216.2 (179.8, 253.8) |
|  | 2 | Stroke | 231.6 (218.0, 237.4) |  | Ischemic heart disease | 212.9 (195.6, 229.0) |
|  | 3 | Tracheal, bronchus, and lung cancer | 35.0 (34.3, 35.6) |  | Stroke | 129.8 (118.7, 139.6) |
|  | 4 | Stomach cancer | 31.5 (30.6, 32.1) |  | Cardiomyopathy and myocarditis | 26.0 (23.6, 28.4) |
|  | 5 | Chronic obstructive pulmonary disease | 29.8 (28.5, 30.4) |  | Cirrhosis and other chronic liver diseases | 23.1 (21.4, 24.7) |
| Rwanda | 1 | Stroke | 297.0 (245.6, 356.8) |  | COVID-19 | 257.8 (212.7, 286.7) |
|  | 2 | Tuberculosis | 249.3 (175.5, 320.9) |  | Stroke | 129.0 (95.1, 164.5) |
|  | 3 | Lower respiratory infections | 197.8 (161.2, 238.2) |  | Lower respiratory infections | 71.5 (52.8, 92.4) |
|  | 4 | Diarrheal diseases | 110.7 (64.4, 172.9) |  | Ischemic heart disease | 65.1 (46.3, 88.1) |
|  | 5 | Malaria | 108.5 (47.5, 226.4) |  | Tuberculosis | 55.1 (36.0, 76.4) |
| Samoa | 1 | Ischemic heart disease | 205.7 (170.8, 240.0) |  | Ischemic heart disease | 226.7 (194.9, 270.9) |
|  | 2 | Stroke | 194.1 (162.6, 221.9) |  | Stroke | 149.8 (125.8, 175.7) |
|  | 3 | Chronic obstructive pulmonary disease | 101.2 (75.6, 135.5) |  | Diabetes mellitus | 92.8 (74.7, 115.4) |
|  | 4 | Diabetes mellitus | 76.2 (64.1, 91.2) |  | Chronic obstructive pulmonary disease | 69.6 (54.9, 86.0) |
|  | 5 | Lower respiratory infections | 65.1 (55.4, 78.4) |  | Lower respiratory infections | 48.6 (40.8, 58.3) |
| Sao Tome and Principe | 1 | Lower respiratory infections | 152.5 (132.4, 171.7) |  | Stroke | 134.2 (115.6, 158.2) |
|  | 2 | Stroke | 137.0 (120.2, 153.9) |  | Ischemic heart disease | 103.5 (89.8, 120.6) |
|  | 3 | Diarrheal diseases | 87.7 (64.2, 111.3) |  | Lower respiratory infections | 99.4 (84.7, 118.6) |
|  | 4 | Ischemic heart disease | 80.5 (70.6, 90.2) |  | COVID-19 | 91.4 (81.2, 100.0) |
|  | 5 | Cirrhosis and other chronic liver diseases | 74.0 (59.0, 89.1) |  | Chronic kidney disease | 72.4 (51.4, 86.9) |
| Saudi Arabia | 1 | Ischemic heart disease | 225.5 (174.3, 279.6) |  | Ischemic heart disease | 185.9 (158.1, 219.1) |
|  | 2 | Stroke | 161.7 (126.5, 200.7) |  | Stroke | 99.5 (83.5, 120.4) |
|  | 3 | Cirrhosis and other chronic liver diseases | 63.3 (37.3, 120.0) |  | Chronic kidney disease | 79.3 (59.8, 95.7) |
|  | 4 | Hypertensive heart disease | 62.6 (40.8, 86.3) |  | COVID-19 | 52.1 (35.2, 71.4) |
|  | 5 | Road injuries | 60.3 (44.8, 76.3) |  | Hypertensive heart disease | 50.4 (28.8, 69.8) |
| Senegal | 1 | Diarrheal diseases | 187.6 (126.0, 265.4) |  | COVID-19 | 229.1 (192.8, 258.6) |
|  | 2 | Stroke | 171.5 (147.4, 198.2) |  | Stroke | 148.8 (119.2, 184.7) |
|  | 3 | Ischemic heart disease | 128.2 (105.2, 151.2) |  | Ischemic heart disease | 121.6 (95.5, 151.3) |
|  | 4 | Lower respiratory infections | 112.5 (97.8, 128.1) |  | Lower respiratory infections | 61.5 (48.4, 76.4) |
|  | 5 | Tuberculosis | 88.6 (75.1, 104.1) |  | Diabetes mellitus | 46.3 (36.1, 58.7) |
| Serbia | 1 | Stroke | 318.0 (288.2, 347.2) |  | Ischemic heart disease | 174.3 (150.3, 200.2) |
|  | 2 | Ischemic heart disease | 309.1 (280.4, 334.4) |  | Stroke | 160.2 (136.1, 186.4) |
|  | 3 | Tracheal, bronchus, and lung cancer | 41.5 (33.2, 51.8) |  | COVID-19 | 117.6 (50.4, 190.8) |
|  | 4 | Hypertensive heart disease | 38.8 (30.2, 50.2) |  | Tracheal, bronchus, and lung cancer | 41.2 (32.4, 50.9) |
|  | 5 | Chronic obstructive pulmonary disease | 33.2 (28.5, 40.2) |  | Hypertensive heart disease | 26.1 (18.9, 32.9) |
| Seychelles | 1 | Ischemic heart disease | 134.1 (123.0, 144.6) |  | COVID-19 | 130.3 (115.0, 146.9) |
|  | 2 | Stroke | 130.4 (119.3, 142.2) |  | Ischemic heart disease | 87.6 (78.8, 98.0) |
|  | 3 | Lower respiratory infections | 85.5 (75.7, 94.4) |  | Stroke | 79.4 (70.3, 89.0) |
|  | 4 | Hypertensive heart disease | 85.3 (74.0, 94.9) |  | Lower respiratory infections | 58.2 (50.3, 66.2) |
|  | 5 | Chronic obstructive pulmonary disease | 35.3 (30.2, 39.7) |  | Hypertensive heart disease | 54.3 (46.8, 64.4) |
| Sierra Leone | 1 | Malaria | 202.4 (73.8, 373.4) |  | Malaria | 169.7 (53.6, 334.2) |
|  | 2 | Lower respiratory infections | 191.4 (156.8, 228.3) |  | Stroke | 155.9 (125.6, 192.8) |
|  | 3 | Diarrheal diseases | 179.8 (106.0, 253.7) |  | Ischemic heart disease | 129.5 (101.8, 159.1) |
|  | 4 | Stroke | 174.8 (146.0, 204.4) |  | COVID-19 | 128.9 (107.4, 153.1) |
|  | 5 | Ischemic heart disease | 128.8 (108.5, 151.7) |  | Lower respiratory infections | 97.2 (76.0, 121.9) |
| Singapore | 1 | Ischemic heart disease | 148.2 (140.1, 153.0) |  | Ischemic heart disease | 46.7 (41.8, 49.9) |
|  | 2 | Stroke | 90.6 (84.5, 94.6) |  | Lower respiratory infections | 38.3 (32.5, 41.7) |
|  | 3 | Lower respiratory infections | 77.6 (70.6, 82.0) |  | Alzheimer's disease and other dementias | 17.9 (4.8, 44.1) |
|  | 4 | Chronic obstructive pulmonary disease | 40.8 (38.2, 42.7) |  | Tracheal, bronchus, and lung cancer | 16.2 (14.5, 18.0) |
|  | 5 | Tracheal, bronchus, and lung cancer | 34.4 (31.9, 37.1) |  | Stroke | 14.2 (12.3, 15.6) |
| Slovakia | 1 | Ischemic heart disease | 327.3 (310.4, 341.4) |  | Ischemic heart disease | 184.8 (160.5, 206.7) |
|  | 2 | Stroke | 144.4 (129.7, 160.1) |  | COVID-19 | 169.2 (117.4, 222.1) |
|  | 3 | Lower respiratory infections | 44.4 (37.9, 49.3) |  | Stroke | 66.0 (55.5, 78.0) |
|  | 4 | Tracheal, bronchus, and lung cancer | 41.6 (35.9, 47.6) |  | Tracheal, bronchus, and lung cancer | 25.7 (20.0, 31.8) |
|  | 5 | Cirrhosis and other chronic liver diseases | 28.2 (25.2, 31.5) |  | Colon and rectum cancer | 24.7 (20.2, 29.5) |
| Slovenia | 1 | Ischemic heart disease | 128.3 (119.0, 135.3) |  | COVID-19 | 65.2 (40.6, 94.4) |
|  | 2 | Stroke | 109.6 (101.0, 116.3) |  | Ischemic heart disease | 45.9 (39.2, 51.6) |
|  | 3 | Cardiomyopathy and myocarditis | 55.8 (50.6, 60.4) |  | Stroke | 33.4 (28.2, 37.6) |
|  | 4 | Tracheal, bronchus, and lung cancer | 35.7 (33.5, 37.9) |  | Tracheal, bronchus, and lung cancer | 27.9 (23.7, 32.0) |
|  | 5 | Cirrhosis and other chronic liver diseases | 27.7 (26.4, 29.0) |  | Alzheimer's disease and other dementias | 20.8 (5.2, 51.9) |
| Solomon Islands | 1 | Ischemic heart disease | 292.8 (239.3, 353.7) |  | Ischemic heart disease | 275.2 (228.5, 339.1) |
|  | 2 | Stroke | 259.6 (208.6, 307.7) |  | Stroke | 227.2 (187.9, 275.9) |
|  | 3 | Lower respiratory infections | 182.4 (134.4, 222.1) |  | Lower respiratory infections | 121.8 (99.8, 145.1) |
|  | 4 | Malaria | 131.0 (14.6, 445.6) |  | Diabetes mellitus | 112.3 (89.7, 140.0) |
|  | 5 | Chronic obstructive pulmonary disease | 87.6 (70.3, 106.1) |  | Chronic obstructive pulmonary disease | 66.5 (54.6, 80.6) |
| Somalia | 1 | Tuberculosis | 296.1 (179.8, 499.4) |  | COVID-19 | 559.0 (406.1, 761.5) |
|  | 2 | Diarrheal diseases | 257.1 (149.9, 404.4) |  | Tuberculosis | 264.8 (158.3, 456.8) |
|  | 3 | Stroke | 217.0 (170.1, 265.6) |  | Stroke | 153.5 (112.7, 200.6) |
|  | 4 | Lower respiratory infections | 192.0 (156.2, 232.9) |  | Diarrheal diseases | 135.9 (71.2, 216.1) |
|  | 5 | Measles | 90.8 (32.1, 166.4) |  | Lower respiratory infections | 125.2 (97.3, 162.2) |
| South Africa | 1 | Lower respiratory infections | 91.0 (82.3, 103.1) |  | COVID-19 | 361.8 (361.2, 362.3) |
|  | 2 | Stroke | 88.3 (71.0, 101.3) |  | HIV/AIDS | 158.3 (151.3, 165.5) |
|  | 3 | Diarrheal diseases | 81.3 (61.6, 118.0) |  | Stroke | 102.8 (92.1, 111.9) |
|  | 4 | Ischemic heart disease | 74.4 (61.3, 84.3) |  | Ischemic heart disease | 78.0 (71.1, 84.7) |
|  | 5 | Tuberculosis | 73.3 (61.4, 91.8) |  | Diabetes mellitus | 77.7 (72.2, 83.3) |
| South Sudan | 1 | Diarrheal diseases | 230.2 (148.3, 342.8) |  | COVID-19 | 264.8 (193.4, 342.6) |
|  | 2 | Stroke | 172.1 (138.2, 212.7) |  | Diarrheal diseases | 166.7 (98.1, 277.5) |
|  | 3 | Lower respiratory infections | 163.8 (137.0, 198.6) |  | Stroke | 129.1 (96.5, 165.0) |
|  | 4 | Tuberculosis | 157.5 (104.0, 248.1) |  | Lower respiratory infections | 116.7 (91.5, 150.3) |
|  | 5 | Protein-energy malnutrition | 83.2 (63.6, 110.1) |  | Tuberculosis | 114.0 (77.7, 181.2) |
| Sri Lanka | 1 | Stroke | 176.9 (161.1, 191.9) |  | Stroke | 110.7 (78.9, 143.3) |
|  | 2 | Ischemic heart disease | 138.4 (125.0, 151.5) |  | Ischemic heart disease | 94.6 (64.8, 125.4) |
|  | 3 | Chronic obstructive pulmonary disease | 76.9 (66.0, 85.8) |  | COVID-19 | 68.2 (59.6, 124.0) |
|  | 4 | Self-harm | 40.0 (35.6, 43.8) |  | Diabetes mellitus | 47.7 (33.6, 63.0) |
|  | 5 | Diabetes mellitus | 39.0 (33.6, 43.9) |  | Chronic obstructive pulmonary disease | 46.2 (31.8, 60.7) |
| Sudan | 1 | Ischemic heart disease | 339.6 (276.2, 415.9) |  | Ischemic heart disease | 255.9 (202.4, 330.7) |
|  | 2 | Stroke | 199.7 (158.2, 244.8) |  | COVID-19 | 187.3 (110.1, 278.3) |
|  | 3 | Lower respiratory infections | 79.0 (64.4, 98.1) |  | Stroke | 119.9 (91.7, 152.9) |
|  | 4 | Neonatal disorders | 78.9 (65.3, 96.3) |  | Hypertensive heart disease | 50.7 (31.3, 72.9) |
|  | 5 | Hypertensive heart disease | 65.3 (40.3, 89.7) |  | Chronic kidney disease | 32.1 (24.3, 43.8) |
| Suriname | 1 | Ischemic heart disease | 173.0 (158.6, 185.2) |  | COVID-19 | 180.9 (177.5, 234.2) |
|  | 2 | Stroke | 130.2 (117.6, 141.1) |  | Stroke | 92.2 (72.2, 113.6) |
|  | 3 | Neonatal disorders | 49.4 (43.4, 56.2) |  | Ischemic heart disease | 90.9 (69.9, 113.4) |
|  | 4 | Diabetes mellitus | 40.5 (36.8, 44.3) |  | Diabetes mellitus | 42.7 (33.3, 53.2) |
|  | 5 | Lower respiratory infections | 39.6 (35.7, 43.6) |  | Chronic kidney disease | 41.4 (31.0, 53.6) |
| Syrian Arab Republic | 1 | Ischemic heart disease | 370.8 (309.8, 437.6) |  | Ischemic heart disease | 353.0 (281.6, 432.0) |
|  | 2 | Stroke | 150.2 (123.3, 181.8) |  | COVID-19 | 107.7 (71.1, 143.7) |
|  | 3 | Hypertensive heart disease | 49.7 (38.0, 61.6) |  | Stroke | 106.3 (84.0, 130.9) |
|  | 4 | Chronic kidney disease | 39.1 (30.4, 52.8) |  | Chronic kidney disease | 47.7 (33.6, 62.8) |
|  | 5 | Neonatal disorders | 35.1 (29.9, 41.7) |  | Hypertensive heart disease | 38.9 (29.6, 51.7) |
| Tajikistan | 1 | Ischemic heart disease | 304.8 (266.0, 336.2) |  | Ischemic heart disease | 244.9 (206.6, 281.6) |
|  | 2 | Stroke | 160.7 (139.0, 182.0) |  | COVID-19 | 219.0 (161.2, 264.3) |
|  | 3 | Lower respiratory infections | 96.2 (86.2, 107.0) |  | Stroke | 130.1 (105.4, 154.8) |
|  | 4 | Chronic obstructive pulmonary disease | 48.4 (39.1, 60.0) |  | Lower respiratory infections | 34.8 (27.8, 43.4) |
|  | 5 | Hypertensive heart disease | 45.1 (29.2, 70.5) |  | Hypertensive heart disease | 33.5 (23.5, 47.4) |
| Thailand | 1 | Stroke | 121.6 (104.5, 138.2) |  | Stroke | 65.6 (51.0, 80.8) |
|  | 2 | Ischemic heart disease | 84.4 (71.2, 97.8) |  | Ischemic heart disease | 47.1 (36.8, 58.6) |
|  | 3 | Chronic obstructive pulmonary disease | 55.1 (45.5, 64.1) |  | COVID-19 | 38.7 (20.2, 76.2) |
|  | 4 | Tuberculosis | 44.0 (34.5, 53.9) |  | Lower respiratory infections | 35.5 (26.5, 44.6) |
|  | 5 | Diarrheal diseases | 39.3 (11.8, 112.6) |  | Chronic kidney disease | 29.9 (22.8, 37.1) |
| Timor-Leste | 1 | Stroke | 189.3 (154.6, 227.6) |  | Stroke | 175.0 (133.8, 219.2) |
|  | 2 | Tuberculosis | 156.3 (102.9, 221.5) |  | Ischemic heart disease | 156.9 (121.1, 196.0) |
|  | 3 | Lower respiratory infections | 134.8 (115.0, 156.9) |  | COVID-19 | 99.5 (34.8, 195.7) |
|  | 4 | Diarrheal diseases | 134.7 (80.7, 239.2) |  | Tuberculosis | 71.3 (49.8, 108.1) |
|  | 5 | Ischemic heart disease | 122.8 (98.1, 150.9) |  | Lower respiratory infections | 64.8 (51.0, 79.3) |
| Togo | 1 | Stroke | 175.2 (151.4, 201.9) |  | COVID-19 | 176.9 (130.7, 205.4) |
|  | 2 | Diarrheal diseases | 169.1 (105.6, 234.9) |  | Stroke | 165.6 (130.8, 204.9) |
|  | 3 | Lower respiratory infections | 147.0 (125.4, 173.0) |  | Ischemic heart disease | 117.0 (89.5, 149.2) |
|  | 4 | Ischemic heart disease | 114.2 (95.4, 136.0) |  | Lower respiratory infections | 108.1 (86.0, 132.6) |
|  | 5 | Malaria | 108.8 (49.9, 195.4) |  | Malaria | 88.6 (29.9, 179.0) |
| Tonga | 1 | Ischemic heart disease | 138.7 (116.3, 161.3) |  | Ischemic heart disease | 139.1 (113.6, 166.6) |
|  | 2 | Stroke | 92.4 (75.3, 108.2) |  | Diabetes mellitus | 111.7 (90.0, 138.7) |
|  | 3 | Diabetes mellitus | 91.4 (76.5, 106.3) |  | Stroke | 78.3 (63.6, 94.5) |
|  | 4 | Chronic obstructive pulmonary disease | 65.2 (52.9, 76.2) |  | Chronic obstructive pulmonary disease | 49.0 (39.8, 58.7) |
|  | 5 | Lower respiratory infections | 60.4 (51.1, 69.4) |  | Lower respiratory infections | 48.7 (39.1, 59.2) |
| Trinidad and Tobago | 1 | Ischemic heart disease | 234.7 (225.5, 241.9) |  | COVID-19 | 175.4 (146.8, 277.8) |
|  | 2 | Stroke | 138.7 (131.2, 144.5) |  | Ischemic heart disease | 106.0 (83.2, 131.9) |
|  | 3 | Diabetes mellitus | 132.3 (126.4, 138.5) |  | Diabetes mellitus | 97.9 (75.9, 121.4) |
|  | 4 | Lower respiratory infections | 44.9 (42.6, 47.1) |  | Stroke | 69.5 (54.8, 86.0) |
|  | 5 | Hypertensive heart disease | 42.5 (39.9, 44.5) |  | Chronic kidney disease | 40.2 (30.1, 51.3) |
| Tunisia | 1 | Ischemic heart disease | 218.3 (180.4, 251.4) |  | COVID-19 | 208.3 (169.2, 253.1) |
|  | 2 | Stroke | 122.6 (99.2, 150.5) |  | Ischemic heart disease | 163.6 (121.8, 215.7) |
|  | 3 | Hypertensive heart disease | 44.8 (31.7, 58.4) |  | Stroke | 81.2 (56.2, 110.3) |
|  | 4 | Neonatal disorders | 44.3 (36.7, 52.4) |  | Hypertensive heart disease | 38.0 (20.7, 63.9) |
|  | 5 | Lower respiratory infections | 34.0 (27.5, 43.4) |  | Chronic kidney disease | 32.1 (23.1, 43.9) |
| Turkey | 1 | Ischemic heart disease | 204.6 (180.7, 228.2) |  | Ischemic heart disease | 133.4 (109.9, 155.9) |
|  | 2 | Stroke | 144.7 (121.4, 166.8) |  | COVID-19 | 126.8 (96.7, 148.8) |
|  | 3 | Chronic obstructive pulmonary disease | 57.5 (45.0, 67.3) |  | Stroke | 72.1 (59.5, 85.7) |
|  | 4 | Neonatal disorders | 52.5 (45.5, 60.8) |  | Chronic obstructive pulmonary disease | 40.8 (33.9, 48.5) |
|  | 5 | Tracheal, bronchus, and lung cancer | 50.0 (39.1, 62.2) |  | Tracheal, bronchus, and lung cancer | 34.4 (27.0, 42.5) |
| Uganda | 1 | HIV/AIDS | 497.3 (314.9, 696.0) |  | COVID-19 | 311.9 (197.6, 382.4) |
|  | 2 | Malaria | 160.8 (95.4, 273.9) |  | Stroke | 102.2 (80.6, 129.1) |
|  | 3 | Stroke | 159.8 (128.7, 194.3) |  | Malaria | 93.0 (28.0, 182.3) |
|  | 4 | Diarrheal diseases | 144.2 (67.2, 279.5) |  | HIV/AIDS | 87.1 (72.7, 110.1) |
|  | 5 | Tuberculosis | 134.0 (91.6, 221.4) |  | Lower respiratory infections | 72.6 (59.2, 91.4) |
| Ukraine | 1 | Ischemic heart disease | 338.1 (318.1, 349.5) |  | Ischemic heart disease | 373.5 (291.6, 459.1) |
|  | 2 | Stroke | 189.4 (178.5, 196.4) |  | COVID-19 | 134.0 (110.8, 167.4) |
|  | 3 | Chronic obstructive pulmonary disease | 49.1 (46.2, 51.4) |  | Stroke | 104.2 (81.5, 129.1) |
|  | 4 | Tracheal, bronchus, and lung cancer | 37.0 (34.7, 39.4) |  | Cirrhosis and other chronic liver diseases | 23.4 (16.8, 30.8) |
|  | 5 | Stomach cancer | 26.3 (24.7, 27.8) |  | Cardiomyopathy and myocarditis | 22.0 (16.3, 28.0) |
| United Arab Emirates | 1 | Ischemic heart disease | 266.9 (214.6, 326.8) |  | Ischemic heart disease | 167.7 (136.6, 196.3) |
|  | 2 | Stroke | 136.3 (110.2, 164.7) |  | Stroke | 88.8 (72.6, 105.9) |
|  | 3 | Lower respiratory infections | 49.1 (40.1, 60.2) |  | COVID-19 | 70.1 (34.7, 94.8) |
|  | 4 | Road injuries | 48.5 (38.8, 61.7) |  | Diabetes mellitus | 43.1 (33.5, 52.2) |
|  | 5 | Diabetes mellitus | 42.3 (32.5, 55.9) |  | Chronic kidney disease | 37.5 (20.9, 49.2) |
| United Republic of Tanzania | 1 | Lower respiratory infections | 172.8 (148.2, 195.8) |  | COVID-19 | 278.8 (249.6, 299.3) |
|  | 2 | Diarrheal diseases | 157.4 (98.2, 214.2) |  | Stroke | 109.7 (85.9, 139.0) |
|  | 3 | Stroke | 135.5 (111.3, 162.0) |  | Ischemic heart disease | 96.3 (72.0, 123.4) |
|  | 4 | Tuberculosis | 132.1 (96.6, 185.6) |  | HIV/AIDS | 78.5 (63.6, 96.5) |
|  | 5 | HIV/AIDS | 125.9 (79.4, 197.1) |  | Lower respiratory infections | 73.5 (61.8, 89.9) |
| Uruguay | 1 | Ischemic heart disease | 152.3 (143.3, 157.8) |  | COVID-19 | 99.8 (99.8, 99.8) |
|  | 2 | Stroke | 118.6 (110.9, 123.9) |  | Ischemic heart disease | 60.5 (55.5, 63.8) |
|  | 3 | Tracheal, bronchus, and lung cancer | 41.3 (38.5, 43.8) |  | Stroke | 53.7 (48.6, 57.4) |
|  | 4 | Chronic obstructive pulmonary disease | 30.3 (28.6, 31.6) |  | Tracheal, bronchus, and lung cancer | 33.0 (30.3, 35.5) |
|  | 5 | Colon and rectum cancer | 26.1 (23.7, 28.4) |  | Chronic obstructive pulmonary disease | 32.4 (29.7, 34.5) |
| Uzbekistan | 1 | Ischemic heart disease | 318.6 (295.1, 333.6) |  | Ischemic heart disease | 339.5 (295.6, 382.0) |
|  | 2 | Stroke | 133.1 (124.8, 139.6) |  | Stroke | 105.4 (92.2, 121.5) |
|  | 3 | Lower respiratory infections | 62.2 (58.2, 66.8) |  | COVID-19 | 60.7 (9.9, 98.4) |
|  | 4 | Cirrhosis and other chronic liver diseases | 34.7 (33.1, 36.4) |  | Cirrhosis and other chronic liver diseases | 28.2 (24.1, 33.1) |
|  | 5 | Chronic obstructive pulmonary disease | 25.1 (21.6, 29.8) |  | Diabetes mellitus | 25.1 (21.2, 29.2) |
| Vanuatu | 1 | Ischemic heart disease | 312.4 (259.6, 376.1) |  | Ischemic heart disease | 308.4 (260.9, 357.1) |
|  | 2 | Stroke | 227.5 (188.5, 278.7) |  | Stroke | 181.6 (147.2, 217.4) |
|  | 3 | Chronic obstructive pulmonary disease | 115.5 (74.1, 152.7) |  | COVID-19 | 105.9 (24.4, 214.3) |
|  | 4 | Lower respiratory infections | 73.6 (60.0, 88.9) |  | Diabetes mellitus | 89.9 (73.4, 112.3) |
|  | 5 | Diabetes mellitus | 72.0 (55.6, 102.8) |  | Chronic obstructive pulmonary disease | 83.4 (54.1, 112.1) |
| Venezuela  (Bolivarian Republic of) | 1 | Ischemic heart disease | 169.6 (157.9, 175.9) |  | COVID-19 | 213.3 (200.3, 230.7) |
|  | 2 | Stroke | 76.1 (71.6, 79.5) |  | Ischemic heart disease | 141.4 (112.2, 177.1) |
|  | 3 | Diabetes mellitus | 34.4 (32.2, 36.2) |  | Stroke | 57.6 (44.0, 72.7) |
|  | 4 | Lower respiratory infections | 33.8 (31.2, 35.2) |  | Interpersonal violence | 48.6 (38.2, 59.3) |
|  | 5 | Hypertensive heart disease | 31.4 (29.1, 33.2) |  | Diabetes mellitus | 43.7 (34.0, 55.6) |
| Viet Nam | 1 | Stroke | 229.3 (186.5, 285.6) |  | Stroke | 196.6 (164.2, 226.8) |
|  | 2 | Tuberculosis | 81.0 (59.1, 105.6) |  | Ischemic heart disease | 77.0 (63.5, 90.7) |
|  | 3 | Ischemic heart disease | 66.4 (52.9, 83.9) |  | Chronic obstructive pulmonary disease | 44.0 (35.1, 54.2) |
|  | 4 | Chronic obstructive pulmonary disease | 54.3 (40.0, 67.9) |  | COVID-19 | 40.8 (36.0, 65.5) |
|  | 5 | Lower respiratory infections | 45.0 (38.0, 56.4) |  | Diabetes mellitus | 36.0 (29.3, 43.2) |
| Yemen | 1 | Ischemic heart disease | 320.4 (252.1, 407.1) |  | Ischemic heart disease | 263.5 (201.8, 344.2) |
|  | 2 | Stroke | 220.6 (166.3, 284.0) |  | COVID-19 | 203.8 (88.6, 318.0) |
|  | 3 | Hypertensive heart disease | 80.8 (47.4, 114.2) |  | Stroke | 165.1 (124.3, 222.4) |
|  | 4 | Neonatal disorders | 72.7 (60.3, 87.9) |  | Hypertensive heart disease | 66.3 (40.4, 108.1) |
|  | 5 | Lower respiratory infections | 70.4 (56.5, 87.6) |  | Conflict and terrorism | 50.1 (45.5, 55.1) |
| Zambia | 1 | HIV/AIDS | 193.4 (113.0, 335.0) |  | COVID-19 | 577.2 (424.6, 735.2) |
|  | 2 | Stroke | 191.2 (161.5, 225.6) |  | Stroke | 169.0 (131.2, 214.2) |
|  | 3 | Diarrheal diseases | 184.9 (119.2, 260.9) |  | HIV/AIDS | 148.3 (120.8, 184.7) |
|  | 4 | Tuberculosis | 184.6 (137.6, 226.1) |  | Ischemic heart disease | 92.7 (71.7, 117.3) |
|  | 5 | Lower respiratory infections | 159.1 (135.9, 182.1) |  | Lower respiratory infections | 79.7 (64.5, 96.6) |
| Zimbabwe | 1 | HIV/AIDS | 232.4 (146.7, 361.0) |  | COVID-19 | 722.3 (559.9, 852.7) |
|  | 2 | Lower respiratory infections | 128.4 (108.8, 147.6) |  | HIV/AIDS | 172.6 (142.0, 215.3) |
|  | 3 | Stroke | 115.2 (94.7, 135.1) |  | Stroke | 160.2 (131.3, 191.7) |
|  | 4 | Tuberculosis | 97.4 (78.6, 116.6) |  | Tuberculosis | 131.7 (84.0, 171.1) |
|  | 5 | Ischemic heart disease | 83.5 (68.2, 98.9) |  | Lower respiratory infections | 129.3 (100.4, 158.1) |

| Supplementary Table 8: Top five causes of age-standardized DALY rate (/100,000 persons) across 149 BRI countries in 1990 and in 2021 | | | | | | |
| --- | --- | --- | --- | --- | --- | --- |
| Country | Rank | 1990 | |  | 2021 | |
|  |  | Cause | DALY rate (95% UI) |  | Cause | DALY rate (95% UI) |
| Afghanistan | 1 | Ischemic heart disease | 8374.2 (6234.3, 10414.2) |  | COVID-19 | 9508.7 (7587.7, 11151.7) |
|  | 2 | Lower respiratory infections | 7810.1 (6243.0, 9803.5) |  | Conflict and terrorism | 8189.9 (7141.3, 9768.7) |
|  | 3 | Stroke | 5857.3 (4360.4, 7301.0) |  | Ischemic heart disease | 6178.3 (4747.6, 7813.3) |
|  | 4 | Tuberculosis | 5517.2 (4072.2, 9226.7) |  | Stroke | 3982.5 (3016.5, 4998.5) |
|  | 5 | Neonatal disorders | 5290.2 (4474.3, 6341.7) |  | Neonatal disorders | 2706.5 (2189.0, 3256.9) |
| Albania | 1 | Stroke | 3539.3 (3161.9, 3902.9) |  | COVID-19 | 2952.7 (1566.0, 4353.1) |
|  | 2 | Ischemic heart disease | 3173.4 (2843.6, 3464.0) |  | Ischemic heart disease | 2558.3 (2184.9, 2998.3) |
|  | 3 | Lower respiratory infections | 3126.0 (2689.3, 3564.9) |  | Stroke | 2316.2 (1899.3, 2764.3) |
|  | 4 | Neonatal disorders | 2327.9 (2020.8, 2638.9) |  | Low back pain | 1465.1 (1042.2, 1978.5) |
|  | 5 | Low back pain | 1450.6 (1037.3, 1949.4) |  | Neonatal disorders | 1463.6 (1199.3, 1776.4) |
| Algeria | 1 | Ischemic heart disease | 5491.8 (4703.6, 6400.4) |  | COVID-19 | 4061.7 (3202.1, 4858.8) |
|  | 2 | Neonatal disorders | 4429.4 (3817.2, 5270.8) |  | Ischemic heart disease | 3452.0 (2807.0, 4159.9) |
|  | 3 | Congenital birth defects | 3119.5 (1628.7, 3959.4) |  | Stroke | 1837.1 (1465.9, 2260.0) |
|  | 4 | Stroke | 3095.1 (2565.4, 3733.2) |  | Neonatal disorders | 1690.0 (1272.5, 2155.0) |
|  | 5 | Road injuries | 2986.4 (2623.4, 3334.6) |  | Road injuries | 1279.6 (1113.2, 1457.9) |
| Angola | 1 | Diarrheal diseases | 13222.6(9653.4, 16824.1) |  | COVID-19 | 6957.7 (5297.2, 8819.7) |
|  | 2 | Lower respiratory infections | 8313.1 (6748.9, 10040.6) |  | HIV/AIDS | 3925.6 (2679.5, 5995.1) |
|  | 3 | Tuberculosis | 8223.2 (5605.5, 10730.3) |  | Neonatal disorders | 3607.7 (3020.6, 4212.0) |
|  | 4 | Neonatal disorders | 7398.6 (6149.8, 8483.9) |  | Malaria | 3417.1 (1399.9, 7141.2) |
|  | 5 | Protein-energy malnutrition | 4706.0 (3412.0, 6853.0) |  | Stroke | 2912.6 (2332.7, 3559.6) |
| Antigua and Barbuda | 1 | Ischemic heart disease | 2532.2 (2376.3, 2673.1) |  | COVID-19 | 2621.2 (2571.7, 2724.4) |
|  | 2 | Stroke | 2502.9 (2337.3, 2656.7) |  | Diabetes mellitus | 2188.1 (1851.1, 2611.8) |
|  | 3 | Diabetes mellitus | 2081.4 (1886.7, 2340.8) |  | Stroke | 1372.6 (1285.9, 1469.9) |
|  | 4 | Neonatal disorders | 1482.5 (1261.3, 1735.3) |  | Ischemic heart disease | 1306.3 (1226.1, 1441.3) |
|  | 5 | Chronic kidney disease | 807.8 (751.9, 862.9) |  | Chronic kidney disease | 1116.5 (1028.1, 1190.1) |
| Argentina | 1 | Ischemic heart disease | 3045.5 (2935.2, 3135.3) |  | COVID-19 | 2889.7 (2814.9, 3039.7) |
|  | 2 | Neonatal disorders | 2756.9 (2654.9, 2879.6) |  | Ischemic heart disease | 1179.9 (1113.6, 1234.1) |
|  | 3 | Stroke | 2510.9 (2396.0, 2622.1) |  | Low back pain | 1086.5 (770.8, 1460.1) |
|  | 4 | Low back pain | 1100.4 (782.7, 1475.9) |  | Neonatal disorders | 1058.3 (871.4, 1269.2) |
|  | 5 | Congenital birth defects | 1027.1 (975.1, 1086.3) |  | Lower respiratory infections | 972.6 (897.7, 1038.5) |
| Armenia | 1 | Ischemic heart disease | 5669.8 (5378.0, 5942.5) |  | Ischemic heart disease | 3788.5 (3403.8, 4233.6) |
|  | 2 | Neonatal disorders | 2835.8 (2545.8, 3193.2) |  | COVID-19 | 3028.5 (2795.6, 3519.4) |
|  | 3 | Lower respiratory infections | 2516.3 (2267.9, 2837.0) |  | Stroke | 1310.1 (1180.0, 1461.3) |
|  | 4 | Stroke | 2396.7 (2260.3, 2508.1) |  | Neonatal disorders | 1126.4 (949.5, 1322.4) |
|  | 5 | Congenital birth defects | 1226.2 (1081.6, 1394.7) |  | Low back pain | 1113.3 (803.1, 1493.4) |
| Austria | 1 | Ischemic heart disease | 2995.0 (2831.2, 3084.8) |  | Ischemic heart disease | 1121.6 (1017.8, 1189.7) |
|  | 2 | Stroke | 1548.7 (1452.9, 1625.3) |  | COVID-19 | 1117.4 (1031.7, 1249.6) |
|  | 3 | Road injuries | 1138.1 (1065.7, 1219.6) |  | Low back pain | 909.1 (654.7, 1219.5) |
|  | 4 | Low back pain | 977.3 (711.5, 1301.8) |  | Anxiety disorders | 846.4 (521.8, 1246.7) |
|  | 5 | Falls | 963.4 (752.5, 1213.2) |  | Falls | 750.3 (566.8, 966.4) |
| Azerbaijan | 1 | Lower respiratory infections | 8101.5 (6988.5, 9248.2) |  | Ischemic heart disease | 5496.3 (4818.7, 6182.3) |
|  | 2 | Ischemic heart disease | 7154.0 (6698.2, 7635.3) |  | COVID-19 | 5249.4 (4263.9, 6310.9) |
|  | 3 | Neonatal disorders | 2868.0 (2451.7, 3327.4) |  | Neonatal disorders | 2385.3 (1959.1, 2872.9) |
|  | 4 | Stroke | 2770.9 (2429.2, 3153.3) |  | Stroke | 2011.5 (1671.6, 2369.7) |
|  | 5 | Diarrheal diseases | 1597.9 (1242.6, 2042.0) |  | Lower respiratory infections | 1627.1 (1328.3, 1976.5) |
| Bahrain | 1 | Ischemic heart disease | 7687.4 (7231.5, 8166.7) |  | COVID-19 | 4138.3 (3356.3, 4820.3) |
|  | 2 | Stroke | 2941.2 (2659.7, 3206.0) |  | Diabetes mellitus | 3437.4 (2914.6, 4060.5) |
|  | 3 | Diabetes mellitus | 2650.6 (2336.8, 2984.7) |  | Ischemic heart disease | 2796.6 (2418.6, 3179.5) |
|  | 4 | Neonatal disorders | 1974.2 (1806.5, 2163.9) |  | Stroke | 1384.2 (1212.7, 1582.0) |
|  | 5 | Congenital birth defects | 1438.7 (1186.4, 1615.6) |  | Depressive disorders | 974.2 (630.4, 1400.6) |
| Bangladesh | 1 | Neonatal disorders | 8189.8 (7274.2, 9420.9) |  | Neonatal disorders | 3447.3 (2714.5, 4261.3) |
|  | 2 | Lower respiratory infections | 5548.1 (4860.3, 6263.4) |  | Stroke | 2937.8 (2411.7, 3565.4) |
|  | 3 | Tuberculosis | 4765.3 (3802.2, 5594.0) |  | COVID-19 | 2604.8 (2171.8, 3207.8) |
|  | 4 | Stroke | 4576.1 (3986.5, 5184.5) |  | Ischemic heart disease | 2366.9 (1856.4, 2929.7) |
|  | 5 | Diarrheal diseases | 4358.7 (3234.2, 5888.9) |  | Chronic obstructive pulmonary disease | 1301.7 (1050.5, 1612.2) |
| Barbados | 1 | Ischemic heart disease | 2226.4 (2109.7, 2327.0) |  | Diabetes mellitus | 2135.8 (1772.3, 2596.8) |
|  | 2 | Diabetes mellitus | 2211.2 (2004.5, 2473.8) |  | COVID-19 | 1478.0 (1443.7, 1553.6) |
|  | 3 | Stroke | 2169.3 (2057.1, 2274.9) |  | Neonatal disorders | 1453.0 (1096.4, 1894.4) |
|  | 4 | Neonatal disorders | 2123.8 (1829.0, 2424.0) |  | Stroke | 1335.8 (1096.1, 1619.1) |
|  | 5 | Road injuries | 741.1 (696.2, 787.5) |  | Ischemic heart disease | 1150.8 (936.2, 1387.6) |
| Belarus | 1 | Ischemic heart disease | 6062.1 (5746.8, 6279.0) |  | Ischemic heart disease | 6244.3 (5262.8, 7270.2) |
|  | 2 | Stroke | 2843.9 (2667.2, 3018.6) |  | COVID-19 | 4664.0 (3584.4, 6042.1) |
|  | 3 | Road injuries | 1576.6 (1500.1, 1661.6) |  | Stroke | 2037.4 (1709.9, 2380.8) |
|  | 4 | Neonatal disorders | 1361.8 (1158.2, 1585.3) |  | Low back pain | 1214.8 (876.3, 1627.4) |
|  | 5 | Congenital birth defects | 1234.9 (1059.3, 1420.3) |  | Alcohol use disorders | 992.5 (839.3, 1182.3) |
| Benin | 1 | Diarrheal diseases | 7721.7 (5462.1, 10287.9) |  | Malaria | 6891.5 (3262.0, 12411.9) |
|  | 2 | Lower respiratory infections | 7516.3 (6203.6, 9006.5) |  | Neonatal disorders | 5149.5 (4379.5, 6026.0) |
|  | 3 | Neonatal disorders | 7300.6 (6665.7, 7940.1) |  | COVID-19 | 4509.8 (4035.9, 5018.0) |
|  | 4 | Malaria | 5708.9 (3140.4, 8812.5) |  | Stroke | 3059.5 (2575.2, 3612.9) |
|  | 5 | Measles | 5039.9 (1880.9, 9711.7) |  | Lower respiratory infections | 2800.6 (2136.5, 3638.0) |
| Bolivia  (Plurinational State of) | 1 | Lower respiratory infections | 7561.0 (5976.4, 9264.2) |  | COVID-19 | 11240.4 (9675.6, 12454.2) |
|  | 2 | Neonatal disorders | 3926.8 (3418.5, 4726.9) |  | Neonatal disorders | 1758.7 (1462.9, 2113.4) |
|  | 3 | Tuberculosis | 3697.8 (2729.6, 4678.6) |  | Ischemic heart disease | 1602.4 (1154.2, 2291.2) |
|  | 4 | Stroke | 2977.1 (2342.1, 3948.5) |  | Lower respiratory infections | 1556.7 (1242.8, 1947.0) |
|  | 5 | Ischemic heart disease | 2859.7 (2236.0, 3774.8) |  | Diabetes mellitus | 1458.4 (1183.8, 1819.3) |
| Bosnia and Herzegovina | 1 | Ischemic heart disease | 3942.9 (3620.8, 4253.7) |  | COVID-19 | 3823.5 (3590.1, 4311.5) |
|  | 2 | Stroke | 3189.0 (2887.8, 3502.6) |  | Ischemic heart disease | 2450.2 (2020.0, 2859.4) |
|  | 3 | Neonatal disorders | 2445.0 (2236.2, 2641.7) |  | Stroke | 2022.8 (1641.7, 2363.5) |
|  | 4 | Low back pain | 1333.6 (956.3, 1782.2) |  | Low back pain | 1372.4 (984.2, 1838.2) |
|  | 5 | Tracheal, bronchus, and lung cancer | 959.0 (847.3, 1077.7) |  | Diabetes mellitus | 1232.2 (990.2, 1515.4) |
| Botswana | 1 | HIV/AIDS | 5372.0 (3597.4, 8879.7) |  | COVID-19 | 13474.4 (9824.6, 16461.7) |
|  | 2 | Diarrheal diseases | 5039.5 (3606.2, 6863.3) |  | HIV/AIDS | 11341.1 (9487.0, 13750.0) |
|  | 3 | Tuberculosis | 4974.1 (3150.3, 7186.7) |  | Neonatal disorders | 3608.9 (2866.7, 4591.0) |
|  | 4 | Neonatal disorders | 4325.6 (3689.7, 5037.0) |  | Stroke | 2206.1 (1787.9, 2790.6) |
|  | 5 | Lower respiratory infections | 4224.3 (3228.1, 5404.8) |  | Lower respiratory infections | 2139.1 (1613.5, 2736.3) |
| Brunei Darussalam | 1 | Stroke | 3211.7 (2820.0, 3631.2) |  | Diabetes mellitus | 2154.8 (1801.3, 2611.7) |
|  | 2 | Ischemic heart disease | 3122.1 (2784.5, 3516.9) |  | Ischemic heart disease | 1726.3 (1525.6, 1929.9) |
|  | 3 | Diabetes mellitus | 2318.7 (2034.9, 2639.7) |  | Stroke | 1509.0 (1324.4, 1727.1) |
|  | 4 | Road injuries | 1828.8 (1617.1, 2042.0) |  | Neonatal disorders | 981.7 (813.1, 1176.8) |
|  | 5 | Chronic obstructive pulmonary disease | 1141.0 (985.2, 1313.3) |  | Low back pain | 972.2 (690.6, 1302.4) |
| Bulgaria | 1 | Ischemic heart disease | 6709.6 (6446.8, 6955.9) |  | COVID-19 | 6154.7 (4259.6, 8135.2) |
|  | 2 | Stroke | 5517.0 (5309.3, 5721.0) |  | Ischemic heart disease | 3852.3 (3333.9, 4409.7) |
|  | 3 | Lower respiratory infections | 1682.2 (1611.0, 1757.8) |  | Stroke | 3430.2 (3007.9, 3916.1) |
|  | 4 | Low back pain | 1415.6 (1020.1, 1894.1) |  | Hypertensive heart disease | 1739.3 (1494.0, 1984.7) |
|  | 5 | Neonatal disorders | 1272.3 (1152.5, 1417.5) |  | Low back pain | 1385.0 (989.3, 1861.5) |
| Burkina Faso | 1 | Malaria | 11157.1(6050.2, 19376.1) |  | Malaria | 8938.2 (4195.3, 15779.3) |
|  | 2 | Diarrheal diseases | 9714.3 (6713.9, 12707.7) |  | COVID-19 | 5181.3 (4180.9, 5954.3) |
|  | 3 | Lower respiratory infections | 9170.5 (7301.6, 10857.8) |  | Neonatal disorders | 4236.9 (3469.5, 5058.5) |
|  | 4 | HIV/AIDS | 7270.8 (4737.0, 10811.8) |  | Lower respiratory infections | 3787.6 (2965.5, 4542.7) |
|  | 5 | Neonatal disorders | 5341.3 (4716.6, 6006.3) |  | Diarrheal diseases | 2778.6 (1987.1, 3699.4) |
| Burundi | 1 | Malaria | 12277.7 (6260.8, 19493.4) |  | Malaria | 5978.6 (2755.3, 11435.0) |
|  | 2 | Tuberculosis | 9962.9 (7892.5, 12365.5) |  | COVID-19 | 4165.4 (3758.1, 4531.1) |
|  | 3 | Lower respiratory infections | 7436.1 (5732.3, 8975.3) |  | Neonatal disorders | 3978.1 (3095.0, 5061.1) |
|  | 4 | Diarrheal diseases | 6348.2 (4228.3, 8531.9) |  | Tuberculosis | 3731.5 (2775.3, 4840.2) |
|  | 5 | Stroke | 6068.0 (4613.9, 7595.2) |  | Stroke | 3067.9 (2470.0, 3802.4) |
| Cabo Verde | 1 | Diarrheal diseases | 3501.4 (2617.4, 4482.6) |  | Ischemic heart disease | 2236.6 (1830.1, 2642.4) |
|  | 2 | Neonatal disorders | 3299.5 (2921.2, 3754.4) |  | Stroke | 2155.9 (1772.5, 2562.6) |
|  | 3 | Stroke | 2311.7 (1958.6, 2647.7) |  | Neonatal disorders | 1739.5 (1337.6, 2253.9) |
|  | 4 | Measles | 2270.6 (996.6, 3906.1) |  | COVID-19 | 1474.3 (1289.7, 1874.3) |
|  | 5 | Lower respiratory infections | 2148.7 (1825.1, 2562.4) |  | Lower respiratory infections | 1353.7 (1088.2, 1626.6) |
| Cambodia | 1 | Lower respiratory infections | 11032.7 (9597.8, 12962.3) |  | Stroke | 3709.4 (2911.2, 4460.1) |
|  | 2 | Tuberculosis | 6219.4 (4312.5, 7796.3) |  | Lower respiratory infections | 2705.2 (2131.2, 3362.4) |
|  | 3 | Stroke | 5286.3 (4497.8, 6119.0) |  | Neonatal disorders | 2370.7 (1961.3, 2867.9) |
|  | 4 | Neonatal disorders | 4363.1 (3588.4, 5186.8) |  | Ischemic heart disease | 2251.2 (1767.8, 2790.1) |
|  | 5 | Diarrheal diseases | 3960.4 (2913.8, 5428.9) |  | COVID-19 | 2015.2 (737.0, 3855.4) |
| Cameroon | 1 | Malaria | 6549.1 (3522.3, 10677.3) |  | COVID-19 | 6862.5 (5948.8, 7779.2) |
|  | 2 | Lower respiratory infections | 5886.1 (4710.5, 7437.6) |  | Malaria | 5668.1 (2298.2, 11313.0) |
|  | 3 | Neonatal disorders | 5827.8 (5133.8, 6551.9) |  | HIV/AIDS | 4024.3 (3442.9, 4748.9) |
|  | 4 | Diarrheal diseases | 5124.0 (3477.6, 7000.1) |  | Neonatal disorders | 3942.7 (3313.6, 4690.0) |
|  | 5 | Measles | 3751.1 (1433.4, 7084.7) |  | Stroke | 3240.8 (2455.2, 4195.6) |
| Central African Republic | 1 | Tuberculosis | 13041.9 (8838.8, 16401.1) |  | Tuberculosis | 9243.7 (6246.0, 12335.1) |
|  | 2 | Lower respiratory infections | 10322.5(8228.4, 12923.8) |  | COVID-19 | 7902.7 (4677.9, 11969.0) |
|  | 3 | Diarrheal diseases | 9227.4 (6431.3, 12376.5) |  | Neonatal disorders | 5920.9 (4738.8, 7306.8) |
|  | 4 | Neonatal disorders | 6593.0 (5622.7, 7602.8) |  | Malaria | 5663.7 (2226.2, 12789.4) |
|  | 5 | Stroke | 5164.4 (3929.7, 6597.9) |  | HIV/AIDS | 5380.4 (3942.4, 7996.5) |
| Chad | 1 | Diarrheal diseases | 14873.8 (10341.6,19742.6) |  | Diarrheal diseases | 7341.2 (5070.0, 10968.2) |
|  | 2 | Lower respiratory infections | 8312.1 (6812.0, 9880.0) |  | Neonatal disorders | 5846.7 (4721.9, 7197.9) |
|  | 3 | Neonatal disorders | 7438.5 (6399.5, 8377.5) |  | Lower respiratory infections | 5267.0 (4134.9, 6473.7) |
|  | 4 | Measles | 5540.9 (1877.0, 11048.7) |  | COVID-19 | 4044.8 (2957.7, 4816.1) |
|  | 5 | Malaria | 4545.0 (2400.8, 8391.8) |  | Stroke | 3607.2 (2844.3, 4487.2) |
| Chile | 1 | Ischemic heart disease | 2103.4 (2010.7, 2173.9) |  | COVID-19 | 2557.5 (2439.5, 2705.7) |
|  | 2 | Stroke | 2033.0 (1942.9, 2124.6) |  | Low back pain | 1097.7 (779.0, 1480.0) |
|  | 3 | Lower respiratory infections | 1619.1 (1545.2, 1684.4) |  | Other musculoskeletal disorders | 902.7 (642.3, 1241.7) |
|  | 4 | Neonatal disorders | 1346.3 (1258.6, 1435.3) |  | Depressive disorders | 849.7 (534.3, 1232.3) |
|  | 5 | Cirrhosis and other chronic liver diseases | 1226.1 (1184.4, 1266.8) |  | Ischemic heart disease | 808.4 (760.8, 847.5) |
| China | 1 | Stroke | 4834.8 (4242.6, 5418.8) |  | Stroke | 2648.0 (2253.4, 3076.9) |
|  | 2 | Chronic obstructive pulmonary disease | 3852.6 (3350.0, 4279.0) |  | Ischemic heart disease | 1856.5 (1548.7, 2159.8) |
|  | 3 | Lower respiratory infections | 3128.4 (2724.1, 3579.6) |  | Chronic obstructive pulmonary disease | 1227.7 (1048.4, 1442.5) |
|  | 4 | Neonatal disorders | 2557.2 (2257.8, 2859.3) |  | Tracheal, bronchus, and lung cancer | 878.2 (703.5, 1068.7) |
|  | 5 | Ischemic heart disease | 1771.1 (1574.8, 1990.7) |  | Road injuries | 783.2 (674.6, 899.8) |
| Comoros | 1 | Neonatal disorders | 7143.7 (6191.6, 8197.4) |  | Neonatal disorders | 4674.9 (3806.4, 5679.9) |
|  | 2 | Lower respiratory infections | 6860.6 (5317.0, 8525.3) |  | COVID-19 | 3881.4 (3462.9, 4456.6) |
|  | 3 | Tuberculosis | 5502.2 (3797.8, 7443.9) |  | Lower respiratory infections | 2531.3 (2030.8, 3116.4) |
|  | 4 | Stroke | 4158.1 (3318.0, 5106.8) |  | Stroke | 2427.6 (1923.4, 3004.4) |
|  | 5 | Diarrheal diseases | 3845.4 (2575.8, 5613.7) |  | Tuberculosis | 1983.0 (1440.9, 2667.3) |
| Congo | 1 | Tuberculosis | 5740.1 (3929.7, 7371.6) |  | COVID-19 | 5641.5 (3979.8, 7280.7) |
|  | 2 | Diarrheal diseases | 5335.8 (3703.4, 7638.9) |  | HIV/AIDS | 4543.5 (3368.0, 6544.3) |
|  | 3 | Lower respiratory infections | 4830.2 (3724.0, 5970.7) |  | Ischemic heart disease | 3210.4 (2515.6, 3995.7) |
|  | 4 | Stroke | 4779.7 (3872.0, 5804.6) |  | Stroke | 3104.2 (2417.7, 3807.0) |
|  | 5 | Malaria | 4651.9 (2191.4, 8093.1) |  | Malaria | 2991.7 (1206.3, 5628.1) |
| Cook Islands | 1 | Diabetes mellitus | 4157.3 (3584.6, 4805.7) |  | Diabetes mellitus | 4042.4 (3362.3, 4896.1) |
|  | 2 | Ischemic heart disease | 3569.2 (3067.0, 4118.5) |  | Ischemic heart disease | 2344.8 (1931.6, 2837.9) |
|  | 3 | Stroke | 3036.3 (2621.5, 3456.8) |  | Stroke | 1533.3 (1281.9, 1804.2) |
|  | 4 | Lower respiratory infections | 2713.0 (2319.1, 3164.4) |  | Hypertensive heart disease | 1184.9 (935.3, 1495.6) |
|  | 5 | Dengue | 2353.8 (2140.4, 2588.4) |  | Lower respiratory infections | 1016.6 (857.1, 1186.7) |
| Costa Rica | 1 | Ischemic heart disease | 2365.6 (2232.5, 2476.4) |  | COVID-19 | 2503.6 (1707.7, 3380.7) |
|  | 2 | Neonatal disorders | 1487.6 (1370.0, 1613.4) |  | Ischemic heart disease | 1157.8 (1033.4, 1276.4) |
|  | 3 | Road injuries | 1055.0 (1005.2, 1105.7) |  | Diabetes mellitus | 1079.2 (855.1, 1368.6) |
|  | 4 | Congenital birth defects | 989.3 (921.3, 1049.7) |  | Road injuries | 915.1 (842.1, 985.4) |
|  | 5 | Stroke | 969.7 (907.2, 1019.8) |  | Neonatal disorders | 872.3 (731.4, 1036.0) |
| Croatia | 1 | Ischemic heart disease | 4878.6 (4659.0, 5087.2) |  | COVID-19 | 2232.3 (1477.2, 3082.7) |
|  | 2 | Stroke | 3543.2 (3355.9, 3737.0) |  | Ischemic heart disease | 2099.6 (1856.5, 2326.0) |
|  | 3 | Low back pain | 1370.0 (987.7, 1808.9) |  | Low back pain | 1346.9 (961.4, 1812.3) |
|  | 4 | Neonatal disorders | 1346.0 (1206.4, 1498.7) |  | Stroke | 1174.3 (1044.6, 1313.8) |
|  | 5 | Tracheal, bronchus, and lung cancer | 1130.8 (1026.9, 1241.0) |  | Tracheal, bronchus, and lung cancer | 830.2 (719.4, 940.5) |
| Cuba | 1 | Ischemic heart disease | 3975.9 (3835.6, 4068.5) |  | COVID-19 | 5197.1 (4298.0, 6141.5) |
|  | 2 | Stroke | 1633.4 (1570.8, 1692.2) |  | Ischemic heart disease | 2017.0 (1763.5, 2255.7) |
|  | 3 | Road injuries | 1210.6 (1160.6, 1265.1) |  | Stroke | 1093.4 (964.2, 1227.6) |
|  | 4 | Neonatal disorders | 1169.5 (1084.7, 1262.4) |  | Diabetes mellitus | 808.0 (619.3, 1047.5) |
|  | 5 | Self-harm | 871.7 (847.6, 892.0) |  | Depressive disorders | 792.4 (528.7, 1189.5) |
| Cyprus | 1 | Ischemic heart disease | 4121.9 (3765.2, 4505.1) |  | Ischemic heart disease | 1395.0 (1225.3, 1564.9) |
|  | 2 | Stroke | 2497.4 (2180.4, 2839.2) |  | Low back pain | 1070.0 (768.3, 1437.9) |
|  | 3 | Neonatal disorders | 1579.0 (1421.8, 1775.5) |  | Diabetes mellitus | 874.6 (719.5, 1081.8) |
|  | 4 | Diabetes mellitus | 1515.5 (1336.7, 1705.8) |  | Anxiety disorders | 836.8 (531.6, 1247.7) |
|  | 5 | Road injuries | 1173.2 (1074.8, 1290.8) |  | Headache disorders | 727.4 (137.7, 1541.7) |
| Czechia | 1 | Ischemic heart disease | 5796.5 (5603.6, 5941.7) |  | COVID-19 | 2862.7 (1956.2, 3844.5) |
|  | 2 | Stroke | 3494.3 (3286.4, 3707.5) |  | Ischemic heart disease | 2101.4 (1857.1, 2327.2) |
|  | 3 | Low back pain | 1534.8 (1091.2, 2045.9) |  | Low back pain | 1488.4 (1068.9, 2002.2) |
|  | 4 | Tracheal, bronchus, and lung cancer | 1274.8 (1190.3, 1366.8) |  | Diabetes mellitus | 833.3 (667.5, 1037.4) |
|  | 5 | Neonatal disorders | 1242.5 (1137.2, 1363.1) |  | Stroke | 812.2 (719.2, 906.7) |
| Côte d'Ivoire | 1 | Malaria | 9083.2 (4595.1, 15114.2) |  | Malaria | 6120.7 (2558.2, 11294.5) |
|  | 2 | HIV/AIDS | 7264.1 (4326.9, 12687.2) |  | Neonatal disorders | 4944.0 (4143.9, 5826.8) |
|  | 3 | Neonatal disorders | 6747.2 (6052.1, 7457.2) |  | COVID-19 | 4201.4 (3583.9, 4860.4) |
|  | 4 | Lower respiratory infections | 6605.8 (5417.2, 8211.2) |  | Stroke | 3105.7 (2443.4, 3922.1) |
|  | 5 | Diarrheal diseases | 4790.4 (3431.3, 6272.6) |  | Lower respiratory infections | 3003.5 (2263.5, 3871.4) |
| Democratic Republic of the Congo | 1 | Malaria | 10124.2 (5930.9, 15767.8) |  | COVID-19 | 6123.1 (5193.0, 7310.8) |
|  | 2 | Lower respiratory infections | 6980.3 (5248.7, 8627.5) |  | Malaria | 4348.7 (2096.1, 7681.4) |
|  | 3 | Tuberculosis | 6870.0 (4863.6, 9376.1) |  | Tuberculosis | 3652.2 (2569.2, 5712.7) |
|  | 4 | Diarrheal diseases | 5588.5 (4018.7, 7254.3) |  | Neonatal disorders | 3490.9 (2579.8, 4415.8) |
|  | 5 | Neonatal disorders | 4089.8 (3438.5, 4882.4) |  | Lower respiratory infections | 2926.5 (2180.5, 3918.3) |
| Djibouti | 1 | Diarrheal diseases | 5856.1 (4111.9, 7860.8) |  | COVID-19 | 5808.9 (3966.5, 7830.2) |
|  | 2 | Lower respiratory infections | 4559.4 (3599.2, 5531.0) |  | HIV/AIDS | 3572.6 (2328.8, 5394.5) |
|  | 3 | Tuberculosis | 4374.2 (3037.8, 5944.3) |  | Neonatal disorders | 2893.9 (2362.1, 3503.6) |
|  | 4 | Neonatal disorders | 4350.1 (3793.2, 4934.3) |  | Stroke | 2616.8 (2006.2, 3400.3) |
|  | 5 | Stroke | 3610.9 (2750.6, 4693.3) |  | Lower respiratory infections | 2086.0 (1554.3, 2811.4) |
| Dominica | 1 | Ischemic heart disease | 2764.0 (2582.6, 2949.6) |  | COVID-19 | 3324.8 (1439.3, 5902.5) |
|  | 2 | Stroke | 2392.4 (2214.0, 2572.0) |  | Neonatal disorders | 2933.3 (2177.2, 3912.9) |
|  | 3 | Diabetes mellitus | 2131.5 (1915.4, 2410.7) |  | Diabetes mellitus | 2587.2 (2167.9, 3111.0) |
|  | 4 | Neonatal disorders | 2004.1 (1717.8, 2323.0) |  | Stroke | 1755.7 (1536.5, 2037.3) |
|  | 5 | Hypertensive heart disease | 1030.5 (887.5, 1187.9) |  | Ischemic heart disease | 1738.9 (1513.8, 2064.5) |
| Dominican Republic | 1 | Neonatal disorders | 4965.1 (4421.5, 5538.5) |  | Ischemic heart disease | 3104.0 (2494.7, 3824.8) |
|  | 2 | Ischemic heart disease | 2975.4 (2653.1, 3344.4) |  | Neonatal disorders | 3048.0 (2508.3, 3689.2) |
|  | 3 | Diarrheal diseases | 2116.5 (1708.5, 2638.6) |  | Stroke | 1680.1 (1344.0, 2119.1) |
|  | 4 | Stroke | 2044.8 (1788.5, 2307.8) |  | Diabetes mellitus | 1555.4 (1235.3, 1929.9) |
|  | 5 | Lower respiratory infections | 1490.0 (1287.7, 1717.3) |  | COVID-19 | 1340.6 (378.4, 3070.2) |
| Ecuador | 1 | Neonatal disorders | 2458.1 (2166.7, 2777.9) |  | COVID-19 | 4140.8 (3062.2, 5089.8) |
|  | 2 | Lower respiratory infections | 2262.6 (2136.8, 2385.7) |  | Ischemic heart disease | 1433.7 (1158.9, 1770.4) |
|  | 3 | Ischemic heart disease | 1887.1 (1814.8, 1949.6) |  | Diabetes mellitus | 1260.3 (1003.0, 1533.2) |
|  | 4 | Stroke | 1741.3 (1675.6, 1802.8) |  | Neonatal disorders | 1233.8 (1007.4, 1511.1) |
|  | 5 | Diarrheal diseases | 1725.5 (1609.0, 1865.2) |  | Road injuries | 1172.4 (993.3, 1375.1) |
| Egypt | 1 | Ischemic heart disease | 7600.0 (6957.3, 8365.6) |  | Ischemic heart disease | 6924.8 (5844.9, 8119.2) |
|  | 2 | Stroke | 5450.2 (4769.4, 6118.2) |  | COVID-19 | 4935.8 (3433.8, 6356.6) |
|  | 3 | Lower respiratory infections | 5423.1 (4616.0, 6382.6) |  | Stroke | 3282.1 (2736.8, 3918.0) |
|  | 4 | Cirrhosis and other chronic liver diseases | 4292.7 (3653.6, 4626.1) |  | Cirrhosis and other chronic liver diseases | 2032.7 (1667.6, 2460.1) |
|  | 5 | Congenital birth defects | 3204.2 (1259.9, 4194.6) |  | Diabetes mellitus | 1825.3 (1548.0, 2217.1) |
| El Salvador | 1 | Interpersonal violence | 3092.1 (2881.5, 3291.8) |  | Interpersonal violence | 3066.6 (2552.6, 3613.4) |
|  | 2 | Neonatal disorders | 3079.8 (2725.4, 3501.3) |  | COVID-19 | 2806.8 (2376.7, 3353.0) |
|  | 3 | Diarrheal diseases | 2494.1 (2073.6, 2990.0) |  | Ischemic heart disease | 1915.9 (1577.5, 2311.2) |
|  | 4 | Ischemic heart disease | 2481.2 (2315.7, 2623.1) |  | Chronic kidney disease | 1904.0 (1419.6, 2342.5) |
|  | 5 | Conflict and terrorism | 2012.4 (1654.9, 2491.7) |  | Diabetes mellitus | 1628.1 (1358.5, 1960.1) |
| Equatorial Guinea | 1 | Diarrheal diseases | 10820.1 (7042.9, 15078.0) |  | HIV/AIDS | 8878.5 (5321.3, 16333.3) |
|  | 2 | Tuberculosis | 9106.9 (6516.1, 11986.7) |  | COVID-19 | 5977.1 (3360.0, 8635.0) |
|  | 3 | Lower respiratory infections | 7579.0 (5900.5, 9474.2) |  | Malaria | 4404.3 (1924.8, 9111.2) |
|  | 4 | Malaria | 6129.2 (2722.8, 11989.4) |  | Neonatal disorders | 3011.7 (2163.9, 3992.7) |
|  | 5 | Neonatal disorders | 4997.9 (4162.4, 5806.7) |  | Ischemic heart disease | 2751.4 (1907.9, 3774.0) |
| Eritrea | 1 | Conflict and terrorism | 19892.5 (16286.8, 24115.0) |  | COVID-19 | 4661.4 (3891.9, 5325.7) |
|  | 2 | Diarrheal diseases | 10931.6 (7229.2, 14861.9) |  | Tuberculosis | 4645.7 (3154.9, 7158.2) |
|  | 3 | Tuberculosis | 9825.9 (6958.4, 12805.0) |  | Lower respiratory infections | 3858.4 (2468.0, 5692.5) |
|  | 4 | Lower respiratory infections | 8402.7 (6159.0, 11004.4) |  | Stroke | 3449.8 (2705.9, 4284.6) |
|  | 5 | Protein-energy malnutrition | 6135.2 (4383.7, 8176.5) |  | Neonatal disorders | 3286.4 (2596.5, 4196.9) |
| Estonia | 1 | Ischemic heart disease | 6565.7 (6260.2, 6824.2) |  | COVID-19 | 2213.1 (1300.5, 3279.1) |
|  | 2 | Stroke | 3227.9 (3058.2, 3400.3) |  | Ischemic heart disease | 1547.9 (1370.4, 1717.0) |
|  | 3 | Road injuries | 1672.6 (1588.7, 1777.9) |  | Low back pain | 1194.8 (857.3, 1601.9) |
|  | 4 | Neonatal disorders | 1457.0 (1338.8, 1577.9) |  | Hypertensive heart disease | 1037.8 (900.2, 1168.8) |
|  | 5 | Low back pain | 1205.9 (857.4, 1629.9) |  | Depressive disorders | 817.2 (546.1, 1182.8) |
| Ethiopia | 1 | Tuberculosis | 11895.4 (9639.7, 13811.9) |  | COVID-19 | 6729.6 (6097.9, 7426.9) |
|  | 2 | Lower respiratory infections | 9620.9 (7886.1, 11499.3) |  | Neonatal disorders | 5040.4 (4056.3, 6231.0) |
|  | 3 | Diarrheal diseases | 9488.5 (5531.7, 14100.7) |  | Diarrheal diseases | 2211.8 (1627.8, 3062.3) |
|  | 4 | Neonatal disorders | 9051.8 (7995.1, 10615.6) |  | Lower respiratory infections | 2057.3 (1809.9, 2366.4) |
|  | 5 | Conflict and terrorism | 6184.1 (6136.1, 6252.5) |  | Tuberculosis | 1833.2 (1559.8, 2167.1) |
| Fiji | 1 | Ischemic heart disease | 7013.5 (6118.2, 8083.7) |  | Diabetes mellitus | 7387.9 (5995.4, 9157.6) |
|  | 2 | Diabetes mellitus | 5314.2 (4555.7, 6203.5) |  | Ischemic heart disease | 5965.6 (4721.1, 7391.8) |
|  | 3 | Stroke | 3881.7 (3397.6, 4472.3) |  | COVID-19 | 4251.5 (2562.0, 8304.1) |
|  | 4 | Neonatal disorders | 1979.7 (1652.5, 2355.1) |  | Stroke | 2896.3 (2322.8, 3548.8) |
|  | 5 | Asthma | 1734.7 (1473.8, 2051.2) |  | Neonatal disorders | 1640.7 (1290.4, 2090.7) |
| Gabon | 1 | Malaria | 4574.1 (2183.1, 8121.9) |  | COVID-19 | 5622.2 (4197.4, 6609.8) |
|  | 2 | Lower respiratory infections | 3975.9 (3072.5, 4882.9) |  | HIV/AIDS | 3508.6 (2540.5, 5177.0) |
|  | 3 | Neonatal disorders | 3900.1 (3335.4, 4524.0) |  | Neonatal disorders | 2719.7 (2057.1, 3569.1) |
|  | 4 | Diarrheal diseases | 3600.3 (2240.5, 5931.1) |  | Malaria | 2640.5 (964.2, 6050.1) |
|  | 5 | Stroke | 3467.8 (2791.1, 4166.5) |  | Ischemic heart disease | 2479.2 (1901.2, 3118.1) |
| Gambia | 1 | Neonatal disorders | 7679.6 (6743.8, 8509.2) |  | COVID-19 | 5158.5 (4287.3, 6066.1) |
|  | 2 | Lower respiratory infections | 6214.6 (5181.0, 7560.5) |  | Neonatal disorders | 4364.8 (3552.7, 5375.2) |
|  | 3 | Diarrheal diseases | 5349.3 (3824.6, 6961.0) |  | Stroke | 3673.0 (2867.3, 4550.2) |
|  | 4 | Malaria | 4704.1 (1828.7, 8734.2) |  | HIV/AIDS | 3448.9 (2139.1, 5275.8) |
|  | 5 | Stroke | 3742.3 (3019.4, 4681.5) |  | Lower respiratory infections | 2950.0 (2319.8, 3608.4) |
| Georgia | 1 | Ischemic heart disease | 6871.1 (6545.3, 7157.6) |  | COVID-19 | 4703.8 (3451.4, 5942.5) |
|  | 2 | Stroke | 3976.2 (3633.0, 4273.8) |  | Stroke | 3054.1 (2706.2, 3392.2) |
|  | 3 | Neonatal disorders | 3403.7 (2986.8, 3861.6) |  | Ischemic heart disease | 2565.2 (2301.9, 2822.2) |
|  | 4 | Lower respiratory infections | 3248.5 (2885.2, 3674.9) |  | Neonatal disorders | 1121.4 (929.1, 1367.8) |
|  | 5 | Road injuries | 1462.7 (1377.8, 1551.9) |  | Low back pain | 955.4 (686.6, 1273.2) |
| Ghana | 1 | Neonatal disorders | 6397.8 (5755.0, 7153.5) |  | COVID-19 | 4692.4 (3920.9, 5634.3) |
|  | 2 | Malaria | 5948.7 (2995.2, 10003.0) |  | Stroke | 4031.0 (3263.1, 4931.3) |
|  | 3 | Stroke | 4746.8 (3997.1, 5645.8) |  | Neonatal disorders | 3687.7 (2846.5, 4765.0) |
|  | 4 | Diarrheal diseases | 4710.1 (3245.0, 6496.1) |  | Malaria | 2882.4 (1395.6, 5267.9) |
|  | 5 | Lower respiratory infections | 4388.7 (3647.7, 5268.5) |  | HIV/AIDS | 2638.7 (1997.0, 3636.1) |
| Greece | 1 | Ischemic heart disease | 2638.9 (2507.7, 2717.3) |  | Ischemic heart disease | 1443.4 (1353.6, 1503.3) |
|  | 2 | Stroke | 2464.9 (2293.0, 2576.3) |  | COVID-19 | 1324.3 (1282.5, 1412.2) |
|  | 3 | Road injuries | 1295.1 (1212.9, 1394.2) |  | Depressive disorders | 1165.9 (755.8, 1745.0) |
|  | 4 | Low back pain | 1020.6 (724.3, 1367.0) |  | Low back pain | 1012.3 (734.4, 1350.3) |
|  | 5 | Tracheal, bronchus, and lung cancer | 991.2 (933.8, 1053.6) |  | Stroke | 909.5 (830.6, 972.0) |
| Grenada | 1 | Stroke | 3478.7 (3204.7, 3779.8) |  | COVID-19 | 5192.4 (4474.2, 8024.3) |
|  | 2 | Ischemic heart disease | 3227.0 (2951.4, 3535.9) |  | Diabetes mellitus | 2826.7 (2409.1, 3299.1) |
|  | 3 | Diabetes mellitus | 2521.6 (2244.0, 2801.2) |  | Ischemic heart disease | 1924.3 (1679.0, 2155.0) |
|  | 4 | Neonatal disorders | 2330.3 (1977.7, 2792.3) |  | Stroke | 1719.6 (1525.4, 1896.2) |
|  | 5 | Lower respiratory infections | 1594.5 (1425.9, 1776.4) |  | Neonatal disorders | 1572.9 (1309.6, 1884.3) |
| Guinea | 1 | Lower respiratory infections | 11302.0 (8941.1, 14033.3) |  | COVID-19 | 6253.4 (4247.2, 7465.1) |
|  | 2 | Diarrheal diseases | 10550.4 (7012.9, 15162.3) |  | Malaria | 5442.2 (2381.2, 10415.5) |
|  | 3 | Neonatal disorders | 7224.6 (6371.3, 8251.7) |  | Neonatal disorders | 4275.3 (3526.9, 5299.7) |
|  | 4 | Measles | 6815.0 (2423.0, 12786.9) |  | Lower respiratory infections | 4136.9 (3191.4, 5227.3) |
|  | 5 | Malaria | 6219.5 (3261.9, 11984.6) |  | Stroke | 3499.6 (2768.9, 4324.3) |
| Guinea-Bissau | 1 | Diarrheal diseases | 9147.3 (6762.6, 12064.6) |  | COVID-19 | 7069.9 (3963.6, 9890.2) |
|  | 2 | Lower respiratory infections | 9043.7 (7237.1, 11037.1) |  | Stroke | 5000.7 (3985.7, 6110.9) |
|  | 3 | Neonatal disorders | 8505.1 (7281.6, 9845.0) |  | Neonatal disorders | 4869.5 (3960.3, 6015.8) |
|  | 4 | Tuberculosis | 7116.8 (5471.1, 8979.0) |  | HIV/AIDS | 4795.8 (1981.3, 8756.1) |
|  | 5 | Measles | 7041.4 (2672.0, 13039.6) |  | Lower respiratory infections | 3379.2 (2759.2, 4019.3) |
| Guyana | 1 | Stroke | 6140.4 (5577.5, 6755.3) |  | COVID-19 | 6718.7 (3594.5, 11828.9) |
|  | 2 | Neonatal disorders | 5534.8 (4870.2, 6246.3) |  | Diabetes mellitus | 3489.0 (2853.9, 4259.7) |
|  | 3 | Ischemic heart disease | 5067.1 (4569.7, 5581.4) |  | Ischemic heart disease | 3075.2 (2425.8, 3871.8) |
|  | 4 | Diabetes mellitus | 2781.0 (2452.6, 3194.9) |  | Stroke | 2909.2 (2301.6, 3616.0) |
|  | 5 | Diarrheal diseases | 1817.7 (1549.7, 2117.7) |  | Neonatal disorders | 2653.7 (2008.8, 3411.8) |
| Hungary | 1 | Ischemic heart disease | 5265.3 (5096.0, 5407.8) |  | COVID-19 | 3889.0 (2732.4, 5185.5) |
|  | 2 | Stroke | 3689.8 (3513.6, 3860.7) |  | Ischemic heart disease | 2781.0 (2470.6, 3060.4) |
|  | 3 | Neonatal disorders | 1938.8 (1802.8, 2089.4) |  | Low back pain | 1573.5 (1118.6, 2089.5) |
|  | 4 | Low back pain | 1593.2 (1145.9, 2126.5) |  | Stroke | 1143.4 (999.5, 1284.7) |
|  | 5 | Cirrhosis and other chronic liver diseases | 1525.1 (1453.8, 1593.5) |  | Tracheal, bronchus, and lung cancer | 1126.4 (962.1, 1295.8) |
| Indonesia | 1 | Diarrheal diseases | 7454.6 (5098.8, 10212.9) |  | Stroke | 4587.1 (3889.4, 5248.2) |
|  | 2 | Stroke | 4869.3 (4245.8, 5526.5) |  | Ischemic heart disease | 3043.1 (2527.3, 3544.7) |
|  | 3 | Neonatal disorders | 3739.2 (3287.5, 4207.4) |  | COVID-19 | 2961.5 (1629.1, 5434.2) |
|  | 4 | Tuberculosis | 3709.1 (2833.4, 4285.2) |  | Neonatal disorders | 2051.4 (1657.2, 2453.3) |
|  | 5 | Lower respiratory infections | 2564.2 (2221.6, 3040.4) |  | Tuberculosis | 1220.1 (1026.9, 1443.6) |
| Iran (Islamic Republic of) | 1 | Neonatal disorders | 5231.4 (4494.8, 6194.7) |  | COVID-19 | 5176.1 (4891.0, 5564.3) |
|  | 2 | Ischemic heart disease | 5166.8 (4824.5, 5465.6) |  | Ischemic heart disease | 2731.3 (2517.3, 2920.9) |
|  | 3 | Exposure to forces of nature | 4035.0 (3667.0, 4439.0) |  | Stroke | 1229.2 (1119.5, 1332.6) |
|  | 4 | Road injuries | 3463.3 (3211.5, 3810.7) |  | Road injuries | 1221.7 (1135.5, 1304.9) |
|  | 5 | Congenital birth defects | 3331.7 (1947.0, 4101.0) |  | Low back pain | 1028.0 (740.5, 1370.3) |
| Iraq | 1 | Ischemic heart disease | 5522.0 (4733.0, 6492.4) |  | Ischemic heart disease | 4905.3 (3858.3, 5827.7) |
|  | 2 | Neonatal disorders | 5130.0 (4612.1, 5802.4) |  | COVID-19 | 4269.8 (3093.4, 5447.0) |
|  | 3 | Stroke | 4564.2 (3882.8, 5352.2) |  | Stroke | 3324.6 (2661.1, 3981.4) |
|  | 4 | Congenital birth defects | 2280.7 (1331.3, 2915.2) |  | Diabetes mellitus | 2191.1 (1758.2, 2756.8) |
|  | 5 | Lower respiratory infections | 1634.7 (1344.8, 2036.8) |  | Neonatal disorders | 2042.0 (1636.2, 2521.4) |
| Italy | 1 | Ischemic heart disease | 1988.7 (1868.5, 2056.7) |  | Low back pain | 1082.2 (773.3, 1455.9) |
|  | 2 | Stroke | 1484.5 (1373.0, 1560.4) |  | COVID-19 | 902.5 (825.4, 1024.1) |
|  | 3 | Road injuries | 1091.4 (1020.6, 1174.1) |  | Anxiety disorders | 814.0 (553.8, 1127.4) |
|  | 4 | Low back pain | 1088.8 (784.0, 1463.1) |  | Depressive disorders | 813.6 (551.1, 1120.3) |
|  | 5 | Neonatal disorders | 1055.4 (996.4, 1123.8) |  | Headache disorders | 788.7 (136.8, 1690.5) |
| Jamaica | 1 | Neonatal disorders | 3245.0 (2781.3, 3752.4) |  | COVID-19 | 2451.9 (1528.8, 3474.2) |
|  | 2 | Stroke | 2490.4 (2376.8, 2594.8) |  | Neonatal disorders | 2258.9 (1744.8, 2906.3) |
|  | 3 | Diabetes mellitus | 1946.8 (1792.9, 2135.3) |  | Diabetes mellitus | 2133.2 (1757.6, 2603.6) |
|  | 4 | Ischemic heart disease | 1357.0 (1290.0, 1417.0) |  | Stroke | 1709.4 (1360.8, 2126.3) |
|  | 5 | Congenital birth defects | 786.0 (655.4, 924.2) |  | Interpersonal violence | 1356.5 (1068.8, 1744.9) |
| Kazakhstan | 1 | Ischemic heart disease | 5827.0 (5426.2, 6249.3) |  | COVID-19 | 5695.0 (4685.9, 6701.4) |
|  | 2 | Stroke | 3841.1 (3510.4, 4168.0) |  | Ischemic heart disease | 3970.5 (3569.8, 4401.1) |
|  | 3 | Lower respiratory infections | 2771.8 (2542.8, 3057.4) |  | Stroke | 3099.2 (2711.1, 3486.1) |
|  | 4 | Neonatal disorders | 1723.2 (1529.0, 1941.6) |  | Cardiomyopathy and myocarditis | 1202.9 (979.7, 1442.3) |
|  | 5 | Road injuries | 1581.3 (1475.6, 1691.1) |  | Cirrhosis and other chronic liver diseases | 1139.6 (988.6, 1365.7) |
| Kenya | 1 | HIV/AIDS | 6009.6 (4060.8, 9071.6) |  | COVID-19 | 7347.2 (6636.5, 8106.8) |
|  | 2 | Diarrheal diseases | 5826.6 (4137.8, 7841.0) |  | HIV/AIDS | 4125.6 (3597.0, 4789.7) |
|  | 3 | Lower respiratory infections | 4448.3 (3838.4, 5351.4) |  | Neonatal disorders | 2808.5 (2321.7, 3424.7) |
|  | 4 | Tuberculosis | 4298.8 (2578.3, 6594.7) |  | Tuberculosis | 2795.5 (1694.6, 3743.5) |
|  | 5 | Neonatal disorders | 3350.6 (2938.4, 3816.1) |  | Lower respiratory infections | 2175.1 (1770.7, 2638.8) |
| Kiribati | 1 | Tuberculosis | 6130.2 (5124.3, 7225.3) |  | Diabetes mellitus | 5569.4 (4520.2, 6970.9) |
|  | 2 | Diarrheal diseases | 5508.9 (3920.2, 7113.3) |  | Ischemic heart disease | 4969.4 (4015.5, 6139.3) |
|  | 3 | Stroke | 5357.2 (4441.6, 6217.2) |  | Stroke | 4685.5 (3886.9, 5751.1) |
|  | 4 | Neonatal disorders | 5200.7 (4233.5, 6343.6) |  | Tuberculosis | 3528.2 (2705.4, 4733.5) |
|  | 5 | Ischemic heart disease | 4765.1 (3903.1, 5656.9) |  | Neonatal disorders | 3165.0 (2577.3, 3912.3) |
| Kuwait | 1 | Ischemic heart disease | 4299.3 (3968.7, 4614.3) |  | Ischemic heart disease | 2299.4 (1917.0, 2762.5) |
|  | 2 | Conflict and terrorism | 4214.8 (3827.6, 4644.5) |  | Diabetes mellitus | 1658.0 (1256.0, 2174.6) |
|  | 3 | Road injuries | 1500.7 (1403.2, 1613.7) |  | COVID-19 | 1446.0 (1233.2, 1846.4) |
|  | 4 | Congenital birth defects | 1390.3 (1199.5, 1572.2) |  | Low back pain | 933.6 (656.4, 1248.0) |
|  | 5 | Neonatal disorders | 1317.6 (1161.7, 1467.5) |  | Congenital birth defects | 768.8 (661.4, 912.3) |
| Kyrgyzstan | 1 | Ischemic heart disease | 5149.8 (4726.5, 5557.7) |  | Ischemic heart disease | 4780.5 (4093.6, 5490.6) |
|  | 2 | Lower respiratory infections | 5028.5 (4543.2, 5574.6) |  | COVID-19 | 2661.9 (1821.5, 3613.9) |
|  | 3 | Stroke | 4315.2 (4012.3, 4629.6) |  | Stroke | 2190.0 (1870.2, 2526.3) |
|  | 4 | Neonatal disorders | 3081.8 (2776.4, 3414.8) |  | Neonatal disorders | 1943.8 (1706.5, 2206.4) |
|  | 5 | Chronic obstructive pulmonary disease | 1977.1 (1839.5, 2129.6) |  | Cirrhosis and other chronic liver diseases | 1149.4 (955.6, 1339.8) |
| Lao People's Democratic Republic | 1 | Lower respiratory infections | 10758.1 (8656.9, 13491.0) |  | Stroke | 4061.5 (3276.8, 4988.7) |
|  | 2 | Diarrheal diseases | 8292.4 (5819.1, 11597.3) |  | Ischemic heart disease | 3667.1 (2896.8, 4573.2) |
|  | 3 | Stroke | 7166.4 (5786.0, 8836.9) |  | Neonatal disorders | 3426.9 (2694.8, 4265.7) |
|  | 4 | Neonatal disorders | 6929.0 (5833.0, 8323.6) |  | Lower respiratory infections | 2110.6 (1599.3, 2693.6) |
|  | 5 | Tuberculosis | 5640.3 (3726.1, 7337.5) |  | COVID-19 | 1861.5 (739.8, 3674.7) |
| Latvia | 1 | Ischemic heart disease | 6428.3 (6135.9, 6666.4) |  | COVID-19 | 3499.6 (2339.9, 4895.9) |
|  | 2 | Stroke | 3723.0 (3544.5, 3899.6) |  | Ischemic heart disease | 2916.5 (2581.6, 3230.6) |
|  | 3 | Road injuries | 2199.6 (2098.8, 2317.9) |  | Stroke | 1912.7 (1701.2, 2112.5) |
|  | 4 | Falls | 1316.7 (1092.3, 1605.6) |  | Low back pain | 1173.0 (834.7, 1566.1) |
|  | 5 | Congenital birth defects | 1216.9 (1143.9, 1285.1) |  | Cardiomyopathy and myocarditis | 984.3 (845.1, 1124.7) |
| Lebanon | 1 | Ischemic heart disease | 4913.4 (4089.9, 6007.8) |  | COVID-19 | 5058.7 (4590.2, 5754.1) |
|  | 2 | Interpersonal violence | 2978.0 (2803.5, 3170.5) |  | Ischemic heart disease | 1735.3 (1488.4, 2027.4) |
|  | 3 | Stroke | 2946.0 (2360.0, 3714.8) |  | Diabetes mellitus | 1541.3 (1196.1, 1935.6) |
|  | 4 | Neonatal disorders | 2939.4 (2443.1, 3527.1) |  | Depressive disorders | 1128.5 (728.6, 1641.3) |
|  | 5 | Conflict and terrorism | 2478.5 (1594.6, 3989.9) |  | Anxiety disorders | 981.0 (592.8, 1442.8) |
| Lesotho | 1 | Diarrheal diseases | 9084.7 (6892.2, 11573.1) |  | HIV/AIDS | 22487.7 (19592.6, 25927.0) |
|  | 2 | Neonatal disorders | 5936.0 (5226.5, 6785.2) |  | COVID-19 | 20796.7 (16853.8, 24402.4) |
|  | 3 | Tuberculosis | 5665.8 (3762.0, 7983.5) |  | Tuberculosis | 7772.7 (5196.9, 10011.2) |
|  | 4 | Lower respiratory infections | 4293.0 (3641.4, 5107.3) |  | Neonatal disorders | 5054.9 (4067.3, 6163.1) |
|  | 5 | Stroke | 3038.3 (2441.0, 3664.9) |  | Diarrheal diseases | 4896.7 (3295.5, 6574.3) |
| Liberia | 1 | Malaria | 10596.3 (4497.8, 19274.9) |  | Malaria | 6836.6 (2425.7, 13296.0) |
|  | 2 | Diarrheal diseases | 10396.8 (7669.7, 13170.6) |  | Neonatal disorders | 4405.8 (3438.0, 5719.1) |
|  | 3 | Neonatal disorders | 9533.4 (8400.0, 10678.6) |  | COVID-19 | 3957.5 (3314.4, 4661.6) |
|  | 4 | Conflict and terrorism | 9453.0 (8983.8, 9951.8) |  | Stroke | 3198.8 (2505.5, 4057.6) |
|  | 5 | Lower respiratory infections | 9312.5 (7116.1, 11888.4) |  | Ischemic heart disease | 2264.0 (1760.7, 2953.8) |
| Libya | 1 | Ischemic heart disease | 3439.5 (2793.3, 4231.4) |  | COVID-19 | 3767.5 (2869.4, 4827.0) |
|  | 2 | Neonatal disorders | 2819.2 (2293.5, 3425.8) |  | Ischemic heart disease | 3765.5 (2952.8, 4839.1) |
|  | 3 | Road injuries | 2598.4 (2201.1, 3012.5) |  | Road injuries | 2141.1 (1735.9, 2643.0) |
|  | 4 | Congenital birth defects | 2431.4 (1698.5, 3031.4) |  | Stroke | 1796.9 (1360.4, 2357.8) |
|  | 5 | Stroke | 1925.4 (1546.6, 2416.4) |  | Congenital birth defects | 1610.3 (1186.9, 2070.6) |
| Lithuania | 1 | Ischemic heart disease | 6509.0 (6200.6, 6730.6) |  | Ischemic heart disease | 3332.3 (3001.0, 3676.8) |
|  | 2 | Stroke | 2081.7 (1960.1, 2206.4) |  | COVID-19 | 3079.0 (1965.6, 4386.9) |
|  | 3 | Road injuries | 1759.3 (1679.1, 1841.7) |  | Stroke | 1335.3 (1182.3, 1476.9) |
|  | 4 | Self-harm | 1247.3 (1200.5, 1299.9) |  | Low back pain | 1191.0 (848.6, 1584.1) |
|  | 5 | Low back pain | 1219.6 (878.7, 1641.2) |  | Depressive disorders | 969.6 (643.4, 1399.9) |
| Luxembourg | 1 | Ischemic heart disease | 2820.3 (2685.4, 2933.7) |  | Low back pain | 1075.2 (763.7, 1449.6) |
|  | 2 | Stroke | 2090.1 (1976.2, 2181.6) |  | COVID-19 | 843.7 (653.0, 1062.0) |
|  | 3 | Road injuries | 1349.4 (1262.7, 1444.2) |  | Ischemic heart disease | 789.8 (717.7, 867.8) |
|  | 4 | Low back pain | 1105.3 (793.6, 1478.3) |  | Anxiety disorders | 758.3 (485.2, 1141.6) |
|  | 5 | Tracheal, bronchus, and lung cancer | 1057.7 (988.4, 1124.3) |  | Falls | 742.4 (556.5, 968.6) |
| Madagascar | 1 | Diarrheal diseases | 8591.0 (6922.2, 10287.8) |  | COVID-19 | 6048.1 (5004.8, 6841.2) |
|  | 2 | Lower respiratory infections | 7205.5 (6456.5, 8022.4) |  | Stroke | 4633.5 (3518.8, 5840.1) |
|  | 3 | Tuberculosis | 5989.9 (4827.6, 7309.2) |  | Diarrheal diseases | 3530.2 (2280.1, 5206.5) |
|  | 4 | Stroke | 5923.7 (5227.8, 6706.2) |  | Neonatal disorders | 3123.3 (2479.6, 3834.9) |
|  | 5 | Protein-energy malnutrition | 4810.2 (3977.8, 5613.7) |  | Lower respiratory infections | 3092.8 (2493.0, 3732.1) |
| Malaysia | 1 | Ischemic heart disease | 3880.7 (3601.2, 4129.3) |  | Ischemic heart disease | 3180.4 (2984.1, 3352.2) |
|  | 2 | Stroke | 3321.8 (3058.8, 3565.9) |  | COVID-19 | 2955.2 (2766.0, 3588.1) |
|  | 3 | Lower respiratory infections | 1558.9 (1388.4, 1822.2) |  | Stroke | 2122.3 (1934.6, 2323.9) |
|  | 4 | Road injuries | 1496.3 (1397.3, 1601.4) |  | Lower respiratory infections | 1516.0 (1272.6, 1690.7) |
|  | 5 | Neonatal disorders | 1489.0 (1358.1, 1662.9) |  | Road injuries | 1140.1 (1060.5, 1224.6) |
| Maldives | 1 | Stroke | 5353.1 (4748.1, 6018.7) |  | COVID-19 | 1632.8 (1512.9, 2101.0) |
|  | 2 | Neonatal disorders | 4548.1 (3935.8, 5222.1) |  | Stroke | 1601.7 (1348.0, 1849.8) |
|  | 3 | Diarrheal diseases | 3836.0 (2834.9, 4892.8) |  | Ischemic heart disease | 1481.0 (1238.4, 1760.0) |
|  | 4 | Ischemic heart disease | 3820.0 (3377.2, 4272.6) |  | Neonatal disorders | 1438.0 (1199.8, 1736.4) |
|  | 5 | Tuberculosis | 2419.9 (1805.9, 3094.7) |  | Diabetes mellitus | 860.6 (696.8, 1093.8) |
| Mali | 1 | Malaria | 10987.5 (5844.7, 19470.5) |  | COVID-19 | 7923.4 (7232.6, 8727.8) |
|  | 2 | Neonatal disorders | 10934.2 (9672.1, 12222.2) |  | Neonatal disorders | 7062.8 (5972.9, 8338.8) |
|  | 3 | Diarrheal diseases | 9373.5 (6541.4, 12488.7) |  | Malaria | 5353.1 (2601.6, 10694.8) |
|  | 4 | Measles | 8438.4 (3104.7, 16513.8) |  | Stroke | 2558.3 (2069.9, 3139.5) |
|  | 5 | Lower respiratory infections | 5219.9 (4219.0, 6389.2) |  | Lower respiratory infections | 2301.0 (1832.9, 2882.5) |
| Malta | 1 | Ischemic heart disease | 3799.4 (3582.5, 3983.4) |  | Ischemic heart disease | 1270.5 (1140.1, 1387.8) |
|  | 2 | Stroke | 1567.2 (1457.6, 1655.4) |  | Low back pain | 1136.6 (823.8, 1525.5) |
|  | 3 | Low back pain | 1142.6 (818.1, 1540.7) |  | Anxiety disorders | 806.3 (503.2, 1241.7) |
|  | 4 | Neonatal disorders | 1134.9 (1028.7, 1239.9) |  | Diabetes mellitus | 739.3 (586.1, 961.2) |
|  | 5 | Congenital birth defects | 844.8 (772.3, 928.0) |  | Headache disorders | 719.6 (137.0, 1536.1) |
| Mauritania | 1 | Neonatal disorders | 5887.3 (5141.4, 6716.5) |  | Neonatal disorders | 3364.7 (2882.7, 3954.0) |
|  | 2 | Diarrheal diseases | 5030.9 (3384.7, 7103.4) |  | COVID-19 | 2870.0 (1986.1, 3618.1) |
|  | 3 | Lower respiratory infections | 4924.6 (4141.1, 5662.0) |  | Stroke | 2712.2 (2085.9, 3546.0) |
|  | 4 | Stroke | 4326.1 (3573.2, 5323.4) |  | Ischemic heart disease | 2126.4 (1570.6, 2744.4) |
|  | 5 | Road injuries | 2778.7 (2342.9, 3188.0) |  | Lower respiratory infections | 1989.1 (1591.7, 2393.6) |
| Micronesia  (Federated States of) | 1 | Stroke | 6424.2 (5241.8, 7784.5) |  | Ischemic heart disease | 6279.7 (4925.7, 8032.0) |
|  | 2 | Ischemic heart disease | 6402.7 (5174.7, 7820.2) |  | Stroke | 4759.5 (3857.8, 5956.4) |
|  | 3 | Lower respiratory infections | 3618.2 (2950.5, 4277.6) |  | Diabetes mellitus | 3951.8 (3200.2, 4831.6) |
|  | 4 | Neonatal disorders | 2828.4 (2328.7, 3504.7) |  | Lower respiratory infections | 1676.9 (1333.1, 2092.7) |
|  | 5 | Diabetes mellitus | 2660.1 (2172.7, 3308.3) |  | Chronic kidney disease | 1629.5 (1205.4, 2229.3) |
| Mongolia | 1 | Lower respiratory infections | 7874.1 (6715.5, 9077.8) |  | Ischemic heart disease | 3892.3 (3366.1, 4387.1) |
|  | 2 | Ischemic heart disease | 6067.4 (5273.2, 6935.8) |  | Stroke | 3036.3 (2536.0, 3573.5) |
|  | 3 | Stroke | 4848.2 (4136.0, 5607.8) |  | COVID-19 | 2680.7 (1446.0, 4300.5) |
|  | 4 | Neonatal disorders | 3285.8 (2786.1, 3850.5) |  | Liver cancer | 1993.2 (1531.4, 2547.2) |
|  | 5 | Tuberculosis | 2118.4 (1411.6, 2899.3) |  | Neonatal disorders | 1394.9 (1177.5, 1684.1) |
| Montenegro | 1 | Stroke | 3472.3 (3076.8, 3871.3) |  | COVID-19 | 5487.9 (4557.4, 6563.9) |
|  | 2 | Ischemic heart disease | 3277.1 (2917.9, 3636.0) |  | Ischemic heart disease | 3538.2 (3127.1, 3990.7) |
|  | 3 | Neonatal disorders | 2167.6 (1945.9, 2395.6) |  | Stroke | 3517.2 (3030.4, 4082.9) |
|  | 4 | Low back pain | 1413.1 (1014.2, 1907.0) |  | Low back pain | 1422.7 (1010.3, 1912.6) |
|  | 5 | Tracheal, bronchus, and lung cancer | 1166.1 (981.6, 1379.0) |  | Tracheal, bronchus, and lung cancer | 1194.2 (940.7, 1476.8) |
| Morocco | 1 | Ischemic heart disease | 6313.8 (5422.2, 7352.0) |  | Ischemic heart disease | 5211.8 (3980.8, 6188.4) |
|  | 2 | Neonatal disorders | 4516.6 (3936.0, 5112.3) |  | COVID-19 | 2732.1 (2161.4, 3404.1) |
|  | 3 | Stroke | 3319.7 (2646.6, 4131.8) |  | Stroke | 2478.0 (1951.6, 3106.0) |
|  | 4 | Diarrheal diseases | 2869.4 (2247.2, 3628.0) |  | Diabetes mellitus | 1593.8 (1214.2, 2063.7) |
|  | 5 | Lower respiratory infections | 2863.6 (2334.0, 3529.9) |  | Neonatal disorders | 1475.8 (1209.1, 1749.3) |
| Mozambique | 1 | Malaria | 12973.1 (7642.1, 21884.7) |  | HIV/AIDS | 9718.0 (8633.6, 11317.9) |
|  | 2 | Neonatal disorders | 8835.5 (6872.3, 10490.8) |  | COVID-19 | 9661.3 (7519.2, 11485.8) |
|  | 3 | Tuberculosis | 8183.2 (6287.2, 11034.3) |  | Malaria | 5637.6 (1872.8, 12272.5) |
|  | 4 | Diarrheal diseases | 7727.9 (4990.6, 10510.1) |  | Stroke | 5087.8 (4029.2, 6246.6) |
|  | 5 | Lower respiratory infections | 6510.2 (5081.3, 8829.4) |  | Neonatal disorders | 4764.1 (3444.2, 6175.3) |
| Myanmar | 1 | Lower respiratory infections | 8343.9 (6530.6, 10401.8) |  | Stroke | 4036.4 (3276.2, 4964.3) |
|  | 2 | Stroke | 7027.9 (5729.5, 8536.2) |  | Ischemic heart disease | 2773.1 (2241.1, 3446.1) |
|  | 3 | Tuberculosis | 6104.3 (4783.2, 7525.7) |  | Neonatal disorders | 2705.5 (2226.8, 3321.6) |
|  | 4 | Diarrheal diseases | 5987.5 (3249.7, 9471.0) |  | COVID-19 | 2387.7 (978.3, 4673.7) |
|  | 5 | Neonatal disorders | 4524.4 (3782.3, 5429.1) |  | Diabetes mellitus | 2002.0 (1638.9, 2418.9) |
| Namibia | 1 | Diarrheal diseases | 6048.0 (4265.7, 8271.8) |  | COVID-19 | 12461.5 (10413.0, 14780.3) |
|  | 2 | Tuberculosis | 4812.3 (3302.2, 6240.2) |  | HIV/AIDS | 7223.7 (6211.5, 8699.9) |
|  | 3 | Neonatal disorders | 4286.4 (3803.6, 4835.8) |  | Stroke | 3093.3 (2407.4, 3861.6) |
|  | 4 | Lower respiratory infections | 4158.4 (3299.6, 5258.3) |  | Neonatal disorders | 2800.2 (2290.9, 3460.3) |
|  | 5 | Stroke | 3959.6 (3357.7, 4551.2) |  | Lower respiratory infections | 2447.1 (1755.9, 3312.9) |
| Nepal | 1 | Lower respiratory infections | 7499.9 (6319.7, 8673.0) |  | COVID-19 | 5166.6 (4749.0, 5988.0) |
|  | 2 | Neonatal disorders | 6985.5 (5886.5, 8200.1) |  | Neonatal disorders | 3348.5 (2645.5, 4260.5) |
|  | 3 | Diarrheal diseases | 5813.0 (4542.8, 7221.3) |  | Ischemic heart disease | 2890.1 (2320.6, 3596.1) |
|  | 4 | Tuberculosis | 4967.7 (3697.9, 6380.2) |  | Chronic obstructive pulmonary disease | 2836.0 (2275.3, 3485.0) |
|  | 5 | Chronic obstructive pulmonary disease | 3611.3 (2493.0, 4552.9) |  | Stroke | 1801.1 (1453.6, 2269.3) |
| New Zealand | 1 | Ischemic heart disease | 3586.9 (3420.3, 3716.2) |  | Low back pain | 1331.3 (951.0, 1787.5) |
|  | 2 | Road injuries | 1602.4 (1504.7, 1713.8) |  | Ischemic heart disease | 1048.6 (948.6, 1108.7) |
|  | 3 | Low back pain | 1410.5 (1008.0, 1887.7) |  | Anxiety disorders | 880.9 (556.5, 1268.6) |
|  | 4 | Stroke | 1214.3 (1125.5, 1290.6) |  | Falls | 703.3 (512.7, 936.3) |
|  | 5 | Tracheal, bronchus, and lung cancer | 866.9 (808.5, 927.0) |  | Depressive disorders | 667.9 (441.1, 936.9) |
| Nicaragua | 1 | Neonatal disorders | 3403.1 (2982.8, 3825.3) |  | COVID-19 | 6313.3 (5887.1, 6904.4) |
|  | 2 | Diarrheal diseases | 3222.0 (2567.1, 4095.7) |  | Chronic kidney disease | 1596.8 (1282.9, 1885.7) |
|  | 3 | Lower respiratory infections | 2258.6 (1968.9, 2576.8) |  | Ischemic heart disease | 1524.2 (1300.6, 1784.5) |
|  | 4 | Ischemic heart disease | 1733.2 (1594.0, 1853.9) |  | Diabetes mellitus | 1471.5 (1215.4, 1783.3) |
|  | 5 | Congenital birth defects | 1584.7 (971.6, 2115.3) |  | Neonatal disorders | 1389.6 (1091.2, 1758.7) |
| Niger | 1 | Diarrheal diseases | 16521.0 (12043.0, 20944.2) |  | Malaria | 7344.9 (2624.9, 13447.2) |
|  | 2 | Lower respiratory infections | 14239.4 (11089.6, 18015.7) |  | Neonatal disorders | 4113.3 (3414.4, 4907.2) |
|  | 3 | Malaria | 10753.3 (4549.5, 21169.5) |  | COVID-19 | 4038.1 (3066.7, 4820.2) |
|  | 4 | Measles | 6909.3 (2181.4, 14371.5) |  | Diarrheal diseases | 3738.4 (2407.2, 5420.8) |
|  | 5 | Neonatal disorders | 6701.7 (5859.0, 7670.5) |  | Lower respiratory infections | 3556.6 (2638.2, 4689.7) |
| Nigeria | 1 | Diarrheal diseases | 11063.4 (7997.7, 13707.0) |  | Malaria | 6017.0 (2146.4, 12367.8) |
|  | 2 | Lower respiratory infections | 9036.8 (7377.0, 10836.9) |  | Neonatal disorders | 5909.7 (4903.0, 7089.1) |
|  | 3 | Malaria | 8872.6 (4716.2, 15153.4) |  | COVID-19 | 3790.0 (3346.0, 4361.2) |
|  | 4 | Neonatal disorders | 7276.2 (6643.4, 8062.1) |  | Lower respiratory infections | 3431.3 (2511.2, 4497.7) |
|  | 5 | Tuberculosis | 3939.5 (3061.8, 5098.1) |  | Diarrheal diseases | 3186.7 (2274.3, 4213.1) |
| Niue | 1 | Ischemic heart disease | 5362.8 (4384.3, 6481.7) |  | Ischemic heart disease | 5269.8 (4331.6, 6254.3) |
|  | 2 | Stroke | 4122.3 (3452.6, 4965.1) |  | Diabetes mellitus | 4208.0 (3367.8, 5170.5) |
|  | 3 | Diabetes mellitus | 2577.5 (2171.6, 3085.4) |  | Neonatal disorders | 3554.6 (3209.2, 3851.0) |
|  | 4 | Lower respiratory infections | 2132.9 (1751.7, 2576.4) |  | Lower respiratory infections | 3181.3 (2857.0, 3574.9) |
|  | 5 | Neonatal disorders | 1936.3 (1630.0, 2296.0) |  | Stroke | 3181.1 (2663.2, 3719.1) |
| North Macedonia | 1 | Stroke | 5343.5 (4903.7, 5812.0) |  | COVID-19 | 6524.3 (5226.1, 7759.1) |
|  | 2 | Ischemic heart disease | 4233.5 (3841.0, 4664.6) |  | Stroke | 4267.2 (3549.7, 4993.9) |
|  | 3 | Neonatal disorders | 3615.9 (3300.2, 3937.9) |  | Ischemic heart disease | 3156.9 (2698.2, 3663.3) |
|  | 4 | Congenital birth defects | 1363.2 (984.3, 1706.7) |  | Low back pain | 1299.8 (924.6, 1753.5) |
|  | 5 | Low back pain | 1309.9 (940.5, 1755.0) |  | Diabetes mellitus | 1250.7 (1020.3, 1553.7) |
| Oman | 1 | Ischemic heart disease | 6490.3 (5047.8, 8203.0) |  | COVID-19 | 5037.5 (4721.3, 5361.6) |
|  | 2 | Road injuries | 4996.8 (4077.5, 6145.6) |  | Ischemic heart disease | 3393.7 (2837.0, 4065.1) |
|  | 3 | Stroke | 2842.4 (2209.2, 3550.1) |  | Road injuries | 1792.4 (1526.7, 2082.0) |
|  | 4 | Neonatal disorders | 2560.9 (2168.7, 3033.0) |  | Diabetes mellitus | 1632.8 (1363.9, 1970.1) |
|  | 5 | Congenital birth defects | 1607.8 (1183.3, 2036.5) |  | Stroke | 1470.8 (1215.6, 1716.3) |
| Pakistan | 1 | Neonatal disorders | 7389.7 (6585.4, 8341.7) |  | Neonatal disorders | 6613.1 (5541.7, 7903.6) |
|  | 2 | Diarrheal diseases | 5683.3 (4255.5, 7629.8) |  | COVID-19 | 4851.4 (4098.7, 5954.4) |
|  | 3 | Tuberculosis | 3736.1 (2391.4, 5160.3) |  | Ischemic heart disease | 4069.0 (3417.0, 4985.8) |
|  | 4 | Lower respiratory infections | 3608.3 (3005.5, 4231.3) |  | Stroke | 2194.4 (1832.7, 2676.9) |
|  | 5 | Ischemic heart disease | 3174.7 (2549.7, 3615.9) |  | Diabetes mellitus | 1600.4 (1332.8, 1917.8) |
| Panama | 1 | Ischemic heart disease | 1985.6 (1871.8, 2070.9) |  | COVID-19 | 1819.9 (1707.0, 2122.9) |
|  | 2 | Neonatal disorders | 1840.8 (1612.3, 2073.6) |  | Diabetes mellitus | 1271.8 (1031.1, 1582.9) |
|  | 3 | Stroke | 1478.4 (1396.3, 1546.8) |  | Ischemic heart disease | 1097.3 (870.2, 1306.8) |
|  | 4 | Congenital birth defects | 1287.6 (1121.5, 1468.6) |  | Neonatal disorders | 1094.1 (886.4, 1341.3) |
|  | 5 | Road injuries | 1210.9 (1148.9, 1276.3) |  | Congenital birth defects | 992.2 (805.2, 1209.9) |
| Papua New Guinea | 1 | Lower respiratory infections | 5982.3 (4932.2, 7312.4) |  | COVID-19 | 4826.3 (1468.3, 9381.5) |
|  | 2 | Stroke | 4956.1 (3857.7, 6269.8) |  | Stroke | 3845.8 (2971.5, 4796.0) |
|  | 3 | Diarrheal diseases | 3754.8 (2592.3, 5100.7) |  | Ischemic heart disease | 3369.3 (2518.5, 4367.4) |
|  | 4 | Chronic obstructive pulmonary disease | 3724.7 (2788.2, 4812.0) |  | Diabetes mellitus | 3083.5 (2526.5, 3772.4) |
|  | 5 | Malaria | 3437.2 (781.9, 10601.6) |  | Lower respiratory infections | 3039.1 (2446.5, 3737.4) |
| Peru | 1 | Lower respiratory infections | 4847.3 (4327.4, 5449.4) |  | COVID-19 | 7815.3 (7632.3, 8161.2) |
|  | 2 | Neonatal disorders | 3595.6 (3180.4, 4073.5) |  | Lower respiratory infections | 1351.9 (1066.8, 1703.8) |
|  | 3 | Tuberculosis | 2060.0 (1758.0, 2401.3) |  | Neonatal disorders | 1227.0 (911.2, 1536.5) |
|  | 4 | Congenital birth defects | 1622.3 (928.5, 2159.8) |  | Ischemic heart disease | 916.1 (723.5, 1141.7) |
|  | 5 | Ischemic heart disease | 1595.1 (1382.3, 1816.2) |  | Anxiety disorders | 866.2 (530.3, 1296.6) |
| Philippines | 1 | Lower respiratory infections | 3767.3 (3373.4, 4363.1) |  | COVID-19 | 5387.8 (5248.3, 5649.9) |
|  | 2 | Ischemic heart disease | 3518.7 (3210.3, 3790.3) |  | Ischemic heart disease | 3326.5 (2831.6, 3850.5) |
|  | 3 | Stroke | 2881.4 (2654.0, 3126.2) |  | Stroke | 2761.8 (2384.5, 3156.6) |
|  | 4 | Neonatal disorders | 2830.5 (2473.3, 3227.6) |  | Neonatal disorders | 1956.1 (1609.2, 2368.2) |
|  | 5 | Diarrheal diseases | 2210.8 (1660.5, 2938.9) |  | Lower respiratory infections | 1822.1 (1550.5, 2087.5) |
| Poland | 1 | Ischemic heart disease | 5481.8 (5302.9, 5596.0) |  | COVID-19 | 2716.3 (1956.9, 3574.6) |
|  | 2 | Stroke | 3095.9 (2966.0, 3206.9) |  | Ischemic heart disease | 1991.5 (1811.4, 2148.1) |
|  | 3 | Neonatal disorders | 1722.2 (1645.8, 1794.9) |  | Low back pain | 1426.8 (1014.5, 1919.4) |
|  | 4 | Low back pain | 1466.0 (1048.5, 1969.5) |  | Stroke | 1118.6 (1019.2, 1224.2) |
|  | 5 | Road injuries | 1405.0 (1337.4, 1485.5) |  | Tracheal, bronchus, and lung cancer | 977.7 (888.6, 1062.9) |
| Portugal | 1 | Stroke | 3711.0 (3504.5, 3850.4) |  | Anxiety disorders | 1156.8 (692.9, 1723.8) |
|  | 2 | Ischemic heart disease | 2112.6 (2014.4, 2179.8) |  | Low back pain | 1144.6 (814.2, 1538.1) |
|  | 3 | Road injuries | 2018.8 (1912.1, 2148.3) |  | Depressive disorders | 1054.5 (671.7, 1517.4) |
|  | 4 | Neonatal disorders | 1213.6 (1138.3, 1283.5) |  | COVID-19 | 1019.3 (936.1, 1169.6) |
|  | 5 | Low back pain | 1165.2 (835.5, 1551.7) |  | Stroke | 763.6 (694.0, 819.2) |
| Qatar | 1 | Ischemic heart disease | 7030.8 (6060.8, 8075.7) |  | Diabetes mellitus | 2558.7 (2063.2, 3174.4) |
|  | 2 | Stroke | 2938.0 (2524.7, 3386.1) |  | Ischemic heart disease | 2139.5 (1690.4, 2627.2) |
|  | 3 | Road injuries | 2583.2 (2227.9, 2991.4) |  | COVID-19 | 1662.7 (1231.7, 2078.6) |
|  | 4 | Diabetes mellitus | 2173.9 (1824.3, 2604.1) |  | Stroke | 1004.2 (818.8, 1201.4) |
|  | 5 | Neonatal disorders | 1481.7 (1236.5, 1757.7) |  | Road injuries | 993.5 (815.8, 1208.6) |
| Republic of Korea | 1 | Stroke | 4649.0 (4269.3, 4967.6) |  | Low back pain | 1020.9 (729.0, 1368.3) |
|  | 2 | Road injuries | 2217.8 (2065.5, 2392.2) |  | Diabetes mellitus | 970.9 (724.7, 1284.8) |
|  | 3 | Cirrhosis and other chronic liver diseases | 1533.0 (1225.8, 1661.8) |  | Stroke | 859.6 (754.7, 961.1) |
|  | 4 | Stomach cancer | 1435.4 (1091.5, 1613.8) |  | Self-harm | 764.8 (498.4, 827.7) |
|  | 5 | Low back pain | 1095.8 (796.8, 1464.3) |  | Other musculoskeletal disorders | 686.1 (480.9, 950.3) |
| Republic of Moldova | 1 | Ischemic heart disease | 6803.8 (6546.1, 7009.0) |  | Ischemic heart disease | 4379.7 (4010.9, 4794.8) |
|  | 2 | Stroke | 3551.0 (3379.6, 3709.2) |  | COVID-19 | 3347.9 (3078.2, 3867.5) |
|  | 3 | Cirrhosis and other chronic liver diseases | 2187.5 (2121.7, 2265.6) |  | Stroke | 1888.9 (1717.3, 2085.9) |
|  | 4 | Neonatal disorders | 2044.8 (1817.5, 2297.3) |  | Cirrhosis and other chronic liver diseases | 1584.6 (1421.0, 1760.7) |
|  | 5 | Road injuries | 1833.9 (1752.5, 1931.1) |  | Low back pain | 1191.9 (855.6, 1607.4) |
| Romania | 1 | Ischemic heart disease | 4916.9 (4742.3, 5071.4) |  | COVID-19 | 3472.5 (2145.3, 4978.7) |
|  | 2 | Stroke | 4274.7 (4067.0, 4462.0) |  | Ischemic heart disease | 2838.0 (2575.4, 3113.3) |
|  | 3 | Lower respiratory infections | 2779.3 (2641.2, 2902.1) |  | Stroke | 2361.2 (2109.0, 2619.1) |
|  | 4 | Neonatal disorders | 1538.2 (1378.1, 1701.5) |  | Low back pain | 1453.0 (1034.9, 1959.2) |
|  | 5 | Low back pain | 1529.1 (1085.4, 2050.8) |  | Cirrhosis and other chronic liver diseases | 988.3 (872.2, 1093.4) |
| Russian Federation | 1 | Ischemic heart disease | 5901.5 (5703.6, 6010.8) |  | COVID-19 | 5281.2 (4297.2, 6306.0) |
|  | 2 | Stroke | 4218.6 (4056.5, 4325.3) |  | Ischemic heart disease | 4082.2 (3760.5, 4389.1) |
|  | 3 | Neonatal disorders | 1699.2 (1610.8, 1796.3) |  | Stroke | 2519.6 (2335.2, 2710.0) |
|  | 4 | Road injuries | 1672.8 (1603.3, 1756.1) |  | Low back pain | 1206.3 (866.7, 1623.3) |
|  | 5 | Self-harm | 1295.3 (1276.9, 1315.1) |  | Cardiomyopathy and myocarditis | 980.9 (888.4, 1065.5) |
| Rwanda | 1 | Lower respiratory infections | 9344.8 (7211.4, 11482.8) |  | COVID-19 | 5970.5 (4939.4, 6631.4) |
|  | 2 | Tuberculosis | 8443.6 (6000.3, 10681.7) |  | Neonatal disorders | 3151.8 (2517.5, 3897.6) |
|  | 3 | Stroke | 6937.6 (5677.3, 8350.6) |  | Stroke | 2684.0 (2008.7, 3385.3) |
|  | 4 | Malaria | 6772.4 (3199.9, 13775.0) |  | Lower respiratory infections | 2049.6 (1598.4, 2579.9) |
|  | 5 | Neonatal disorders | 6729.2 (5835.5, 7650.9) |  | HIV/AIDS | 1960.7 (1590.4, 2456.8) |
| Samoa | 1 | Ischemic heart disease | 4467.1 (3693.9, 5231.2) |  | Ischemic heart disease | 4993.9 (4206.3, 6087.2) |
|  | 2 | Stroke | 4381.2 (3655.7, 5049.0) |  | Stroke | 3473.3 (2910.0, 4119.9) |
|  | 3 | Diabetes mellitus | 2373.2 (1983.7, 2828.3) |  | Diabetes mellitus | 3409.2 (2829.9, 4120.0) |
|  | 4 | Lower respiratory infections | 2360.6 (1924.2, 2846.9) |  | Chronic obstructive pulmonary disease | 1396.4 (1123.6, 1732.6) |
|  | 5 | Neonatal disorders | 2218.6 (1761.5, 2804.1) |  | Lower respiratory infections | 1345.0 (1099.1, 1650.2) |
| Sao Tome and Principe | 1 | Lower respiratory infections | 6216.1 (5267.3, 7139.5) |  | Stroke | 2906.3 (2491.8, 3469.5) |
|  | 2 | Diarrheal diseases | 4993.0 (3874.2, 6143.4) |  | Lower respiratory infections | 2325.7 (1905.4, 2810.3) |
|  | 3 | Neonatal disorders | 3348.8 (2915.6, 3848.5) |  | COVID-19 | 2170.3 (1923.4, 2437.0) |
|  | 4 | Stroke | 3056.0 (2667.8, 3425.3) |  | Ischemic heart disease | 2006.4 (1715.2, 2352.6) |
|  | 5 | Measles | 2876.6 (1080.4, 5632.9) |  | Chronic kidney disease | 1636.6 (1240.0, 1979.7) |
| Saudi Arabia | 1 | Ischemic heart disease | 4888.4 (3749.1, 6144.0) |  | Ischemic heart disease | 4219.8 (3530.6, 5082.9) |
|  | 2 | Neonatal disorders | 3680.5 (2987.6, 4523.4) |  | Road injuries | 2196.9 (1789.5, 2696.2) |
|  | 3 | Stroke | 3342.4 (2659.9, 4148.0) |  | Stroke | 2023.3 (1687.1, 2452.5) |
|  | 4 | Road injuries | 3034.9 (2393.3, 3717.6) |  | Chronic kidney disease | 1762.8 (1351.1, 2169.6) |
|  | 5 | Congenital birth defects | 2167.8 (1358.8, 2899.7) |  | Diabetes mellitus | 1449.3 (1169.9, 1831.3) |
| Senegal | 1 | Diarrheal diseases | 8727.7 (6514.1, 10892.2) |  | COVID-19 | 5453.1 (4576.6, 6292.7) |
|  | 2 | Neonatal disorders | 6090.3 (5304.6, 6904.6) |  | Neonatal disorders | 3646.6 (3015.1, 4367.6) |
|  | 3 | Lower respiratory infections | 5348.1 (4467.9, 6225.8) |  | Stroke | 3134.8 (2522.8, 3830.5) |
|  | 4 | Malaria | 3949.1 (1189.3, 9775.3) |  | Ischemic heart disease | 2346.8 (1836.0, 2939.9) |
|  | 5 | Stroke | 3923.1 (3381.5, 4551.9) |  | Lower respiratory infections | 1862.8 (1498.0, 2269.3) |
| Serbia | 1 | Stroke | 5409.5 (4919.2, 5911.8) |  | Ischemic heart disease | 2952.0 (2558.3, 3364.1) |
|  | 2 | Ischemic heart disease | 5206.7 (4785.5, 5596.7) |  | COVID-19 | 2723.5 (1288.9, 4291.0) |
|  | 3 | Neonatal disorders | 3227.1 (2981.4, 3525.5) |  | Stroke | 2695.7 (2279.5, 3134.5) |
|  | 4 | Low back pain | 1442.9 (1043.9, 1926.0) |  | Low back pain | 1443.1 (1031.7, 1935.5) |
|  | 5 | Congenital birth defects | 1292.4 (908.3, 1542.0) |  | Tracheal, bronchus, and lung cancer | 1077.6 (846.4, 1332.4) |
| Seychelles | 1 | Stroke | 3060.0 (2826.6, 3309.4) |  | COVID-19 | 3193.1 (2839.1, 3620.5) |
|  | 2 | Ischemic heart disease | 3012.4 (2786.3, 3250.4) |  | Ischemic heart disease | 1807.8 (1629.1, 2020.4) |
|  | 3 | Lower respiratory infections | 2183.9 (1997.6, 2394.9) |  | Stroke | 1765.4 (1581.7, 1955.8) |
|  | 4 | Hypertensive heart disease | 1706.2 (1455.3, 1901.3) |  | Diabetes mellitus | 1521.6 (1198.6, 1925.9) |
|  | 5 | Neonatal disorders | 1587.1 (1423.6, 1778.6) |  | Lower respiratory infections | 1334.5 (1170.5, 1488.3) |
| Sierra Leone | 1 | Malaria | 11916.2 (4685.9, 21469.6) |  | Malaria | 8940.3 (3029.8, 17358.5) |
|  | 2 | Neonatal disorders | 10049.4(8738.8, 11476.3) |  | Neonatal disorders | 6485.9 (5095.9, 8064.5) |
|  | 3 | Lower respiratory infections | 9813.5 (7510.8, 12416.6) |  | Stroke | 3477.3 (2748.7, 4401.7) |
|  | 4 | Diarrheal diseases | 8651.4 (5630.2, 11911.5) |  | Lower respiratory infections | 3019.0 (2250.0, 3972.9) |
|  | 5 | Measles | 5614.7 (1952.7, 11333.9) |  | COVID-19 | 2953.0 (2470.2, 3533.1) |
| Singapore | 1 | Ischemic heart disease | 3172.8 (3053.1, 3258.4) |  | Ischemic heart disease | 934.1 (866.5, 985.5) |
|  | 2 | Stroke | 2103.7 (1984.5, 2223.2) |  | Low back pain | 847.1 (603.3, 1134.9) |
|  | 3 | Lower respiratory infections | 1528.6 (1435.1, 1601.7) |  | Other musculoskeletal disorders | 728.3 (499.8, 1005.8) |
|  | 4 | Low back pain | 916.3 (656.0, 1223.2) |  | Diabetes mellitus | 660.5 (454.1, 928.9) |
|  | 5 | Congenital birth defects | 862.8 (788.6, 931.3) |  | Lower respiratory infections | 572.7 (505.9, 617.6) |
| Slovakia | 1 | Ischemic heart disease | 6333.3 (6065.6, 6585.2) |  | COVID-19 | 3940.6 (2806.0, 5147.3) |
|  | 2 | Stroke | 2973.1 (2700.1, 3268.5) |  | Ischemic heart disease | 3080.7 (2709.5, 3443.4) |
|  | 3 | Low back pain | 1484.2 (1062.3, 1986.0) |  | Low back pain | 1421.0 (1015.8, 1920.8) |
|  | 4 | Neonatal disorders | 1472.7 (1324.6, 1626.9) |  | Stroke | 1345.6 (1150.5, 1564.9) |
|  | 5 | Lower respiratory infections | 1380.9 (1250.2, 1505.9) |  | Falls | 784.9 (608.3, 1002.8) |
| Slovenia | 1 | Ischemic heart disease | 2452.1 (2323.7, 2565.5) |  | COVID-19 | 1600.6 (1011.5, 2286.9) |
|  | 2 | Stroke | 2094.7 (1961.0, 2222.0) |  | Low back pain | 1302.0 (925.2, 1744.6) |
|  | 3 | Low back pain | 1322.4 (943.0, 1757.6) |  | Falls | 828.4 (642.2, 1052.7) |
|  | 4 | Road injuries | 1232.7 (1155.2, 1318.6) |  | Ischemic heart disease | 822.8 (715.7, 920.5) |
|  | 5 | Falls | 1180.4 (968.4, 1428.3) |  | Tracheal, bronchus, and lung cancer | 644.7 (545.9, 747.5) |
| Solomon Islands | 1 | Ischemic heart disease | 6645.5 (4883.2, 8426.4) |  | Ischemic heart disease | 6228.5 (5006.1, 7901.1) |
|  | 2 | Lower respiratory infections | 6033.0 (4542.2, 7401.3) |  | Stroke | 5159.0 (4239.6, 6353.5) |
|  | 3 | Stroke | 5869.3 (4343.6, 7146.3) |  | Diabetes mellitus | 3522.8 (2844.4, 4307.7) |
|  | 4 | Malaria | 5252.5 (749.8, 16592.5) |  | Lower respiratory infections | 3436.8 (2832.8, 4091.4) |
|  | 5 | Diabetes mellitus | 2436.1 (1729.7, 3293.7) |  | Malaria | 1432.1 (870.7, 2482.4) |
| Somalia | 1 | Diarrheal diseases | 11307.6 (7302.8, 15980.1) |  | COVID-19 | 12874.9 (9306.3, 17277.2) |
|  | 2 | Tuberculosis | 9734.8 (6130.3, 16044.1) |  | Tuberculosis | 7691.2 (4664.6, 13131.8) |
|  | 3 | Lower respiratory infections | 8542.4 (6576.0, 10920.7) |  | Diarrheal diseases | 4889.4 (3087.2, 7195.7) |
|  | 4 | Measles | 7831.5 (2779.5, 14272.5) |  | Neonatal disorders | 4855.0 (3897.9, 6067.0) |
|  | 5 | Neonatal disorders | 5465.9 (4294.7, 6691.4) |  | Lower respiratory infections | 4227.0 (3236.1, 5506.1) |
| South Africa | 1 | Diarrheal diseases | 4825.8 (4125.7, 5868.5) |  | HIV/AIDS | 9363.4 (8783.3, 10017.2) |
|  | 2 | Neonatal disorders | 4461.2 (4022.8, 4952.8) |  | COVID-19 | 8401.2 (8229.3, 8773.8) |
|  | 3 | Lower respiratory infections | 4347.8 (3923.1, 4920.7) |  | Neonatal disorders | 3409.5 (2844.8, 4126.6) |
|  | 4 | Tuberculosis | 3420.5 (2935.5, 4192.0) |  | Diabetes mellitus | 2194.5 (1993.3, 2419.6) |
|  | 5 | Interpersonal violence | 3409.7 (3166.0, 3624.9) |  | Lower respiratory infections | 2156.3 (1947.3, 2401.2) |
| South Sudan | 1 | Diarrheal diseases | 11446.7 (7752.6, 15702.2) |  | Diarrheal diseases | 7069.8 (4651.7, 10778.4) |
|  | 2 | Lower respiratory infections | 7964.4 (6433.9, 10107.4) |  | Neonatal disorders | 6676.2 (5070.8, 8627.6) |
|  | 3 | Neonatal disorders | 6073.6 (5049.6, 7170.8) |  | COVID-19 | 6009.5 (4369.6, 7724.1) |
|  | 4 | Tuberculosis | 5623.5 (3905.0, 8433.7) |  | Lower respiratory infections | 4503.1 (3379.0, 6514.5) |
|  | 5 | Protein-energy malnutrition | 5053.4 (3656.9, 7167.1) |  | Malaria | 4423.0 (1868.7, 8462.2) |
| Sri Lanka | 1 | Stroke | 3270.7 (3000.6, 3558.0) |  | Stroke | 1992.1 (1450.5, 2562.0) |
|  | 2 | Ischemic heart disease | 2865.4 (2621.9, 3142.0) |  | Ischemic heart disease | 1963.6 (1330.0, 2662.4) |
|  | 3 | Conflict and terrorism | 2274.2 (2144.0, 2434.0) |  | Diabetes mellitus | 1956.2 (1518.6, 2440.7) |
|  | 4 | Neonatal disorders | 2271.0 (1982.4, 2539.8) |  | COVID-19 | 1611.3 (1378.3, 2882.8) |
|  | 5 | Self-harm | 1915.3 (1713.9, 2092.9) |  | Neonatal disorders | 898.7 (736.7, 1093.7) |
| Sudan | 1 | Ischemic heart disease | 7582.5 (6084.0, 9429.2) |  | Ischemic heart disease | 5338.3 (4083.7, 7066.9) |
|  | 2 | Neonatal disorders | 7246.4 (6016.4, 8801.4) |  | COVID-19 | 4270.3 (2562.4, 6241.7) |
|  | 3 | Lower respiratory infections | 5100.1 (3948.6, 6830.9) |  | Neonatal disorders | 2989.3 (2416.3, 3719.4) |
|  | 4 | Congenital birth defects | 4919.3 (1729.8, 6669.1) |  | Stroke | 2532.7 (1919.8, 3220.1) |
|  | 5 | Stroke | 4696.0 (3765.5, 5644.6) |  | Congenital birth defects | 1964.3 (1433.0, 2532.4) |
| Suriname | 1 | Neonatal disorders | 4728.0 (4172.4, 5346.4) |  | COVID-19 | 4224.0 (4004.8, 5555.6) |
|  | 2 | Ischemic heart disease | 3737.5 (3432.5, 3989.9) |  | Neonatal disorders | 2780.1 (2159.2, 3558.6) |
|  | 3 | Stroke | 2908.0 (2640.6, 3135.7) |  | Diabetes mellitus | 2143.6 (1726.1, 2674.3) |
|  | 4 | Diabetes mellitus | 1500.0 (1297.0, 1724.9) |  | Stroke | 2063.3 (1650.2, 2507.1) |
|  | 5 | Diarrheal diseases | 1499.6 (1106.3, 1892.8) |  | Ischemic heart disease | 2036.8 (1594.7, 2533.6) |
| Syrian Arab Republic | 1 | Ischemic heart disease | 7886.6 (6446.1, 9443.0) |  | Ischemic heart disease | 6688.8 (5230.4, 8518.9) |
|  | 2 | Stroke | 3546.3 (2899.4, 4294.7) |  | COVID-19 | 2389.7 (1613.1, 3179.4) |
|  | 3 | Neonatal disorders | 3419.5 (2926.0, 4022.9) |  | Stroke | 2124.4 (1670.8, 2652.8) |
|  | 4 | Congenital birth defects | 1972.7 (1177.6, 2545.1) |  | Conflict and terrorism | 1504.4 (1265.3, 1825.2) |
|  | 5 | Lower respiratory infections | 1441.9 (1146.4, 1830.6) |  | Diabetes mellitus | 1089.8 (847.2, 1420.6) |
| Tajikistan | 1 | Lower respiratory infections | 6970.1 (6186.9, 7786.0) |  | COVID-19 | 5808.0 (4447.8, 7180.4) |
|  | 2 | Ischemic heart disease | 5960.0 (5301.7, 6541.1) |  | Ischemic heart disease | 4400.4 (3740.1, 5106.8) |
|  | 3 | Stroke | 3524.0 (3099.9, 3970.4) |  | Stroke | 2614.5 (2123.2, 3122.5) |
|  | 4 | Diarrheal diseases | 3293.2 (2672.6, 4028.8) |  | Neonatal disorders | 2354.5 (1922.8, 2866.3) |
|  | 5 | Neonatal disorders | 2625.1 (2287.7, 2959.4) |  | Lower respiratory infections | 2136.5 (1603.0, 2757.2) |
| Thailand | 1 | Stroke | 2751.6 (2424.3, 3080.5) |  | Road injuries | 1697.3 (1383.3, 2077.5) |
|  | 2 | Neonatal disorders | 2272.5 (1912.4, 2708.7) |  | Stroke | 1636.0 (1316.7, 1984.0) |
|  | 3 | Road injuries | 2181.1 (1950.7, 2427.5) |  | Ischemic heart disease | 1039.7 (825.6, 1278.9) |
|  | 4 | Ischemic heart disease | 1685.4 (1453.3, 1952.6) |  | Diabetes mellitus | 1018.3 (817.0, 1261.8) |
|  | 5 | Diarrheal diseases | 1270.8 (512.5, 2863.7) |  | COVID-19 | 920.9 (481.5, 1768.3) |
| Timor-Leste | 1 | Lower respiratory infections | 8502.5 (7148.2, 10309.7) |  | Stroke | 3749.5 (2901.8, 4710.0) |
|  | 2 | Diarrheal diseases | 7015.5 (4492.1, 10286.7) |  | Ischemic heart disease | 3163.0 (2433.5, 4007.9) |
|  | 3 | Tuberculosis | 5642.8 (3894.5, 7962.9) |  | Neonatal disorders | 2670.4 (2234.4, 3170.5) |
|  | 4 | Neonatal disorders | 4900.2 (4147.7, 5595.0) |  | COVID-19 | 2346.2 (863.9, 4474.8) |
|  | 5 | Stroke | 4256.5 (3528.9, 5091.7) |  | Tuberculosis | 2175.9 (1572.9, 3085.2) |
| Togo | 1 | Diarrheal diseases | 7996.7 (5381.8, 10877.1) |  | Malaria | 4325.4 (1605.3, 8067.0) |
|  | 2 | Malaria | 6563.5 (3317.1, 11325.4) |  | COVID-19 | 4200.9 (3104.7, 4926.3) |
|  | 3 | Neonatal disorders | 6436.1 (5794.1, 7014.3) |  | Neonatal disorders | 3963.0 (3256.3, 4769.9) |
|  | 4 | Lower respiratory infections | 5937.0 (4927.7, 6948.1) |  | Stroke | 3580.6 (2763.8, 4446.1) |
|  | 5 | Stroke | 3961.3 (3417.4, 4583.8) |  | Lower respiratory infections | 3107.1 (2406.8, 3858.5) |
| Tonga | 1 | Ischemic heart disease | 3121.1 (2608.9, 3637.3) |  | Diabetes mellitus | 3650.4 (3006.0, 4497.7) |
|  | 2 | Diabetes mellitus | 2713.2 (2333.1, 3118.4) |  | Ischemic heart disease | 3065.3 (2467.3, 3781.7) |
|  | 3 | Stroke | 2119.3 (1752.1, 2472.2) |  | Stroke | 1751.0 (1415.6, 2118.6) |
|  | 4 | Lower respiratory infections | 1977.1 (1693.0, 2294.6) |  | Lower respiratory infections | 1286.9 (991.5, 1614.5) |
|  | 5 | Neonatal disorders | 1762.0 (1495.5, 2043.5) |  | Neonatal disorders | 993.6 (791.7, 1242.4) |
| Trinidad and Tobago | 1 | Ischemic heart disease | 4701.9 (4550.0, 4842.6) |  | COVID-19 | 4011.2 (3311.2, 6280.3) |
|  | 2 | Diabetes mellitus | 3933.3 (3639.9, 4270.4) |  | Diabetes mellitus | 3487.0 (2848.1, 4287.3) |
|  | 3 | Neonatal disorders | 3118.4 (2672.2, 3583.6) |  | Ischemic heart disease | 2270.9 (1754.6, 2870.8) |
|  | 4 | Stroke | 2736.2 (2612.5, 2850.2) |  | Neonatal disorders | 1714.2 (1371.6, 2139.7) |
|  | 5 | Congenital birth defects | 1031.6 (900.3, 1176.2) |  | Interpersonal violence | 1547.0 (1238.7, 1899.8) |
| Tunisia | 1 | Neonatal disorders | 4192.1 (3519.4, 4920.0) |  | COVID-19 | 5006.4 (3987.5, 5999.9) |
|  | 2 | Ischemic heart disease | 4177.6 (3489.9, 4821.3) |  | Ischemic heart disease | 3036.7 (2267.1, 4065.1) |
|  | 3 | Congenital birth defects | 2590.0 (1389.9, 3269.7) |  | Stroke | 1508.2 (1095.6, 2036.5) |
|  | 4 | Stroke | 2353.3 (1923.5, 2839.3) |  | Depressive disorders | 1255.1 (800.1, 1861.4) |
|  | 5 | Road injuries | 1802.0 (1578.2, 2023.3) |  | Diabetes mellitus | 1119.5 (848.5, 1502.0) |
| Turkey | 1 | Neonatal disorders | 4993.8 (4375.5, 5749.6) |  | COVID-19 | 2997.2 (2313.0, 3618.8) |
|  | 2 | Ischemic heart disease | 4255.1 (3773.1, 4724.6) |  | Ischemic heart disease | 2419.3 (2008.1, 2857.0) |
|  | 3 | Congenital birth defects | 3513.9 (1642.4, 4525.0) |  | Stroke | 1343.0 (1130.4, 1571.1) |
|  | 4 | Stroke | 3116.2 (2686.9, 3602.1) |  | Diabetes mellitus | 1078.5 (875.1, 1322.8) |
|  | 5 | Lower respiratory infections | 2664.0 (2103.1, 3443.2) |  | Neonatal disorders | 1070.2 (907.2, 1276.4) |
| Uganda | 1 | HIV/AIDS | 26004.4 (16548.9, 36077.8) |  | COVID-19 | 7239.4 (4680.4, 8845.4) |
|  | 2 | Malaria | 11032.6 (6908.0, 17725.4) |  | Malaria | 6008.6 (2044.8, 11064.8) |
|  | 3 | Diarrheal diseases | 6251.9 (3296.8, 9904.6) |  | HIV/AIDS | 4620.2 (3940.8, 5744.0) |
|  | 4 | Neonatal disorders | 6024.6 (5272.0, 6843.9) |  | Neonatal disorders | 3950.9 (3208.8, 4820.0) |
|  | 5 | Lower respiratory infections | 4369.7 (3439.2, 5992.9) |  | Stroke | 2241.3 (1767.7, 2826.8) |
| Ukraine | 1 | Ischemic heart disease | 5894.2 (5605.3, 6105.8) |  | Ischemic heart disease | 6522.7 (5003.4, 8125.7) |
|  | 2 | Stroke | 3503.7 (3323.4, 3644.7) |  | COVID-19 | 3323.7 (2679.7, 4191.1) |
|  | 3 | Road injuries | 1589.0 (1511.7, 1678.2) |  | Stroke | 2153.0 (1674.5, 2670.4) |
|  | 4 | Neonatal disorders | 1497.1 (1320.1, 1672.5) |  | Low back pain | 1372.7 (983.1, 1829.6) |
|  | 5 | Low back pain | 1431.5 (1018.3, 1915.1) |  | Cirrhosis and other chronic liver diseases | 935.0 (668.6, 1228.9) |
| United Arab Emirates | 1 | Ischemic heart disease | 5579.1 (4474.0, 6897.4) |  | Ischemic heart disease | 2923.7 (2395.6, 3422.0) |
|  | 2 | Stroke | 2921.9 (2382.7, 3506.8) |  | COVID-19 | 1695.6 (829.8, 2322.9) |
|  | 3 | Road injuries | 2348.9 (1942.2, 2860.3) |  | Stroke | 1648.8 (1364.3, 1951.6) |
|  | 4 | Neonatal disorders | 1660.2 (1387.6, 1970.5) |  | Diabetes mellitus | 1502.6 (1203.3, 1843.2) |
|  | 5 | Congenital birth defects | 1390.9 (1017.6, 1764.2) |  | Road injuries | 1037.8 (869.4, 1229.5) |
| United Republic of Tanzania | 1 | Lower respiratory infections | 8183.7 (6729.4, 9727.8) |  | COVID-19 | 6498.0 (5771.9, 7111.5) |
|  | 2 | HIV/AIDS | 6968.4 (4739.3, 10676.9) |  | HIV/AIDS | 4154.5 (3478.0, 5072.4) |
|  | 3 | Diarrheal diseases | 6782.0 (4915.7, 8844.5) |  | Neonatal disorders | 3514.5 (2727.2, 4545.4) |
|  | 4 | Malaria | 6438.2 (3341.3, 10917.9) |  | Lower respiratory infections | 2356.9 (1945.3, 2824.6) |
|  | 5 | Neonatal disorders | 4655.1 (4068.4, 5289.7) |  | Stroke | 2284.8 (1809.0, 2877.0) |
| Uruguay | 1 | Ischemic heart disease | 2929.7 (2799.4, 3017.3) |  | COVID-19 | 2309.2 (2235.5, 2464.6) |
|  | 2 | Stroke | 2512.7 (2386.5, 2612.4) |  | Ischemic heart disease | 1196.7 (1130.2, 1250.9) |
|  | 3 | Neonatal disorders | 1939.9 (1818.1, 2063.6) |  | Stroke | 1110.3 (1032.6, 1185.4) |
|  | 4 | Tracheal, bronchus, and lung cancer | 1097.7 (1025.1, 1169.7) |  | Low back pain | 1106.9 (790.6, 1483.1) |
|  | 5 | Low back pain | 1029.0 (740.2, 1370.7) |  | Other musculoskeletal disorders | 881.9 (629.9, 1188.8) |
| Uzbekistan | 1 | Ischemic heart disease | 6122.2 (5815.2, 6372.1) |  | Ischemic heart disease | 6218.8 (5449.8, 7058.1) |
|  | 2 | Lower respiratory infections | 4903.2 (4545.0, 5308.4) |  | Stroke | 2302.2 (2012.6, 2639.8) |
|  | 3 | Stroke | 3199.0 (3025.8, 3362.6) |  | Neonatal disorders | 1587.7 (1322.5, 1880.6) |
|  | 4 | Neonatal disorders | 2223.1 (1965.4, 2534.1) |  | COVID-19 | 1564.4 (413.8, 2426.3) |
|  | 5 | Road injuries | 1213.5 (1155.0, 1272.1) |  | Lower respiratory infections | 1407.7 (1191.6, 1640.7) |
| Vanuatu | 1 | Ischemic heart disease | 7311.5 (5948.6, 9023.9) |  | Ischemic heart disease | 7189.7 (6037.2, 8411.3) |
|  | 2 | Stroke | 5382.9 (4397.4, 6649.6) |  | Stroke | 4387.3 (3603.9, 5210.2) |
|  | 3 | Lower respiratory infections | 2759.3 (2221.0, 3322.3) |  | Diabetes mellitus | 3039.8 (2541.7, 3648.0) |
|  | 4 | Malaria | 2685.3 (1120.3, 5488.4) |  | COVID-19 | 2400.9 (559.5, 4837.5) |
|  | 5 | Neonatal disorders | 2457.7 (2027.5, 3051.4) |  | Neonatal disorders | 1719.0 (1367.4, 2157.9) |
| Venezuela  (Bolivarian Republic of) | 1 | Ischemic heart disease | 3553.6 (3419.4, 3653.1) |  | COVID-19 | 4953.8 (4616.7, 5424.6) |
|  | 2 | Neonatal disorders | 2678.6 (2521.3, 2835.5) |  | Interpersonal violence | 2999.9 (2388.7, 3618.7) |
|  | 3 | Stroke | 1680.7 (1609.3, 1747.1) |  | Ischemic heart disease | 2941.8 (2286.7, 3718.3) |
|  | 4 | Road injuries | 1651.2 (1584.1, 1729.9) |  | Neonatal disorders | 1972.8 (1524.5, 2452.7) |
|  | 5 | Diarrheal diseases | 1369.4 (1294.7, 1450.0) |  | Diabetes mellitus | 1582.0 (1293.8, 1951.6) |
| Viet Nam | 1 | Stroke | 4783.4 (3893.3, 5960.8) |  | Stroke | 3952.5 (3287.4, 4604.9) |
|  | 2 | Neonatal disorders | 3188.1 (2827.1, 3585.1) |  | Ischemic heart disease | 1464.6 (1198.7, 1740.5) |
|  | 3 | Tuberculosis | 2361.4 (1795.6, 3035.4) |  | Neonatal disorders | 1127.5 (939.7, 1372.6) |
|  | 4 | Lower respiratory infections | 2205.9 (1873.3, 2585.3) |  | Diabetes mellitus | 1126.1 (925.2, 1367.0) |
|  | 5 | Road injuries | 1412.1 (1158.1, 1738.8) |  | Road injuries | 1055.5 (850.6, 1277.7) |
| Yemen | 1 | Ischemic heart disease | 7122.8 (5449.9, 9124.4) |  | Ischemic heart disease | 5442.6 (4100.2, 7104.9) |
|  | 2 | Neonatal disorders | 6837.3 (5736.8, 8217.7) |  | COVID-19 | 4618.9 (2074.5, 7157.5) |
|  | 3 | Stroke | 4871.8 (3687.4, 6173.2) |  | Conflict and terrorism | 3468.0 (3142.4, 3846.0) |
|  | 4 | Diarrheal diseases | 4816.1 (3235.0, 6658.7) |  | Neonatal disorders | 3395.8 (2800.5, 4192.7) |
|  | 5 | Congenital birth defects | 4246.9 (1533.3, 5845.4) |  | Stroke | 3331.1 (2501.0, 4441.5) |
| Zambia | 1 | HIV/AIDS | 10937.8 (6889.2, 17988.9) |  | COVID-19 | 13297.2 (9805.4, 16731.9) |
|  | 2 | Diarrheal diseases | 8634.2 (6082.3, 11415.1) |  | HIV/AIDS | 7803.2 (6706.4, 9083.7) |
|  | 3 | Lower respiratory infections | 7660.6 (6410.8, 9327.8) |  | Stroke | 3570.4 (2750.1, 4575.9) |
|  | 4 | Tuberculosis | 6507.3 (5019.1, 7932.5) |  | Neonatal disorders | 3163.2 (2447.9, 4098.2) |
|  | 5 | Malaria | 5220.3 (2872.1, 9046.4) |  | Lower respiratory infections | 2403.7 (1879.6, 2973.0) |
| Zimbabwe | 1 | HIV/AIDS | 13503.4 (9250.0, 19855.5) |  | COVID-19 | 16474.9 (12797.2, 19351.8) |
|  | 2 | Lower respiratory infections | 4487.8 (3978.6, 5036.3) |  | HIV/AIDS | 8735.4 (7512.4, 10194.9) |
|  | 3 | Neonatal disorders | 3382.5 (3013.0, 3754.0) |  | Lower respiratory infections | 4491.0 (3528.8, 5513.1) |
|  | 4 | Tuberculosis | 3304.7 (2717.7, 3891.4) |  | Tuberculosis | 4427.7 (2879.6, 5875.1) |
|  | 5 | Diarrheal diseases | 2454.6 (1745.3, 3122.6) |  | Neonatal disorders | 3944.5 (3308.5, 4674.0) |

| Supplementary Table 9: Maternal mortality ratio (/100,000 persons) in 149 BRI Countries between 1990 and 2021 | | | | |
| --- | --- | --- | --- | --- |
| Country |  | 1990 | 2021 | EAPC during 1990-2021 (95% CI) (%) |
| Afghanistan |  | 522.5 (340.0 to 750.9) | 304.8 (177.8 to 470.5) | -1.15 (-1.41 to -0.90) |
| Albania |  | 27.7 (19.4 to 38.3) | 6.5 (3.9 to 10.5) | -3.86 (-4.14 to -3.58) |
| Algeria |  | 161.9 (104.9 to 224.8) | 64.4 (38.9 to 97.5) | -3.32 (-3.62 to -3.02) |
| Angola |  | 464.6 (284.6 to 686.4) | 184.2 (108.0 to 288.6) | -3.43 (-3.65 to -3.21) |
| Antigua and Barbuda |  | 30.2 (25.1 to 35.9) | 41.3 (34.9 to 49.1) | 2.60 (1.98 to 3.23) |
| Argentina |  | 65.8 (52.5 to 81.2) | 45.2 (34.4 to 58.8) | -0.76 (-1.00 to -0.53) |
| Armenia |  | 32.6 (26.7 to 39.3) | 11.4 (8.8 to 14.5) | -3.40 (-3.93 to -2.87) |
| Austria |  | 6.5 (5.5 to 7.8) | 2.6 (2.2 to 3.1) | -1.96 (-2.38 to -1.55) |
| Azerbaijan |  | 37.1 (28.9 to 47.3) | 16.2 (10.6 to 23.3) | -3.79 (-4.16 to -3.42) |
| Bahrain |  | 46.7 (30.6 to 64.9) | 35.2 (22.3 to 52.2) | -1.25 (-1.76 to -0.74) |
| Bangladesh |  | 504.3 (416.9 to 597.2) | 148.5 (101.8 to 204.8) | -4.02 (-4.40 to -3.64) |
| Barbados |  | 47.1 (38.7 to 55.6) | 59.5 (44.2 to 78.8) | 1.63 (1.11 to 2.16) |
| Belarus |  | 27.0 (22.8 to 31.6) | 9.1 (6.5 to 12.6) | -4.00 (-4.31 to -3.68) |
| Benin |  | 497.5 (359.3 to 661.9) | 228.2 (138.5 to 336.9) | -2.19 (-2.39 to -2.00) |
| Bolivia |  | 353.8 (267.2 to 457.5) | 201.6 (141.2 to 283.7) | -2.27 (-2.43 to -2.11) |
| Bosnia and Herzegovina |  | 20.7 (14.5 to 27.9) | 4.4 (2.7 to 6.5) | -6.06 (-6.72 to -5.40) |
| Botswana |  | 138.3 (80.7 to 217.6) | 78.3 (46.1 to 113.7) | -1.05 (-1.70 to -0.40) |
| Brunei Darussalam |  | 43.8 (25.5 to 67.4) | 21.5 (13.3 to 34.2) | -1.42 (-1.76 to -1.09) |
| Bulgaria |  | 30.1 (25.9 to 35.6) | 7.4 (5.6 to 9.4) | -4.83 (-5.23 to -4.42) |
| Burkina Faso |  | 311.4 (218.1 to 427.6) | 212.7 (143.5 to 304.8) | -1.34 (-1.45 to -1.24) |
| Burundi |  | 655.1 (420.2 to 913.9) | 285.2 (176.9 to 414.3) | -3.67 (-4.10 to -3.25) |
| Côte d'Ivoire |  | 324.0 (214.6 to 461.0) | 274.3 (158.9 to 422.1) | -0.41 (-0.80 to -0.02) |
| Cabo Verde |  | 124.0 (75.0 to 183.3) | 62.0 (34.5 to 102.4) | -2.34 (-2.60 to -2.07) |
| Cambodia |  | 361.6 (252.7 to 495.8) | 138.5 (85.6 to 222.2) | -4.17 (-4.72 to -3.62) |
| Cameroon |  | 396.1 (284.9 to 529.7) | 295.1 (185.4 to 411.3) | -0.97 (-1.27 to -0.66) |
| Central African Republic |  | 647.9 (457.9 to 836.1) | 503.5 (300.1 to 783.1) | -0.74 (-0.96 to -0.52) |
| Chad |  | 522.0 (377.6 to 672.7) | 452.3 (298.0 to 649.9) | -0.48 (-0.79 to -0.16) |
| Chile |  | 43.8 (38.5 to 49.9) | 23.7 (19.3 to 28.7) | -1.42 (-1.78 to -1.06) |
| China |  | 68.0 (46.3 to 92.7) | 14.1 (9.9 to 19.5) | -5.71 (-6.24 to -5.17) |
| Comoros |  | 266.7 (128.7 to 402.1) | 190.0 (112.0 to 304.3) | -1.95 (-2.50 to -1.39) |
| Congo |  | 430.4 (268.9 to 610.5) | 347.9 (205.3 to 549.9) | -0.93 (-1.28 to -0.59) |
| Cook Islands |  | 5.2 (2.6 to 9.5) | 3.9 (2.1 to 6.6) | 0.00 (-0.34 to 0.35) |
| Costa Rica |  | 30.1 (26.0 to 34.9) | 27.4 (22.5 to 33.3) | -1.15 (-1.69 to -0.60) |
| Croatia |  | 14.4 (11.9 to 17.0) | 2.4 (1.8 to 3.0) | -4.91 (-5.45 to -4.38) |
| Cuba |  | 55.3 (48.3 to 64.1) | 46.4 (37.9 to 56.4) | -0.42 (-0.74 to -0.11) |
| Cyprus |  | 10.3 (5.6 to 16.2) | 3.7 (2.2 to 6.0) | -4.04 (-4.64 to -3.44) |
| Czechia |  | 13.1 (10.7 to 15.6) | 1.8 (1.4 to 2.3) | -6.17 (-6.58 to -5.76) |
| Democratic Republic of the Congo |  | 411.9 (271.7 to 564.0) | 409.2 (271.4 to 573.4) | 0.75 (0.31 to 1.19) |
| Djibouti |  | 691.9 (434.4 to 991.1) | 501.5 (260.8 to 841.3) | -1.16 (-1.39 to -0.92) |
| Dominica |  | 34.0 (24.7 to 45.6) | 89.9 (51.0 to 145.3) | 3.99 (3.69 to 4.30) |
| Dominican Republic |  | 91.5 (73.4 to 111.0) | 96.9 (69.8 to 130.2) | 1.38 (0.88 to 1.88) |
| Ecuador |  | 136.7 (120.1 to 154.4) | 81.8 (59.5 to 108.7) | -0.96 (-1.34 to -0.58) |
| Egypt |  | 150.6 (101.3 to 199.2) | 33.6 (21.7 to 48.3) | -5.02 (-5.52 to -4.52) |
| El Salvador |  | 192.2 (146.6 to 235.2) | 42.7 (29.4 to 59.3) | -3.72 (-5.07 to -2.34) |
| Equatorial Guinea |  | 597.5 (325.8 to 931.6) | 231.6 (103.8 to 431.4) | -3.16 (-3.59 to -2.73) |
| Eritrea |  | 868.3 (632.0 to 1116.9) | 425.0 (236.2 to 690.1) | -2.00 (-2.16 to -1.84) |
| Estonia |  | 34.4 (29.2 to 41.0) | 3.2 (2.5 to 3.8) | -8.23 (-8.67 to -7.79) |
| Ethiopia |  | 744.9 (589.6 to 884.9) | 218.4 (148.5 to 300.9) | -4.44 (-4.75 to -4.12) |
| Fiji |  | 114.9 (64.2 to 181.0) | 98.6 (54.4 to 162.8) | -1.07 (-1.41 to -0.72) |
| Gabon |  | 299.8 (211.8 to 411.7) | 185.6 (98.4 to 316.3) | -1.91 (-2.32 to -1.49) |
| Gambia |  | 498.0 (312.1 to 718.9) | 407.5 (244.4 to 629.1) | -0.74 (-0.97 to -0.50) |
| Georgia |  | 48.0 (40.2 to 56.9) | 20.3 (16.3 to 24.8) | -3.19 (-3.69 to -2.68) |
| Ghana |  | 236.7 (148.3 to 335.5) | 176.7 (122.0 to 255.7) | -0.75 (-0.89 to -0.61) |
| Greece |  | 7.1 (6.0 to 8.5) | 4.8 (3.9 to 5.6) | -0.78 (-1.08 to -0.47) |
| Grenada |  | 35.4 (28.7 to 42.9) | 74.7 (57.4 to 94.0) | 3.40 (2.88 to 3.92) |
| Guinea |  | 663.7 (483.6 to 872.8) | 407.5 (250.2 to 610.1) | -1.36 (-1.46 to -1.26) |
| Guinea-Bissau |  | 407.1 (256.2 to 600.5) | 223.4 (132.3 to 362.2) | -1.92 (-2.10 to -1.74) |
| Guyana |  | 82.1 (61.3 to 106.2) | 165.7 (109.6 to 235.5) | 2.72 (2.29 to 3.15) |
| Hungary |  | 20.1 (17.3 to 23.7) | 5.1 (4.1 to 6.3) | -3.69 (-4.55 to -2.83) |
| Indonesia |  | 472.5 (399.4 to 547.5) | 145.0 (107.8 to 204.4) | -4.08 (-4.24 to -3.92) |
| Iran |  | 45.4 (32.4 to 60.2) | 16.1 (11.0 to 21.6) | -3.39 (-3.54 to -3.24) |
| Iraq |  | 66.6 (39.2 to 105.1) | 28.6 (16.3 to 48.5) | -2.89 (-3.17 to -2.60) |
| Italy |  | 8.7 (7.4 to 10.4) | 2.9 (2.3 to 3.4) | -2.37 (-3.06 to -1.67) |
| Jamaica |  | 32.5 (26.1 to 40.4) | 73.8 (48.4 to 110.2) | 1.06 (0.50 to 1.62) |
| Kazakhstan |  | 50.8 (43.7 to 58.4) | 10.2 (8.0 to 12.9) | -6.34 (-7.01 to -5.66) |
| Kenya |  | 362.8 (263.3 to 481.4) | 291.7 (193.8 to 424.7) | -0.30 (-0.60 to 0.00) |
| Kiribati |  | 209.5 (136.5 to 314.6) | 181.2 (103.8 to 296.5) | -0.50 (-0.57 to -0.43) |
| Kuwait |  | 5.4 (4.4 to 6.8) | 7.0 (5.6 to 9.0) | -0.06 (-1.45 to 1.36) |
| Kyrgyzstan |  | 71.3 (60.7 to 82.4) | 28.9 (22.1 to 36.6) | -3.00 (-3.31 to -2.69) |
| Lao People's Democratic Republic |  | 661.5 (447.8 to 923.1) | 153.3 (76.2 to 278.6) | -4.88 (-5.08 to -4.68) |
| Latvia |  | 37.1 (31.1 to 43.7) | 4.5 (3.6 to 5.7) | -7.45 (-7.97 to -6.93) |
| Lebanon |  | 38.1 (21.1 to 61.3) | 15.3 (8.7 to 25.1) | -2.96 (-3.37 to -2.55) |
| Lesotho |  | 268.2 (183.7 to 375.2) | 331.4 (193.5 to 528.3) | 1.54 (0.81 to 2.28) |
| Liberia |  | 688.2 (470.7 to 930.8) | 564.1 (348.3 to 840.3) | -0.84 (-0.98 to -0.70) |
| Libya |  | 51.0 (28.6 to 79.5) | 59.2 (31.7 to 99.7) | 1.35 (0.90 to 1.80) |
| Lithuania |  | 23.0 (19.7 to 27.4) | 3.4 (2.7 to 4.3) | -6.32 (-6.57 to -6.07) |
| Luxembourg |  | 10.5 (9.0 to 12.6) | 4.8 (3.8 to 5.9) | -2.56 (-2.95 to -2.17) |
| Madagascar |  | 341.4 (242.8 to 463.8) | 288.3 (188.4 to 399.7) | -0.83 (-1.14 to -0.53) |
| Malaysia |  | 92.1 (58.0 to 134.0) | 65.8 (48.1 to 87.0) | -1.20 (-1.45 to -0.95) |
| Maldives |  | 292.8 (209.0 to 387.2) | 62.6 (40.6 to 91.3) | -5.19 (-5.51 to -4.88) |
| Mali |  | 525.6 (445.2 to 607.7) | 271.4 (197.2 to 370.6) | -2.21 (-2.34 to -2.08) |
| Malta |  | 12.6 (10.7 to 15.0) | 8.1 (6.7 to 9.8) | -0.10 (-0.75 to 0.55) |
| Mauritania |  | 934.6 (704.1 to 1185.1) | 387.6 (208.7 to 634.9) | -3.09 (-3.20 to -2.98) |
| Micronesia |  | 162.3 (89.7 to 257.2) | 133.1 (70.1 to 224.9) | -0.58 (-0.76 to -0.40) |
| Mongolia |  | 129.7 (87.2 to 175.7) | 38.5 (25.5 to 55.3) | -5.13 (-5.60 to -4.65) |
| Montenegro |  | 10.9 (6.1 to 17.1) | 6.2 (3.6 to 10.1) | -1.60 (-2.20 to -1.00) |
| Morocco |  | 360.8 (270.9 to 467.4) | 91.7 (49.2 to 157.5) | -4.80 (-5.32 to -4.28) |
| Mozambique |  | 329.8 (236.1 to 445.9) | 216.7 (134.2 to 327.3) | -0.67 (-0.94 to -0.41) |
| Myanmar |  | 204.6 (149.4 to 278.1) | 150.0 (99.7 to 210.5) | -1.20 (-1.52 to -0.89) |
| Namibia |  | 220.3 (146.5 to 303.2) | 111.1 (54.3 to 202.8) | -2.50 (-3.14 to -1.86) |
| Nepal |  | 584.6 (367.0 to 830.9) | 190.0 (106.6 to 317.8) | -3.29 (-3.55 to -3.03) |
| New Zealand |  | 10.7 (9.1 to 12.5) | 5.8 (5.0 to 7.1) | -1.64 (-2.61 to -0.66) |
| Nicaragua |  | 77.9 (60.8 to 94.8) | 46.4 (35.6 to 59.8) | -2.11 (-2.49 to -1.74) |
| Niger |  | 453.3 (346.1 to 591.4) | 335.4 (209.2 to 496.8) | -1.00 (-1.11 to -0.89) |
| Nigeria |  | 429.0 (276.8 to 607.9) | 298.8 (192.2 to 447.7) | -1.32 (-1.42 to -1.23) |
| Niue |  | 65.9 (33.0 to 117.3) | 112.6 (67.9 to 176.7) | 0.30 (-0.17 to 0.77) |
| North Macedonia |  | 18.7 (12.3 to 26.0) | 7.0 (4.2 to 11.2) | -2.92 (-3.21 to -2.64) |
| Oman |  | 32.9 (16.7 to 60.1) | 19.0 (11.0 to 31.7) | -1.34 (-1.52 to -1.16) |
| Pakistan |  | 309.2 (197.9 to 435.7) | 189.9 (106.0 to 319.7) | -2.00 (-2.37 to -1.64) |
| Panama |  | 68.3 (57.2 to 80.1) | 68.9 (53.0 to 86.3) | 0.17 (-0.20 to 0.54) |
| Papua New Guinea |  | 272.6 (136.2 to 412.7) | 212.6 (139.2 to 301.3) | -0.69 (-0.93 to -0.45) |
| Peru |  | 203.6 (155.6 to 259.4) | 90.7 (56.2 to 136.6) | -3.53 (-4.07 to -3.00) |
| Philippines |  | 94.7 (72.1 to 119.4) | 82.3 (60.6 to 113.1) | -0.19 (-0.35 to -0.03) |
| Poland |  | 16.0 (13.4 to 19.0) | 1.8 (1.4 to 2.2) | -6.88 (-7.32 to -6.45) |
| Portugal |  | 13.8 (12.0 to 16.1) | 7.0 (5.8 to 8.0) | -1.91 (-2.26 to -1.56) |
| Qatar |  | 33.3 (19.1 to 52.3) | 10.2 (5.6 to 17.4) | -4.12 (-4.51 to -3.74) |
| Republic of Korea |  | 21.7 (16.8 to 27.2) | 9.7 (6.8 to 13.3) | -2.11 (-2.36 to -1.87) |
| Republic of Moldova |  | 51.1 (44.3 to 59.7) | 12.5 (10.1 to 15.3) | -4.87 (-5.24 to -4.50) |
| Romania |  | 77.7 (64.6 to 90.6) | 11.7 (9.4 to 14.3) | -5.97 (-6.36 to -5.58) |
| Russian Federation |  | 52.1 (43.1 to 60.5) | 11.1 (9.0 to 13.7) | -6.22 (-6.66 to -5.78) |
| Rwanda |  | 521.7 (380.1 to 695.1) | 242.9 (157.2 to 349.7) | -3.06 (-3.44 to -2.68) |
| Samoa |  | 40.9 (22.5 to 68.0) | 26.9 (14.9 to 45.7) | -1.41 (-1.80 to -1.02) |
| Sao Tome and Principe |  | 177.0 (104.9 to 265.5) | 86.7 (44.5 to 155.7) | -3.14 (-3.51 to -2.77) |
| Saudi Arabia |  | 95.3 (52.1 to 156.0) | 89.5 (52.8 to 142.9) | 0.28 (-0.03 to 0.59) |
| Senegal |  | 415.6 (311.6 to 525.2) | 313.5 (212.7 to 450.3) | -0.76 (-0.89 to -0.63) |
| Serbia |  | 21.7 (13.5 to 31.1) | 8.4 (5.3 to 12.7) | -2.86 (-3.06 to -2.67) |
| Seychelles |  | 57.9 (38.1 to 80.9) | 61.5 (39.9 to 89.7) | -0.26 (-0.60 to 0.08) |
| Sierra Leone |  | 538.9 (347.8 to 750.9) | 454.3 (270.2 to 679.6) | -0.44 (-0.81 to -0.06) |
| Singapore |  | 10.9 (9.4 to 12.9) | 2.8 (2.4 to 3.4) | -4.03 (-4.35 to -3.70) |
| Slovakia |  | 13.5 (8.7 to 19.5) | 4.7 (3.0 to 7.2) | -3.16 (-3.55 to -2.77) |
| Slovenia |  | 13.1 (10.7 to 15.3) | 1.4 (1.0 to 1.8) | -6.72 (-7.03 to -6.41) |
| Solomon Islands |  | 260.1 (110.6 to 421.5) | 315.0 (195.8 to 473.4) | 0.67 (0.54 to 0.80) |
| Somalia |  | 734.1 (457.8 to 1048.6) | 460.0 (292.0 to 681.0) | -1.65 (-1.69 to -1.60) |
| South Africa |  | 218.2 (148.6 to 300.9) | 143.0 (108.6 to 181.2) | -0.29 (-1.69 to 1.13) |
| South Sudan |  | 520.4 (285.4 to 810.3) | 484.4 (274.5 to 782.4) | -0.41 (-0.69 to -0.12) |
| Sri Lanka |  | 78.5 (50.5 to 113.3) | 23.5 (14.2 to 36.0) | -4.41 (-4.73 to -4.09) |
| Sudan |  | 406.9 (273.2 to 581.3) | 196.5 (98.8 to 338.6) | -2.23 (-2.33 to -2.14) |
| Suriname |  | 93.1 (51.0 to 139.7) | 138.1 (83.7 to 204.7) | 1.55 (1.01 to 2.11) |
| Syrian Arab Republic |  | 99.3 (62.7 to 143.5) | 33.8 (18.0 to 59.6) | -3.74 (-4.50 to -2.98) |
| Tajikistan |  | 63.2 (51.5 to 77.8) | 24.9 (16.4 to 36.0) | -3.90 (-4.37 to -3.42) |
| Thailand |  | 59.9 (33.4 to 94.6) | 63.2 (38.2 to 100.5) | -0.05 (-0.77 to 0.67) |
| Timor-Leste |  | 631.4 (401.0 to 876.7) | 189.9 (117.2 to 297.1) | -4.27 (-4.51 to -4.03) |
| Togo |  | 335.6 (224.1 to 466.3) | 216.2 (121.7 to 338.1) | -1.79 (-2.23 to -1.35) |
| Tonga |  | 93.7 (55.6 to 143.9) | 88.1 (50.2 to 142.6) | -0.37 (-0.56 to -0.18) |
| Trinidad and Tobago |  | 59.9 (51.0 to 71.3) | 93.0 (65.2 to 127.8) | 1.28 (1.01 to 1.56) |
| Tunisia |  | 73.8 (48.4 to 105.5) | 34.3 (18.1 to 58.3) | -2.51 (-2.58 to -2.44) |
| Turkey |  | 126.4 (78.2 to 185.4) | 26.4 (16.2 to 41.3) | -5.72 (-6.64 to -4.79) |
| Uganda |  | 256.9 (180.5 to 345.2) | 168.2 (105.5 to 238.1) | -1.39 (-1.70 to -1.09) |
| Ukraine |  | 33.8 (30.2 to 38.0) | 13.8 (7.4 to 22.2) | -3.47 (-3.92 to -3.02) |
| United Arab Emirates |  | 28.5 (14.4 to 51.1) | 24.8 (14.5 to 40.6) | -0.72 (-1.43 to 0.00) |
| United Republic of Tanzania |  | 388.0 (298.1 to 485.5) | 319.4 (212.2 to 445.5) | -0.21 (-0.45 to 0.03) |
| Uruguay |  | 31.3 (26.8 to 36.2) | 28.1 (23.1 to 36.1) | -0.10 (-0.39 to 0.19) |
| Uzbekistan |  | 39.7 (31.5 to 48.3) | 22.2 (16.3 to 29.0) | -1.33 (-2.05 to -0.60) |
| Vanuatu |  | 72.0 (36.7 to 129.6) | 79.9 (45.5 to 132.5) | -0.02 (-0.20 to 0.16) |
| Venezuela |  | 72.7 (63.7 to 82.6) | 104.2 (73.2 to 141.2) | 1.29 (0.63 to 1.95) |
| Viet Nam |  | 45.2 (22.5 to 75.3) | 17.0 (9.4 to 29.2) | -3.24 (-3.61 to -2.88) |
| Yemen |  | 425.8 (266.1 to 608.9) | 195.5 (108.8 to 329.3) | -2.82 (-2.96 to -2.67) |
| Zambia |  | 281.8 (204.2 to 375.9) | 182.3 (105.4 to 279.8) | -1.95 (-2.20 to -1.70) |
| Zimbabwe |  | 194.3 (132.1 to 270.7) | 287.9 (167.8 to 449.8) | 2.77 (1.74 to 3.81) |

BRI countries: the Belt and Road countries; EAPC: the estimated annual percentage change.

| Supplementary Table 10: Under-five mortality rate (/1,000 livebirths) in 149 BRI Countries between 1990 and 2021 | | | | |
| --- | --- | --- | --- | --- |
| Country |  | 1990 | 2021 | EAPC during 1990-2021 (95% CI) (%) |
| Afghanistan |  | 150.3 | 47.9 | -3.75 (-4.05 to -3.45) |
| Albania |  | 40.3 | 13.1 | -3.78 (-3.98 to -3.58) |
| Algeria |  | 57.2 | 17.0 | -3.55 (-3.66 to -3.44) |
| Angola |  | 199.0 | 54.1 | -4.48 (-4.74 to -4.22) |
| Antigua and Barbuda |  | 13.8 | 9.4 | -1.24 (-1.49 to -0.98) |
| Argentina |  | 29.0 | 9.8 | -3.37 (-3.52 to -3.22) |
| Armenia |  | 41.8 | 11.1 | -4.44 (-4.66 to -4.22) |
| Austria |  | 9.4 | 3.1 | -3.21 (-3.48 to -2.95) |
| Azerbaijan |  | 77.3 | 28.1 | -3.62 (-3.83 to -3.40) |
| Bahrain |  | 23.1 | 6.0 | -3.83 (-4.26 to -3.39) |
| Bangladesh |  | 132.4 | 28.2 | -4.77 (-4.88 to -4.66) |
| Barbados |  | 18.0 | 11.9 | -1.07 (-1.34 to -0.80) |
| Belarus |  | 19.2 | 4.1 | -5.55 (-5.93 to -5.17) |
| Benin |  | 170.3 | 75.8 | -2.42 (-2.52 to -2.32) |
| Bolivia |  | 109.1 | 27.7 | -4.41 (-4.46 to -4.35) |
| Bosnia and Herzegovina |  | 17.3 | 5.6 | -3.94 (-4.49 to -3.39) |
| Botswana |  | 62.3 | 40.5 | -1.80 (-2.16 to -1.45) |
| Brunei Darussalam |  | 11.4 | 9.3 | -0.21 (-0.40 to -0.03) |
| Bulgaria |  | 19.9 | 6.7 | -4.22 (-4.51 to -3.93) |
| Burkina Faso |  | 203.2 | 92.3 | -2.49 (-2.63 to -2.36) |
| Burundi |  | 177.9 | 63.1 | -3.77 (-3.99 to -3.55) |
| Côte d’Ivoire |  | 143.1 | 67.7 | -2.44 (-2.67 to -2.20) |
| Cabo Verde |  | 65.1 | 15.1 | -4.99 (-5.25 to -4.73) |
| Cambodia |  | 118.1 | 29.4 | -4.91 (-5.16 to -4.66) |
| Cameroon |  | 132.0 | 65.4 | -2.35 (-2.57 to -2.13) |
| Central African Republic |  | 183.2 | 107.7 | -1.59 (-1.77 to -1.41) |
| Chad |  | 189.4 | 108.0 | -1.86 (-1.99 to -1.72) |
| Chile |  | 19.1 | 5.9 | -2.95 (-3.21 to -2.69) |
| China |  | 54.1 | 8.0 | -6.76 (-7.04 to -6.48) |
| Comoros |  | 120.9 | 47.2 | -2.97 (-3.11 to -2.82) |
| Congo |  | 99.2 | 38.9 | -3.31 (-3.61 to -3.00) |
| Cook Islands |  | 26.3 | 5.5 | -6.52 (-7.37 to -5.66) |
| Costa Rica |  | 17.5 | 9.5 | -2.15 (-2.38 to -1.93) |
| Croatia |  | 12.4 | 4.6 | -3.62 (-3.92 to -3.31) |
| Cuba |  | 14.0 | 4.7 | -3.11 (-3.52 to -2.71) |
| Cyprus |  | 12.8 | 2.4 | -5.79 (-6.07 to -5.50) |
| Czechia |  | 12.0 | 2.7 | -4.77 (-5.34 to -4.19) |
| Democratic Republic of the Congo |  | 158.4 | 57.6 | -3.20 (-3.56 to -2.84) |
| Djibouti |  | 107.1 | 44.3 | -2.97 (-3.17 to -2.77) |
| Dominica |  | 18.2 | 28.2 | 2.01 (1.61 to 2.41) |
| Dominican Republic |  | 63.6 | 25.0 | -2.70 (-2.90 to -2.51) |
| Ecuador |  | 41.0 | 13.8 | -3.32 (-3.56 to -3.09) |
| Egypt |  | 83.4 | 12.7 | -5.66 (-5.79 to -5.52) |
| El Salvador |  | 50.4 | 9.5 | -5.03 (-5.16 to -4.90) |
| Equatorial Guinea |  | 157.5 | 46.5 | -4.62 (-4.87 to -4.37) |
| Eritrea |  | 146.0 | 44.8 | -3.66 (-3.82 to -3.51) |
| Estonia |  | 18.0 | 2.5 | -7.30 (-7.57 to -7.04) |
| Ethiopia |  | 185.6 | 51.3 | -4.32 (-4.47 to -4.18) |
| Fiji |  | 25.5 | 21.0 | -0.59 (-0.74 to -0.43) |
| Gabon |  | 77.3 | 32.2 | -2.48 (-2.76 to -2.20) |
| Gambia |  | 120.2 | 43.1 | -3.25 (-3.34 to -3.16) |
| Georgia |  | 43.1 | 9.8 | -5.53 (-5.88 to -5.18) |
| Ghana |  | 119.9 | 43.4 | -2.97 (-3.18 to -2.76) |
| Greece |  | 10.6 | 4.0 | -3.06 (-3.68 to -2.44) |
| Grenada |  | 23.2 | 12.5 | -1.44 (-1.67 to -1.21) |
| Guinea |  | 221.6 | 85.9 | -2.86 (-2.97 to -2.75) |
| Guinea-Bissau |  | 198.9 | 60.6 | -4.04 (-4.25 to -3.83) |
| Guyana |  | 52.0 | 22.2 | -2.43 (-2.56 to -2.30) |
| Hungary |  | 17.0 | 4.1 | -4.69 (-4.89 to -4.49) |
| Indonesia |  | 84.3 | 24.3 | -3.82 (-3.93 to -3.70) |
| Iran |  | 76.3 | 5.5 | -6.16 (-6.84 to -5.48) |
| Iraq |  | 52.4 | 16.8 | -3.53 (-3.71 to -3.35) |
| Italy |  | 9.5 | 3.0 | -3.67 (-3.99 to -3.35) |
| Jamaica |  | 29.1 | 15.1 | -1.86 (-2.00 to -1.72) |
| Kazakhstan |  | 36.5 | 9.7 | -4.95 (-5.47 to -4.44) |
| Kenya |  | 92.7 | 36.6 | -3.15 (-3.35 to -2.95) |
| Kiribati |  | 80.4 | 36.4 | -2.53 (-2.57 to -2.48) |
| Kuwait |  | 18.9 | 8.0 | -1.59 (-2.01 to -1.17) |
| Kyrgyzstan |  | 57.2 | 16.7 | -4.34 (-4.57 to -4.10) |
| Lao People’s Democratic Republic |  | 166.1 | 39.7 | -4.78 (-4.96 to -4.60) |
| Latvia |  | 18.0 | 3.8 | -5.89 (-6.25 to -5.53) |
| Lebanon |  | 30.4 | 7.5 | -4.44 (-4.53 to -4.34) |
| Lesotho |  | 88.2 | 77.2 | -0.64 (-0.95 to -0.32) |
| Liberia |  | 235.4 | 66.5 | -4.39 (-4.72 to -4.05) |
| Libya |  | 40.3 | 22.4 | -1.17 (-1.72 to -0.62) |
| Lithuania |  | 14.9 | 3.6 | -5.41 (-5.68 to -5.14) |
| Luxembourg |  | 8.7 | 3.5 | -3.32 (-4.14 to -2.50) |
| Madagascar |  | 138.3 | 56.0 | -2.64 (-2.74 to -2.54) |
| Malaysia |  | 17.2 | 6.3 | -2.58 (-3.13 to -2.03) |
| Maldives |  | 64.6 | 12.6 | -4.99 (-5.29 to -4.68) |
| Mali |  | 225.0 | 95.0 | -2.72 (-2.83 to -2.60) |
| Malta |  | 11.0 | 5.3 | -1.88 (-2.17 to -1.58) |
| Mauritania |  | 96.2 | 33.6 | -3.26 (-3.47 to -3.05) |
| Micronesia |  | 41.7 | 15.5 | -3.30 (-3.47 to -3.13) |
| Mongolia |  | 85.4 | 16.2 | -5.72 (-5.90 to -5.54) |
| Montenegro |  | 16.6 | 3.9 | -5.49 (-6.02 to -4.96) |
| Morocco |  | 74.9 | 14.6 | -4.90 (-5.08 to -4.72) |
| Mozambique |  | 209.6 | 61.7 | -3.85 (-3.98 to -3.72) |
| Myanmar |  | 145.0 | 39.3 | -4.32 (-4.62 to -4.03) |
| Namibia |  | 67.6 | 32.9 | -2.28 (-2.61 to -1.96) |
| Nepal |  | 132.0 | 28.4 | -4.84 (-4.91 to -4.76) |
| New Zealand |  | 10.7 | 5.0 | -2.19 (-2.43 to -1.95) |
| Nicaragua |  | 62.1 | 13.9 | -4.70 (-4.90 to -4.50) |
| Niger |  | 270.8 | 85.2 | -4.01 (-4.20 to -3.82) |
| Nigeria |  | 190.1 | 94.4 | -2.15 (-2.32 to -1.99) |
| Niue |  | 26.1 | 53.2 | -0.01 (-0.85 to 0.85) |
| North Macedonia |  | 37.9 | 5.7 | -4.84 (-5.39 to -4.29) |
| Oman |  | 32.7 | 8.9 | -3.28 (-4.08 to -2.47) |
| Pakistan |  | 108.8 | 57.0 | -1.87 (-1.97 to -1.78) |
| Panama |  | 27.0 | 14.4 | -1.86 (-1.94 to -1.77) |
| Papua New Guinea |  | 81.2 | 51.2 | -1.43 (-1.50 to -1.36) |
| Peru |  | 72.7 | 13.9 | -5.03 (-5.33 to -4.73) |
| Philippines |  | 53.0 | 21.8 | -2.28 (-2.48 to -2.08) |
| Poland |  | 18.4 | 4.4 | -5.02 (-5.36 to -4.68) |
| Portugal |  | 14.2 | 3.0 | -4.73 (-5.19 to -4.26) |
| Qatar |  | 16.5 | 3.7 | -4.58 (-4.71 to -4.45) |
| Republic of Korea |  | 11.9 | 2.5 | -4.33 (-4.63 to -4.02) |
| Republic of Moldova |  | 33.2 | 11.0 | -3.66 (-3.91 to -3.40) |
| Romania |  | 36.0 | 6.8 | -5.27 (-5.52 to -5.02) |
| Russian Federation |  | 22.1 | 6.0 | -4.80 (-5.11 to -4.48) |
| Rwanda |  | 161.4 | 40.5 | -5.28 (-5.69 to -4.86) |
| Samoa |  | 29.1 | 12.7 | -2.05 (-2.51 to -1.58) |
| Sao Tome and Principe |  | 102.4 | 17.8 | -5.71 (-6.03 to -5.40) |
| Saudi Arabia |  | 45.1 | 4.3 | -7.41 (-7.55 to -7.27) |
| Senegal |  | 133.2 | 40.3 | -3.93 (-4.16 to -3.70) |
| Serbia |  | 27.5 | 5.2 | -5.61 (-6.16 to -5.06) |
| Seychelles |  | 18.4 | 13.3 | -0.48 (-0.80 to -0.17) |
| Sierra Leone |  | 227.4 | 95.6 | -2.91 (-3.13 to -2.68) |
| Singapore |  | 8.0 | 1.7 | -4.40 (-4.76 to -4.05) |
| Slovakia |  | 14.5 | 5.7 | -3.23 (-3.50 to -2.97) |
| Slovenia |  | 10.2 | 2.2 | -4.99 (-5.23 to -4.75) |
| Solomon Islands |  | 43.5 | 19.1 | -2.46 (-2.58 to -2.35) |
| Somalia |  | 182.3 | 89.5 | -2.04 (-2.60 to -1.48) |
| South Africa |  | 77.9 | 38.9 | -2.43 (-2.85 to -2.01) |
| South Sudan |  | 194.8 | 124.3 | -1.35 (-1.67 to -1.03) |
| Sri Lanka |  | 20.6 | 6.0 | -3.89 (-4.53 to -3.24) |
| Sudan |  | 136.1 | 36.3 | -4.05 (-4.19 to -3.91) |
| Suriname |  | 49.4 | 24.5 | -2.30 (-2.42 to -2.18) |
| Syrian Arab Republic |  | 45.6 | 10.1 | -3.39 (-4.28 to -2.50) |
| Tajikistan |  | 77.9 | 34.0 | -3.01 (-3.23 to -2.80) |
| Thailand |  | 28.9 | 7.0 | -4.47 (-4.70 to -4.25) |
| Timor-Leste |  | 123.2 | 34.3 | -4.36 (-4.54 to -4.18) |
| Togo |  | 139.1 | 56.8 | -2.76 (-2.95 to -2.58) |
| Tonga |  | 24.8 | 11.6 | -2.32 (-2.48 to -2.16) |
| Trinidad and Tobago |  | 27.8 | 13.8 | -2.52 (-2.75 to -2.28) |
| Tunisia |  | 51.9 | 10.4 | -4.82 (-4.94 to -4.70) |
| Turkey |  | 70.6 | 10.8 | -5.78 (-5.90 to -5.66) |
| Uganda |  | 159.6 | 63.4 | -3.49 (-3.65 to -3.32) |
| Ukraine |  | 19.2 | 7.9 | -3.01 (-3.22 to -2.80) |
| United Arab Emirates |  | 20.6 | 5.1 | -3.47 (-3.85 to -3.09) |
| United Republic of Tanzania |  | 147.5 | 51.6 | -3.49 (-3.62 to -3.37) |
| Uruguay |  | 23.4 | 6.9 | -3.90 (-4.09 to -3.72) |
| Uzbekistan |  | 52.4 | 21.4 | -3.07 (-3.26 to -2.88) |
| Vanuatu |  | 35.8 | 20.6 | -1.77 (-2.06 to -1.48) |
| Venezuela |  | 32.7 | 21.5 | -1.34 (-2.05 to -0.63) |
| Viet Nam |  | 45.7 | 11.3 | -4.39 (-4.52 to -4.26) |
| Yemen |  | 128.3 | 38.2 | -3.91 (-4.01 to -3.81) |
| Zambia |  | 153.6 | 45.8 | -4.16 (-4.48 to -3.84) |
| Zimbabwe |  | 66.3 | 52.2 | -0.77 (-1.13 to -0.41) |

BRI countries: the Belt and Road countries; EAPC: the estimated annual percentage change.

| Supplementary Table 11: Neonatal mortality rate (/1,000 livebirths) in 149 BRI Countries between 1990 and 2021 | | | | |
| --- | --- | --- | --- | --- |
| Country |  | 1990 | 2021 | EAPC during 1990-2021 (95% CI) (%) |
| Afghanistan |  | 48.5 | 19.9 | -2.99 (-3.15 to -2.84) |
| Albania |  | 14.0 | 8.1 | -1.69 (-1.89 to -1.49) |
| Algeria |  | 25.3 | 10.4 | -2.51 (-2.67 to -2.35) |
| Angola |  | 47.5 | 20.2 | -2.88 (-3.00 to -2.75) |
| Antigua and Barbuda |  | 8.1 | 5.0 | -1.67 (-1.88 to -1.46) |
| Argentina |  | 15.9 | 5.9 | -3.16 (-3.35 to -2.98) |
| Armenia |  | 19.9 | 5.8 | -4.13 (-4.30 to -3.95) |
| Austria |  | 4.6 | 2.0 | -2.40 (-2.63 to -2.17) |
| Azerbaijan |  | 30.5 | 17.0 | -2.05 (-2.32 to -1.78) |
| Bahrain |  | 11.3 | 2.4 | -4.52 (-5.02 to -4.02) |
| Bangladesh |  | 58.2 | 19.0 | -3.29 (-3.44 to -3.13) |
| Barbados |  | 12.1 | 8.2 | -0.78 (-1.10 to -0.46) |
| Belarus |  | 10.4 | 2.0 | -5.89 (-6.28 to -5.50) |
| Benin |  | 44.4 | 28.9 | -1.20 (-1.28 to -1.12) |
| Bolivia |  | 34.2 | 13.7 | -2.88 (-2.92 to -2.85) |
| Bosnia and Herzegovina |  | 13.4 | 4.0 | -4.08 (-4.58 to -3.57) |
| Botswana |  | 27.4 | 19.7 | -0.94 (-1.04 to -0.85) |
| Brunei Darussalam |  | 5.6 | 5.3 | 0.27 (0.03 to 0.51) |
| Bulgaria |  | 8.2 | 3.2 | -3.66 (-3.95 to -3.37) |
| Burkina Faso |  | 47.2 | 27.9 | -1.50 (-1.56 to -1.43) |
| Burundi |  | 41.4 | 24.6 | -1.76 (-1.93 to -1.59) |
| Côte d’Ivoire |  | 45.8 | 29.1 | -1.41 (-1.57 to -1.24) |
| Cabo Verde |  | 19.9 | 8.9 | -2.25 (-2.55 to -1.95) |
| Cambodia |  | 41.2 | 15.8 | -3.10 (-3.17 to -3.03) |
| Cameroon |  | 39.7 | 22.9 | -1.63 (-1.71 to -1.56) |
| Central African Republic |  | 51.6 | 37.2 | -0.95 (-1.01 to -0.88) |
| Chad |  | 47.2 | 33.3 | -1.09 (-1.12 to -1.06) |
| Chile |  | 8.8 | 3.9 | -1.53 (-1.86 to -1.19) |
| China |  | 17.5 | 3.2 | -5.58 (-5.96 to -5.20) |
| Comoros |  | 48.4 | 26.1 | -2.00 (-2.14 to -1.86) |
| Congo |  | 26.6 | 16.3 | -1.59 (-1.80 to -1.37) |
| Cook Islands |  | 8.8 | 1.8 | -6.61 (-7.25 to -5.96) |
| Costa Rica |  | 9.5 | 6.0 | -1.59 (-1.75 to -1.44) |
| Croatia |  | 7.9 | 3.0 | -3.60 (-3.88 to -3.32) |
| Cuba |  | 7.1 | 1.9 | -3.68 (-4.04 to -3.32) |
| Cyprus |  | 7.4 | 1.4 | -5.78 (-6.09 to -5.47) |
| Czechia |  | 7.0 | 1.4 | -4.93 (-5.58 to -4.27) |
| Democratic Republic of the Congo |  | 36.5 | 21.7 | -1.48 (-1.70 to -1.26) |
| Djibouti |  | 33.6 | 19.3 | -1.77 (-1.90 to -1.64) |
| Dominica |  | 10.7 | 16.2 | 1.81 (1.53 to 2.10) |
| Dominican Republic |  | 26.7 | 17.5 | -1.20 (-1.35 to -1.06) |
| Ecuador |  | 17.1 | 7.3 | -2.54 (-2.83 to -2.24) |
| Egypt |  | 31.6 | 4.7 | -5.93 (-6.37 to -5.48) |
| El Salvador |  | 16.8 | 4.5 | -3.67 (-3.85 to -3.48) |
| Equatorial Guinea |  | 41.5 | 17.8 | -3.08 (-3.22 to -2.95) |
| Eritrea |  | 34.0 | 17.9 | -1.87 (-1.95 to -1.79) |
| Estonia |  | 9.1 | 1.1 | -7.84 (-8.14 to -7.53) |
| Ethiopia |  | 53.7 | 25.4 | -2.44 (-2.56 to -2.32) |
| Fiji |  | 10.8 | 9.7 | -0.44 (-0.61 to -0.27) |
| Gabon |  | 27.7 | 16.0 | -1.45 (-1.65 to -1.25) |
| Gambia |  | 40.3 | 21.4 | -2.01 (-2.11 to -1.92) |
| Georgia |  | 22.1 | 5.6 | -5.13 (-5.68 to -4.57) |
| Ghana |  | 39.3 | 20.6 | -1.77 (-1.94 to -1.60) |
| Greece |  | 6.6 | 2.3 | -3.55 (-4.27 to -2.83) |
| Grenada |  | 12.5 | 7.9 | -0.99 (-1.21 to -0.76) |
| Guinea |  | 57.1 | 30.1 | -2.06 (-2.12 to -1.99) |
| Guinea-Bissau |  | 53.0 | 27.6 | -2.13 (-2.34 to -1.92) |
| Guyana |  | 30.5 | 14.1 | -2.20 (-2.35 to -2.05) |
| Hungary |  | 11.0 | 2.1 | -5.33 (-5.55 to -5.11) |
| Indonesia |  | 30.7 | 13.2 | -2.66 (-2.71 to -2.61) |
| Iran |  | 29.2 | 3.2 | -5.28 (-5.99 to -4.57) |
| Iraq |  | 26.1 | 9.7 | -3.16 (-3.36 to -2.96) |
| Italy |  | 5.5 | 1.8 | -3.57 (-3.89 to -3.26) |
| Jamaica |  | 17.6 | 12.1 | -1.17 (-1.28 to -1.07) |
| Kazakhstan |  | 13.5 | 4.5 | -3.92 (-4.52 to -3.32) |
| Kenya |  | 28.5 | 17.4 | -1.37 (-1.53 to -1.21) |
| Kiribati |  | 21.4 | 14.5 | -1.29 (-1.44 to -1.13) |
| Kuwait |  | 9.2 | 4.4 | -1.42 (-1.81 to -1.03) |
| Kyrgyzstan |  | 27.3 | 10.8 | -3.03 (-3.22 to -2.84) |
| Lao People’s Democratic Republic |  | 51.3 | 19.0 | -3.35 (-3.47 to -3.24) |
| Latvia |  | 8.9 | 2.1 | -5.52 (-5.91 to -5.14) |
| Lebanon |  | 16.5 | 4.0 | -4.49 (-4.60 to -4.39) |
| Lesotho |  | 39.0 | 29.7 | -0.91 (-1.01 to -0.80) |
| Liberia |  | 52.0 | 25.3 | -2.28 (-2.41 to -2.15) |
| Libya |  | 20.0 | 10.3 | -1.90 (-2.47 to -1.33) |
| Lithuania |  | 7.7 | 1.9 | -5.30 (-5.66 to -4.95) |
| Luxembourg |  | 4.3 | 2.0 | -2.80 (-3.61 to -1.97) |
| Madagascar |  | 35.9 | 22.6 | -1.31 (-1.43 to -1.19) |
| Malaysia |  | 8.2 | 3.5 | -1.98 (-2.55 to -1.40) |
| Maldives |  | 28.5 | 7.5 | -4.06 (-4.23 to -3.89) |
| Mali |  | 62.8 | 37.7 | -1.53 (-1.60 to -1.47) |
| Malta |  | 7.6 | 3.7 | -1.86 (-2.16 to -1.55) |
| Mauritania |  | 37.9 | 18.2 | -2.21 (-2.39 to -2.03) |
| Micronesia |  | 15.0 | 7.3 | -2.41 (-2.55 to -2.26) |
| Mongolia |  | 22.8 | 8.0 | -3.60 (-3.87 to -3.33) |
| Montenegro |  | 10.8 | 2.5 | -5.82 (-6.43 to -5.21) |
| Morocco |  | 34.2 | 9.2 | -3.96 (-4.20 to -3.72) |
| Mozambique |  | 47.2 | 24.7 | -2.07 (-2.15 to -1.99) |
| Myanmar |  | 40.7 | 18.7 | -2.58 (-2.80 to -2.36) |
| Namibia |  | 27.2 | 15.2 | -1.55 (-1.70 to -1.40) |
| Nepal |  | 55.4 | 19.1 | -3.32 (-3.42 to -3.22) |
| New Zealand |  | 5.0 | 2.9 | -1.49 (-1.75 to -1.22) |
| Nicaragua |  | 20.5 | 8.3 | -2.72 (-2.83 to -2.62) |
| Niger |  | 42.1 | 24.6 | -2.20 (-2.40 to -1.99) |
| Nigeria |  | 51.1 | 35.4 | -1.04 (-1.15 to -0.94) |
| Niue |  | 10.0 | 17.7 | 0.13 (-0.54 to 0.80) |
| North Macedonia |  | 18.1 | 3.8 | -3.78 (-4.30 to -3.26) |
| Oman |  | 15.8 | 4.5 | -3.17 (-3.93 to -2.41) |
| Pakistan |  | 50.0 | 36.0 | -1.22 (-1.31 to -1.14) |
| Panama |  | 11.8 | 6.4 | -1.81 (-1.98 to -1.64) |
| Papua New Guinea |  | 22.3 | 18.4 | -0.65 (-0.81 to -0.50) |
| Peru |  | 25.8 | 6.9 | -3.79 (-4.08 to -3.50) |
| Philippines |  | 18.2 | 11.0 | -1.23 (-1.34 to -1.13) |
| Poland |  | 10.3 | 2.6 | -4.72 (-5.02 to -4.42) |
| Portugal |  | 7.2 | 1.6 | -4.19 (-4.69 to -3.68) |
| Qatar |  | 8.1 | 1.9 | -4.45 (-4.57 to -4.33) |
| Republic of Korea |  | 5.2 | 1.1 | -4.01 (-4.35 to -3.67) |
| Republic of Moldova |  | 16.3 | 6.6 | -2.97 (-3.37 to -2.58) |
| Romania |  | 9.9 | 3.3 | -3.79 (-4.27 to -3.31) |
| Russian Federation |  | 11.9 | 2.9 | -4.95 (-5.32 to -4.57) |
| Rwanda |  | 42.6 | 17.6 | -2.76 (-2.85 to -2.67) |
| Samoa |  | 11.1 | 5.8 | -1.94 (-2.09 to -1.78) |
| Sao Tome and Principe |  | 22.9 | 9.0 | -2.88 (-3.27 to -2.48) |
| Saudi Arabia |  | 18.6 | 2.1 | -7.04 (-7.30 to -6.77) |
| Senegal |  | 37.8 | 21.2 | -1.82 (-2.01 to -1.63) |
| Serbia |  | 17.7 | 3.6 | -5.37 (-5.92 to -4.81) |
| Seychelles |  | 10.3 | 8.1 | -0.17 (-0.42 to 0.08) |
| Sierra Leone |  | 56.3 | 29.8 | -2.07 (-2.14 to -2.01) |
| Singapore |  | 3.7 | 0.9 | -3.93 (-4.42 to -3.45) |
| Slovakia |  | 8.0 | 2.7 | -3.74 (-4.10 to -3.39) |
| Slovenia |  | 5.7 | 1.3 | -4.67 (-4.88 to -4.46) |
| Solomon Islands |  | 15.4 | 8.8 | -1.72 (-1.76 to -1.67) |
| Somalia |  | 43.9 | 31.1 | -1.03 (-1.12 to -0.94) |
| South Africa |  | 28.7 | 19.1 | -1.61 (-1.81 to -1.42) |
| South Sudan |  | 44.5 | 41.2 | -0.22 (-0.28 to -0.16) |
| Sri Lanka |  | 11.8 | 3.7 | -3.89 (-4.31 to -3.46) |
| Sudan |  | 44.1 | 18.0 | -2.60 (-2.73 to -2.47) |
| Suriname |  | 25.3 | 14.5 | -1.83 (-1.91 to -1.75) |
| Syrian Arab Republic |  | 20.4 | 4.8 | -3.64 (-4.28 to -3.01) |
| Tajikistan |  | 22.7 | 15.5 | -1.34 (-1.48 to -1.21) |
| Thailand |  | 14.4 | 2.6 | -5.59 (-5.86 to -5.32) |
| Timor-Leste |  | 32.9 | 14.7 | -2.65 (-2.75 to -2.55) |
| Togo |  | 42.3 | 23.2 | -1.82 (-1.91 to -1.74) |
| Tonga |  | 9.5 | 5.1 | -1.94 (-2.00 to -1.88) |
| Trinidad and Tobago |  | 17.4 | 8.8 | -2.29 (-2.54 to -2.04) |
| Tunisia |  | 25.0 | 6.5 | -4.04 (-4.16 to -3.92) |
| Turkey |  | 31.0 | 5.5 | -5.19 (-5.36 to -5.03) |
| Uganda |  | 35.6 | 22.4 | -1.35 (-1.47 to -1.22) |
| Ukraine |  | 11.2 | 4.1 | -3.29 (-3.48 to -3.11) |
| United Arab Emirates |  | 11.0 | 2.6 | -3.69 (-4.07 to -3.32) |
| United Republic of Tanzania |  | 36.3 | 22.2 | -1.43 (-1.50 to -1.35) |
| Uruguay |  | 11.7 | 3.9 | -3.45 (-3.62 to -3.27) |
| Uzbekistan |  | 19.7 | 11.1 | -1.86 (-2.07 to -1.64) |
| Vanuatu |  | 13.7 | 9.5 | -1.17 (-1.30 to -1.05) |
| Venezuela |  | 15.5 | 12.7 | -0.31 (-0.88 to 0.26) |
| Viet Nam |  | 21.2 | 6.2 | -3.78 (-3.87 to -3.69) |
| Yemen |  | 41.8 | 19.3 | -2.51 (-2.60 to -2.42) |
| Zambia |  | 30.5 | 19.5 | -1.25 (-1.56 to -0.94) |
| Zimbabwe |  | 21.0 | 23.1 | 0.92 (0.68 to 1.16) |

BRI countries: the Belt and Road countries; EAPC: the estimated annual percentage change.

| Supplementary Table 12: HIV incidence rate (/100,000 persons) in 149 BRI Countries between 1990 and 2021 | | | | |
| --- | --- | --- | --- | --- |
| Country |  | 1990 | 2021 | EAPC during 1990-2021 (95% CI) (%) |
| Afghanistan |  | 0.7 (0.3 to 1.8) | 1.6 (0.6 to 2.6) | 3.54 (3.14 to 3.94) |
| Albania |  | 0.1 (0.1 to 0.2) | 0.1 (0.1 to 0.1) | -2.34 (-2.65 to -2.03) |
| Algeria |  | 0.4 (0.3 to 0.4) | 3.7 (3.3 to 4.2) | 8.12 (7.38 to 8.87) |
| Angola |  | 27.6 (5.2 to 69.5) | 208.2 (94.9 to 391.1) | 4.73 (3.84 to 5.62) |
| Antigua and Barbuda |  | 24.5 (16.4 to 54.3) | 12.1 (8.4 to 17.8) | -1.52 (-1.96 to -1.07) |
| Argentina |  | 19.3 (17.8 to 21.5) | 14.8 (13.5 to 16.2) | -0.71 (-0.85 to -0.57) |
| Armenia |  | 0.2 (0.0 to 1.0) | 9.0 (6.8 to 12.3) | 16.66 (13.81 to 19.57) |
| Austria |  | 15.7 (10.6 to 21.7) | 3.4 (1.8 to 5.5) | -3.09 (-4.00 to -2.17) |
| Azerbaijan |  | 0.7 (0.4 to 2.0) | 2.1 (1.2 to 3.5) | 2.58 (2.08 to 3.09) |
| Bahrain |  | 2.3 (1.8 to 3.0) | 1.0 (0.8 to 1.3) | -3.14 (-3.80 to -2.47) |
| Bangladesh |  | 0.0 (0.0 to 0.0) | 0.9 (0.7 to 1.1) | 14.24 (11.63 to 16.91) |
| Barbados |  | 54.7 (45.8 to 66.0) | 52.4 (40.0 to 71.1) | -1.21 (-1.74 to -0.68) |
| Belarus |  | 3.5 (0.0 to 11.1) | 44.0 (31.2 to 63.7) | 10.58 (9.86 to 11.30) |
| Benin |  | 44.4 (25.7 to 66.3) | 26.2 (12.8 to 50.6) | -4.44 (-5.51 to -3.37) |
| Bolivia |  | 3.3 (0.8 to 8.9) | 9.1 (5.6 to 13.3) | -1.48 (-3.93 to 1.04) |
| Bosnia and Herzegovina |  | 0.1 (0.1 to 0.1) | 0.3 (0.1 to 0.5) | 5.16 (4.43 to 5.89) |
| Botswana |  | 1444.3 (1211.9 to 1783.7) | 226.0 (115.7 to 404.8) | -7.45 (-8.09 to -6.80) |
| Brunei Darussalam |  | 1.8 (1.1 to 2.6) | 5.0 (2.7 to 8.3) | 3.78 (2.90 to 4.65) |
| Bulgaria |  | 1.1 (0.0 to 2.0) | 5.7 (3.6 to 7.5) | 5.76 (5.35 to 6.18) |
| Burkina Faso |  | 359.5 (239.8 to 509.8) | 8.8 (3.4 to 18.3) | -10.13 (-10.53 to -9.72) |
| Burundi |  | 716.4 (238.8 to 1214.2) | 6.1 (3.2 to 11.6) | -14.04 (-14.45 to -13.62) |
| Côte d'Ivoire |  | 901.8 (593.9 to 1294.9) | 49.5 (24.6 to 92.5) | -8.16 (-8.49 to -7.82) |
| Cabo Verde |  | 84.7 (25.3 to 166.4) | 14.7 (3.1 to 63.3) | -5.65 (-5.91 to -5.39) |
| Cambodia |  | 0.6 (0.5 to 0.7) | 6.6 (4.6 to 9.3) | -3.67 (-8.01 to 0.87) |
| Cameroon |  | 202.4 (132.4 to 294.1) | 102.9 (60.7 to 175.5) | -4.03 (-5.00 to -3.05) |
| Central African Republic |  | 1475.2 (1072.4 to 2093.0) | 123.2 (39.6 to 290.0) | -6.36 (-6.96 to -5.76) |
| Chad |  | 129.6 (51.6 to 245.4) | 40.1 (16.5 to 78.2) | -4.11 (-4.48 to -3.74) |
| Chile |  | 5.8 (4.1 to 7.5) | 23.8 (12.7 to 38.5) | 4.30 (4.13 to 4.46) |
| China |  | 0.7 (0.4 to 1.3) | 2.3 (1.3 to 3.8) | 2.36 (0.67 to 4.08) |
| Comoros |  | 0.3 (0.1 to 0.8) | 0.3 (0.1 to 0.5) | -1.07 (-3.20 to 1.10) |
| Congo |  | 586.6 (330.9 to 898.4) | 127.2 (48.3 to 298.8) | -3.82 (-4.37 to -3.26) |
| Cook Islands |  | 0.8 (0.5 to 1.7) | 19.8 (12.4 to 25.7) | 10.56 (10.34 to 10.77) |
| Costa Rica |  | 6.2 (5.5 to 7.1) | 8.2 (6.4 to 10.9) | 0.99 (0.79 to 1.19) |
| Croatia |  | 1.7 (1.2 to 2.2) | 1.8 (0.8 to 2.8) | 0.84 (0.08 to 1.60) |
| Cuba |  | 1.5 (1.3 to 1.9) | 15.7 (8.7 to 24.8) | 10.17 (9.04 to 11.32) |
| Cyprus |  | 0.5 (0.3 to 0.7) | 1.0 (0.6 to 1.6) | 1.74 (1.34 to 2.14) |
| Czechia |  | 0.3 (0.2 to 0.4) | 2.1 (0.9 to 3.4) | 6.67 (5.56 to 7.79) |
| Democratic Republic of the Congo |  | 216.7 (141.8 to 295.7) | 7.2 (4.2 to 12.2) | -9.63 (-10.31 to -8.95) |
| Djibouti |  | 11.9 (1.6 to 41.1) | 77.3 (26.6 to 171.3) | -0.95 (-3.69 to 1.87) |
| Dominica |  | 20.4 (16.7 to 26.1) | 10.8 (7.8 to 16.4) | -1.89 (-2.72 to -1.06) |
| Dominican Republic |  | 118.8 (74.7 to 173.5) | 15.6 (5.0 to 36.7) | -5.73 (-6.18 to -5.27) |
| Ecuador |  | 3.2 (1.8 to 7.9) | 19.9 (15.7 to 26.6) | 5.30 (4.43 to 6.18) |
| Egypt |  | 0.2 (0.1 to 0.3) | 0.7 (0.4 to 1.3) | 5.18 (4.62 to 5.74) |
| El Salvador |  | 9.2 (6.6 to 12.7) | 9.9 (7.1 to 14.7) | -1.28 (-2.33 to -0.22) |
| Equatorial Guinea |  | 78.8 (23.4 to 195.6) | 571.8 (114.6 to 1545.9) | 4.92 (3.84 to 6.01) |
| Eritrea |  | 184.1 (53.9 to 347.7) | 12.6 (6.2 to 23.2) | -9.54 (-10.02 to -9.05) |
| Estonia |  | 0.7 (0.4 to 1.3) | 20.2 (13.6 to 33.8) | 8.19 (4.42 to 12.10) |
| Ethiopia |  | 175.4 (71.3 to 299.4) | 22.4 (12.9 to 45.8) | -7.56 (-7.98 to -7.14) |
| Fiji |  | 1.4 (0.9 to 3.5) | 4.8 (2.8 to 8.4) | 2.61 (1.71 to 3.51) |
| Gabon |  | 192.0 (96.6 to 345.1) | 121.0 (42.6 to 316.5) | -2.04 (-2.77 to -1.31) |
| Gambia |  | 36.9 (12.2 to 73.3) | 133.3 (55.9 to 259.8) | 1.77 (0.54 to 3.02) |
| Georgia |  | 1.4 (0.3 to 3.9) | 7.3 (4.4 to 13.5) | 9.72 (7.01 to 12.49) |
| Ghana |  | 153.8 (113.2 to 198.6) | 71.9 (40.6 to 121.7) | -3.69 (-4.13 to -3.25) |
| Greece |  | 4.5 (3.1 to 6.3) | 6.4 (4.3 to 10.8) | 2.19 (1.45 to 2.93) |
| Grenada |  | 17.1 (11.5 to 37.1) | 4.5 (3.5 to 6.5) | -3.75 (-4.05 to -3.44) |
| Guinea |  | 67.4 (32.4 to 114.2) | 53.4 (26.5 to 93.6) | -2.05 (-2.85 to -1.24) |
| Guinea-Bissau |  | 58.6 (11.5 to 121.8) | 238.5 (16.9 to 630.0) | 2.85 (2.00 to 3.71) |
| Guyana |  | 42.4 (33.0 to 55.0) | 37.9 (29.5 to 55.5) | -1.95 (-2.91 to -0.97) |
| Hungary |  | 1.0 (0.8 to 1.2) | 3.0 (1.9 to 4.0) | 3.92 (3.25 to 4.59) |
| Indonesia |  | 0.6 (0.2 to 1.0) | 3.4 (2.5 to 5.2) | 7.17 (5.84 to 8.51) |
| Iran |  | 0.2 (0.1 to 0.7) | 2.3 (1.8 to 3.0) | 7.97 (6.82 to 9.13) |
| Iraq |  | 0.1 (0.1 to 0.3) | 0.6 (0.2 to 1.3) | 4.86 (4.20 to 5.52) |
| Italy |  | 14.5 (10.3 to 23.3) | 5.2 (3.4 to 7.1) | -1.90 (-2.37 to -1.43) |
| Jamaica |  | 36.8 (21.1 to 86.5) | 33.6 (25.3 to 46.0) | -0.04 (-0.37 to 0.30) |
| Kazakhstan |  | 10.3 (7.4 to 13.7) | 41.3 (25.4 to 57.3) | 4.35 (3.43 to 5.26) |
| Kenya |  | 1277.8 (1071.9 to 1480.8) | 72.9 (54.2 to 93.6) | -9.14 (-9.55 to -8.74) |
| Kiribati |  | 1.5 (1.2 to 1.9) | 0.7 (0.3 to 1.9) | -2.15 (-2.76 to -1.54) |
| Kuwait |  | 1.3 (0.8 to 1.8) | 0.4 (0.3 to 0.7) | -1.89 (-2.78 to -0.99) |
| Kyrgyzstan |  | 2.1 (1.4 to 2.9) | 39.8 (23.4 to 60.1) | 9.26 (8.53 to 9.99) |
| Lao People's Democratic Republic |  | 0.1 (0.1 to 0.1) | 6.5 (4.8 to 8.6) | 9.59 (4.76 to 14.64) |
| Latvia |  | 10.2 (7.6 to 13.3) | 31.4 (19.8 to 38.1) | 3.76 (3.02 to 4.49) |
| Lebanon |  | 0.8 (0.7 to 1.0) | 3.5 (3.1 to 3.9) | 4.45 (4.08 to 4.82) |
| Lesotho |  | 691.8 (535.5 to 890.9) | 818.4 (534.7 to 1183.3) | -1.87 (-2.92 to -0.80) |
| Liberia |  | 82.3 (22.4 to 191.1) | 39.4 (18.2 to 80.1) | -3.66 (-4.47 to -2.83) |
| Libya |  | 0.7 (0.1 to 2.7) | 1.8 (0.1 to 10.0) | 2.91 (2.03 to 3.79) |
| Lithuania |  | 2.0 (1.6 to 2.6) | 11.2 (6.4 to 18.9) | 5.35 (4.82 to 5.89) |
| Luxembourg |  | 16.9 (12.9 to 21.1) | 7.9 (4.0 to 13.2) | -1.56 (-2.02 to -1.09) |
| Madagascar |  | 0.5 (0.1 to 1.2) | 22.6 (8.4 to 48.9) | 7.35 (4.55 to 10.22) |
| Malaysia |  | 13.1 (8.9 to 18.7) | 13.6 (9.7 to 18.7) | -1.08 (-1.68 to -0.47) |
| Maldives |  | 0.4 (0.3 to 0.6) | 0.5 (0.4 to 0.7) | -0.61 (-1.17 to -0.05) |
| Mali |  | 84.5 (25.8 to 173.9) | 32.3 (15.6 to 60.5) | -4.68 (-5.30 to -4.06) |
| Malta |  | 5.4 (4.8 to 6.3) | 23.5 (11.5 to 39.1) | 6.24 (5.06 to 7.43) |
| Mauritania |  | 0.7 (0.3 to 1.7) | 0.5 (0.1 to 2.5) | -0.55 (-2.27 to 1.20) |
| Micronesia |  | 0.9 (0.5 to 1.7) | 21.1 (12.9 to 27.1) | 10.45 (10.20 to 10.71) |
| Mongolia |  | 0.0 (0.0 to 0.0) | 1.0 (0.3 to 2.4) | 9.44 (6.54 to 12.42) |
| Montenegro |  | 0.4 (0.3 to 0.5) | 0.8 (0.5 to 1.1) | 0.72 (0.22 to 1.21) |
| Morocco |  | 2.4 (1.6 to 3.5) | 1.7 (1.3 to 2.4) | -0.58 (-1.67 to 0.52) |
| Mozambique |  | 210.9 (112.7 to 354.0) | 402.4 (165.1 to 835.1) | 0.95 (-0.19 to 2.11) |
| Myanmar |  | 18.3 (15.6 to 21.3) | 15.1 (11.8 to 19.2) | -2.11 (-3.20 to -1.01) |
| Namibia |  | 397.5 (302.4 to 521.5) | 180.5 (126.0 to 244.0) | -4.73 (-6.06 to -3.39) |
| Nepal |  | 0.0 (0.0 to 0.0) | 2.8 (2.1 to 3.9) | 8.78 (1.09 to 17.06) |
| New Zealand |  | 2.4 (1.9 to 3.0) | 2.2 (1.1 to 3.5) | 1.78 (1.29 to 2.28) |
| Nicaragua |  | 1.5 (1.0 to 2.1) | 12.2 (6.1 to 22.4) | 6.28 (5.68 to 6.88) |
| Niger |  | 53.1 (19.1 to 107.8) | 4.5 (1.5 to 11.5) | -8.86 (-9.62 to -8.11) |
| Nigeria |  | 119.3 (90.9 to 157.3) | 71.6 (58.2 to 90.1) | -3.08 (-3.53 to -2.63) |
| Niue |  | 0.8 (0.5 to 1.7) | 19.5 (12.1 to 25.4) | 10.57 (10.32 to 10.81) |
| North Macedonia |  | 0.0 (0.0 to 0.1) | 0.2 (0.1 to 0.4) | 5.58 (5.16 to 5.99) |
| Oman |  | 0.7 (0.5 to 1.0) | 5.7 (2.3 to 10.9) | 3.54 (2.22 to 4.88) |
| Pakistan |  | 0.0 (0.0 to 0.0) | 10.5 (0.7 to 37.6) | 30.73 (26.37 to 35.24) |
| Panama |  | 32.7 (22.3 to 46.0) | 46.0 (34.8 to 64.2) | 1.20 (0.33 to 2.08) |
| Papua New Guinea |  | 1.1 (0.5 to 2.3) | 35.2 (14.2 to 70.0) | 6.45 (3.28 to 9.72) |
| Peru |  | 8.6 (4.7 to 27.9) | 12.0 (7.6 to 17.4) | 1.65 (1.17 to 2.14) |
| Philippines |  | 0.1 (0.1 to 0.2) | 39.6 (22.2 to 63.2) | 11.64 (9.24 to 14.09) |
| Poland |  | 0.7 (0.4 to 1.2) | 2.7 (1.4 to 4.8) | 3.31 (2.59 to 4.02) |
| Portugal |  | 26.3 (25.1 to 27.5) | 6.9 (6.1 to 7.9) | -5.56 (-6.07 to -5.04) |
| Qatar |  | 1.0 (0.8 to 1.3) | 0.2 (0.2 to 0.3) | -5.66 (-6.63 to -4.68) |
| Republic of Korea |  | 0.4 (0.0 to 1.0) | 1.1 (0.2 to 1.8) | 2.65 (1.10 to 4.23) |
| Republic of Moldova |  | 6.0 (2.3 to 27.3) | 16.6 (11.2 to 23.8) | 2.42 (0.88 to 3.98) |
| Romania |  | 0.9 (0.6 to 1.2) | 5.0 (2.8 to 7.9) | 3.96 (2.65 to 5.29) |
| Russian Federation |  | 4.0 (2.6 to 4.9) | 70.2 (55.9 to 92.3) | 11.43 (10.25 to 12.63) |
| Rwanda |  | 349.8 (108.8 to 684.3) | 55.7 (28.4 to 102.4) | -6.57 (-6.99 to -6.15) |
| Samoa |  | 0.9 (0.5 to 1.7) | 21.0 (13.1 to 27.1) | 10.64 (10.43 to 10.85) |
| Sao Tome and Principe |  | 2.4 (0.9 to 10.4) | 0.9 (0.4 to 1.5) | -5.48 (-7.17 to -3.75) |
| Saudi Arabia |  | 1.5 (0.8 to 3.3) | 2.7 (1.0 to 6.4) | 1.15 (0.51 to 1.78) |
| Senegal |  | 37.6 (21.2 to 59.4) | 14.9 (6.1 to 29.1) | -4.24 (-5.03 to -3.44) |
| Serbia |  | 0.4 (0.0 to 0.6) | 0.4 (0.2 to 0.8) | 1.48 (-0.42 to 3.43) |
| Seychelles |  | 11.1 (7.5 to 16.3) | 3.8 (3.2 to 5.1) | -2.81 (-3.15 to -2.46) |
| Sierra Leone |  | 45.8 (11.6 to 109.3) | 42.0 (18.7 to 83.9) | -1.31 (-2.29 to -0.32) |
| Singapore |  | 23.4 (15.7 to 34.1) | 8.8 (5.4 to 14.1) | -3.35 (-4.56 to -2.13) |
| Slovakia |  | 0.1 (0.1 to 0.1) | 0.3 (0.1 to 0.5) | 3.39 (3.08 to 3.70) |
| Slovenia |  | 1.7 (0.0 to 2.9) | 1.4 (0.8 to 2.5) | -1.68 (-2.42 to -0.93) |
| Solomon Islands |  | 0.9 (0.6 to 1.7) | 21.2 (13.3 to 27.4) | 10.40 (10.16 to 10.64) |
| Somalia |  | 15.1 (2.6 to 49.4) | 17.9 (5.7 to 42.9) | -3.87 (-5.47 to -2.24) |
| South Africa |  | 149.9 (112.2 to 193.1) | 373.3 (270.4 to 488.7) | -1.34 (-3.28 to 0.64) |
| South Sudan |  | 61.4 (8.3 to 200.3) | 99.1 (16.9 to 284.6) | 0.17 (-0.67 to 1.01) |
| Sri Lanka |  | 0.6 (0.1 to 3.2) | 1.1 (0.5 to 2.2) | 1.31 (0.72 to 1.89) |
| Sudan |  | 11.9 (1.1 to 46.2) | 39.5 (3.9 to 145.7) | 2.35 (1.85 to 2.86) |
| Suriname |  | 29.8 (20.2 to 46.7) | 42.6 (28.8 to 64.1) | -0.36 (-1.14 to 0.42) |
| Syrian Arab Republic |  | 0.1 (0.1 to 0.1) | 0.3 (0.2 to 0.5) | 4.09 (3.72 to 4.47) |
| Tajikistan |  | 1.9 (1.3 to 2.8) | 6.3 (3.0 to 9.4) | 2.48 (1.70 to 3.27) |
| Thailand |  | 93.7 (59.0 to 135.3) | 23.9 (15.2 to 39.3) | -3.90 (-5.11 to -2.68) |
| Timor-Leste |  | 27.1 (23.8 to 30.6) | 18.3 (14.8 to 22.3) | -0.92 (-1.36 to -0.49) |
| Togo |  | 205.6 (117.0 to 323.9) | 38.1 (19.3 to 66.5) | -6.33 (-7.06 to -5.59) |
| Tonga |  | 0.9 (0.5 to 1.7) | 23.9 (15.5 to 30.9) | 11.10 (10.90 to 11.30) |
| Trinidad and Tobago |  | 56.0 (41.0 to 76.9) | 87.8 (72.2 to 112.3) | 0.38 (-0.20 to 0.97) |
| Tunisia |  | 0.1 (0.0 to 0.3) | 0.9 (0.5 to 1.5) | 7.77 (6.22 to 9.35) |
| Turkey |  | 0.1 (0.0 to 0.2) | 0.5 (0.4 to 0.8) | 5.67 (4.48 to 6.88) |
| Uganda |  | 683.7 (613.6 to 774.7) | 153.5 (77.1 to 282.9) | -3.56 (-3.95 to -3.17) |
| Ukraine |  | 7.8 (5.9 to 12.3) | 87.4 (63.7 to 120.4) | 5.80 (4.15 to 7.48) |
| United Arab Emirates |  | 0.5 (0.3 to 0.9) | 1.7 (1.1 to 2.4) | 2.98 (1.95 to 4.02) |
| United Republic of Tanzania |  | 797.7 (600.6 to 1016.8) | 57.5 (22.5 to 120.2) | -6.34 (-7.27 to -5.41) |
| Uruguay |  | 11.6 (4.9 to 36.0) | 28.5 (19.3 to 38.6) | 1.92 (1.12 to 2.72) |
| Uzbekistan |  | 2.8 (1.0 to 5.2) | 23.0 (7.6 to 38.6) | 6.59 (5.41 to 7.78) |
| Vanuatu |  | 0.8 (0.5 to 1.7) | 20.9 (12.8 to 27.0) | 10.55 (10.30 to 10.79) |
| Venezuela |  | 9.5 (7.4 to 12.3) | 22.9 (17.5 to 29.1) | 1.70 (1.25 to 2.15) |
| Viet Nam |  | 5.4 (4.1 to 6.9) | 14.1 (11.2 to 18.5) | 2.63 (1.51 to 3.77) |
| Yemen |  | 0.6 (0.2 to 1.3) | 4.6 (1.9 to 8.0) | 7.08 (6.29 to 7.89) |
| Zambia |  | 1463.9 (1210.7 to 1771.5) | 205.7 (119.3 to 331.4) | -5.48 (-5.81 to -5.15) |
| Zimbabwe |  | 2495.6 (2184.3 to 2859.1) | 138.7 (95.2 to 199.7) | -8.35 (-8.83 to -7.87) |

BRI countries: the Belt and Road countries; EAPC: the estimated annual percentage change.

| Supplementary Table 13: Tuberculosis incidence rate (/100,000 persons) in 149 BRI Countries between 1990 and 2021 | | | | |
| --- | --- | --- | --- | --- |
| Country |  | 1990 | 2021 | EAPC during 1990-2021 (95% CI) (%) |
| Afghanistan |  | 310.7 (279.8 to 342.8) | 164.5 (144.1 to 188.8) | -2.17 (-2.51 to -1.82) |
| Albania |  | 25.3 (22.6 to 28.2) | 10.3 (8.9 to 11.9) | -3.14 (-3.22 to -3.05) |
| Algeria |  | 121.6 (106.1 to 138.9) | 44.8 (38.8 to 51.2) | -3.23 (-3.28 to -3.18) |
| Angola |  | 536.0 (480.6 to 594.8) | 320.0 (285.7 to 359.3) | -1.67 (-1.84 to -1.51) |
| Antigua and Barbuda |  | 25.9 (22.5 to 29.7) | 12.5 (10.9 to 14.7) | -2.24 (-2.33 to -2.15) |
| Argentina |  | 25.8 (22.5 to 29.8) | 13.9 (11.9 to 16.2) | -1.88 (-2.08 to -1.68) |
| Armenia |  | 42.2 (36.6 to 48.2) | 22.3 (19.3 to 25.9) | -1.84 (-2.31 to -1.38) |
| Austria |  | 12.7 (10.8 to 15.1) | 5.1 (4.3 to 6.0) | -3.08 (-3.23 to -2.93) |
| Azerbaijan |  | 88.0 (78.1 to 99.3) | 50.6 (43.3 to 60.0) | -2.10 (-2.42 to -1.77) |
| Bahrain |  | 36.7 (32.2 to 41.3) | 15.2 (13.1 to 17.5) | -3.38 (-3.69 to -3.07) |
| Bangladesh |  | 524.9 (458.4 to 598.9) | 140.7 (123.1 to 161.9) | -4.17 (-4.24 to -4.11) |
| Barbados |  | 10.0 (8.6 to 11.5) | 4.4 (3.8 to 5.2) | -2.58 (-2.73 to -2.44) |
| Belarus |  | 49.9 (42.6 to 59.0) | 21.4 (18.1 to 25.3) | -2.74 (-3.31 to -2.17) |
| Benin |  | 256.7 (232.2 to 282.4) | 119.6 (103.6 to 136.4) | -2.47 (-2.74 to -2.20) |
| Bolivia |  | 239.5 (215.2 to 267.8) | 73.0 (64.2 to 83.6) | -4.13 (-4.26 to -4.00) |
| Bosnia and Herzegovina |  | 46.4 (40.8 to 52.5) | 18.3 (15.8 to 20.9) | -3.33 (-3.49 to -3.17) |
| Botswana |  | 338.4 (293.7 to 391.7) | 286.7 (244.9 to 341.9) | -1.63 (-2.30 to -0.95) |
| Brunei Darussalam |  | 119.8 (108.7 to 132.7) | 59.8 (51.5 to 68.3) | -2.50 (-2.64 to -2.36) |
| Bulgaria |  | 23.9 (20.7 to 27.2) | 13.3 (11.5 to 15.5) | -2.01 (-2.45 to -1.57) |
| Burkina Faso |  | 308.4 (279.5 to 339.5) | 172.7 (149.9 to 196.6) | -1.83 (-2.10 to -1.55) |
| Burundi |  | 690.6 (622.3 to 774.0) | 366.1 (320.3 to 415.1) | -2.52 (-2.78 to -2.26) |
| Côte d’Ivoire |  | 356.1 (320.6 to 395.6) | 162.3 (141.1 to 185.7) | -2.25 (-2.47 to -2.03) |
| Cabo Verde |  | 216.6 (192.7 to 243.7) | 85.1 (73.7 to 97.3) | -3.09 (-3.19 to -2.98) |
| Cambodia |  | 683.0 (628.2 to 737.6) | 267.5 (238.8 to 297.9) | -3.11 (-3.23 to -2.98) |
| Cameroon |  | 229.8 (208.6 to 253.5) | 144.2 (124.7 to 165.7) | -1.74 (-2.34 to -1.14) |
| Central African Republic |  | 732.5 (661.8 to 803.7) | 596.0 (543.9 to 649.6) | -0.48 (-0.56 to -0.40) |
| Chad |  | 390.8 (354.2 to 433.4) | 237.9 (209.4 to 269.4) | -1.41 (-1.59 to -1.24) |
| Chile |  | 32.3 (28.2 to 36.6) | 11.1 (9.6 to 12.9) | -3.60 (-3.84 to -3.36) |
| China |  | 109.0 (94.8 to 124.6) | 36.3 (32.6 to 40.5) | -3.78 (-3.90 to -3.66) |
| Comoros |  | 536.9 (484.6 to 590.2) | 261.5 (226.3 to 301.2) | -2.48 (-2.58 to -2.37) |
| Congo |  | 459.1 (412.5 to 510.1) | 268.6 (238.8 to 301.9) | -1.71 (-1.89 to -1.52) |
| Cook Islands |  | 24.1 (21.5 to 27.5) | 12.3 (10.6 to 14.1) | -2.26 (-2.33 to -2.20) |
| Costa Rica |  | 22.7 (19.8 to 25.8) | 7.5 (6.5 to 8.7) | -3.98 (-4.13 to -3.82) |
| Croatia |  | 30.4 (26.4 to 35.3) | 7.0 (6.1 to 8.0) | -5.25 (-5.49 to -5.00) |
| Cuba |  | 11.0 (9.6 to 12.6) | 4.4 (3.8 to 5.1) | -3.17 (-3.29 to -3.05) |
| Cyprus |  | 11.8 (10.2 to 13.7) | 3.6 (3.1 to 4.2) | -3.80 (-3.93 to -3.67) |
| Czechia |  | 13.4 (11.5 to 15.3) | 3.7 (3.3 to 4.4) | -4.57 (-4.76 to -4.38) |
| Democratic Republic of the Congo |  | 549.0 (494.1 to 607.2) | 422.6 (377.9 to 470.7) | -0.68 (-0.89 to -0.46) |
| Djibouti |  | 510.0 (465.0 to 555.2) | 249.6 (216.5 to 283.9) | -2.25 (-2.46 to -2.04) |
| Dominica |  | 33.2 (29.6 to 37.1) | 16.1 (14.1 to 18.4) | -2.36 (-2.48 to -2.23) |
| Dominican Republic |  | 94.4 (85.2 to 105.2) | 44.9 (39.1 to 51.5) | -2.35 (-2.54 to -2.15) |
| Ecuador |  | 120.9 (106.5 to 138.1) | 41.3 (35.7 to 47.8) | -4.34 (-4.61 to -4.06) |
| Egypt |  | 29.6 (27.1 to 32.1) | 10.0 (8.9 to 11.2) | -2.96 (-3.20 to -2.72) |
| El Salvador |  | 100.7 (90.9 to 110.9) | 35.5 (30.9 to 40.9) | -3.50 (-3.70 to -3.30) |
| Equatorial Guinea |  | 604.7 (546.6 to 670.7) | 159.6 (139.1 to 181.8) | -4.77 (-5.00 to -4.54) |
| Eritrea |  | 959.2 (868.7 to 1056.4) | 537.3 (470.7 to 608.3) | -1.74 (-1.83 to -1.65) |
| Estonia |  | 45.3 (38.7 to 53.1) | 11.4 (9.7 to 13.4) | -5.21 (-5.67 to -4.74) |
| Ethiopia |  | 750.8 (664.8 to 848.1) | 270.0 (241.8 to 297.3) | -3.60 (-3.72 to -3.48) |
| Fiji |  | 66.5 (59.9 to 74.2) | 35.0 (31.8 to 38.8) | -2.25 (-2.40 to -2.10) |
| Gabon |  | 260.1 (229.3 to 293.4) | 183.9 (162.3 to 209.9) | -1.24 (-1.62 to -0.87) |
| Gambia |  | 467.7 (418.2 to 524.0) | 225.7 (194.7 to 260.6) | -2.14 (-2.25 to -2.02) |
| Georgia |  | 56.6 (49.1 to 65.1) | 51.2 (44.2 to 60.0) | 0.16 (-0.39 to 0.71) |
| Ghana |  | 364.9 (331.1 to 399.0) | 196.3 (170.8 to 222.6) | -1.64 (-1.84 to -1.45) |
| Greece |  | 7.4 (6.4 to 8.8) | 3.2 (2.8 to 3.8) | -2.44 (-2.59 to -2.29) |
| Grenada |  | 16.4 (14.5 to 18.5) | 7.9 (6.8 to 9.2) | -2.35 (-2.43 to -2.27) |
| Guinea |  | 241.4 (221.0 to 266.0) | 167.3 (147.0 to 190.4) | -0.76 (-1.11 to -0.41) |
| Guinea-Bissau |  | 456.8 (412.2 to 515.9) | 223.6 (196.2 to 252.9) | -2.03 (-2.13 to -1.94) |
| Guyana |  | 61.8 (55.8 to 67.7) | 40.0 (34.5 to 46.2) | -0.70 (-1.01 to -0.39) |
| Hungary |  | 20.6 (17.9 to 23.4) | 4.9 (4.2 to 5.6) | -5.11 (-5.42 to -4.79) |
| Indonesia |  | 353.0 (318.7 to 392.0) | 187.8 (168.6 to 205.6) | -2.20 (-2.32 to -2.07) |
| Iran |  | 22.6 (20.1 to 25.4) | 13.5 (12.0 to 15.1) | -1.55 (-1.64 to -1.46) |
| Iraq |  | 74.7 (66.7 to 84.2) | 27.3 (23.7 to 31.4) | -3.47 (-3.66 to -3.27) |
| Italy |  | 12.0 (10.1 to 14.5) | 6.0 (5.0 to 7.3) | -2.26 (-2.39 to -2.13) |
| Jamaica |  | 8.1 (7.1 to 9.3) | 3.2 (2.8 to 3.7) | -3.08 (-3.19 to -2.97) |
| Kazakhstan |  | 118.2 (102.3 to 136.4) | 53.0 (45.8 to 62.2) | -3.09 (-3.86 to -2.31) |
| Kenya |  | 394.2 (345.6 to 445.2) | 240.1 (210.3 to 268.1) | -1.85 (-2.25 to -1.44) |
| Kiribati |  | 424.8 (391.7 to 462.8) | 341.7 (313.1 to 371.6) | -0.77 (-0.83 to -0.71) |
| Kuwait |  | 39.9 (34.5 to 45.8) | 17.7 (15.3 to 20.3) | -2.77 (-2.88 to -2.66) |
| Kyrgyzstan |  | 104.0 (91.8 to 117.6) | 81.6 (70.7 to 94.5) | -0.67 (-0.96 to -0.37) |
| Lao People’s Democratic Republic |  | 483.0 (421.5 to 552.4) | 125.7 (109.4 to 143.4) | -4.70 (-4.87 to -4.53) |
| Latvia |  | 58.5 (50.2 to 67.8) | 22.0 (18.7 to 26.2) | -3.49 (-3.90 to -3.07) |
| Lebanon |  | 34.9 (30.6 to 40.0) | 12.0 (10.5 to 13.9) | -3.55 (-3.60 to -3.49) |
| Lesotho |  | 545.3 (485.0 to 628.8) | 513.9 (448.2 to 588.6) | 0.76 (0.44 to 1.09) |
| Liberia |  | 415.9 (377.9 to 453.9) | 154.7 (135.4 to 176.5) | -3.68 (-3.90 to -3.46) |
| Libya |  | 56.5 (49.7 to 64.2) | 29.2 (25.3 to 33.6) | -2.21 (-2.36 to -2.07) |
| Lithuania |  | 68.1 (58.5 to 78.9) | 31.9 (27.3 to 37.7) | -2.47 (-2.80 to -2.14) |
| Luxembourg |  | 15.2 (12.6 to 18.6) | 7.4 (6.2 to 9.0) | -2.12 (-2.26 to -1.98) |
| Madagascar |  | 619.4 (563.1 to 683.4) | 302.8 (265.2 to 346.4) | -1.97 (-2.07 to -1.86) |
| Malaysia |  | 117.5 (103.9 to 131.9) | 60.0 (52.4 to 67.9) | -2.40 (-2.52 to -2.27) |
| Maldives |  | 193.0 (172.4 to 215.5) | 55.5 (48.0 to 62.9) | -4.63 (-4.88 to -4.38) |
| Mali |  | 383.8 (349.5 to 423.6) | 183.4 (159.5 to 207.3) | -2.24 (-2.34 to -2.14) |
| Malta |  | 33.6 (27.9 to 40.8) | 23.7 (19.0 to 29.1) | -0.57 (-0.88 to -0.26) |
| Mauritania |  | 234.1 (212.3 to 259.4) | 80.1 (69.6 to 92.0) | -3.29 (-3.35 to -3.22) |
| Micronesia |  | 139.4 (126.8 to 153.1) | 82.9 (74.9 to 90.6) | -1.65 (-1.75 to -1.55) |
| Mongolia |  | 210.6 (190.2 to 233.7) | 99.9 (87.5 to 114.5) | -2.36 (-2.71 to -2.02) |
| Montenegro |  | 16.7 (14.4 to 19.3) | 8.6 (7.4 to 10.0) | -2.49 (-2.76 to -2.22) |
| Morocco |  | 168.9 (151.6 to 188.1) | 70.5 (61.0 to 80.7) | -2.85 (-2.96 to -2.73) |
| Mozambique |  | 639.8 (577.2 to 717.9) | 408.2 (358.0 to 466.2) | -1.01 (-1.16 to -0.86) |
| Myanmar |  | 606.4 (551.8 to 661.6) | 162.1 (146.7 to 178.5) | -4.22 (-4.30 to -4.15) |
| Namibia |  | 680.4 (604.3 to 774.3) | 337.1 (294.5 to 385.4) | -1.86 (-2.05 to -1.66) |
| Nepal |  | 475.8 (433.0 to 517.2) | 157.4 (137.2 to 179.8) | -3.66 (-3.71 to -3.62) |
| New Zealand |  | 11.7 (10.1 to 13.8) | 6.6 (5.4 to 7.9) | -1.85 (-2.35 to -1.35) |
| Nicaragua |  | 84.1 (76.0 to 92.8) | 31.1 (27.1 to 35.5) | -3.49 (-3.59 to -3.39) |
| Niger |  | 432.2 (392.6 to 474.2) | 226.7 (198.7 to 255.8) | -2.07 (-2.12 to -2.02) |
| Nigeria |  | 389.2 (350.2 to 429.9) | 178.2 (156.3 to 201.2) | -2.71 (-3.15 to -2.27) |
| Niue |  | 49.0 (43.7 to 54.8) | 31.1 (27.6 to 34.7) | -1.62 (-1.68 to -1.57) |
| North Macedonia |  | 32.2 (28.6 to 36.2) | 12.8 (11.2 to 14.7) | -3.40 (-3.58 to -3.22) |
| Oman |  | 30.1 (26.5 to 34.4) | 10.1 (8.8 to 11.6) | -3.50 (-3.59 to -3.41) |
| Pakistan |  | 415.9 (364.4 to 474.8) | 182.8 (161.6 to 208.5) | -2.83 (-3.04 to -2.61) |
| Panama |  | 59.5 (52.9 to 66.7) | 30.2 (26.3 to 35.4) | -1.95 (-2.07 to -1.84) |
| Papua New Guinea |  | 197.5 (179.7 to 217.5) | 142.1 (129.8 to 155.2) | -1.07 (-1.12 to -1.02) |
| Peru |  | 224.7 (196.5 to 256.0) | 68.3 (59.0 to 80.1) | -4.31 (-4.86 to -3.76) |
| Philippines |  | 265.0 (236.6 to 297.4) | 341.0 (307.7 to 377.6) | 0.82 (0.49 to 1.14) |
| Poland |  | 34.0 (29.6 to 39.0) | 10.9 (9.3 to 12.8) | -3.98 (-4.15 to -3.82) |
| Portugal |  | 32.8 (27.5 to 38.8) | 10.3 (8.8 to 12.3) | -3.80 (-3.94 to -3.67) |
| Qatar |  | 109.5 (95.1 to 123.7) | 32.2 (27.9 to 36.7) | -4.10 (-4.28 to -3.92) |
| Republic of Korea |  | 135.9 (124.1 to 148.1) | 37.8 (32.6 to 43.2) | -3.52 (-3.76 to -3.28) |
| Republic of Moldova |  | 74.8 (65.2 to 85.4) | 53.0 (45.1 to 62.1) | -0.36 (-0.85 to 0.12) |
| Romania |  | 72.6 (64.2 to 81.7) | 37.2 (32.3 to 43.2) | -2.26 (-2.58 to -1.95) |
| Russian Federation |  | 114.9 (99.5 to 134.3) | 61.1 (51.1 to 74.9) | -2.86 (-3.78 to -1.94) |
| Rwanda |  | 536.4 (487.0 to 612.8) | 190.9 (167.0 to 216.0) | -3.51 (-3.64 to -3.38) |
| Samoa |  | 67.0 (59.7 to 74.8) | 47.0 (42.4 to 52.6) | -1.18 (-1.23 to -1.13) |
| Sao Tome and Principe |  | 186.2 (166.9 to 208.0) | 78.6 (67.8 to 90.5) | -2.69 (-2.90 to -2.47) |
| Saudi Arabia |  | 119.0 (108.8 to 130.4) | 41.5 (36.3 to 47.8) | -3.40 (-3.51 to -3.30) |
| Senegal |  | 282.2 (254.3 to 312.8) | 140.2 (122.2 to 159.3) | -2.42 (-2.62 to -2.22) |
| Serbia |  | 21.0 (18.1 to 24.1) | 8.7 (7.6 to 10.1) | -3.20 (-3.39 to -3.00) |
| Seychelles |  | 51.8 (45.6 to 58.1) | 30.9 (27.1 to 34.9) | -1.91 (-1.99 to -1.82) |
| Sierra Leone |  | 449.3 (407.7 to 496.8) | 251.3 (220.7 to 288.3) | -1.86 (-2.31 to -1.41) |
| Singapore |  | 73.5 (64.9 to 81.6) | 27.5 (23.6 to 31.7) | -3.09 (-3.36 to -2.82) |
| Slovakia |  | 12.3 (10.8 to 14.0) | 4.4 (3.9 to 5.2) | -3.47 (-3.54 to -3.41) |
| Slovenia |  | 18.1 (15.6 to 20.6) | 4.4 (3.8 to 5.1) | -5.24 (-5.51 to -4.96) |
| Solomon Islands |  | 103.9 (94.4 to 114.1) | 79.0 (72.3 to 86.1) | -0.89 (-0.91 to -0.86) |
| Somalia |  | 798.4 (702.1 to 903.1) | 658.7 (568.4 to 753.7) | -0.57 (-0.69 to -0.44) |
| South Africa |  | 561.6 (500.2 to 625.1) | 458.1 (403.1 to 517.2) | -0.12 (-0.33 to 0.10) |
| South Sudan |  | 593.1 (533.6 to 660.8) | 433.5 (381.3 to 493.8) | -0.97 (-1.06 to -0.89) |
| Sri Lanka |  | 67.7 (59.0 to 77.6) | 45.5 (39.7 to 51.5) | -1.78 (-1.96 to -1.60) |
| Sudan |  | 122.8 (111.0 to 136.4) | 47.7 (41.7 to 54.5) | -3.08 (-3.17 to -2.99) |
| Suriname |  | 29.4 (26.3 to 33.0) | 14.2 (12.5 to 16.3) | -2.53 (-2.63 to -2.44) |
| Syrian Arab Republic |  | 24.7 (21.9 to 27.8) | 10.3 (8.9 to 11.9) | -2.90 (-3.20 to -2.60) |
| Tajikistan |  | 119.3 (106.2 to 134.0) | 54.9 (48.0 to 63.3) | -2.73 (-3.12 to -2.34) |
| Thailand |  | 202.4 (177.1 to 229.1) | 115.7 (100.2 to 131.7) | -1.88 (-1.98 to -1.79) |
| Timor-Leste |  | 513.0 (464.4 to 566.8) | 339.5 (308.1 to 372.4) | -1.65 (-1.79 to -1.51) |
| Togo |  | 385.1 (349.8 to 425.1) | 198.9 (173.1 to 227.2) | -1.84 (-2.05 to -1.63) |
| Tonga |  | 48.0 (43.2 to 53.4) | 34.5 (30.7 to 38.1) | -1.10 (-1.13 to -1.06) |
| Trinidad and Tobago |  | 17.5 (15.5 to 19.6) | 8.7 (7.4 to 10.2) | -2.10 (-2.21 to -1.98) |
| Tunisia |  | 75.1 (65.9 to 84.8) | 22.6 (19.6 to 26.2) | -4.08 (-4.19 to -3.97) |
| Turkey |  | 49.1 (44.3 to 54.4) | 12.0 (10.2 to 13.9) | -5.04 (-5.22 to -4.86) |
| Uganda |  | 386.0 (351.7 to 420.1) | 236.1 (214.6 to 258.3) | -1.89 (-2.12 to -1.67) |
| Ukraine |  | 70.1 (59.6 to 82.4) | 59.7 (50.2 to 72.1) | -0.45 (-1.03 to 0.13) |
| United Arab Emirates |  | 20.6 (18.8 to 22.8) | 8.0 (6.9 to 9.2) | -3.22 (-3.34 to -3.10) |
| United Republic of Tanzania |  | 458.8 (400.3 to 522.4) | 188.7 (163.2 to 215.8) | -3.40 (-3.87 to -2.94) |
| Uruguay |  | 31.9 (27.7 to 36.5) | 22.9 (19.3 to 27.2) | -0.62 (-0.94 to -0.31) |
| Uzbekistan |  | 87.9 (77.0 to 100.7) | 50.1 (43.4 to 57.5) | -1.74 (-2.07 to -1.42) |
| Vanuatu |  | 86.0 (77.8 to 95.3) | 64.0 (58.7 to 69.8) | -0.96 (-0.98 to -0.94) |
| Venezuela |  | 42.5 (37.7 to 47.7) | 22.8 (20.0 to 26.1) | -2.23 (-2.55 to -1.91) |
| Viet Nam |  | 247.6 (228.1 to 266.5) | 150.3 (137.5 to 162.9) | -1.64 (-1.68 to -1.60) |
| Yemen |  | 60.6 (54.3 to 67.5) | 26.8 (23.4 to 30.4) | -2.56 (-2.71 to -2.40) |
| Zambia |  | 399.0 (356.4 to 441.2) | 236.9 (202.3 to 271.8) | -2.92 (-3.57 to -2.26) |
| Zimbabwe |  | 481.4 (410.4 to 568.4) | 322.8 (277.2 to 373.6) | -0.75 (-1.06 to -0.44) |

BRI countries: the Belt and Road countries; EAPC: the estimated annual percentage change.

| Supplementary Table 14: Malaria incidence rate (/100,000 persons) in 149 BRI Countries between 1990 and 2021 | | | | |
| --- | --- | --- | --- | --- |
| Country |  | 1990 | 2021 | EAPC during 1990-2021 (95% CI) (%) |
| Afghanistan |  | 8460.8 (6449.7 to 10824.5) | 772.7 (624.9 to 950.4) | -5.59 (-7.04 to -4.13) |
| Albania |  | 0.0 (0.0 to 0.0) | 0.0 (0.0 to 0.0) | 0.00 (0.00 to 0.00) |
| Algeria |  | 10.1 (1.7 to 34.1) | 0.0 (0.0 to 0.0) | -26.41 (-28.64 to -24.10) |
| Angola |  | 22904.4 (14778.8 to 32254.2) | 17668.9 (10760.8 to 26623.1) | -1.97 (-2.63 to -1.31) |
| Antigua and Barbuda |  | 0.0 (0.0 to 0.0) | 0.0 (0.0 to 0.0) | 0.00 (0.00 to 0.00) |
| Argentina |  | 5.1 (5.1 to 5.1) | 0.0 (0.0 to 0.0) | -28.19 (-32.05 to -24.12) |
| Armenia |  | 0.0 (0.0 to 0.0) | 0.0 (0.0 to 0.0) | -19.17 (-31.37 to -4.79) |
| Austria |  | 0.0 (0.0 to 0.0) | 0.0 (0.0 to 0.0) | 0.00 (0.00 to 0.00) |
| Azerbaijan |  | 2.2 (1.4 to 3.1) | 0.0 (0.0 to 0.0) | -35.38 (-41.64 to -28.43) |
| Bahrain |  | 0.0 (0.0 to 0.0) | 0.0 (0.0 to 0.0) | 0.00 (0.00 to 0.00) |
| Bangladesh |  | 1384.0 (377.4 to 3735.3) | 34.4 (30.4 to 39.0) | -10.09 (-11.60 to -8.56) |
| Barbados |  | 0.0 (0.0 to 0.0) | 0.0 (0.0 to 0.0) | 0.00 (0.00 to 0.00) |
| Belarus |  | 0.0 (0.0 to 0.0) | 0.0 (0.0 to 0.0) | 0.00 (0.00 to 0.00) |
| Benin |  | 29656.3 (22106.5 to 35835.1) | 27371.1 (18769.5 to 35280.9) | -0.30 (-0.57 to -0.04) |
| Bolivia |  | 2390.2 (1100.6 to 4479.1) | 63.7 (47.8 to 83.4) | -9.89 (-10.85 to -8.92) |
| Bosnia and Herzegovina |  | 0.0 (0.0 to 0.0) | 0.0 (0.0 to 0.0) | 0.00 (0.00 to 0.00) |
| Botswana |  | 71.6 (62.4 to 82.8) | 120.2 (102.2 to 140.0) | -4.25 (-7.24 to -1.15) |
| Brunei Darussalam |  | 0.0 (0.0 to 0.0) | 0.0 (0.0 to 0.0) | 0.00 (0.00 to 0.00) |
| Bulgaria |  | 0.0 (0.0 to 0.0) | 0.0 (0.0 to 0.0) | 0.00 (0.00 to 0.00) |
| Burkina Faso |  | 40028.7 (32231.3 to 50037.2) | 26759.1 (17858.4 to 37140.7) | -1.51 (-1.83 to -1.18) |
| Burundi |  | 31670.5 (22726.3 to 39192.3) | 21282.8 (13890.4 to 29788.5) | -2.74 (-3.67 to -1.81) |
| Côte d’Ivoire |  | 35750.2 (29221.6 to 45248.9) | 20865.9 (10810.8 to 30262.4) | -2.28 (-2.84 to -1.72) |
| Cabo Verde |  | 1.8 (1.3 to 2.5) | 8.9 (7.4 to 10.5) | 0.40 (-9.11 to 10.91) |
| Cambodia |  | 7227.5 (6152.9 to 8402.2) | 95.1 (79.7 to 112.0) | -7.96 (-9.56 to -6.33) |
| Cameroon |  | 29646.9 (23222.2 to 36289.9) | 18889.8 (12234.4 to 27349.7) | -1.96 (-2.31 to -1.60) |
| Central African Republic |  | 30183.3 (19839.4 to 39601.3) | 23265.7 (13120.0 to 36698.7) | -0.85 (-1.07 to -0.62) |
| Chad |  | 21176.0 (13974.5 to 29887.0) | 14141.1 (8561.4 to 21916.1) | -1.56 (-1.78 to -1.33) |
| Chile |  | 0.0 (0.0 to 0.0) | 0.0 (0.0 to 0.0) | 0.00 (0.00 to 0.00) |
| China |  | 24.5 (20.8 to 32.1) | 0.0 (0.0 to 0.0) | -29.83 (-33.84 to -25.58) |
| Comoros |  | 18092.7 (379.9 to 89671.9) | 1687.9 (1395.0 to 2031.5) | -6.94 (-10.08 to -3.69) |
| Congo |  | 26102.8 (18958.9 to 34986.7) | 18817.4 (12065.1 to 29733.9) | -1.67 (-2.08 to -1.27) |
| Cook Islands |  | 0.0 (0.0 to 0.0) | 0.0 (0.0 to 0.0) | 0.00 (0.00 to 0.00) |
| Costa Rica |  | 96.7 (79.5 to 115.2) | 7.1 (7.1 to 7.1) | -24.97 (-31.22 to -18.16) |
| Croatia |  | 0.0 (0.0 to 0.0) | 0.0 (0.0 to 0.0) | 0.00 (0.00 to 0.00) |
| Cuba |  | 0.0 (0.0 to 0.0) | 0.0 (0.0 to 0.0) | 0.00 (0.00 to 0.00) |
| Cyprus |  | 0.0 (0.0 to 0.0) | 0.0 (0.0 to 0.0) | 0.00 (0.00 to 0.00) |
| Czechia |  | 0.0 (0.0 to 0.0) | 0.0 (0.0 to 0.0) | 0.00 (0.00 to 0.00) |
| Democratic Republic of the Congo |  | 32243.3 (25733.0 to 40857.9) | 22589.0 (17024.3 to 28769.4) | -1.47 (-1.79 to -1.15) |
| Djibouti |  | 3400.1 (2968.0 to 3942.9) | 9947.7 (9195.7 to 10761.6) | -0.17 (-7.30 to 7.51) |
| Dominica |  | 0.0 (0.0 to 0.0) | 0.0 (0.0 to 0.0) | 0.00 (0.00 to 0.00) |
| Dominican Republic |  | 18.1 (14.3 to 22.8) | 20.6 (5.2 to 56.7) | -4.94 (-7.39 to -2.43) |
| Ecuador |  | 2020.8 (1656.5 to 2476.4) | 35.9 (28.9 to 44.1) | -16.61 (-20.10 to -12.96) |
| Egypt |  | 0.0 (0.0 to 0.0) | 0.0 (0.0 to 0.0) | 0.00 (0.00 to 0.00) |
| El Salvador |  | 945.6 (689.4 to 1270.1) | 0.0 (0.0 to 0.0) | -35.84 (-38.78 to -32.76) |
| Equatorial Guinea |  | 22065.4 (13683.0 to 29691.6) | 17045.5 (8015.4 to 26554.0) | -0.59 (-0.83 to -0.35) |
| Eritrea |  | 2285.0 (555.5 to 6199.7) | 714.9 (673.0 to 759.6) | -2.54 (-4.96 to -0.06) |
| Estonia |  | 0.0 (0.0 to 0.0) | 0.0 (0.0 to 0.0) | 0.00 (0.00 to 0.00) |
| Ethiopia |  | 2218.3 (1525.2 to 3234.7) | 3047.6 (2507.6 to 3576.3) | 2.35 (-0.30 to 5.07) |
| Fiji |  | 0.0 (0.0 to 0.0) | 0.0 (0.0 to 0.0) | 0.00 (0.00 to 0.00) |
| Gabon |  | 25961.2 (17175.1 to 36489.4) | 19918.3 (9676.8 to 34898.2) | -1.31 (-2.20 to -0.41) |
| Gambia |  | 12679.2 (10538.9 to 15085.0) | 6278.3 (5506.8 to 7167.7) | 0.02 (-1.64 to 1.71) |
| Georgia |  | 0.0 (0.0 to 0.0) | 0.0 (0.0 to 0.0) | -10.90 (-24.48 to 5.12) |
| Ghana |  | 33959.5 (25309.7 to 40596.4) | 13998.9 (9902.1 to 19710.9) | -2.68 (-3.26 to -2.09) |
| Greece |  | 0.0 (0.0 to 0.0) | 0.0 (0.0 to 0.0) | 0.00 (0.00 to 0.00) |
| Grenada |  | 0.0 (0.0 to 0.0) | 0.0 (0.0 to 0.0) | 0.00 (0.00 to 0.00) |
| Guinea |  | 32052.1 (24912.2 to 41799.2) | 24017.7 (13371.4 to 33220.0) | -0.87 (-1.13 to -0.61) |
| Guinea-Bissau |  | 28932.1 (19078.7 to 43203.2) | 8353.1 (2630.0 to 18310.4) | -6.09 (-7.24 to -4.93) |
| Guyana |  | 13155.8 (10857.1 to 15816.0) | 11875.4 (9785.3 to 14345.3) | -3.37 (-4.94 to -1.78) |
| Hungary |  | 0.0 (0.0 to 0.0) | 0.0 (0.0 to 0.0) | 0.00 (0.00 to 0.00) |
| Indonesia |  | 1483.2 (606.4 to 3172.2) | 411.0 (350.8 to 480.4) | -4.93 (-5.54 to -4.32) |
| Iran |  | 504.6 (398.6 to 617.8) | 1.1 (1.1 to 1.2) | -26.35 (-29.11 to -23.48) |
| Iraq |  | 100.7 (72.8 to 136.3) | 0.0 (0.0 to 0.0) | -40.61 (-45.32 to -35.49) |
| Italy |  | 0.0 (0.0 to 0.0) | 0.0 (0.0 to 0.0) | 0.00 (0.00 to 0.00) |
| Jamaica |  | 0.0 (0.0 to 0.0) | 0.0 (0.0 to 0.0) | 0.00 (0.00 to 0.00) |
| Kazakhstan |  | 0.0 (0.0 to 0.0) | 0.0 (0.0 to 0.0) | 0.00 (0.00 to 0.00) |
| Kenya |  | 17005.7 (13370.8 to 22050.0) | 5426.3 (3918.3 to 6956.7) | -5.24 (-6.12 to -4.35) |
| Kiribati |  | 0.0 (0.0 to 0.0) | 0.0 (0.0 to 0.0) | 0.00 (0.00 to 0.00) |
| Kuwait |  | 0.0 (0.0 to 0.0) | 0.0 (0.0 to 0.0) | 0.00 (0.00 to 0.00) |
| Kyrgyzstan |  | 0.0 (0.0 to 0.0) | 0.0 (0.0 to 0.0) | -6.98 (-20.83 to 9.30) |
| Lao People’s Democratic Republic |  | 1922.0 (1534.1 to 2370.3) | 118.8 (99.2 to 140.2) | -8.91 (-10.78 to -7.00) |
| Latvia |  | 0.0 (0.0 to 0.0) | 0.0 (0.0 to 0.0) | 0.00 (0.00 to 0.00) |
| Lebanon |  | 0.0 (0.0 to 0.0) | 0.0 (0.0 to 0.0) | 0.00 (0.00 to 0.00) |
| Lesotho |  | 0.0 (0.0 to 0.0) | 0.0 (0.0 to 0.0) | 0.00 (0.00 to 0.00) |
| Liberia |  | 32053.9 (18177.3 to 44088.2) | 27702.7 (14565.4 to 38887.5) | -0.74 (-1.09 to -0.39) |
| Libya |  | 0.0 (0.0 to 0.0) | 0.0 (0.0 to 0.0) | 0.00 (0.00 to 0.00) |
| Lithuania |  | 0.0 (0.0 to 0.0) | 0.0 (0.0 to 0.0) | 0.00 (0.00 to 0.00) |
| Luxembourg |  | 0.0 (0.0 to 0.0) | 0.0 (0.0 to 0.0) | 0.00 (0.00 to 0.00) |
| Madagascar |  | 16274.6 (12227.2 to 20541.9) | 7802.4 (4338.0 to 11387.2) | -3.47 (-4.13 to -2.80) |
| Malaysia |  | 689.6 (628.6 to 753.3) | 0.0 (0.0 to 0.0) | -32.00 (-37.27 to -26.29) |
| Maldives |  | 0.0 (0.0 to 0.0) | 0.0 (0.0 to 0.0) | 0.00 (0.00 to 0.00) |
| Mali |  | 32683.7 (26456.6 to 42046.3) | 19654.8 (13531.4 to 28757.0) | -1.65 (-1.97 to -1.33) |
| Malta |  | 0.0 (0.0 to 0.0) | 0.0 (0.0 to 0.0) | 0.00 (0.00 to 0.00) |
| Mauritania |  | 2465.8 (1792.5 to 3367.2) | 2616.4 (2160.3 to 3155.9) | -0.42 (-2.68 to 1.90) |
| Micronesia |  | 0.0 (0.0 to 0.0) | 0.0 (0.0 to 0.0) | 0.00 (0.00 to 0.00) |
| Mongolia |  | 0.0 (0.0 to 0.0) | 0.0 (0.0 to 0.0) | 0.00 (0.00 to 0.00) |
| Montenegro |  | 0.0 (0.0 to 0.0) | 0.0 (0.0 to 0.0) | 0.00 (0.00 to 0.00) |
| Morocco |  | 14.3 (10.0 to 20.2) | 0.0 (0.0 to 0.0) | -27.43 (-32.07 to -22.48) |
| Mozambique |  | 32923.1 (27098.3 to 39800.1) | 23058.5 (16693.0 to 29781.0) | -1.50 (-1.70 to -1.31) |
| Myanmar |  | 2287.8 (1875.6 to 2772.6) | 356.1 (300.8 to 420.6) | -3.50 (-5.61 to -1.33) |
| Namibia |  | 8254.0 (977.0 to 31899.4) | 1024.5 (841.9 to 1234.9) | -8.70 (-12.94 to -4.26) |
| Nepal |  | 778.4 (632.1 to 952.8) | 8.4 (7.2 to 9.7) | -15.21 (-17.31 to -13.07) |
| New Zealand |  | 0.0 (0.0 to 0.0) | 0.0 (0.0 to 0.0) | 0.00 (0.00 to 0.00) |
| Nicaragua |  | 2554.2 (2090.0 to 3012.4) | 2340.8 (44.7 to 6919.1) | -9.61 (-14.46 to -4.48) |
| Niger |  | 26095.2 (14899.1 to 37226.0) | 21332.5 (13006.5 to 30283.7) | -0.35 (-0.73 to 0.04) |
| Nigeria |  | 31206.9 (25034.8 to 39079.4) | 22958.3 (16489.5 to 29577.0) | -1.35 (-1.59 to -1.12) |
| Niue |  | 0.0 (0.0 to 0.0) | 0.0 (0.0 to 0.0) | 0.00 (0.00 to 0.00) |
| North Macedonia |  | 0.0 (0.0 to 0.0) | 0.0 (0.0 to 0.0) | 0.00 (0.00 to 0.00) |
| Oman |  | 262.6 (52.9 to 886.7) | 2.4 (0.0 to 18.1) | -18.26 (-28.27 to -6.85) |
| Pakistan |  | 4642.1 (818.8 to 14315.1) | 1682.6 (1333.6 to 2061.6) | -2.64 (-3.41 to -1.86) |
| Panama |  | 47.6 (40.0 to 56.2) | 123.4 (22.2 to 371.3) | -0.74 (-3.61 to 2.21) |
| Papua New Guinea |  | 42491.0 (21927.4 to 84707.3) | 19170.8 (16755.7 to 21790.0) | -3.24 (-3.82 to -2.65) |
| Peru |  | 3462.5 (1109.3 to 9817.8) | 189.9 (39.4 to 374.8) | -8.88 (-10.19 to -7.55) |
| Philippines |  | 429.8 (349.7 to 512.0) | 12.2 (10.3 to 14.4) | -11.30 (-12.25 to -10.35) |
| Poland |  | 0.0 (0.0 to 0.0) | 0.0 (0.0 to 0.0) | 0.00 (0.00 to 0.00) |
| Portugal |  | 0.0 (0.0 to 0.0) | 0.0 (0.0 to 0.0) | 0.00 (0.00 to 0.00) |
| Qatar |  | 0.0 (0.0 to 0.0) | 0.0 (0.0 to 0.0) | 0.00 (0.00 to 0.00) |
| Republic of Korea |  | 3.8 (1.6 to 7.2) | 0.8 (0.2 to 2.0) | -7.02 (-9.41 to -4.58) |
| Republic of Moldova |  | 0.0 (0.0 to 0.0) | 0.0 (0.0 to 0.0) | 0.00 (0.00 to 0.00) |
| Romania |  | 0.0 (0.0 to 0.0) | 0.0 (0.0 to 0.0) | 0.00 (0.00 to 0.00) |
| Russian Federation |  | 0.0 (0.0 to 0.0) | 0.0 (0.0 to 0.0) | 0.00 (0.00 to 0.00) |
| Rwanda |  | 14916.9 (7557.9 to 24178.1) | 3708.8 (2681.0 to 5102.3) | -5.28 (-6.36 to -4.19) |
| Samoa |  | 0.0 (0.0 to 0.0) | 0.0 (0.0 to 0.0) | 0.00 (0.00 to 0.00) |
| Sao Tome and Principe |  | 15374.8 (1764.7 to 58935.5) | 2208.0 (1900.1 to 2554.7) | -8.11 (-10.66 to -5.48) |
| Saudi Arabia |  | 102.1 (102.1 to 102.1) | 0.5 (0.0 to 2.3) | -21.65 (-25.37 to -17.74) |
| Senegal |  | 8507.2 (3990.0 to 15875.5) | 5970.3 (5152.1 to 6792.3) | -2.77 (-3.80 to -1.73) |
| Serbia |  | 0.0 (0.0 to 0.0) | 0.0 (0.0 to 0.0) | 0.00 (0.00 to 0.00) |
| Seychelles |  | 0.0 (0.0 to 0.0) | 0.0 (0.0 to 0.0) | 0.00 (0.00 to 0.00) |
| Sierra Leone |  | 32684.7 (17792.4 to 42073.3) | 25205.7 (14430.1 to 36137.6) | -0.64 (-0.85 to -0.43) |
| Singapore |  | 0.0 (0.0 to 0.0) | 0.0 (0.0 to 0.0) | 0.00 (0.00 to 0.00) |
| Slovakia |  | 0.0 (0.0 to 0.0) | 0.0 (0.0 to 0.0) | 0.00 (0.00 to 0.00) |
| Slovenia |  | 0.0 (0.0 to 0.0) | 0.0 (0.0 to 0.0) | 0.00 (0.00 to 0.00) |
| Solomon Islands |  | 41779.5 (12103.4 to 103434.7) | 26543.4 (23807.8 to 29509.5) | -4.03 (-5.25 to -2.79) |
| Somalia |  | 11521.9 (7269.1 to 18672.2) | 5185.4 (3097.4 to 7087.9) | -4.61 (-5.71 to -3.50) |
| South Africa |  | 217.9 (18.0 to 876.5) | 44.3 (34.2 to 56.0) | -5.84 (-8.13 to -3.50) |
| South Sudan |  | 26350.7 (19435.9 to 33838.0) | 19786.0 (11968.4 to 28729.6) | -1.18 (-1.40 to -0.97) |
| Sri Lanka |  | 4875.9 (4378.6 to 5412.7) | 0.0 (0.0 to 0.0) | -47.26 (-51.06 to -43.16) |
| Sudan |  | 9066.7 (6666.6 to 11987.0) | 7016.3 (3418.4 to 12471.2) | -2.86 (-4.01 to -1.70) |
| Suriname |  | 5685.5 (449.5 to 27300.7) | 198.0 (15.3 to 482.6) | -16.75 (-20.09 to -13.26) |
| Syrian Arab Republic |  | 2.8 (2.2 to 3.6) | 0.0 (0.0 to 0.0) | -30.31 (-35.30 to -24.93) |
| Tajikistan |  | 757.2 (81.1 to 2882.1) | 0.0 (0.0 to 0.0) | -42.47 (-49.12 to -34.97) |
| Thailand |  | 1518.5 (1357.1 to 1704.1) | 8.5 (7.3 to 9.8) | -14.07 (-15.56 to -12.56) |
| Timor-Leste |  | 8658.5 (795.1 to 38601.1) | 0.0 (0.0 to 0.0) | -32.70 (-41.33 to -22.80) |
| Togo |  | 31913.4 (23209.4 to 39774.5) | 19235.8 (10868.6 to 27835.1) | -1.67 (-2.08 to -1.25) |
| Tonga |  | 0.0 (0.0 to 0.0) | 0.0 (0.0 to 0.0) | 0.00 (0.00 to 0.00) |
| Trinidad and Tobago |  | 0.0 (0.0 to 0.0) | 0.0 (0.0 to 0.0) | 0.00 (0.00 to 0.00) |
| Tunisia |  | 0.0 (0.0 to 0.0) | 0.0 (0.0 to 0.0) | 0.00 (0.00 to 0.00) |
| Turkey |  | 19.7 (12.5 to 29.8) | 0.0 (0.0 to 0.0) | -36.39 (-40.79 to -31.67) |
| Uganda |  | 31595.6 (25702.0 to 39581.9) | 17141.6 (12932.3 to 20995.7) | -2.17 (-2.50 to -1.84) |
| Ukraine |  | 0.0 (0.0 to 0.0) | 0.0 (0.0 to 0.0) | 0.00 (0.00 to 0.00) |
| United Arab Emirates |  | 0.0 (0.0 to 0.0) | 0.0 (0.0 to 0.0) | 0.00 (0.00 to 0.00) |
| United Republic of Tanzania |  | 25010.6 (19778.4 to 31741.5) | 10156.3 (6568.5 to 13820.3) | -4.08 (-4.61 to -3.54) |
| Uruguay |  | 0.0 (0.0 to 0.0) | 0.0 (0.0 to 0.0) | 0.00 (0.00 to 0.00) |
| Uzbekistan |  | 0.1 (0.0 to 0.3) | 0.0 (0.0 to 0.0) | -8.78 (-18.92 to 2.64) |
| Vanuatu |  | 36939.3 (33726.5 to 40725.3) | 221.8 (192.2 to 255.0) | -12.07 (-14.70 to -9.36) |
| Venezuela |  | 686.9 (573.8 to 808.9) | 1834.5 (977.5 to 3102.6) | 7.44 (4.71 to 10.24) |
| Viet Nam |  | 232.2 (74.4 to 573.0) | 1.2 (1.0 to 1.5) | -13.06 (-14.95 to -11.14) |
| Yemen |  | 11242.2 (3785.1 to 25628.3) | 6391.8 (5059.8 to 7910.3) | -2.73 (-4.52 to -0.90) |
| Zambia |  | 25501.1 (19754.5 to 32738.8) | 13838.7 (8356.0 to 19341.5) | -2.69 (-3.29 to -2.09) |
| Zimbabwe |  | 6639.4 (5499.9 to 7880.9) | 3819.5 (2899.9 to 4942.4) | 0.35 (-1.10 to 1.83) |

BRI countries: the Belt and Road countries; EAPC: the estimated annual percentage change.

| Supplementary Table 15: NTDs prevalence rate (/100,000 persons) in 149 BRI Countries between 1990 and 2021 | | | | |
| --- | --- | --- | --- | --- |
| Country |  | 1990 | 2021 | EAPC during 1990-2021 (95% CI) (%) |
| Afghanistan |  | 29648.9 | 19412.6 | -1.65 (-2.01 to -1.29) |
| Albania |  | 21.4 | 17.6 | -0.94 (-1.15 to -0.74) |
| Algeria |  | 6304.6 | 6313.7 | -0.86 (-1.28 to -0.45) |
| Angola |  | 76049.5 | 24076.7 | -3.56 (-3.78 to -3.34) |
| Antigua and Barbuda |  | 19713.5 | 12412.3 | -1.79 (-2.45 to -1.12) |
| Argentina |  | 19765.9 | 8506.1 | -1.86 (-2.45 to -1.28) |
| Armenia |  | 6569.7 | 2101.8 | -3.28 (-3.96 to -2.59) |
| Austria |  | 80.1 | 61.8 | -1.05 (-1.12 to -0.98) |
| Azerbaijan |  | 8441.5 | 3015.5 | -2.98 (-3.33 to -2.63) |
| Bahrain |  | 4279.5 | 5353.5 | -0.51 (-1.02 to 0.00) |
| Bangladesh |  | 50923.6 | 23294.9 | -2.84 (-3.20 to -2.49) |
| Barbados |  | 11608.4 | 6482.4 | -2.22 (-3.25 to -1.17) |
| Belarus |  | 138.9 | 89.1 | -1.55 (-1.73 to -1.37) |
| Benin |  | 90248.7 | 24206.6 | -3.18 (-3.73 to -2.62) |
| Bolivia |  | 33066.4 | 21117.0 | -1.30 (-1.66 to -0.93) |
| Bosnia and Herzegovina |  | 110.5 | 98.2 | -0.69 (-0.79 to -0.59) |
| Botswana |  | 52516.5 | 24109.9 | -2.08 (-2.62 to -1.54) |
| Brunei Darussalam |  | 1775.9 | 962.0 | -3.69 (-4.89 to -2.47) |
| Bulgaria |  | 23.5 | 20.8 | -0.61 (-0.96 to -0.25) |
| Burkina Faso |  | 99655.3 | 5678.2 | -9.31 (-10.66 to -7.93) |
| Burundi |  | 52298.1 | 23892.3 | -2.32 (-3.18 to -1.45) |
| Côte d’Ivoire |  | 106959.3 | 24436.7 | -4.34 (-4.77 to -3.90) |
| Cabo Verde |  | 23572.0 | 7321.9 | -3.56 (-3.93 to -3.20) |
| Cambodia |  | 49057.5 | 6343.2 | -7.54 (-8.01 to -7.08) |
| Cameroon |  | 99396.8 | 28377.6 | -4.18 (-4.57 to -3.79) |
| Central African Republic |  | 119284.1 | 49931.3 | -2.79 (-2.95 to -2.63) |
| Chad |  | 62961.5 | 36698.3 | -0.86 (-1.24 to -0.47) |
| Chile |  | 15662.6 | 6769.7 | -1.92 (-2.50 to -1.34) |
| China |  | 38715.1 | 7760.5 | -5.35 (-6.08 to -4.61) |
| Comoros |  | 38543.6 | 12929.6 | -3.71 (-3.96 to -3.45) |
| Congo |  | 64381.4 | 20935.6 | -2.72 (-3.10 to -2.34) |
| Cook Islands |  | 9051.9 | 4177.1 | -2.30 (-2.56 to -2.04) |
| Costa Rica |  | 24510.0 | 15235.8 | -1.49 (-1.60 to -1.38) |
| Croatia |  | 202.7 | 130.0 | -1.46 (-1.65 to -1.26) |
| Cuba |  | 52555.0 | 20633.3 | -3.76 (-4.28 to -3.24) |
| Cyprus |  | 1.5 | 1.5 | 0.22 (0.08 to 0.36) |
| Czechia |  | 2.4 | 2.3 | -0.07 (-0.19 to 0.04) |
| Democratic Republic of the Congo |  | 110361.6 | 50972.9 | -2.04 (-2.50 to -1.58) |
| Djibouti |  | 31124.5 | 9700.7 | -3.79 (-3.96 to -3.62) |
| Dominica |  | 12677.2 | 5089.6 | -3.37 (-4.62 to -2.10) |
| Dominican Republic |  | 41784.7 | 19281.7 | -3.46 (-4.14 to -2.76) |
| Ecuador |  | 34551.5 | 28792.1 | -0.67 (-1.05 to -0.30) |
| Egypt |  | 47423.6 | 10523.2 | -5.62 (-6.35 to -4.88) |
| El Salvador |  | 39559.5 | 16258.1 | -2.98 (-3.10 to -2.86) |
| Equatorial Guinea |  | 79193.9 | 13517.9 | -6.51 (-6.87 to -6.15) |
| Eritrea |  | 39332.4 | 13335.3 | -2.25 (-2.80 to -1.69) |
| Estonia |  | 121.6 | 115.2 | -0.03 (-0.30 to 0.25) |
| Ethiopia |  | 85128.8 | 35941.8 | -3.24 (-3.46 to -3.02) |
| Fiji |  | 44709.8 | 18316.9 | -3.47 (-4.17 to -2.77) |
| Gabon |  | 59983.8 | 15286.9 | -3.21 (-3.65 to -2.78) |
| Gambia |  | 38117.5 | 15894.1 | -1.99 (-2.28 to -1.69) |
| Georgia |  | 5312.5 | 2674.3 | -1.11 (-1.90 to -0.31) |
| Ghana |  | 81862.4 | 30825.0 | -2.71 (-3.14 to -2.29) |
| Greece |  | 77.1 | 56.6 | -0.87 (-0.97 to -0.77) |
| Grenada |  | 17524.1 | 5323.5 | -4.08 (-5.23 to -2.92) |
| Guinea |  | 79321.4 | 29842.9 | -2.44 (-2.83 to -2.05) |
| Guinea-Bissau |  | 75524.0 | 25095.5 | -2.78 (-3.20 to -2.37) |
| Guyana |  | 63050.1 | 24397.7 | -3.46 (-3.84 to -3.07) |
| Hungary |  | 137.0 | 91.0 | -1.57 (-1.65 to -1.50) |
| Indonesia |  | 58000.8 | 7952.4 | -6.90 (-7.29 to -6.52) |
| Iran |  | 5988.9 | 5282.2 | -1.09 (-1.46 to -0.73) |
| Iraq |  | 12933.2 | 12295.4 | -0.89 (-1.20 to -0.57) |
| Italy |  | 14.9 | 9.6 | -1.36 (-1.53 to -1.19) |
| Jamaica |  | 12646.4 | 5808.5 | -3.02 (-4.16 to -1.86) |
| Kazakhstan |  | 7112.2 | 2307.2 | -3.47 (-3.76 to -3.17) |
| Kenya |  | 63973.4 | 32473.3 | -2.68 (-2.99 to -2.38) |
| Kiribati |  | 66896.3 | 14078.6 | -5.40 (-6.03 to -4.77) |
| Kuwait |  | 83.7 | 47.6 | -1.70 (-2.07 to -1.33) |
| Kyrgyzstan |  | 6866.0 | 3373.4 | -1.95 (-2.26 to -1.65) |
| Lao People’s Democratic Republic |  | 65672.2 | 22563.1 | -3.04 (-3.57 to -2.51) |
| Latvia |  | 115.1 | 103.5 | -0.56 (-0.75 to -0.38) |
| Lebanon |  | 4407.0 | 5143.4 | -0.57 (-1.03 to -0.12) |
| Lesotho |  | 49513.2 | 12144.9 | -4.49 (-5.57 to -3.40) |
| Liberia |  | 134666.2 | 64730.1 | -2.09 (-2.33 to -1.84) |
| Libya |  | 13874.1 | 13582.0 | -0.50 (-0.64 to -0.36) |
| Lithuania |  | 149.0 | 119.4 | -0.75 (-0.83 to -0.66) |
| Luxembourg |  | 2.6 | 2.1 | -1.30 (-2.49 to -0.09) |
| Madagascar |  | 58505.1 | 19227.0 | -4.40 (-4.99 to -3.81) |
| Malaysia |  | 42596.0 | 6072.0 | -7.56 (-8.22 to -6.90) |
| Maldives |  | 46303.0 | 3208.1 | -10.02 (-11.09 to -8.93) |
| Mali |  | 97346.3 | 27167.0 | -4.29 (-4.90 to -3.67) |
| Malta |  | 2.1 | 2.5 | 0.94 (0.49 to 1.38) |
| Mauritania |  | 20411.8 | 13284.4 | -0.15 (-0.75 to 0.45) |
| Micronesia |  | 50994.9 | 15007.1 | -3.74 (-4.10 to -3.38) |
| Mongolia |  | 17943.0 | 5331.3 | -3.93 (-4.36 to -3.49) |
| Montenegro |  | 84.8 | 67.1 | -0.84 (-0.91 to -0.78) |
| Morocco |  | 11025.5 | 9351.2 | -1.28 (-1.68 to -0.88) |
| Mozambique |  | 114133.5 | 22645.3 | -5.47 (-6.10 to -4.84) |
| Myanmar |  | 76916.2 | 16020.3 | -6.21 (-6.96 to -5.45) |
| Namibia |  | 55181.7 | 29582.3 | -1.64 (-1.80 to -1.47) |
| Nepal |  | 81346.2 | 17395.6 | -5.19 (-5.84 to -4.54) |
| New Zealand |  | 3.0 | 3.1 | 0.03 (-0.05 to 0.11) |
| Nicaragua |  | 54420.2 | 34108.9 | -1.50 (-1.68 to -1.31) |
| Niger |  | 63985.0 | 26222.6 | -2.34 (-2.60 to -2.07) |
| Nigeria |  | 87771.2 | 33732.6 | -3.65 (-4.01 to -3.28) |
| Niue |  | 18324.8 | 7039.2 | -3.13 (-3.62 to -2.64) |
| North Macedonia |  | 101.2 | 71.3 | -1.45 (-1.54 to -1.36) |
| Oman |  | 9597.9 | 6067.5 | -1.86 (-1.98 to -1.74) |
| Pakistan |  | 54365.2 | 11586.4 | -5.46 (-5.81 to -5.11) |
| Panama |  | 25465.2 | 15926.8 | -1.51 (-1.62 to -1.40) |
| Papua New Guinea |  | 94423.6 | 29802.2 | -4.05 (-4.20 to -3.91) |
| Peru |  | 25907.7 | 42460.6 | 2.09 (1.41 to 2.78) |
| Philippines |  | 49532.4 | 8004.2 | -6.40 (-6.91 to -5.89) |
| Poland |  | 130.2 | 119.0 | -0.28 (-0.41 to -0.15) |
| Portugal |  | 1348.0 | 746.3 | -2.69 (-3.20 to -2.18) |
| Qatar |  | 3256.1 | 4699.2 | -0.06 (-0.61 to 0.50) |
| Republic of Korea |  | 15219.7 | 8772.5 | -0.82 (-1.46 to -0.18) |
| Republic of Moldova |  | 233.5 | 173.6 | -1.15 (-1.24 to -1.07) |
| Romania |  | 146.1 | 115.5 | -1.01 (-1.12 to -0.90) |
| Russian Federation |  | 888.4 | 640.4 | -1.17 (-1.31 to -1.03) |
| Rwanda |  | 64424.6 | 30953.2 | -1.78 (-2.30 to -1.25) |
| Samoa |  | 33725.9 | 14520.4 | -2.19 (-2.82 to -1.55) |
| Sao Tome and Principe |  | 43921.4 | 19128.8 | -2.91 (-3.56 to -2.25) |
| Saudi Arabia |  | 8118.7 | 6176.1 | -1.69 (-2.05 to -1.34) |
| Senegal |  | 84494.6 | 35453.7 | -2.18 (-2.60 to -1.76) |
| Serbia |  | 149.8 | 119.9 | -0.77 (-0.81 to -0.73) |
| Seychelles |  | 37560.4 | 5994.8 | -6.33 (-6.79 to -5.87) |
| Sierra Leone |  | 109841.9 | 25604.5 | -4.83 (-5.49 to -4.17) |
| Singapore |  | 434.5 | 522.4 | 1.34 (0.19 to 2.50) |
| Slovakia |  | 147.5 | 121.1 | -0.58 (-0.66 to -0.51) |
| Slovenia |  | 13.7 | 11.0 | -0.79 (-0.81 to -0.77) |
| Solomon Islands |  | 48729.7 | 21953.5 | -2.54 (-2.60 to -2.48) |
| Somalia |  | 75784.0 | 54141.3 | -1.05 (-1.11 to -0.99) |
| South Africa |  | 53294.8 | 16855.7 | -4.45 (-4.78 to -4.12) |
| South Sudan |  | 69827.4 | 44880.8 | -1.25 (-1.41 to -1.10) |
| Sri Lanka |  | 42352.1 | 6268.2 | -7.11 (-7.47 to -6.74) |
| Sudan |  | 24747.7 | 11200.6 | -2.72 (-3.09 to -2.35) |
| Suriname |  | 29388.5 | 17510.4 | -2.02 (-2.73 to -1.31) |
| Syrian Arab Republic |  | 9866.2 | 11737.8 | -0.03 (-0.27 to 0.21) |
| Tajikistan |  | 12653.2 | 6618.5 | -1.70 (-1.85 to -1.55) |
| Thailand |  | 55358.2 | 6233.2 | -7.52 (-7.85 to -7.18) |
| Timor-Leste |  | 95929.5 | 18373.0 | -7.34 (-8.30 to -6.38) |
| Togo |  | 81963.4 | 20922.8 | -3.72 (-4.49 to -2.95) |
| Tonga |  | 21657.6 | 8531.3 | -2.83 (-3.03 to -2.64) |
| Trinidad and Tobago |  | 12593.1 | 5796.5 | -3.17 (-4.35 to -1.96) |
| Tunisia |  | 6131.0 | 7095.5 | -0.24 (-0.60 to 0.11) |
| Turkey |  | 7049.0 | 5444.4 | -1.92 (-2.33 to -1.50) |
| Uganda |  | 106368.0 | 22324.4 | -4.31 (-5.03 to -3.57) |
| Ukraine |  | 800.0 | 1439.1 | 3.13 (2.40 to 3.86) |
| United Arab Emirates |  | 4362.9 | 4831.7 | -0.66 (-1.24 to -0.08) |
| United Republic of Tanzania |  | 107313.8 | 26217.3 | -4.44 (-5.11 to -3.77) |
| Uruguay |  | 1349.0 | 566.1 | -2.79 (-2.83 to -2.76) |
| Uzbekistan |  | 8888.1 | 3892.0 | -2.27 (-2.78 to -1.75) |
| Vanuatu |  | 75565.7 | 28834.4 | -3.62 (-4.05 to -3.19) |
| Venezuela |  | 44098.6 | 24123.8 | -2.49 (-3.15 to -1.82) |
| Viet Nam |  | 73111.9 | 13889.6 | -6.09 (-6.38 to -5.80) |
| Yemen |  | 32398.8 | 13021.5 | -3.92 (-4.40 to -3.45) |
| Zambia |  | 77198.0 | 23070.9 | -3.84 (-4.39 to -3.29) |
| Zimbabwe |  | 57531.6 | 19972.9 | -2.39 (-2.88 to -1.89) |

BRI countries: the Belt and Road countries; NTDs: the neglected tropical diseases; EAPC: the estimated annual percentage change.

| Supplementary Table 16: NCD mortality rate (/100,000 persons) in 149 BRI Countries between 1990 and 2021 | | | | |
| --- | --- | --- | --- | --- |
| Country |  | 1990 | 2021 | EAPC during 1990-2021 (95% CI) (%) |
| Afghanistan |  | 1047.0 | 778.5 | -1.19 (-1.35 to -1.03) |
| Albania |  | 392.0 | 240.0 | -1.33 (-1.57 to -1.09) |
| Algeria |  | 383.1 | 253.6 | -1.53 (-1.60 to -1.47) |
| Angola |  | 672.0 | 489.2 | -1.23 (-1.31 to -1.14) |
| Antigua and Barbuda |  | 456.5 | 316.5 | -1.22 (-1.43 to -1.01) |
| Argentina |  | 497.8 | 288.3 | -1.66 (-1.73 to -1.58) |
| Armenia |  | 594.5 | 365.8 | -1.81 (-1.97 to -1.64) |
| Austria |  | 380.7 | 178.8 | -2.34 (-2.48 to -2.20) |
| Azerbaijan |  | 670.3 | 434.8 | -1.73 (-1.95 to -1.51) |
| Bahrain |  | 656.9 | 286.2 | -3.60 (-3.94 to -3.26) |
| Bangladesh |  | 667.1 | 386.9 | -1.58 (-1.69 to -1.48) |
| Barbados |  | 428.4 | 306.4 | -0.96 (-1.03 to -0.88) |
| Belarus |  | 614.6 | 517.0 | -1.26 (-1.69 to -0.84) |
| Benin |  | 458.2 | 358.5 | -0.79 (-0.92 to -0.66) |
| Bolivia |  | 528.8 | 336.9 | -1.54 (-1.67 to -1.41) |
| Bosnia and Herzegovina |  | 502.4 | 350.3 | -1.32 (-1.46 to -1.17) |
| Botswana |  | 603.4 | 401.7 | -1.38 (-1.67 to -1.10) |
| Brunei Darussalam |  | 616.1 | 342.8 | -1.78 (-1.99 to -1.56) |
| Bulgaria |  | 679.2 | 567.8 | -0.88 (-1.04 to -0.71) |
| Burkina Faso |  | 446.0 | 388.4 | -0.34 (-0.45 to -0.23) |
| Burundi |  | 757.7 | 439.0 | -2.29 (-2.55 to -2.03) |
| Côte d’Ivoire |  | 466.2 | 406.1 | -0.57 (-0.75 to -0.39) |
| Cabo Verde |  | 349.8 | 365.4 | -0.17 (-0.40 to 0.06) |
| Cambodia |  | 638.0 | 462.8 | -1.19 (-1.28 to -1.11) |
| Cameroon |  | 483.8 | 448.2 | -0.25 (-0.52 to 0.03) |
| Central African Republic |  | 859.2 | 707.6 | -0.74 (-0.82 to -0.66) |
| Chad |  | 442.4 | 472.0 | 0.14 (-0.06 to 0.34) |
| Chile |  | 381.8 | 199.9 | -1.90 (-1.97 to -1.83) |
| China |  | 608.8 | 315.7 | -2.24 (-2.33 to -2.15) |
| Comoros |  | 571.5 | 396.2 | -1.49 (-1.71 to -1.26) |
| Congo |  | 835.5 | 581.0 | -1.51 (-1.67 to -1.34) |
| Cook Islands |  | 712.3 | 457.8 | -1.36 (-1.50 to -1.23) |
| Costa Rica |  | 307.7 | 230.9 | -1.30 (-1.53 to -1.08) |
| Croatia |  | 507.7 | 290.5 | -1.91 (-2.04 to -1.79) |
| Cuba |  | 412.3 | 324.3 | -0.78 (-0.93 to -0.63) |
| Cyprus |  | 338.8 | 178.1 | -2.02 (-2.12 to -1.92) |
| Czechia |  | 654.9 | 271.3 | -2.75 (-2.84 to -2.65) |
| Democratic Republic of the Congo |  | 560.7 | 492.0 | -0.46 (-0.51 to -0.41) |
| Djibouti |  | 490.5 | 426.1 | -0.58 (-0.67 to -0.49) |
| Dominica |  | 505.8 | 432.7 | -0.45 (-0.60 to -0.29) |
| Dominican Republic |  | 348.6 | 375.1 | 0.60 (0.45 to 0.75) |
| Ecuador |  | 307.8 | 230.7 | -0.90 (-1.06 to -0.74) |
| Egypt |  | 683.9 | 574.4 | -0.26 (-0.40 to -0.12) |
| El Salvador |  | 312.1 | 282.6 | -0.48 (-0.70 to -0.26) |
| Equatorial Guinea |  | 772.2 | 455.1 | -2.01 (-2.38 to -1.64) |
| Eritrea |  | 776.9 | 541.3 | -1.20 (-1.26 to -1.14) |
| Estonia |  | 641.0 | 287.9 | -3.12 (-3.39 to -2.85) |
| Ethiopia |  | 691.6 | 314.2 | -3.05 (-3.25 to -2.85) |
| Fiji |  | 1017.2 | 874.7 | -0.55 (-0.64 to -0.47) |
| Gabon |  | 631.4 | 502.2 | -0.85 (-0.97 to -0.72) |
| Gambia |  | 472.7 | 489.1 | -0.08 (-0.27 to 0.12) |
| Georgia |  | 644.5 | 453.4 | -1.33 (-1.57 to -1.08) |
| Ghana |  | 544.2 | 450.4 | -0.53 (-0.66 to -0.41) |
| Greece |  | 329.1 | 251.2 | -0.91 (-1.00 to -0.83) |
| Grenada |  | 642.1 | 445.0 | -1.16 (-1.42 to -0.89) |
| Guinea |  | 493.4 | 473.2 | 0.06 (-0.06 to 0.18) |
| Guinea-Bissau |  | 842.2 | 681.5 | -0.59 (-0.65 to -0.54) |
| Guyana |  | 846.8 | 596.1 | -0.85 (-0.94 to -0.76) |
| Hungary |  | 718.0 | 437.5 | -1.77 (-1.85 to -1.68) |
| Indonesia |  | 527.9 | 506.0 | -0.07 (-0.17 to 0.03) |
| Iran |  | 446.4 | 256.5 | -1.73 (-1.83 to -1.63) |
| Iraq |  | 644.5 | 486.7 | -1.29 (-1.41 to -1.18) |
| Italy |  | 354.0 | 166.7 | -2.46 (-2.54 to -2.38) |
| Jamaica |  | 397.3 | 350.0 | -0.43 (-0.76 to -0.11) |
| Kazakhstan |  | 736.8 | 473.4 | -2.10 (-2.60 to -1.59) |
| Kenya |  | 286.3 | 329.1 | 0.76 (0.50 to 1.01) |
| Kiribati |  | 976.9 | 920.9 | -0.22 (-0.28 to -0.17) |
| Kuwait |  | 368.1 | 183.6 | -2.07 (-2.54 to -1.61) |
| Kyrgyzstan |  | 690.3 | 432.0 | -1.91 (-2.27 to -1.55) |
| Lao People’s Democratic Republic |  | 949.5 | 548.7 | -1.93 (-2.01 to -1.86) |
| Latvia |  | 650.3 | 433.8 | -1.87 (-2.18 to -1.55) |
| Lebanon |  | 549.1 | 231.7 | -2.62 (-2.89 to -2.35) |
| Lesotho |  | 459.4 | 797.2 | 2.63 (2.06 to 3.19) |
| Liberia |  | 478.7 | 446.2 | -0.31 (-0.45 to -0.17) |
| Libya |  | 392.8 | 433.3 | 0.55 (0.37 to 0.72) |
| Lithuania |  | 576.3 | 387.9 | -1.40 (-1.69 to -1.10) |
| Luxembourg |  | 412.0 | 169.5 | -2.82 (-2.90 to -2.73) |
| Madagascar |  | 681.6 | 566.2 | -0.69 (-0.75 to -0.63) |
| Malaysia |  | 535.3 | 398.3 | -0.98 (-1.18 to -0.77) |
| Maldives |  | 638.7 | 173.8 | -4.73 (-4.97 to -4.49) |
| Mali |  | 523.1 | 424.8 | -0.58 (-0.69 to -0.48) |
| Malta |  | 380.1 | 184.5 | -2.26 (-2.35 to -2.16) |
| Mauritania |  | 591.1 | 385.5 | -1.56 (-1.74 to -1.39) |
| Micronesia |  | 1053.4 | 911.8 | -0.46 (-0.49 to -0.43) |
| Mongolia |  | 875.9 | 583.9 | -1.58 (-1.83 to -1.32) |
| Montenegro |  | 491.6 | 426.7 | -0.41 (-0.65 to -0.17) |
| Morocco |  | 530.5 | 440.8 | -0.60 (-0.65 to -0.54) |
| Mozambique |  | 489.8 | 566.1 | 0.98 (0.77 to 1.20) |
| Myanmar |  | 916.3 | 533.2 | -2.02 (-2.16 to -1.89) |
| Namibia |  | 562.7 | 503.5 | -0.56 (-0.89 to -0.23) |
| Nepal |  | 620.2 | 450.6 | -0.89 (-1.15 to -0.62) |
| New Zealand |  | 440.3 | 199.2 | -2.76 (-2.95 to -2.57) |
| Nicaragua |  | 255.9 | 213.7 | -0.50 (-0.65 to -0.34) |
| Niger |  | 426.8 | 345.4 | -0.62 (-0.75 to -0.50) |
| Nigeria |  | 412.1 | 305.6 | -1.15 (-1.27 to -1.02) |
| Niue |  | 730.5 | 663.7 | -0.51 (-0.58 to -0.44) |
| North Macedonia |  | 601.9 | 419.2 | -1.35 (-1.57 to -1.14) |
| Oman |  | 611.3 | 296.3 | -2.07 (-2.33 to -1.81) |
| Pakistan |  | 515.7 | 543.7 | -0.12 (-0.35 to 0.12) |
| Panama |  | 279.2 | 216.7 | -0.87 (-1.03 to -0.71) |
| Papua New Guinea |  | 819.1 | 686.4 | -0.57 (-0.61 to -0.54) |
| Peru |  | 294.7 | 204.2 | -1.49 (-1.82 to -1.16) |
| Philippines |  | 450.1 | 485.5 | 0.27 (0.16 to 0.38) |
| Poland |  | 658.2 | 337.8 | -2.25 (-2.32 to -2.18) |
| Portugal |  | 383.7 | 202.6 | -2.16 (-2.24 to -2.08) |
| Qatar |  | 535.6 | 214.0 | -3.28 (-3.83 to -2.72) |
| Republic of Korea |  | 481.8 | 140.7 | -4.21 (-4.31 to -4.10) |
| Republic of Moldova |  | 628.9 | 446.5 | -1.30 (-1.56 to -1.04) |
| Romania |  | 578.3 | 447.4 | -1.35 (-1.55 to -1.15) |
| Russian Federation |  | 669.4 | 494.9 | -1.64 (-2.18 to -1.10) |
| Rwanda |  | 866.3 | 392.7 | -3.66 (-4.10 to -3.21) |
| Samoa |  | 704.4 | 668.7 | -0.15 (-0.25 to -0.06) |
| Sao Tome and Principe |  | 418.7 | 416.8 | -0.17 (-0.39 to 0.04) |
| Saudi Arabia |  | 513.6 | 428.3 | -0.51 (-0.69 to -0.32) |
| Senegal |  | 500.8 | 423.1 | -0.50 (-0.62 to -0.38) |
| Serbia |  | 585.4 | 417.8 | -1.47 (-1.71 to -1.23) |
| Seychelles |  | 585.0 | 359.2 | -1.31 (-1.44 to -1.17) |
| Sierra Leone |  | 478.6 | 439.4 | 0.01 (-0.16 to 0.18) |
| Singapore |  | 445.1 | 129.2 | -4.00 (-4.09 to -3.92) |
| Slovakia |  | 660.7 | 345.3 | -2.09 (-2.17 to -2.01) |
| Slovenia |  | 439.9 | 194.2 | -2.88 (-3.02 to -2.73) |
| Solomon Islands |  | 923.9 | 870.4 | -0.15 (-0.31 to 0.02) |
| Somalia |  | 712.2 | 556.9 | -0.85 (-0.90 to -0.80) |
| South Africa |  | 434.5 | 471.8 | 0.30 (-0.10 to 0.70) |
| South Sudan |  | 552.9 | 492.3 | -0.58 (-0.87 to -0.29) |
| Sri Lanka |  | 424.0 | 277.6 | -1.38 (-1.61 to -1.14) |
| Sudan |  | 727.4 | 478.3 | -1.48 (-1.53 to -1.43) |
| Suriname |  | 512.1 | 405.4 | -0.80 (-1.04 to -0.56) |
| Syrian Arab Republic |  | 616.6 | 460.9 | -1.35 (-1.57 to -1.12) |
| Tajikistan |  | 632.2 | 389.1 | -1.87 (-2.13 to -1.60) |
| Thailand |  | 421.9 | 288.4 | -1.73 (-1.94 to -1.52) |
| Timor-Leste |  | 495.6 | 453.4 | -0.14 (-0.37 to 0.10) |
| Togo |  | 484.0 | 466.5 | -0.15 (-0.28 to -0.01) |
| Tonga |  | 592.1 | 548.3 | -0.24 (-0.34 to -0.15) |
| Trinidad and Tobago |  | 658.2 | 414.6 | -1.97 (-2.21 to -1.73) |
| Tunisia |  | 370.2 | 286.9 | -1.09 (-1.20 to -0.99) |
| Turkey |  | 582.6 | 311.5 | -2.26 (-2.52 to -2.01) |
| Uganda |  | 500.1 | 422.7 | -1.25 (-1.56 to -0.93) |
| Ukraine |  | 633.9 | 512.5 | -1.29 (-1.68 to -0.90) |
| United Arab Emirates |  | 681.9 | 212.1 | -3.42 (-4.06 to -2.77) |
| United Republic of Tanzania |  | 467.9 | 387.4 | -0.82 (-0.90 to -0.74) |
| Uruguay |  | 513.2 | 340.5 | -1.46 (-1.53 to -1.39) |
| Uzbekistan |  | 591.0 | 453.1 | -1.08 (-1.33 to -0.83) |
| Vanuatu |  | 936.2 | 853.5 | -0.44 (-0.54 to -0.33) |
| Venezuela |  | 445.4 | 370.9 | -1.06 (-1.29 to -0.84) |
| Viet Nam |  | 470.4 | 398.0 | -0.30 (-0.41 to -0.19) |
| Yemen |  | 771.1 | 545.1 | -1.34 (-1.45 to -1.24) |
| Zambia |  | 571.0 | 549.3 | -0.49 (-0.66 to -0.31) |
| Zimbabwe |  | 429.8 | 669.9 | 1.99 (1.34 to 2.65) |

BRI countries: the Belt and Road countries; NCD: Non-communicable diseases; EAPC: the estimated annual percentage change.

| Supplementary Table 17: Suicide mortality rate (/100,000 persons) in 149 BRI Countries between 1990 and 2021 | | | | |
| --- | --- | --- | --- | --- |
| Country |  | 1990 | 2021 | EAPC during 1990-2021 (95% CI) (%) |
| Afghanistan |  | 6.7 (4.9 to 9.2) | 5.3 (4.0 to 7.4) | -0.96 (-1.13 to -0.79) |
| Albania |  | 4.9 (4.3 to 5.7) | 3.2 (2.6 to 4.1) | -1.25 (-1.56 to -0.95) |
| Algeria |  | 4.5 (3.0 to 5.6) | 2.9 (2.0 to 3.6) | -1.76 (-1.88 to -1.63) |
| Angola |  | 18.4 (13.9 to 23.2) | 13.8 (10.3 to 17.3) | -1.08 (-1.16 to -1.00) |
| Antigua and Barbuda |  | 2.0 (1.8 to 2.2) | 1.2 (1.1 to 1.3) | -1.38 (-1.55 to -1.21) |
| Argentina |  | 8.7 (8.4 to 9.1) | 9.6 (9.0 to 10.2) | 0.24 (-0.08 to 0.55) |
| Armenia |  | 4.1 (4.0 to 4.3) | 4.5 (4.1 to 5.1) | 0.66 (-0.03 to 1.36) |
| Austria |  | 19.4 (18.9 to 20.0) | 10.0 (9.4 to 10.5) | -1.98 (-2.14 to -1.83) |
| Azerbaijan |  | 3.2 (2.8 to 3.6) | 2.3 (1.8 to 3.1) | -0.91 (-1.28 to -0.53) |
| Bahrain |  | 7.3 (6.5 to 8.2) | 3.9 (3.3 to 4.8) | -3.04 (-3.76 to -2.32) |
| Bangladesh |  | 8.1 (6.6 to 9.5) | 3.7 (2.8 to 5.1) | -2.76 (-2.93 to -2.59) |
| Barbados |  | 4.8 (4.5 to 5.2) | 3.5 (2.7 to 4.5) | -1.17 (-1.40 to -0.94) |
| Belarus |  | 20.0 (19.2 to 20.8) | 16.1 (13.2 to 19.4) | -1.94 (-2.86 to -1.01) |
| Benin |  | 10.3 (8.6 to 12.4) | 9.8 (7.1 to 14.0) | -0.24 (-0.38 to -0.10) |
| Bolivia |  | 6.8 (5.5 to 8.2) | 5.7 (3.9 to 7.8) | -0.66 (-0.74 to -0.57) |
| Bosnia and Herzegovina |  | 14.0 (12.8 to 15.5) | 7.7 (5.7 to 9.4) | -2.18 (-2.37 to -2.00) |
| Botswana |  | 16.3 (11.6 to 22.6) | 10.5 (7.6 to 14.6) | -1.75 (-2.10 to -1.39) |
| Brunei Darussalam |  | 4.9 (3.9 to 5.8) | 3.7 (2.9 to 4.2) | -0.59 (-0.84 to -0.34) |
| Bulgaria |  | 16.7 (16.0 to 17.6) | 9.3 (8.0 to 10.6) | -2.66 (-2.97 to -2.35) |
| Burkina Faso |  | 14.1 (11.5 to 16.7) | 12.6 (10.2 to 16.3) | -0.29 (-0.41 to -0.18) |
| Burundi |  | 21.7 (15.3 to 31.4) | 13.8 (10.6 to 20.1) | -1.92 (-2.11 to -1.72) |
| Côte d’Ivoire |  | 14.1 (11.7 to 17.2) | 12.5 (9.1 to 17.5) | -0.60 (-0.79 to -0.41) |
| Cabo Verde |  | 14.4 (11.6 to 16.9) | 15.9 (12.3 to 20.1) | 0.06 (-0.08 to 0.21) |
| Cambodia |  | 6.8 (5.4 to 8.1) | 4.9 (3.6 to 6.4) | -1.22 (-1.30 to -1.14) |
| Cameroon |  | 11.4 (9.6 to 13.8) | 12.4 (8.3 to 17.6) | 0.12 (-0.17 to 0.40) |
| Central African Republic |  | 22.3 (17.4 to 27.8) | 19.6 (14.0 to 26.2) | -0.48 (-0.55 to -0.41) |
| Chad |  | 8.2 (5.9 to 12.0) | 11.7 (7.9 to 18.9) | 1.06 (0.72 to 1.41) |
| Chile |  | 21.1 (20.1 to 22.1) | 9.2 (8.8 to 9.7) | -2.36 (-2.73 to -1.99) |
| China |  | 21.5 (14.4 to 24.6) | 7.1 (5.8 to 8.8) | -4.24 (-4.53 to -3.95) |
| Comoros |  | 10.6 (6.8 to 15.5) | 8.3 (5.7 to 12.7) | -1.18 (-1.48 to -0.87) |
| Congo |  | 20.0 (15.9 to 24.5) | 14.2 (10.9 to 18.2) | -1.40 (-1.57 to -1.22) |
| Cook Islands |  | 16.3 (13.0 to 20.3) | 10.8 (8.5 to 13.8) | -1.06 (-1.35 to -0.77) |
| Costa Rica |  | 6.1 (5.7 to 6.4) | 8.9 (8.0 to 9.8) | 0.81 (0.48 to 1.15) |
| Croatia |  | 20.3 (19.4 to 21.2) | 9.3 (8.2 to 10.4) | -2.51 (-2.61 to -2.42) |
| Cuba |  | 19.9 (19.3 to 20.3) | 11.0 (9.5 to 12.4) | -2.34 (-2.73 to -1.96) |
| Cyprus |  | 6.3 (5.6 to 7.2) | 3.2 (2.7 to 3.7) | -2.43 (-2.58 to -2.27) |
| Czechia |  | 18.3 (17.6 to 19.0) | 9.2 (8.1 to 10.2) | -1.84 (-2.03 to -1.65) |
| Democratic Republic of the Congo |  | 15.1 (12.0 to 19.0) | 14.4 (10.3 to 20.6) | -0.13 (-0.22 to -0.04) |
| Djibouti |  | 8.6 (5.6 to 13.7) | 8.6 (5.4 to 14.5) | -0.09 (-0.25 to 0.08) |
| Dominica |  | 3.7 (3.3 to 4.2) | 3.7 (2.9 to 4.5) | -0.06 (-0.22 to 0.10) |
| Dominican Republic |  | 5.6 (4.8 to 6.6) | 5.3 (4.3 to 6.8) | 0.39 (0.16 to 0.63) |
| Ecuador |  | 5.1 (4.9 to 5.3) | 8.4 (6.7 to 10.3) | 1.75 (1.28 to 2.22) |
| Egypt |  | 1.5 (1.1 to 1.7) | 1.1 (0.9 to 1.4) | -0.65 (-0.76 to -0.53) |
| El Salvador |  | 10.2 (9.5 to 11.4) | 8.2 (6.6 to 10.1) | -0.64 (-0.86 to -0.42) |
| Equatorial Guinea |  | 20.4 (15.3 to 25.6) | 12.1 (8.4 to 17.3) | -1.98 (-2.41 to -1.55) |
| Eritrea |  | 18.2 (14.5 to 22.6) | 15.7 (11.6 to 21.1) | -0.51 (-0.56 to -0.46) |
| Estonia |  | 24.3 (23.2 to 25.5) | 11.2 (9.8 to 12.4) | -3.90 (-4.38 to -3.41) |
| Ethiopia |  | 22.6 (15.5 to 27.0) | 11.3 (9.4 to 13.5) | -2.61 (-2.75 to -2.47) |
| Fiji |  | 11.1 (9.4 to 13.0) | 9.2 (6.9 to 11.8) | -0.53 (-0.64 to -0.42) |
| Gabon |  | 16.9 (13.3 to 21.1) | 13.7 (10.0 to 18.4) | -0.78 (-0.87 to -0.68) |
| Gambia |  | 7.9 (5.3 to 12.1) | 9.6 (6.4 to 14.2) | 0.32 (0.11 to 0.53) |
| Georgia |  | 5.1 (4.8 to 5.5) | 7.2 (6.3 to 8.2) | 1.02 (0.46 to 1.58) |
| Ghana |  | 7.1 (5.6 to 8.9) | 8.7 (6.7 to 11.1) | 1.11 (0.90 to 1.32) |
| Greece |  | 3.2 (3.1 to 3.3) | 3.6 (3.4 to 3.9) | 0.56 (0.22 to 0.90) |
| Grenada |  | 6.2 (5.6 to 6.9) | 4.1 (3.5 to 4.8) | -1.35 (-1.50 to -1.19) |
| Guinea |  | 7.0 (5.1 to 9.8) | 8.9 (5.9 to 13.4) | 0.87 (0.65 to 1.09) |
| Guinea-Bissau |  | 14.8 (9.6 to 20.1) | 15.1 (11.2 to 19.5) | 0.13 (0.00 to 0.26) |
| Guyana |  | 28.7 (25.2 to 31.9) | 31.5 (24.2 to 40.3) | 0.53 (0.17 to 0.89) |
| Hungary |  | 33.3 (31.9 to 34.7) | 11.6 (10.4 to 12.8) | -3.31 (-3.48 to -3.14) |
| Indonesia |  | 2.2 (1.8 to 2.5) | 1.7 (1.4 to 2.1) | -0.85 (-0.98 to -0.72) |
| Iran |  | 6.3 (5.3 to 6.9) | 4.1 (3.7 to 4.6) | -1.21 (-1.28 to -1.14) |
| Iraq |  | 7.4 (5.9 to 8.9) | 4.9 (3.7 to 6.5) | -1.64 (-1.78 to -1.51) |
| Italy |  | 6.5 (6.4 to 6.7) | 4.3 (4.1 to 4.5) | -1.45 (-1.67 to -1.24) |
| Jamaica |  | 1.2 (1.0 to 1.4) | 1.3 (1.0 to 1.7) | 0.22 (-0.25 to 0.70) |
| Kazakhstan |  | 21.3 (19.9 to 22.8) | 18.0 (15.9 to 20.1) | -1.57 (-2.39 to -0.74) |
| Kenya |  | 11.4 (8.9 to 17.7) | 11.7 (9.1 to 15.8) | 0.11 (-0.13 to 0.36) |
| Kiribati |  | 18.2 (14.4 to 22.1) | 17.6 (12.8 to 22.6) | -0.15 (-0.20 to -0.11) |
| Kuwait |  | 1.6 (1.5 to 1.7) | 1.8 (1.5 to 2.1) | -1.08 (-2.56 to 0.41) |
| Kyrgyzstan |  | 15.7 (14.5 to 16.9) | 10.0 (8.5 to 11.6) | -1.90 (-2.15 to -1.65) |
| Lao People’s Democratic Republic |  | 10.6 (6.5 to 14.0) | 5.5 (4.1 to 7.4) | -2.32 (-2.41 to -2.23) |
| Latvia |  | 25.1 (23.9 to 26.3) | 14.3 (12.7 to 15.9) | -3.07 (-3.51 to -2.62) |
| Lebanon |  | 1.7 (0.9 to 2.2) | 0.9 (0.7 to 1.0) | -2.04 (-2.17 to -1.91) |
| Lesotho |  | 13.2 (9.4 to 22.3) | 24.3 (17.2 to 32.9) | 2.68 (2.14 to 3.23) |
| Liberia |  | 9.7 (7.4 to 12.0) | 11.3 (7.6 to 16.0) | 0.45 (0.29 to 0.61) |
| Libya |  | 4.4 (3.0 to 5.6) | 4.9 (3.2 to 6.8) | 0.66 (0.50 to 0.82) |
| Lithuania |  | 27.3 (26.3 to 28.4) | 20.6 (18.3 to 22.7) | -1.85 (-2.46 to -1.23) |
| Luxembourg |  | 16.6 (15.8 to 17.5) | 6.5 (5.9 to 7.2) | -2.99 (-3.12 to -2.87) |
| Madagascar |  | 13.4 (11.2 to 16.5) | 11.0 (7.9 to 14.9) | -0.67 (-0.73 to -0.61) |
| Malaysia |  | 6.8 (6.1 to 7.5) | 5.1 (4.6 to 5.8) | -1.16 (-1.35 to -0.98) |
| Maldives |  | 5.0 (2.8 to 6.2) | 2.0 (1.6 to 2.5) | -3.10 (-3.25 to -2.94) |
| Mali |  | 8.1 (5.5 to 11.6) | 8.1 (5.6 to 11.8) | 0.08 (0.02 to 0.14) |
| Malta |  | 5.1 (4.7 to 5.4) | 3.6 (3.2 to 4.0) | -0.92 (-1.09 to -0.76) |
| Mauritania |  | 6.7 (4.8 to 10.1) | 5.9 (3.9 to 9.8) | -0.61 (-0.69 to -0.52) |
| Micronesia |  | 25.0 (16.4 to 31.5) | 20.1 (13.6 to 26.2) | -0.70 (-0.79 to -0.61) |
| Mongolia |  | 15.6 (12.0 to 20.0) | 15.2 (12.5 to 18.2) | -0.23 (-0.47 to 0.00) |
| Montenegro |  | 15.2 (13.2 to 17.8) | 12.1 (10.1 to 14.5) | -0.70 (-0.96 to -0.45) |
| Morocco |  | 5.2 (3.4 to 6.5) | 3.6 (2.3 to 5.0) | -1.35 (-1.49 to -1.20) |
| Mozambique |  | 14.4 (11.8 to 17.4) | 17.7 (12.8 to 22.9) | 1.20 (1.01 to 1.40) |
| Myanmar |  | 5.8 (3.4 to 7.8) | 3.6 (2.4 to 4.6) | -1.83 (-1.97 to -1.70) |
| Namibia |  | 13.6 (10.9 to 16.4) | 13.7 (9.8 to 18.6) | -0.28 (-0.70 to 0.14) |
| Nepal |  | 18.2 (11.9 to 23.1) | 14.2 (10.7 to 18.5) | -0.74 (-0.84 to -0.63) |
| New Zealand |  | 12.8 (12.4 to 13.3) | 10.2 (9.7 to 10.8) | -1.02 (-1.20 to -0.84) |
| Nicaragua |  | 5.4 (4.9 to 7.1) | 5.8 (4.8 to 7.0) | -0.04 (-0.54 to 0.45) |
| Niger |  | 7.6 (5.5 to 11.2) | 7.9 (5.2 to 12.1) | 0.13 (0.05 to 0.22) |
| Nigeria |  | 10.4 (6.8 to 13.4) | 8.3 (5.9 to 10.6) | -0.82 (-0.97 to -0.67) |
| Niue |  | 16.0 (11.7 to 21.2) | 15.4 (12.3 to 19.3) | -0.83 (-1.06 to -0.61) |
| North Macedonia |  | 9.3 (8.2 to 10.6) | 6.1 (4.8 to 7.4) | -1.51 (-1.82 to -1.21) |
| Oman |  | 1.9 (1.4 to 2.6) | 1.0 (0.8 to 1.3) | -1.53 (-1.79 to -1.27) |
| Pakistan |  | 6.3 (4.4 to 8.4) | 6.4 (4.4 to 9.5) | -0.33 (-0.61 to -0.04) |
| Panama |  | 4.2 (3.9 to 4.5) | 5.1 (4.2 to 6.1) | 0.20 (-0.20 to 0.61) |
| Papua New Guinea |  | 3.0 (2.2 to 4.0) | 2.8 (2.1 to 4.3) | -0.25 (-0.36 to -0.13) |
| Peru |  | 3.2 (2.6 to 3.8) | 2.8 (2.0 to 3.6) | -0.81 (-1.08 to -0.54) |
| Philippines |  | 4.3 (3.8 to 4.7) | 3.8 (3.2 to 4.6) | -0.18 (-0.29 to -0.06) |
| Poland |  | 15.9 (15.6 to 16.2) | 12.1 (11.2 to 13.1) | -1.10 (-1.34 to -0.86) |
| Portugal |  | 14.1 (13.5 to 14.5) | 7.2 (6.8 to 7.7) | -1.77 (-1.98 to -1.56) |
| Qatar |  | 7.7 (6.3 to 9.5) | 3.4 (2.6 to 4.5) | -2.86 (-3.45 to -2.27) |
| Republic of Korea |  | 12.9 (11.0 to 30.4) | 18.1 (11.1 to 19.7) | 1.63 (0.89 to 2.37) |
| Republic of Moldova |  | 16.9 (16.2 to 17.6) | 11.3 (10.1 to 12.7) | -1.50 (-1.81 to -1.19) |
| Romania |  | 9.3 (8.9 to 9.6) | 7.9 (7.0 to 8.9) | -0.90 (-1.25 to -0.54) |
| Russian Federation |  | 27.5 (27.1 to 27.9) | 19.9 (18.4 to 21.3) | -2.18 (-2.83 to -1.53) |
| Rwanda |  | 23.6 (18.0 to 29.2) | 11.9 (8.6 to 16.6) | -3.36 (-3.80 to -2.93) |
| Samoa |  | 16.0 (11.9 to 20.4) | 13.6 (9.9 to 17.5) | -0.47 (-0.71 to -0.22) |
| Sao Tome and Principe |  | 1.5 (1.0 to 1.8) | 1.5 (1.1 to 2.1) | -0.05 (-0.25 to 0.16) |
| Saudi Arabia |  | 9.2 (6.6 to 11.8) | 6.6 (5.0 to 8.6) | -1.26 (-1.44 to -1.08) |
| Senegal |  | 13.3 (10.6 to 16.1) | 12.5 (9.7 to 16.4) | -0.14 (-0.25 to -0.03) |
| Serbia |  | 20.7 (18.3 to 22.9) | 10.9 (9.1 to 12.8) | -2.36 (-2.59 to -2.13) |
| Seychelles |  | 8.6 (7.7 to 9.6) | 5.1 (4.3 to 5.8) | -1.53 (-1.81 to -1.25) |
| Sierra Leone |  | 7.1 (4.9 to 10.1) | 8.5 (5.7 to 12.6) | 0.75 (0.54 to 0.97) |
| Singapore |  | 14.9 (14.3 to 15.5) | 6.6 (6.2 to 7.0) | -2.56 (-2.83 to -2.29) |
| Slovakia |  | 16.4 (14.6 to 18.0) | 9.3 (7.8 to 10.9) | -1.48 (-1.66 to -1.30) |
| Slovenia |  | 26.4 (25.3 to 27.7) | 10.9 (9.6 to 12.2) | -3.14 (-3.37 to -2.91) |
| Solomon Islands |  | 21.9 (10.0 to 30.1) | 19.9 (13.5 to 26.8) | -0.33 (-0.44 to -0.22) |
| Somalia |  | 13.6 (8.4 to 22.2) | 12.9 (8.4 to 21.9) | -0.20 (-0.28 to -0.11) |
| South Africa |  | 19.2 (15.5 to 22.1) | 14.7 (13.2 to 16.7) | -1.05 (-1.47 to -0.63) |
| South Sudan |  | 14.8 (10.3 to 20.0) | 15.1 (11.1 to 20.3) | -0.15 (-0.36 to 0.05) |
| Sri Lanka |  | 40.0 (35.6 to 43.8) | 15.1 (10.3 to 20.6) | -3.98 (-4.42 to -3.54) |
| Sudan |  | 6.6 (3.8 to 8.8) | 4.7 (2.8 to 6.8) | -1.22 (-1.29 to -1.16) |
| Suriname |  | 25.3 (20.9 to 27.8) | 22.2 (17.8 to 26.9) | -0.34 (-0.54 to -0.15) |
| Syrian Arab Republic |  | 1.7 (1.3 to 2.2) | 0.9 (0.7 to 1.2) | -2.80 (-3.15 to -2.45) |
| Tajikistan |  | 6.2 (5.2 to 7.1) | 3.9 (2.9 to 5.4) | -1.92 (-2.14 to -1.70) |
| Thailand |  | 11.1 (9.4 to 13.5) | 10.1 (7.8 to 12.5) | -1.24 (-1.61 to -0.87) |
| Timor-Leste |  | 6.3 (4.5 to 8.2) | 4.7 (3.5 to 6.5) | -0.94 (-1.42 to -0.45) |
| Togo |  | 10.2 (8.1 to 12.8) | 12.5 (8.8 to 17.1) | 0.59 (0.33 to 0.85) |
| Tonga |  | 5.3 (4.4 to 6.4) | 5.2 (3.9 to 6.9) | 0.00 (-0.10 to 0.09) |
| Trinidad and Tobago |  | 16.5 (15.7 to 17.3) | 11.9 (9.1 to 15.1) | -1.27 (-1.52 to -1.03) |
| Tunisia |  | 3.6 (2.5 to 5.2) | 2.9 (1.9 to 4.2) | -1.04 (-1.18 to -0.90) |
| Turkey |  | 4.3 (3.0 to 5.8) | 3.6 (2.7 to 4.3) | -0.41 (-0.83 to 0.01) |
| Uganda |  | 13.1 (10.2 to 16.1) | 11.6 (8.8 to 14.5) | -0.98 (-1.26 to -0.69) |
| Ukraine |  | 19.0 (18.3 to 19.7) | 18.6 (13.5 to 24.4) | -1.35 (-1.91 to -0.78) |
| United Arab Emirates |  | 5.3 (3.9 to 7.2) | 2.8 (2.2 to 3.6) | -1.54 (-1.83 to -1.24) |
| United Republic of Tanzania |  | 13.0 (10.9 to 15.4) | 9.8 (7.5 to 13.1) | -1.24 (-1.36 to -1.11) |
| Uruguay |  | 11.3 (10.8 to 11.8) | 18.2 (17.2 to 19.4) | 1.21 (0.96 to 1.46) |
| Uzbekistan |  | 9.4 (9.0 to 9.8) | 9.1 (7.9 to 10.5) | -0.11 (-0.40 to 0.19) |
| Vanuatu |  | 19.7 (12.5 to 25.4) | 17.6 (12.8 to 22.5) | -0.56 (-0.66 to -0.46) |
| Venezuela |  | 6.3 (6.1 to 6.6) | 8.0 (6.1 to 10.2) | 0.26 (-0.10 to 0.63) |
| Viet Nam |  | 9.7 (7.3 to 12.2) | 7.7 (5.8 to 9.7) | -0.65 (-0.73 to -0.57) |
| Yemen |  | 5.6 (2.8 to 8.0) | 4.5 (2.2 to 6.5) | -1.01 (-1.22 to -0.81) |
| Zambia |  | 16.5 (13.9 to 19.6) | 14.9 (11.2 to 19.4) | -0.75 (-1.02 to -0.49) |
| Zimbabwe |  | 14.6 (9.5 to 19.5) | 23.8 (15.3 to 31.1) | 1.89 (1.33 to 2.46) |

BRI countries: the Belt and Road countries; EAPC: the estimated annual percentage change.

| Supplementary Table 18: Road traffic mortality rate (/100,000 persons) in 149 BRI Countries between 1990 and 2021 | | | | |
| --- | --- | --- | --- | --- |
| Country |  | 1990 | 2021 | EAPC during 1990-2021 (95% CI) (%) |
| Afghanistan |  | 45.8 (35.7 to 57.7) | 31.3 (24.3 to 39.8) | -1.50 (-1.64 to -1.35) |
| Albania |  | 10.7 (10.0 to 11.4) | 5.3 (4.4 to 6.3) | -1.78 (-2.09 to -1.47) |
| Algeria |  | 49.5 (42.8 to 55.9) | 23.7 (19.8 to 27.6) | -2.52 (-2.63 to -2.41) |
| Angola |  | 71.1 (57.2 to 87.1) | 41.4 (31.9 to 52.1) | -1.87 (-1.95 to -1.79) |
| Antigua and Barbuda |  | 9.9 (9.2 to 10.7) | 4.0 (3.7 to 4.3) | -2.88 (-3.13 to -2.62) |
| Argentina |  | 13.2 (12.8 to 13.6) | 10.2 (9.7 to 10.6) | -0.66 (-0.98 to -0.34) |
| Armenia |  | 19.1 (17.9 to 20.5) | 10.0 (8.9 to 11.2) | -1.33 (-2.16 to -0.50) |
| Austria |  | 16.9 (16.5 to 17.3) | 3.5 (3.3 to 3.7) | -5.29 (-5.46 to -5.12) |
| Azerbaijan |  | 15.9 (14.5 to 17.4) | 5.2 (4.3 to 6.2) | -3.57 (-4.14 to -2.99) |
| Bahrain |  | 28.8 (26.0 to 31.7) | 12.8 (10.8 to 15.0) | -3.29 (-3.70 to -2.87) |
| Bangladesh |  | 9.2 (7.7 to 10.7) | 7.0 (5.6 to 8.6) | -0.26 (-0.50 to -0.03) |
| Barbados |  | 13.2 (12.5 to 13.9) | 7.1 (5.6 to 9.1) | -2.33 (-2.62 to -2.04) |
| Belarus |  | 25.8 (24.9 to 26.7) | 7.6 (6.3 to 9.0) | -4.37 (-4.96 to -3.77) |
| Benin |  | 29.8 (24.8 to 35.0) | 21.4 (16.9 to 26.6) | -1.14 (-1.28 to -1.00) |
| Bolivia |  | 34.5 (29.4 to 40.4) | 18.1 (13.9 to 23.6) | -2.14 (-2.28 to -2.01) |
| Bosnia and Herzegovina |  | 7.3 (6.7 to 8.0) | 3.8 (3.1 to 4.6) | -2.10 (-2.30 to -1.89) |
| Botswana |  | 24.9 (19.0 to 32.2) | 17.9 (13.6 to 22.2) | -0.93 (-1.27 to -0.60) |
| Brunei Darussalam |  | 28.1 (24.6 to 31.2) | 9.1 (8.0 to 10.3) | -3.87 (-4.14 to -3.60) |
| Bulgaria |  | 18.0 (17.0 to 19.0) | 8.4 (7.2 to 9.6) | -2.40 (-2.65 to -2.14) |
| Burkina Faso |  | 40.2 (31.7 to 47.8) | 32.4 (25.3 to 39.7) | -0.46 (-0.62 to -0.31) |
| Burundi |  | 42.3 (33.1 to 51.6) | 29.2 (23.1 to 36.4) | -1.45 (-1.56 to -1.35) |
| Côte d'Ivoire |  | 26.0 (21.0 to 31.0) | 21.5 (16.3 to 27.9) | -0.71 (-0.91 to -0.50) |
| Cabo Verde |  | 7.2 (6.1 to 8.4) | 8.4 (6.7 to 10.5) | 0.16 (-0.15 to 0.47) |
| Cambodia |  | 25.0 (20.8 to 29.8) | 18.7 (14.2 to 24.5) | -1.02 (-1.09 to -0.96) |
| Cameroon |  | 40.0 (33.9 to 47.7) | 33.1 (24.4 to 44.2) | -0.65 (-0.86 to -0.43) |
| Central African Republic |  | 83.9 (70.8 to 102.6) | 69.6 (51.7 to 90.8) | -0.65 (-0.72 to -0.58) |
| Chad |  | 23.1 (19.2 to 27.8) | 25.0 (19.6 to 31.2) | 0.25 (0.03 to 0.47) |
| Chile |  | 15.3 (14.7 to 15.8) | 9.5 (9.0 to 10.0) | -1.83 (-1.99 to -1.66) |
| China |  | 20.8 (18.4 to 23.7) | 14.4 (12.0 to 17.0) | -0.90 (-1.39 to -0.40) |
| Comoros |  | 37.4 (27.9 to 45.8) | 28.8 (23.0 to 37.2) | -1.19 (-1.47 to -0.91) |
| Congo |  | 67.4 (55.4 to 81.6) | 41.1 (31.2 to 52.9) | -1.94 (-2.14 to -1.74) |
| Cook Islands |  | 32.7 (27.3 to 39.9) | 17.4 (13.9 to 21.9) | -2.08 (-2.20 to -1.96) |
| Costa Rica |  | 20.9 (20.1 to 21.7) | 17.3 (15.7 to 18.7) | -0.97 (-1.27 to -0.66) |
| Croatia |  | 18.6 (17.9 to 19.3) | 5.9 (5.3 to 6.6) | -3.49 (-3.83 to -3.15) |
| Cuba |  | 21.3 (20.6 to 21.9) | 7.6 (6.6 to 8.7) | -3.96 (-4.46 to -3.44) |
| Cyprus |  | 18.9 (17.4 to 20.4) | 5.8 (5.1 to 6.7) | -4.35 (-4.84 to -3.86) |
| Czechia |  | 13.4 (12.8 to 14.0) | 4.9 (4.4 to 5.5) | -3.82 (-4.13 to -3.50) |
| Democratic Republic of the Congo |  | 58.3 (48.1 to 69.6) | 48.5 (37.9 to 59.6) | -0.49 (-0.57 to -0.42) |
| Djibouti |  | 24.7 (19.5 to 31.3) | 21.9 (16.4 to 29.3) | -0.51 (-0.73 to -0.28) |
| Dominica |  | 16.7 (15.2 to 18.4) | 13.5 (11.0 to 16.3) | -0.77 (-1.05 to -0.49) |
| Dominican Republic |  | 24.9 (22.2 to 27.8) | 21.3 (17.5 to 25.6) | 0.10 (-0.13 to 0.34) |
| Ecuador |  | 29.8 (28.8 to 30.6) | 22.1 (18.1 to 26.9) | -0.29 (-0.73 to 0.15) |
| Egypt |  | 20.7 (18.8 to 23.1) | 11.8 (9.7 to 13.9) | -1.39 (-1.55 to -1.24) |
| El Salvador |  | 31.8 (29.9 to 33.7) | 19.2 (15.5 to 23.1) | -2.19 (-2.51 to -1.87) |
| Equatorial Guinea |  | 67.8 (53.2 to 86.2) | 36.7 (25.1 to 52.3) | -2.43 (-2.86 to -1.99) |
| Eritrea |  | 40.9 (33.5 to 50.2) | 32.1 (24.3 to 41.3) | -0.89 (-0.96 to -0.83) |
| Estonia |  | 26.9 (25.6 to 28.1) | 3.9 (3.4 to 4.3) | -6.45 (-6.83 to -6.06) |
| Ethiopia |  | 40.8 (35.4 to 49.4) | 17.8 (15.2 to 20.6) | -3.14 (-3.35 to -2.94) |
| Fiji |  | 13.2 (11.5 to 15.4) | 9.1 (7.0 to 11.5) | -1.42 (-1.57 to -1.28) |
| Gabon |  | 55.3 (45.8 to 66.8) | 38.7 (27.9 to 51.5) | -1.20 (-1.30 to -1.10) |
| Gambia |  | 23.8 (18.6 to 29.5) | 23.6 (18.0 to 29.1) | -0.25 (-0.45 to -0.05) |
| Georgia |  | 26.5 (24.8 to 28.4) | 14.9 (13.2 to 16.8) | -0.06 (-1.07 to 0.96) |
| Ghana |  | 26.6 (21.1 to 32.1) | 21.6 (17.1 to 27.4) | -0.32 (-0.48 to -0.17) |
| Greece |  | 20.1 (19.6 to 20.6) | 7.6 (7.3 to 7.9) | -3.50 (-3.75 to -3.24) |
| Grenada |  | 14.9 (13.5 to 16.4) | 7.1 (6.2 to 8.1) | -2.58 (-2.76 to -2.40) |
| Guinea |  | 26.0 (20.9 to 30.7) | 22.5 (17.2 to 29.0) | -0.29 (-0.41 to -0.17) |
| Guinea-Bissau |  | 50.6 (40.2 to 63.2) | 33.4 (25.7 to 42.1) | -1.22 (-1.35 to -1.10) |
| Guyana |  | 20.0 (17.4 to 22.5) | 15.7 (12.3 to 20.0) | -0.24 (-0.55 to 0.07) |
| Hungary |  | 21.8 (21.1 to 22.5) | 6.1 (5.4 to 6.8) | -4.20 (-4.59 to -3.81) |
| Indonesia |  | 26.6 (23.8 to 29.5) | 15.8 (13.0 to 19.5) | -1.98 (-2.18 to -1.78) |
| Iran |  | 57.3 (53.2 to 62.3) | 21.5 (20.3 to 23.1) | -3.22 (-3.51 to -2.94) |
| Iraq |  | 24.7 (20.7 to 29.5) | 14.4 (11.1 to 18.3) | -2.02 (-2.20 to -1.85) |
| Italy |  | 16.6 (16.3 to 16.9) | 4.8 (4.7 to 5.0) | -4.53 (-4.83 to -4.23) |
| Jamaica |  | 3.9 (3.8 to 4.2) | 3.5 (2.7 to 4.5) | -0.72 (-1.42 to -0.01) |
| Kazakhstan |  | 27.5 (25.5 to 29.4) | 10.8 (9.6 to 12.2) | -2.23 (-3.02 to -1.44) |
| Kenya |  | 16.7 (13.9 to 19.6) | 17.9 (14.5 to 22.1) | 0.48 (0.21 to 0.76) |
| Kiribati |  | 10.7 (8.8 to 12.8) | 13.0 (10.1 to 16.0) | 0.98 (0.64 to 1.32) |
| Kuwait |  | 28.2 (26.2 to 30.4) | 10.7 (8.7 to 12.9) | -3.23 (-3.88 to -2.58) |
| Kyrgyzstan |  | 30.0 (27.2 to 32.9) | 15.4 (12.9 to 18.1) | -0.66 (-1.43 to 0.11) |
| Lao People's Democratic Republic |  | 26.6 (21.1 to 34.1) | 16.2 (12.2 to 21.1) | -1.68 (-1.81 to -1.56) |
| Latvia |  | 36.4 (35.1 to 37.6) | 6.6 (5.8 to 7.3) | -5.86 (-6.28 to -5.44) |
| Lebanon |  | 16.5 (13.8 to 19.9) | 8.0 (6.8 to 9.6) | -1.87 (-2.08 to -1.66) |
| Lesotho |  | 29.1 (23.4 to 35.8) | 54.7 (41.5 to 69.5) | 2.85 (2.23 to 3.47) |
| Liberia |  | 24.0 (19.1 to 28.7) | 19.2 (14.4 to 26.8) | -0.86 (-1.07 to -0.65) |
| Libya |  | 46.6 (37.8 to 55.5) | 40.2 (31.7 to 50.5) | -0.23 (-0.37 to -0.09) |
| Lithuania |  | 29.8 (28.8 to 30.7) | 6.6 (5.9 to 7.2) | -4.88 (-5.52 to -4.24) |
| Luxembourg |  | 19.8 (19.0 to 20.6) | 3.3 (3.0 to 3.7) | -6.21 (-6.48 to -5.94) |
| Madagascar |  | 25.2 (21.5 to 28.6) | 19.5 (15.2 to 24.1) | -0.83 (-0.89 to -0.77) |
| Malaysia |  | 29.3 (27.2 to 31.7) | 23.7 (21.8 to 25.7) | -0.81 (-0.98 to -0.64) |
| Maldives |  | 8.2 (7.0 to 9.9) | 2.7 (2.3 to 3.3) | -3.50 (-3.60 to -3.40) |
| Mali |  | 31.5 (26.5 to 36.8) | 22.5 (18.0 to 27.3) | -1.01 (-1.08 to -0.94) |
| Malta |  | 6.6 (6.2 to 6.9) | 1.9 (1.7 to 2.1) | -4.48 (-4.73 to -4.23) |
| Mauritania |  | 51.1 (42.9 to 59.9) | 31.3 (24.7 to 40.3) | -1.72 (-1.80 to -1.63) |
| Micronesia |  | 25.6 (20.4 to 31.9) | 20.9 (16.2 to 26.6) | -0.65 (-0.68 to -0.62) |
| Mongolia |  | 14.0 (12.0 to 16.6) | 15.3 (13.3 to 17.4) | 0.62 (0.21 to 1.02) |
| Montenegro |  | 9.3 (8.4 to 10.3) | 6.6 (5.7 to 7.5) | -1.01 (-1.35 to -0.66) |
| Morocco |  | 41.9 (35.8 to 48.0) | 23.0 (18.2 to 29.7) | -1.98 (-2.04 to -1.93) |
| Mozambique |  | 31.6 (26.6 to 38.0) | 33.3 (25.1 to 41.8) | 0.56 (0.36 to 0.77) |
| Myanmar |  | 29.5 (23.4 to 37.5) | 14.5 (11.5 to 18.4) | -2.69 (-2.88 to -2.50) |
| Namibia |  | 33.7 (28.5 to 39.2) | 32.7 (24.1 to 44.6) | -0.30 (-0.71 to 0.12) |
| Nepal |  | 12.0 (9.9 to 14.6) | 8.5 (6.9 to 10.7) | -1.04 (-1.26 to -0.83) |
| New Zealand |  | 22.3 (21.8 to 22.7) | 6.9 (6.6 to 7.2) | -3.84 (-4.17 to -3.50) |
| Nicaragua |  | 18.1 (16.9 to 19.1) | 10.5 (9.0 to 12.4) | -1.61 (-1.82 to -1.41) |
| Niger |  | 23.4 (18.8 to 27.7) | 16.7 (12.7 to 21.7) | -1.11 (-1.24 to -0.97) |
| Nigeria |  | 22.8 (18.9 to 27.1) | 15.8 (12.2 to 20.0) | -1.31 (-1.41 to -1.20) |
| Niue |  | 16.5 (12.6 to 21.9) | 19.4 (16.5 to 23.8) | -0.52 (-0.85 to -0.19) |
| North Macedonia |  | 9.8 (9.1 to 10.6) | 4.9 (4.1 to 5.9) | -2.31 (-2.57 to -2.05) |
| Oman |  | 94.4 (73.6 to 119.4) | 35.6 (29.7 to 42.4) | -2.73 (-3.05 to -2.40) |
| Pakistan |  | 13.4 (11.2 to 15.2) | 19.1 (15.6 to 23.2) | 1.02 (0.69 to 1.35) |
| Panama |  | 22.2 (21.3 to 23.2) | 12.0 (9.7 to 14.2) | -2.09 (-2.27 to -1.91) |
| Papua New Guinea |  | 22.6 (15.7 to 29.7) | 18.5 (14.6 to 22.5) | -0.63 (-0.69 to -0.56) |
| Peru |  | 19.6 (17.3 to 22.2) | 14.6 (11.5 to 18.1) | -0.98 (-1.19 to -0.78) |
| Philippines |  | 12.9 (11.8 to 14.1) | 11.8 (9.9 to 13.8) | -0.04 (-0.18 to 0.09) |
| Poland |  | 23.5 (22.8 to 24.2) | 8.2 (7.5 to 8.9) | -3.80 (-4.04 to -3.55) |
| Portugal |  | 31.1 (30.3 to 31.8) | 5.4 (5.1 to 5.7) | -6.39 (-6.78 to -6.00) |
| Qatar |  | 58.9 (50.1 to 69.8) | 21.1 (16.5 to 26.1) | -3.34 (-4.14 to -2.53) |
| Republic of Korea |  | 37.8 (35.7 to 39.5) | 6.6 (5.9 to 7.7) | -6.42 (-6.68 to -6.15) |
| Republic of Moldova |  | 29.6 (28.7 to 30.5) | 7.7 (6.9 to 8.6) | -3.43 (-3.85 to -3.01) |
| Romania |  | 21.6 (20.8 to 22.4) | 9.1 (8.1 to 9.9) | -2.73 (-3.03 to -2.43) |
| Russian Federation |  | 26.7 (26.2 to 26.9) | 10.5 (9.7 to 11.2) | -2.91 (-3.49 to -2.32) |
| Rwanda |  | 59.2 (49.8 to 71.7) | 31.0 (22.7 to 41.6) | -2.98 (-3.31 to -2.65) |
| Samoa |  | 18.3 (14.4 to 23.2) | 16.6 (12.7 to 21.1) | -0.23 (-0.43 to -0.02) |
| Sao Tome and Principe |  | 15.0 (12.4 to 17.5) | 13.9 (10.6 to 18.0) | -0.26 (-0.44 to -0.08) |
| Saudi Arabia |  | 60.3 (44.8 to 76.3) | 44.7 (36.0 to 55.7) | -0.78 (-0.90 to -0.66) |
| Senegal |  | 27.4 (22.7 to 32.5) | 21.4 (17.0 to 27.2) | -0.80 (-0.91 to -0.69) |
| Serbia |  | 13.7 (12.4 to 15.0) | 5.4 (4.6 to 6.3) | -3.22 (-3.47 to -2.98) |
| Seychelles |  | 12.0 (10.9 to 13.0) | 10.0 (8.9 to 11.3) | -0.18 (-0.54 to 0.17) |
| Sierra Leone |  | 26.7 (20.9 to 32.6) | 21.7 (16.8 to 28.0) | -0.42 (-0.55 to -0.28) |
| Singapore |  | 11.2 (10.8 to 11.6) | 1.9 (1.7 to 2.0) | -6.04 (-6.25 to -5.84) |
| Slovakia |  | 17.9 (16.7 to 19.0) | 6.0 (5.1 to 7.0) | -3.75 (-3.96 to -3.55) |
| Slovenia |  | 19.9 (19.1 to 20.7) | 4.1 (3.6 to 4.6) | -5.50 (-5.79 to -5.22) |
| Solomon Islands |  | 22.9 (13.8 to 31.8) | 20.4 (15.7 to 26.4) | -0.41 (-0.48 to -0.35) |
| Somalia |  | 37.1 (27.6 to 48.0) | 34.4 (25.4 to 44.9) | -0.28 (-0.42 to -0.15) |
| South Africa |  | 55.1 (51.3 to 58.8) | 38.9 (35.9 to 41.8) | -1.23 (-1.63 to -0.83) |
| South Sudan |  | 28.8 (22.6 to 36.1) | 27.9 (20.6 to 36.4) | -0.32 (-0.59 to -0.05) |
| Sri Lanka |  | 19.6 (17.8 to 21.5) | 10.5 (7.4 to 13.7) | -2.26 (-2.53 to -1.99) |
| Sudan |  | 41.5 (33.6 to 50.4) | 24.3 (18.6 to 32.3) | -1.83 (-1.86 to -1.79) |
| Suriname |  | 26.1 (21.4 to 28.5) | 16.2 (13.1 to 19.6) | -1.52 (-1.80 to -1.24) |
| Syrian Arab Republic |  | 20.1 (16.7 to 24.0) | 12.9 (10.1 to 16.4) | -1.62 (-1.77 to -1.47) |
| Tajikistan |  | 14.6 (13.0 to 16.3) | 6.8 (5.6 to 8.3) | -2.31 (-2.74 to -1.87) |
| Thailand |  | 38.2 (33.9 to 42.8) | 29.7 (23.8 to 36.9) | -1.85 (-2.29 to -1.42) |
| Timor-Leste |  | 15.4 (12.3 to 18.7) | 13.2 (10.2 to 16.4) | -0.42 (-0.85 to 0.01) |
| Togo |  | 28.7 (23.5 to 34.2) | 23.8 (17.3 to 31.2) | -0.56 (-0.70 to -0.41) |
| Tonga |  | 12.5 (10.3 to 15.7) | 11.7 (8.9 to 15.9) | -0.22 (-0.39 to -0.04) |
| Trinidad and Tobago |  | 17.6 (16.9 to 18.3) | 13.1 (10.3 to 16.6) | -1.16 (-1.38 to -0.94) |
| Tunisia |  | 31.4 (27.2 to 35.6) | 19.7 (14.7 to 25.6) | -1.66 (-1.77 to -1.54) |
| Turkey |  | 18.7 (16.2 to 21.3) | 9.2 (7.7 to 11.0) | -2.00 (-2.46 to -1.55) |
| Uganda |  | 28.5 (22.4 to 35.0) | 26.7 (21.4 to 33.1) | -0.73 (-1.02 to -0.44) |
| Ukraine |  | 25.7 (24.9 to 26.6) | 10.3 (7.6 to 13.3) | -3.12 (-3.68 to -2.55) |
| United Arab Emirates |  | 48.5 (38.8 to 61.7) | 20.2 (15.7 to 25.3) | -2.03 (-2.55 to -1.51) |
| United Republic of Tanzania |  | 24.2 (20.8 to 27.7) | 17.6 (14.1 to 22.0) | -1.23 (-1.34 to -1.12) |
| Uruguay |  | 13.6 (13.1 to 14.1) | 13.5 (12.8 to 14.4) | -0.10 (-0.43 to 0.23) |
| Uzbekistan |  | 21.3 (20.5 to 22.0) | 11.0 (9.6 to 12.7) | -0.71 (-1.26 to -0.16) |
| Vanuatu |  | 21.4 (15.5 to 28.8) | 20.5 (16.2 to 25.1) | -0.35 (-0.44 to -0.26) |
| Venezuela |  | 29.6 (28.8 to 30.5) | 25.4 (19.5 to 32.3) | -0.68 (-1.01 to -0.36) |
| Viet Nam |  | 27.6 (21.9 to 34.2) | 21.7 (17.0 to 26.7) | -0.32 (-0.54 to -0.09) |
| Yemen |  | 69.5 (47.3 to 90.5) | 43.0 (31.0 to 57.2) | -1.81 (-1.94 to -1.67) |
| Zambia |  | 29.5 (25.4 to 34.2) | 23.5 (17.8 to 30.3) | -1.14 (-1.41 to -0.87) |
| Zimbabwe |  | 25.0 (21.1 to 29.6) | 26.4 (20.8 to 32.8) | 0.14 (-0.25 to 0.54) |

BRI countries: the Belt and Road countries; EAPC: the estimated annual percentage change.

| Supplementary Table 19. Achievement of SDGs for maternal mortality ratio in 149 BRI countries | | | |
| --- | --- | --- | --- |
| Country |  | 2021 | 2030 |
| Afghanistan |  | Not achieved | Not achieved yet |
| Albania |  | Achieved | Achieved |
| Algeria |  | Not achieved | Achieved |
| Angola |  | Not achieved | Not achieved yet |
| Antigua and Barbuda |  | Achieved | Achieved |
| Argentina |  | Achieved | Achieved |
| Armenia |  | Achieved | Achieved |
| Austria |  | Achieved | Achieved |
| Azerbaijan |  | Achieved | Achieved |
| Bahrain |  | Achieved | Achieved |
| Bangladesh |  | Not achieved | Not achieved yet |
| Barbados |  | Achieved | Achieved |
| Belarus |  | Achieved | Achieved |
| Benin |  | Not achieved | Not achieved yet |
| Bolivia (Plurinational State of) |  | Not achieved | Not achieved yet |
| Bosnia and Herzegovina |  | Achieved | Achieved |
| Botswana |  | Not achieved | Achieved |
| Brunei Darussalam |  | Achieved | Achieved |
| Bulgaria |  | Achieved | Achieved |
| Burkina Faso |  | Not achieved | Not achieved yet |
| Burundi |  | Not achieved | Not achieved yet |
| Cabo Verde |  | Achieved | Achieved |
| Cambodia |  | Not achieved | Not achieved yet |
| Cameroon |  | Not achieved | Not achieved yet |
| Central African Republic |  | Not achieved | Not achieved yet |
| Chad |  | Not achieved | Not achieved yet |
| Chile |  | Achieved | Achieved |
| China |  | Achieved | Achieved |
| Comoros |  | Not achieved | Not achieved yet |
| Congo |  | Not achieved | Not achieved yet |
| Cook Islands |  | Achieved | Achieved |
| Costa Rica |  | Achieved | Achieved |
| Côte d'Ivoire |  | Not achieved | Not achieved yet |
| Croatia |  | Achieved | Achieved |
| Cuba |  | Achieved | Achieved |
| Cyprus |  | Achieved | Achieved |
| Czechia |  | Achieved | Achieved |
| Democratic Republic of the Congo |  | Not achieved | Not achieved yet |
| Djibouti |  | Not achieved | Not achieved yet |
| Dominica |  | Not achieved | Not achieved yet |
| Dominican Republic |  | Not achieved | Not achieved yet |
| Ecuador |  | Not achieved | Not achieved yet |
| Egypt |  | Achieved | Achieved |
| El Salvador |  | Achieved | Achieved |
| Equatorial Guinea |  | Not achieved | Not achieved yet |
| Eritrea |  | Not achieved | Not achieved yet |
| Estonia |  | Achieved | Achieved |
| Ethiopia |  | Not achieved | Not achieved yet |
| Fiji |  | Not achieved | Not achieved yet |
| Gabon |  | Not achieved | Not achieved yet |
| Gambia |  | Not achieved | Not achieved yet |
| Georgia |  | Achieved | Achieved |
| Ghana |  | Not achieved | Not achieved yet |
| Greece |  | Achieved | Achieved |
| Grenada |  | Not achieved | Not achieved yet |
| Guinea |  | Not achieved | Not achieved yet |
| Guinea-Bissau |  | Not achieved | Not achieved yet |
| Guyana |  | Not achieved | Not achieved yet |
| Hungary |  | Achieved | Achieved |
| Indonesia |  | Not achieved | Not achieved yet |
| Iran (Islamic Republic of) |  | Achieved | Achieved |
| Iraq |  | Achieved | Achieved |
| Italy |  | Achieved | Achieved |
| Jamaica |  | Not achieved | Not achieved yet |
| Kazakhstan |  | Achieved | Achieved |
| Kenya |  | Not achieved | Not achieved yet |
| Kiribati |  | Not achieved | Not achieved yet |
| Kuwait |  | Achieved | Achieved |
| Kyrgyzstan |  | Achieved | Achieved |
| Lao People's Democratic Republic |  | Not achieved | Not achieved yet |
| Latvia |  | Achieved | Achieved |
| Lebanon |  | Achieved | Achieved |
| Lesotho |  | Not achieved | Not achieved yet |
| Liberia |  | Not achieved | Not achieved yet |
| Libya |  | Achieved | Achieved |
| Lithuania |  | Achieved | Achieved |
| Luxembourg |  | Achieved | Achieved |
| Madagascar |  | Not achieved | Not achieved yet |
| Malaysia |  | Achieved | Achieved |
| Maldives |  | Achieved | Achieved |
| Mali |  | Not achieved | Not achieved yet |
| Malta |  | Achieved | Achieved |
| Mauritania |  | Not achieved | Not achieved yet |
| Micronesia (Federated States of) |  | Not achieved | Not achieved yet |
| Mongolia |  | Achieved | Achieved |
| Montenegro |  | Achieved | Achieved |
| Morocco |  | Not achieved | Achieved |
| Mozambique |  | Not achieved | Not achieved yet |
| Myanmar |  | Not achieved | Not achieved yet |
| Namibia |  | Not achieved | Not achieved yet |
| Nepal |  | Not achieved | Not achieved yet |
| New Zealand |  | Achieved | Achieved |
| Nicaragua |  | Achieved | Achieved |
| Niger |  | Not achieved | Not achieved yet |
| Nigeria |  | Not achieved | Not achieved yet |
| Niue |  | Achieved | Achieved |
| North Macedonia |  | Achieved | Achieved |
| Oman |  | Achieved | Achieved |
| Pakistan |  | Not achieved | Not achieved yet |
| Panama |  | Not achieved | Achieved |
| Papua New Guinea |  | Not achieved | Not achieved yet |
| Peru |  | Not achieved | Not achieved yet |
| Philippines |  | Not achieved | Not achieved yet |
| Poland |  | Achieved | Achieved |
| Portugal |  | Achieved | Achieved |
| Qatar |  | Achieved | Achieved |
| Republic of Korea |  | Achieved | Achieved |
| Republic of Moldova |  | Achieved | Achieved |
| Romania |  | Achieved | Achieved |
| Russian Federation |  | Achieved | Achieved |
| Rwanda |  | Not achieved | Not achieved yet |
| Samoa |  | Achieved | Achieved |
| Sao Tome and Principe |  | Not achieved | Achieved |
| Saudi Arabia |  | Not achieved | Not achieved yet |
| Senegal |  | Not achieved | Not achieved yet |
| Serbia |  | Achieved | Achieved |
| Seychelles |  | Achieved | Achieved |
| Sierra Leone |  | Not achieved | Not achieved yet |
| Singapore |  | Achieved | Achieved |
| Slovakia |  | Achieved | Achieved |
| Slovenia |  | Achieved | Achieved |
| Solomon Islands |  | Not achieved | Not achieved yet |
| Somalia |  | Not achieved | Not achieved yet |
| South Africa |  | Not achieved | Not achieved yet |
| South Sudan |  | Not achieved | Not achieved yet |
| Sri Lanka |  | Achieved | Achieved |
| Sudan |  | Not achieved | Not achieved yet |
| Suriname |  | Not achieved | Not achieved yet |
| Syrian Arab Republic |  | Achieved | Achieved |
| Tajikistan |  | Achieved | Achieved |
| Thailand |  | Achieved | Achieved |
| Timor-Leste |  | Not achieved | Not achieved yet |
| Togo |  | Not achieved | Not achieved yet |
| Tonga |  | Not achieved | Not achieved yet |
| Trinidad and Tobago |  | Not achieved | Not achieved yet |
| Tunisia |  | Achieved | Achieved |
| Turkey |  | Achieved | Achieved |
| Uganda |  | Not achieved | Not achieved yet |
| Ukraine |  | Achieved | Achieved |
| United Arab Emirates |  | Achieved | Achieved |
| United Republic of Tanzania |  | Not achieved | Not achieved yet |
| Uruguay |  | Achieved | Achieved |
| Uzbekistan |  | Achieved | Achieved |
| Vanuatu |  | Not achieved | Not achieved yet |
| Venezuela (Bolivarian Republic of) |  | Not achieved | Not achieved yet |
| Viet Nam |  | Achieved | Achieved |
| Yemen |  | Not achieved | Not achieved yet |
| Zambia |  | Not achieved | Not achieved yet |
| Zimbabwe |  | Not achieved | Not achieved yet |

Note: BRI countries: the Belt and Road countries.

The achievement of SDGs in 2021 was assessed based on the GBD estimate for 2021, and the future achievement by 2030 was the projected value from the health-related SDGs visualizations in GBD 2021 (https://vizhub.healthdata.org/sdg/).

| Supplementary Table 20**.** Achievement of SDGs for under-five mortality rate in 149 BRI countries | | | |
| --- | --- | --- | --- |
| Country |  | 2021 | 2030 |
| Afghanistan |  | Not achieved | Not achieved yet |
| Albania |  | Achieved | Achieved |
| Algeria |  | Achieved | Achieved |
| Angola |  | Not achieved | Not achieved yet |
| Antigua and Barbuda |  | Achieved | Achieved |
| Argentina |  | Achieved | Achieved |
| Armenia |  | Achieved | Achieved |
| Austria |  | Achieved | Achieved |
| Azerbaijan |  | Not achieved | Achieved |
| Bahrain |  | Achieved | Achieved |
| Bangladesh |  | Not achieved | Achieved |
| Barbados |  | Achieved | Achieved |
| Belarus |  | Achieved | Achieved |
| Benin |  | Not achieved | Not achieved yet |
| Bolivia (Plurinational State of) |  | Not achieved | Achieved |
| Bosnia and Herzegovina |  | Achieved | Achieved |
| Botswana |  | Not achieved | Not achieved yet |
| Brunei Darussalam |  | Achieved | Achieved |
| Bulgaria |  | Achieved | Achieved |
| Burkina Faso |  | Not achieved | Not achieved yet |
| Burundi |  | Not achieved | Not achieved yet |
| Cabo Verde |  | Achieved | Achieved |
| Cambodia |  | Not achieved | Achieved |
| Cameroon |  | Not achieved | Not achieved yet |
| Central African Republic |  | Not achieved | Not achieved yet |
| Chad |  | Not achieved | Not achieved yet |
| Chile |  | Achieved | Achieved |
| China |  | Achieved | Achieved |
| Comoros |  | Not achieved | Not achieved yet |
| Congo |  | Not achieved | Not achieved yet |
| Cook Islands |  | Achieved | Achieved |
| Costa Rica |  | Achieved | Achieved |
| Côte d'Ivoire |  | Not achieved | Not achieved yet |
| Croatia |  | Achieved | Achieved |
| Cuba |  | Achieved | Achieved |
| Cyprus |  | Achieved | Achieved |
| Czechia |  | Achieved | Achieved |
| Democratic Republic of the Congo |  | Not achieved | Not achieved yet |
| Djibouti |  | Not achieved | Not achieved yet |
| Dominica |  | Not achieved | Not achieved yet |
| Dominican Republic |  | Achieved | Achieved |
| Ecuador |  | Achieved | Achieved |
| Egypt |  | Achieved | Achieved |
| El Salvador |  | Achieved | Achieved |
| Equatorial Guinea |  | Not achieved | Not achieved yet |
| Eritrea |  | Not achieved | Not achieved yet |
| Estonia |  | Achieved | Achieved |
| Ethiopia |  | Not achieved | Not achieved yet |
| Fiji |  | Achieved | Achieved |
| Gabon |  | Not achieved | Achieved |
| Gambia |  | Not achieved | Not achieved yet |
| Georgia |  | Achieved | Achieved |
| Ghana |  | Not achieved | Not achieved yet |
| Greece |  | Achieved | Achieved |
| Grenada |  | Achieved | Achieved |
| Guinea |  | Not achieved | Not achieved yet |
| Guinea-Bissau |  | Not achieved | Not achieved yet |
| Guyana |  | Achieved | Achieved |
| Hungary |  | Achieved | Achieved |
| Indonesia |  | Achieved | Achieved |
| Iran (Islamic Republic of) |  | Achieved | Achieved |
| Iraq |  | Achieved | Achieved |
| Italy |  | Achieved | Achieved |
| Jamaica |  | Achieved | Achieved |
| Kazakhstan |  | Achieved | Achieved |
| Kenya |  | Not achieved | Not achieved yet |
| Kiribati |  | Not achieved | Not achieved yet |
| Kuwait |  | Achieved | Achieved |
| Kyrgyzstan |  | Achieved | Achieved |
| Lao People's Democratic Republic |  | Not achieved | Not achieved yet |
| Latvia |  | Achieved | Achieved |
| Lebanon |  | Achieved | Achieved |
| Lesotho |  | Not achieved | Not achieved yet |
| Liberia |  | Not achieved | Not achieved yet |
| Libya |  | Achieved | Achieved |
| Lithuania |  | Achieved | Achieved |
| Luxembourg |  | Achieved | Achieved |
| Madagascar |  | Not achieved | Not achieved yet |
| Malaysia |  | Achieved | Achieved |
| Maldives |  | Achieved | Achieved |
| Mali |  | Not achieved | Not achieved yet |
| Malta |  | Achieved | Achieved |
| Mauritania |  | Not achieved | Achieved |
| Micronesia (Federated States of) |  | Achieved | Achieved |
| Mongolia |  | Achieved | Achieved |
| Montenegro |  | Achieved | Achieved |
| Morocco |  | Achieved | Achieved |
| Mozambique |  | Not achieved | Not achieved yet |
| Myanmar |  | Not achieved | Not achieved yet |
| Namibia |  | Not achieved | Not achieved yet |
| Nepal |  | Not achieved | Achieved |
| New Zealand |  | Achieved | Achieved |
| Nicaragua |  | Achieved | Achieved |
| Niger |  | Not achieved | Not achieved yet |
| Nigeria |  | Not achieved | Not achieved yet |
| Niue |  | Achieved | Achieved |
| North Macedonia |  | Achieved | Achieved |
| Oman |  | Achieved | Achieved |
| Pakistan |  | Not achieved | Not achieved yet |
| Panama |  | Achieved | Achieved |
| Papua New Guinea |  | Not achieved | Not achieved yet |
| Peru |  | Achieved | Achieved |
| Philippines |  | Achieved | Achieved |
| Poland |  | Achieved | Achieved |
| Portugal |  | Achieved | Achieved |
| Qatar |  | Achieved | Achieved |
| Republic of Korea |  | Achieved | Achieved |
| Republic of Moldova |  | Achieved | Achieved |
| Romania |  | Achieved | Achieved |
| Russian Federation |  | Achieved | Achieved |
| Rwanda |  | Not achieved | Not achieved yet |
| Samoa |  | Achieved | Achieved |
| Sao Tome and Principe |  | Achieved | Achieved |
| Saudi Arabia |  | Achieved | Achieved |
| Senegal |  | Not achieved | Not achieved yet |
| Serbia |  | Achieved | Achieved |
| Seychelles |  | Achieved | Achieved |
| Sierra Leone |  | Not achieved | Not achieved yet |
| Singapore |  | Achieved | Achieved |
| Slovakia |  | Achieved | Achieved |
| Slovenia |  | Achieved | Achieved |
| Solomon Islands |  | Achieved | Achieved |
| Somalia |  | Not achieved | Not achieved yet |
| South Africa |  | Not achieved | Not achieved yet |
| South Sudan |  | Not achieved | Not achieved yet |
| Sri Lanka |  | Achieved | Achieved |
| Sudan |  | Not achieved | Not achieved yet |
| Suriname |  | Achieved | Achieved |
| Syrian Arab Republic |  | Achieved | Achieved |
| Tajikistan |  | Not achieved | Not achieved yet |
| Thailand |  | Achieved | Achieved |
| Timor-Leste |  | Not achieved | Achieved |
| Togo |  | Not achieved | Not achieved yet |
| Tonga |  | Achieved | Achieved |
| Trinidad and Tobago |  | Achieved | Achieved |
| Tunisia |  | Achieved | Achieved |
| Turkey |  | Achieved | Achieved |
| Uganda |  | Not achieved | Not achieved yet |
| Ukraine |  | Achieved | Achieved |
| United Arab Emirates |  | Achieved | Achieved |
| United Republic of Tanzania |  | Not achieved | Not achieved yet |
| Uruguay |  | Achieved | Achieved |
| Uzbekistan |  | Achieved | Achieved |
| Vanuatu |  | Achieved | Achieved |
| Venezuela (Bolivarian Republic of) |  | Achieved | Not achieved yet |
| Viet Nam |  | Achieved | Achieved |
| Yemen |  | Not achieved | Not achieved yet |
| Zambia |  | Not achieved | Not achieved yet |
| Zimbabwe |  | Not achieved | Not achieved yet |

Note: BRI countries: the Belt and Road countries.

The achievement of SDGs in 2021 was assessed based on the GBD estimate for 2021, and the future achievement by 2030 was the projected value from the health-related SDGs visualizations in GBD 2021 (https://vizhub.healthdata.org/sdg/).

| Supplementary Table 21. Achievement of SDGs for neonatal mortality rate in 149 BRI countries | | | |
| --- | --- | --- | --- |
| Country |  | 2021 | 2030 |
| Afghanistan |  | Not achieved | Not achieved yet |
| Albania |  | Achieved | Achieved |
| Algeria |  | Achieved | Achieved |
| Angola |  | Not achieved | Not achieved yet |
| Antigua and Barbuda |  | Achieved | Achieved |
| Argentina |  | Achieved | Achieved |
| Armenia |  | Achieved | Achieved |
| Austria |  | Achieved | Achieved |
| Azerbaijan |  | Not achieved | Not achieved yet |
| Bahrain |  | Achieved | Achieved |
| Bangladesh |  | Not achieved | Not achieved yet |
| Barbados |  | Achieved | Achieved |
| Belarus |  | Achieved | Achieved |
| Benin |  | Not achieved | Not achieved yet |
| Bolivia (Plurinational State of) |  | Not achieved | Achieved |
| Bosnia and Herzegovina |  | Achieved | Achieved |
| Botswana |  | Not achieved | Not achieved yet |
| Brunei Darussalam |  | Achieved | Achieved |
| Bulgaria |  | Achieved | Achieved |
| Burkina Faso |  | Not achieved | Not achieved yet |
| Burundi |  | Not achieved | Not achieved yet |
| Cabo Verde |  | Achieved | Achieved |
| Cambodia |  | Not achieved | Not achieved yet |
| Cameroon |  | Not achieved | Not achieved yet |
| Central African Republic |  | Not achieved | Not achieved yet |
| Chad |  | Not achieved | Not achieved yet |
| Chile |  | Achieved | Achieved |
| China |  | Achieved | Achieved |
| Comoros |  | Not achieved | Not achieved yet |
| Congo |  | Not achieved | Not achieved yet |
| Cook Islands |  | Achieved | Achieved |
| Costa Rica |  | Achieved | Achieved |
| Côte d'Ivoire |  | Not achieved | Not achieved yet |
| Croatia |  | Achieved | Achieved |
| Cuba |  | Achieved | Achieved |
| Cyprus |  | Achieved | Achieved |
| Czechia |  | Achieved | Achieved |
| Democratic Republic of the Congo |  | Not achieved | Not achieved yet |
| Djibouti |  | Not achieved | Not achieved yet |
| Dominica |  | Not achieved | Not achieved yet |
| Dominican Republic |  | Not achieved | Not achieved yet |
| Ecuador |  | Achieved | Achieved |
| Egypt |  | Achieved | Achieved |
| El Salvador |  | Achieved | Achieved |
| Equatorial Guinea |  | Not achieved | Not achieved yet |
| Eritrea |  | Not achieved | Not achieved yet |
| Estonia |  | Achieved | Achieved |
| Ethiopia |  | Not achieved | Not achieved yet |
| Fiji |  | Achieved | Achieved |
| Gabon |  | Not achieved | Not achieved yet |
| Gambia |  | Not achieved | Not achieved yet |
| Georgia |  | Achieved | Achieved |
| Ghana |  | Not achieved | Not achieved yet |
| Greece |  | Achieved | Achieved |
| Grenada |  | Achieved | Achieved |
| Guinea |  | Not achieved | Not achieved yet |
| Guinea-Bissau |  | Not achieved | Not achieved yet |
| Guyana |  | Not achieved | Achieved |
| Hungary |  | Achieved | Achieved |
| Indonesia |  | Not achieved | Achieved |
| Iran (Islamic Republic of) |  | Achieved | Achieved |
| Iraq |  | Achieved | Achieved |
| Italy |  | Achieved | Achieved |
| Jamaica |  | Achieved | Achieved |
| Kazakhstan |  | Achieved | Achieved |
| Kenya |  | Not achieved | Not achieved yet |
| Kiribati |  | Not achieved | Not achieved yet |
| Kuwait |  | Achieved | Achieved |
| Kyrgyzstan |  | Achieved | Achieved |
| Lao People's Democratic Republic |  | Not achieved | Not achieved yet |
| Latvia |  | Achieved | Achieved |
| Lebanon |  | Achieved | Achieved |
| Lesotho |  | Not achieved | Not achieved yet |
| Liberia |  | Not achieved | Not achieved yet |
| Libya |  | Achieved | Achieved |
| Lithuania |  | Achieved | Achieved |
| Luxembourg |  | Achieved | Achieved |
| Madagascar |  | Not achieved | Not achieved yet |
| Malaysia |  | Achieved | Achieved |
| Maldives |  | Achieved | Achieved |
| Mali |  | Not achieved | Not achieved yet |
| Malta |  | Achieved | Achieved |
| Mauritania |  | Not achieved | Not achieved yet |
| Micronesia (Federated States of) |  | Achieved | Achieved |
| Mongolia |  | Achieved | Achieved |
| Montenegro |  | Achieved | Achieved |
| Morocco |  | Achieved | Achieved |
| Mozambique |  | Not achieved | Not achieved yet |
| Myanmar |  | Not achieved | Not achieved yet |
| Namibia |  | Not achieved | Not achieved yet |
| Nepal |  | Not achieved | Not achieved yet |
| New Zealand |  | Achieved | Achieved |
| Nicaragua |  | Achieved | Achieved |
| Niger |  | Not achieved | Not achieved yet |
| Nigeria |  | Not achieved | Not achieved yet |
| Niue |  | Achieved | Achieved |
| North Macedonia |  | Achieved | Achieved |
| Oman |  | Achieved | Achieved |
| Pakistan |  | Not achieved | Not achieved yet |
| Panama |  | Achieved | Achieved |
| Papua New Guinea |  | Not achieved | Not achieved yet |
| Peru |  | Achieved | Achieved |
| Philippines |  | Achieved | Achieved |
| Poland |  | Achieved | Achieved |
| Portugal |  | Achieved | Achieved |
| Qatar |  | Achieved | Achieved |
| Republic of Korea |  | Achieved | Achieved |
| Republic of Moldova |  | Achieved | Achieved |
| Romania |  | Achieved | Achieved |
| Russian Federation |  | Achieved | Achieved |
| Rwanda |  | Not achieved | Not achieved yet |
| Samoa |  | Achieved | Achieved |
| Sao Tome and Principe |  | Achieved | Achieved |
| Saudi Arabia |  | Achieved | Achieved |
| Senegal |  | Not achieved | Not achieved yet |
| Serbia |  | Achieved | Achieved |
| Seychelles |  | Achieved | Achieved |
| Sierra Leone |  | Not achieved | Not achieved yet |
| Singapore |  | Achieved | Achieved |
| Slovakia |  | Achieved | Achieved |
| Slovenia |  | Achieved | Achieved |
| Solomon Islands |  | Achieved | Achieved |
| Somalia |  | Not achieved | Not achieved yet |
| South Africa |  | Not achieved | Not achieved yet |
| South Sudan |  | Not achieved | Not achieved yet |
| Sri Lanka |  | Achieved | Achieved |
| Sudan |  | Not achieved | Not achieved yet |
| Suriname |  | Not achieved | Not achieved yet |
| Syrian Arab Republic |  | Achieved | Achieved |
| Tajikistan |  | Not achieved | Not achieved yet |
| Thailand |  | Achieved | Achieved |
| Timor-Leste |  | Not achieved | Achieved |
| Togo |  | Not achieved | Not achieved yet |
| Tonga |  | Achieved | Achieved |
| Trinidad and Tobago |  | Achieved | Achieved |
| Tunisia |  | Achieved | Achieved |
| Turkey |  | Achieved | Achieved |
| Uganda |  | Not achieved | Not achieved yet |
| Ukraine |  | Achieved | Achieved |
| United Arab Emirates |  | Achieved | Achieved |
| United Republic of Tanzania |  | Not achieved | Not achieved yet |
| Uruguay |  | Achieved | Achieved |
| Uzbekistan |  | Achieved | Achieved |
| Vanuatu |  | Achieved | Achieved |
| Venezuela (Bolivarian Republic of) |  | Not achieved | Not achieved yet |
| Viet Nam |  | Achieved | Achieved |
| Yemen |  | Not achieved | Not achieved yet |
| Zambia |  | Not achieved | Not achieved yet |
| Zimbabwe |  | Not achieved | Not achieved yet |

Note: BRI countries: the Belt and Road countries.

The achievement of SDGs in 2021 was assessed based on the GBD estimate for 2021, and the future achievement by 2030 was the projected value from the health-related SDGs visualizations in GBD 2021 (https://vizhub.healthdata.org/sdg/).

| Supplementary Table 22. Achievement of SDGs for HIV incidence rate in 149 BRI countries | | | |
| --- | --- | --- | --- |
| Country |  | 2021 | 2030 |
| Afghanistan |  | Not achieved | Achieved |
| Albania |  | Achieved | Achieved |
| Algeria |  | Not achieved | Not achieved yet |
| Angola |  | Not achieved | Not achieved yet |
| Antigua and Barbuda |  | Not achieved | Not achieved yet |
| Argentina |  | Not achieved | Not achieved yet |
| Armenia |  | Not achieved | Not achieved yet |
| Austria |  | Not achieved | Not achieved yet |
| Azerbaijan |  | Not achieved | Not achieved yet |
| Bahrain |  | Not achieved | Not achieved yet |
| Bangladesh |  | Not achieved | Not achieved yet |
| Barbados |  | Not achieved | Not achieved yet |
| Belarus |  | Not achieved | Not achieved yet |
| Benin |  | Not achieved | Not achieved yet |
| Bolivia (Plurinational State of) |  | Not achieved | Not achieved yet |
| Bosnia and Herzegovina |  | Achieved | Achieved |
| Botswana |  | Not achieved | Not achieved yet |
| Brunei Darussalam |  | Not achieved | Not achieved yet |
| Bulgaria |  | Not achieved | Not achieved yet |
| Burkina Faso |  | Not achieved | Not achieved yet |
| Burundi |  | Not achieved | Achieved |
| Cabo Verde |  | Not achieved | Not achieved yet |
| Cambodia |  | Not achieved | Not achieved yet |
| Cameroon |  | Not achieved | Not achieved yet |
| Central African Republic |  | Not achieved | Not achieved yet |
| Chad |  | Not achieved | Not achieved yet |
| Chile |  | Not achieved | Not achieved yet |
| China |  | Not achieved | Not achieved yet |
| Comoros |  | Achieved | Achieved |
| Congo |  | Not achieved | Not achieved yet |
| Cook Islands |  | Not achieved | Not achieved yet |
| Costa Rica |  | Not achieved | Not achieved yet |
| Côte d'Ivoire |  | Not achieved | Not achieved yet |
| Croatia |  | Not achieved | Not achieved yet |
| Cuba |  | Not achieved | Not achieved yet |
| Cyprus |  | Not achieved | Not achieved yet |
| Czechia |  | Not achieved | Not achieved yet |
| Democratic Republic of the Congo |  | Not achieved | Not achieved yet |
| Djibouti |  | Not achieved | Not achieved yet |
| Dominica |  | Not achieved | Not achieved yet |
| Dominican Republic |  | Not achieved | Not achieved yet |
| Ecuador |  | Not achieved | Not achieved yet |
| Egypt |  | Not achieved | Not achieved yet |
| El Salvador |  | Not achieved | Not achieved yet |
| Equatorial Guinea |  | Not achieved | Not achieved yet |
| Eritrea |  | Not achieved | Not achieved yet |
| Estonia |  | Not achieved | Not achieved yet |
| Ethiopia |  | Not achieved | Not achieved yet |
| Fiji |  | Not achieved | Not achieved yet |
| Gabon |  | Not achieved | Not achieved yet |
| Gambia |  | Not achieved | Not achieved yet |
| Georgia |  | Not achieved | Not achieved yet |
| Ghana |  | Not achieved | Not achieved yet |
| Greece |  | Not achieved | Not achieved yet |
| Grenada |  | Not achieved | Not achieved yet |
| Guinea |  | Not achieved | Not achieved yet |
| Guinea-Bissau |  | Not achieved | Not achieved yet |
| Guyana |  | Not achieved | Not achieved yet |
| Hungary |  | Not achieved | Not achieved yet |
| Indonesia |  | Not achieved | Not achieved yet |
| Iran (Islamic Republic of) |  | Not achieved | Not achieved yet |
| Iraq |  | Not achieved | Not achieved yet |
| Italy |  | Not achieved | Not achieved yet |
| Jamaica |  | Not achieved | Not achieved yet |
| Kazakhstan |  | Not achieved | Not achieved yet |
| Kenya |  | Not achieved | Not achieved yet |
| Kiribati |  | Not achieved | Not achieved yet |
| Kuwait |  | Achieved | Achieved |
| Kyrgyzstan |  | Not achieved | Not achieved yet |
| Lao People's Democratic Republic |  | Not achieved | Not achieved yet |
| Latvia |  | Not achieved | Not achieved yet |
| Lebanon |  | Not achieved | Not achieved yet |
| Lesotho |  | Not achieved | Not achieved yet |
| Liberia |  | Not achieved | Not achieved yet |
| Libya |  | Not achieved | Not achieved yet |
| Lithuania |  | Not achieved | Not achieved yet |
| Luxembourg |  | Not achieved | Not achieved yet |
| Madagascar |  | Not achieved | Not achieved yet |
| Malaysia |  | Not achieved | Not achieved yet |
| Maldives |  | Not achieved | Not achieved yet |
| Mali |  | Not achieved | Not achieved yet |
| Malta |  | Not achieved | Not achieved yet |
| Mauritania |  | Achieved | Achieved |
| Micronesia (Federated States of) |  | Not achieved | Not achieved yet |
| Mongolia |  | Not achieved | Achieved |
| Montenegro |  | Not achieved | Not achieved yet |
| Morocco |  | Not achieved | Not achieved yet |
| Mozambique |  | Not achieved | Not achieved yet |
| Myanmar |  | Not achieved | Not achieved yet |
| Namibia |  | Not achieved | Not achieved yet |
| Nepal |  | Not achieved | Not achieved yet |
| New Zealand |  | Not achieved | Not achieved yet |
| Nicaragua |  | Not achieved | Not achieved yet |
| Niger |  | Not achieved | Achieved |
| Nigeria |  | Not achieved | Not achieved yet |
| Niue |  | Not achieved | Not achieved yet |
| North Macedonia |  | Achieved | Achieved |
| Oman |  | Not achieved | Not achieved yet |
| Pakistan |  | Not achieved | Not achieved yet |
| Panama |  | Not achieved | Not achieved yet |
| Papua New Guinea |  | Not achieved | Not achieved yet |
| Peru |  | Not achieved | Not achieved yet |
| Philippines |  | Not achieved | Not achieved yet |
| Poland |  | Not achieved | Not achieved yet |
| Portugal |  | Not achieved | Not achieved yet |
| Qatar |  | Achieved | Achieved |
| Republic of Korea |  | Achieved | Not achieved yet |
| Republic of Moldova |  | Not achieved | Not achieved yet |
| Romania |  | Not achieved | Not achieved yet |
| Russian Federation |  | Not achieved | Not achieved yet |
| Rwanda |  | Not achieved | Not achieved yet |
| Samoa |  | Not achieved | Not achieved yet |
| Sao Tome and Principe |  | Not achieved | Not achieved yet |
| Saudi Arabia |  | Not achieved | Not achieved yet |
| Senegal |  | Not achieved | Not achieved yet |
| Serbia |  | Achieved | Achieved |
| Seychelles |  | Not achieved | Not achieved yet |
| Sierra Leone |  | Not achieved | Not achieved yet |
| Singapore |  | Not achieved | Not achieved yet |
| Slovakia |  | Achieved | Achieved |
| Slovenia |  | Not achieved | Not achieved yet |
| Solomon Islands |  | Not achieved | Not achieved yet |
| Somalia |  | Not achieved | Not achieved yet |
| South Africa |  | Not achieved | Not achieved yet |
| South Sudan |  | Not achieved | Not achieved yet |
| Sri Lanka |  | Not achieved | Not achieved yet |
| Sudan |  | Not achieved | Not achieved yet |
| Suriname |  | Not achieved | Not achieved yet |
| Syrian Arab Republic |  | Achieved | Achieved |
| Tajikistan |  | Not achieved | Not achieved yet |
| Thailand |  | Not achieved | Not achieved yet |
| Timor-Leste |  | Not achieved | Not achieved yet |
| Togo |  | Not achieved | Not achieved yet |
| Tonga |  | Not achieved | Not achieved yet |
| Trinidad and Tobago |  | Not achieved | Not achieved yet |
| Tunisia |  | Not achieved | Achieved |
| Turkey |  | Not achieved | Achieved |
| Uganda |  | Not achieved | Not achieved yet |
| Ukraine |  | Not achieved | Not achieved yet |
| United Arab Emirates |  | Not achieved | Not achieved yet |
| United Republic of Tanzania |  | Not achieved | Not achieved yet |
| Uruguay |  | Not achieved | Not achieved yet |
| Uzbekistan |  | Not achieved | Not achieved yet |
| Vanuatu |  | Not achieved | Not achieved yet |
| Venezuela (Bolivarian Republic of) |  | Not achieved | Not achieved yet |
| Viet Nam |  | Not achieved | Not achieved yet |
| Yemen |  | Not achieved | Not achieved yet |
| Zambia |  | Not achieved | Not achieved yet |
| Zimbabwe |  | Not achieved | Not achieved yet |

Note: BRI countries: the Belt and Road countries.

The achievement of SDGs in 2021 was assessed based on the GBD estimate for 2021, and the future achievement by 2030 was the projected value from the health-related SDGs visualizations in GBD 2021 (https://vizhub.healthdata.org/sdg/).

| Supplementary Table 23. Achievement of SDGs for tuberculosis incidence rate in 149 BRI countries | | | |
| --- | --- | --- | --- |
| Country |  | 2021 | 2030 |
| Afghanistan |  | Not achieved | Not achieved yet |
| Albania |  | Not achieved | Not achieved yet |
| Algeria |  | Not achieved | Not achieved yet |
| Angola |  | Not achieved | Not achieved yet |
| Antigua and Barbuda |  | Not achieved | Not achieved yet |
| Argentina |  | Not achieved | Not achieved yet |
| Armenia |  | Not achieved | Not achieved yet |
| Austria |  | Not achieved | Not achieved yet |
| Azerbaijan |  | Not achieved | Not achieved yet |
| Bahrain |  | Not achieved | Not achieved yet |
| Bangladesh |  | Not achieved | Not achieved yet |
| Barbados |  | Not achieved | Not achieved yet |
| Belarus |  | Not achieved | Not achieved yet |
| Benin |  | Not achieved | Not achieved yet |
| Bolivia (Plurinational State of) |  | Not achieved | Not achieved yet |
| Bosnia and Herzegovina |  | Not achieved | Not achieved yet |
| Botswana |  | Not achieved | Not achieved yet |
| Brunei Darussalam |  | Not achieved | Not achieved yet |
| Bulgaria |  | Not achieved | Not achieved yet |
| Burkina Faso |  | Not achieved | Not achieved yet |
| Burundi |  | Not achieved | Not achieved yet |
| Cabo Verde |  | Not achieved | Not achieved yet |
| Cambodia |  | Not achieved | Not achieved yet |
| Cameroon |  | Not achieved | Not achieved yet |
| Central African Republic |  | Not achieved | Not achieved yet |
| Chad |  | Not achieved | Not achieved yet |
| Chile |  | Not achieved | Not achieved yet |
| China |  | Not achieved | Not achieved yet |
| Comoros |  | Not achieved | Not achieved yet |
| Congo |  | Not achieved | Not achieved yet |
| Cook Islands |  | Not achieved | Not achieved yet |
| Costa Rica |  | Not achieved | Not achieved yet |
| Côte d'Ivoire |  | Not achieved | Not achieved yet |
| Croatia |  | Not achieved | Not achieved yet |
| Cuba |  | Not achieved | Not achieved yet |
| Cyprus |  | Not achieved | Not achieved yet |
| Czechia |  | Not achieved | Not achieved yet |
| Democratic Republic of the Congo |  | Not achieved | Not achieved yet |
| Djibouti |  | Not achieved | Not achieved yet |
| Dominica |  | Not achieved | Not achieved yet |
| Dominican Republic |  | Not achieved | Not achieved yet |
| Ecuador |  | Not achieved | Not achieved yet |
| Egypt |  | Not achieved | Not achieved yet |
| El Salvador |  | Not achieved | Not achieved yet |
| Equatorial Guinea |  | Not achieved | Not achieved yet |
| Eritrea |  | Not achieved | Not achieved yet |
| Estonia |  | Not achieved | Not achieved yet |
| Ethiopia |  | Not achieved | Not achieved yet |
| Fiji |  | Not achieved | Not achieved yet |
| Gabon |  | Not achieved | Not achieved yet |
| Gambia |  | Not achieved | Not achieved yet |
| Georgia |  | Not achieved | Not achieved yet |
| Ghana |  | Not achieved | Not achieved yet |
| Greece |  | Not achieved | Not achieved yet |
| Grenada |  | Not achieved | Not achieved yet |
| Guinea |  | Not achieved | Not achieved yet |
| Guinea-Bissau |  | Not achieved | Not achieved yet |
| Guyana |  | Not achieved | Not achieved yet |
| Hungary |  | Not achieved | Not achieved yet |
| Indonesia |  | Not achieved | Not achieved yet |
| Iran (Islamic Republic of) |  | Not achieved | Not achieved yet |
| Iraq |  | Not achieved | Not achieved yet |
| Italy |  | Not achieved | Not achieved yet |
| Jamaica |  | Not achieved | Not achieved yet |
| Kazakhstan |  | Not achieved | Not achieved yet |
| Kenya |  | Not achieved | Not achieved yet |
| Kiribati |  | Not achieved | Not achieved yet |
| Kuwait |  | Not achieved | Not achieved yet |
| Kyrgyzstan |  | Not achieved | Not achieved yet |
| Lao People's Democratic Republic |  | Not achieved | Not achieved yet |
| Latvia |  | Not achieved | Not achieved yet |
| Lebanon |  | Not achieved | Not achieved yet |
| Lesotho |  | Not achieved | Not achieved yet |
| Liberia |  | Not achieved | Not achieved yet |
| Libya |  | Not achieved | Not achieved yet |
| Lithuania |  | Not achieved | Not achieved yet |
| Luxembourg |  | Not achieved | Not achieved yet |
| Madagascar |  | Not achieved | Not achieved yet |
| Malaysia |  | Not achieved | Not achieved yet |
| Maldives |  | Not achieved | Not achieved yet |
| Mali |  | Not achieved | Not achieved yet |
| Malta |  | Not achieved | Not achieved yet |
| Mauritania |  | Not achieved | Not achieved yet |
| Micronesia (Federated States of) |  | Not achieved | Not achieved yet |
| Mongolia |  | Not achieved | Not achieved yet |
| Montenegro |  | Not achieved | Not achieved yet |
| Morocco |  | Not achieved | Not achieved yet |
| Mozambique |  | Not achieved | Not achieved yet |
| Myanmar |  | Not achieved | Not achieved yet |
| Namibia |  | Not achieved | Not achieved yet |
| Nepal |  | Not achieved | Not achieved yet |
| New Zealand |  | Not achieved | Not achieved yet |
| Nicaragua |  | Not achieved | Not achieved yet |
| Niger |  | Not achieved | Not achieved yet |
| Nigeria |  | Not achieved | Not achieved yet |
| Niue |  | Not achieved | Not achieved yet |
| North Macedonia |  | Not achieved | Not achieved yet |
| Oman |  | Not achieved | Not achieved yet |
| Pakistan |  | Not achieved | Not achieved yet |
| Panama |  | Not achieved | Not achieved yet |
| Papua New Guinea |  | Not achieved | Not achieved yet |
| Peru |  | Not achieved | Not achieved yet |
| Philippines |  | Not achieved | Not achieved yet |
| Poland |  | Not achieved | Not achieved yet |
| Portugal |  | Not achieved | Not achieved yet |
| Qatar |  | Not achieved | Not achieved yet |
| Republic of Korea |  | Not achieved | Not achieved yet |
| Republic of Moldova |  | Not achieved | Not achieved yet |
| Romania |  | Not achieved | Not achieved yet |
| Russian Federation |  | Not achieved | Not achieved yet |
| Rwanda |  | Not achieved | Not achieved yet |
| Samoa |  | Not achieved | Not achieved yet |
| Sao Tome and Principe |  | Not achieved | Not achieved yet |
| Saudi Arabia |  | Not achieved | Not achieved yet |
| Senegal |  | Not achieved | Not achieved yet |
| Serbia |  | Not achieved | Not achieved yet |
| Seychelles |  | Not achieved | Not achieved yet |
| Sierra Leone |  | Not achieved | Not achieved yet |
| Singapore |  | Not achieved | Not achieved yet |
| Slovakia |  | Not achieved | Not achieved yet |
| Slovenia |  | Not achieved | Not achieved yet |
| Solomon Islands |  | Not achieved | Not achieved yet |
| Somalia |  | Not achieved | Not achieved yet |
| South Africa |  | Not achieved | Not achieved yet |
| South Sudan |  | Not achieved | Not achieved yet |
| Sri Lanka |  | Not achieved | Not achieved yet |
| Sudan |  | Not achieved | Not achieved yet |
| Suriname |  | Not achieved | Not achieved yet |
| Syrian Arab Republic |  | Not achieved | Not achieved yet |
| Tajikistan |  | Not achieved | Not achieved yet |
| Thailand |  | Not achieved | Not achieved yet |
| Timor-Leste |  | Not achieved | Not achieved yet |
| Togo |  | Not achieved | Not achieved yet |
| Tonga |  | Not achieved | Not achieved yet |
| Trinidad and Tobago |  | Not achieved | Not achieved yet |
| Tunisia |  | Not achieved | Not achieved yet |
| Turkey |  | Not achieved | Not achieved yet |
| Uganda |  | Not achieved | Not achieved yet |
| Ukraine |  | Not achieved | Not achieved yet |
| United Arab Emirates |  | Not achieved | Not achieved yet |
| United Republic of Tanzania |  | Not achieved | Not achieved yet |
| Uruguay |  | Not achieved | Not achieved yet |
| Uzbekistan |  | Not achieved | Not achieved yet |
| Vanuatu |  | Not achieved | Not achieved yet |
| Venezuela (Bolivarian Republic of) |  | Not achieved | Not achieved yet |
| Viet Nam |  | Not achieved | Not achieved yet |
| Yemen |  | Not achieved | Not achieved yet |
| Zambia |  | Not achieved | Not achieved yet |
| Zimbabwe |  | Not achieved | Not achieved yet |

Note: BRI countries: the Belt and Road countries.

The achievement of SDGs in 2021 was assessed based on the GBD estimate for 2021, and the future achievement by 2030 was the projected value from the health-related SDGs visualizations in GBD 2021 (https://vizhub.healthdata.org/sdg/).

| Supplementary Table 24. Achievement of SDGs for malaria incidence rate in 149 BRI countries | | | |
| --- | --- | --- | --- |
| Country |  | 2021 | 2030 |
| Afghanistan |  | Not achieved | Not achieved yet |
| Albania |  | Achieved | Achieved |
| Algeria |  | Achieved | Achieved |
| Angola |  | Not achieved | Not achieved yet |
| Antigua and Barbuda |  | Achieved | Achieved |
| Argentina |  | Achieved | Achieved |
| Armenia |  | Achieved | Achieved |
| Austria |  | Achieved | Achieved |
| Azerbaijan |  | Achieved | Achieved |
| Bahrain |  | Achieved | Achieved |
| Bangladesh |  | Not achieved | Not achieved yet |
| Barbados |  | Achieved | Achieved |
| Belarus |  | Achieved | Achieved |
| Benin |  | Not achieved | Not achieved yet |
| Bolivia (Plurinational State of) |  | Not achieved | Not achieved yet |
| Bosnia and Herzegovina |  | Achieved | Achieved |
| Botswana |  | Not achieved | Not achieved yet |
| Brunei Darussalam |  | Achieved | Achieved |
| Bulgaria |  | Achieved | Achieved |
| Burkina Faso |  | Not achieved | Not achieved yet |
| Burundi |  | Not achieved | Not achieved yet |
| Cabo Verde |  | Not achieved | Not achieved yet |
| Cambodia |  | Not achieved | Not achieved yet |
| Cameroon |  | Not achieved | Not achieved yet |
| Central African Republic |  | Not achieved | Not achieved yet |
| Chad |  | Not achieved | Not achieved yet |
| Chile |  | Achieved | Achieved |
| China |  | Achieved | Achieved |
| Comoros |  | Not achieved | Not achieved yet |
| Congo |  | Not achieved | Not achieved yet |
| Cook Islands |  | Achieved | Achieved |
| Costa Rica |  | Not achieved | Achieved |
| Côte d'Ivoire |  | Not achieved | Not achieved yet |
| Croatia |  | Achieved | Achieved |
| Cuba |  | Achieved | Achieved |
| Cyprus |  | Achieved | Achieved |
| Czechia |  | Achieved | Achieved |
| Democratic Republic of the Congo |  | Not achieved | Not achieved yet |
| Djibouti |  | Not achieved | Not achieved yet |
| Dominica |  | Achieved | Achieved |
| Dominican Republic |  | Not achieved | Not achieved yet |
| Ecuador |  | Not achieved | Not achieved yet |
| Egypt |  | Achieved | Achieved |
| El Salvador |  | Achieved | Achieved |
| Equatorial Guinea |  | Not achieved | Not achieved yet |
| Eritrea |  | Not achieved | Not achieved yet |
| Estonia |  | Achieved | Achieved |
| Ethiopia |  | Not achieved | Not achieved yet |
| Fiji |  | Achieved | Achieved |
| Gabon |  | Not achieved | Not achieved yet |
| Gambia |  | Not achieved | Not achieved yet |
| Georgia |  | Achieved | Achieved |
| Ghana |  | Not achieved | Not achieved yet |
| Greece |  | Achieved | Achieved |
| Grenada |  | Achieved | Achieved |
| Guinea |  | Not achieved | Not achieved yet |
| Guinea-Bissau |  | Not achieved | Not achieved yet |
| Guyana |  | Not achieved | Not achieved yet |
| Hungary |  | Achieved | Achieved |
| Indonesia |  | Not achieved | Not achieved yet |
| Iran (Islamic Republic of) |  | Not achieved | Achieved |
| Iraq |  | Achieved | Achieved |
| Italy |  | Achieved | Achieved |
| Jamaica |  | Achieved | Achieved |
| Kazakhstan |  | Achieved | Achieved |
| Kenya |  | Not achieved | Not achieved yet |
| Kiribati |  | Achieved | Achieved |
| Kuwait |  | Achieved | Achieved |
| Kyrgyzstan |  | Achieved | Achieved |
| Lao People's Democratic Republic |  | Not achieved | Not achieved yet |
| Latvia |  | Achieved | Achieved |
| Lebanon |  | Achieved | Achieved |
| Lesotho |  | Achieved | Achieved |
| Liberia |  | Not achieved | Not achieved yet |
| Libya |  | Achieved | Achieved |
| Lithuania |  | Achieved | Achieved |
| Luxembourg |  | Achieved | Achieved |
| Madagascar |  | Not achieved | Not achieved yet |
| Malaysia |  | Achieved | Achieved |
| Maldives |  | Achieved | Achieved |
| Mali |  | Not achieved | Not achieved yet |
| Malta |  | Achieved | Achieved |
| Mauritania |  | Not achieved | Not achieved yet |
| Micronesia (Federated States of) |  | Achieved | Achieved |
| Mongolia |  | Achieved | Achieved |
| Montenegro |  | Achieved | Achieved |
| Morocco |  | Achieved | Achieved |
| Mozambique |  | Not achieved | Not achieved yet |
| Myanmar |  | Not achieved | Not achieved yet |
| Namibia |  | Not achieved | Not achieved yet |
| Nepal |  | Not achieved | Not achieved yet |
| New Zealand |  | Achieved | Achieved |
| Nicaragua |  | Not achieved | Not achieved yet |
| Niger |  | Not achieved | Not achieved yet |
| Nigeria |  | Not achieved | Not achieved yet |
| Niue |  | Achieved | Achieved |
| North Macedonia |  | Achieved | Achieved |
| Oman |  | Not achieved | Achieved |
| Pakistan |  | Not achieved | Not achieved yet |
| Panama |  | Not achieved | Not achieved yet |
| Papua New Guinea |  | Not achieved | Not achieved yet |
| Peru |  | Not achieved | Not achieved yet |
| Philippines |  | Not achieved | Not achieved yet |
| Poland |  | Achieved | Achieved |
| Portugal |  | Achieved | Achieved |
| Qatar |  | Achieved | Achieved |
| Republic of Korea |  | Not achieved | Achieved |
| Republic of Moldova |  | Achieved | Achieved |
| Romania |  | Achieved | Achieved |
| Russian Federation |  | Achieved | Achieved |
| Rwanda |  | Not achieved | Not achieved yet |
| Samoa |  | Achieved | Achieved |
| Sao Tome and Principe |  | Not achieved | Not achieved yet |
| Saudi Arabia |  | Not achieved | Achieved |
| Senegal |  | Not achieved | Not achieved yet |
| Serbia |  | Achieved | Achieved |
| Seychelles |  | Achieved | Achieved |
| Sierra Leone |  | Not achieved | Not achieved yet |
| Singapore |  | Achieved | Achieved |
| Slovakia |  | Achieved | Achieved |
| Slovenia |  | Achieved | Achieved |
| Solomon Islands |  | Not achieved | Not achieved yet |
| Somalia |  | Not achieved | Not achieved yet |
| South Africa |  | Not achieved | Not achieved yet |
| South Sudan |  | Not achieved | Not achieved yet |
| Sri Lanka |  | Achieved | Achieved |
| Sudan |  | Not achieved | Not achieved yet |
| Suriname |  | Not achieved | Not achieved yet |
| Syrian Arab Republic |  | Achieved | Achieved |
| Tajikistan |  | Achieved | Achieved |
| Thailand |  | Not achieved | Not achieved yet |
| Timor-Leste |  | Achieved | Achieved |
| Togo |  | Not achieved | Not achieved yet |
| Tonga |  | Achieved | Achieved |
| Trinidad and Tobago |  | Achieved | Achieved |
| Tunisia |  | Achieved | Achieved |
| Turkey |  | Achieved | Achieved |
| Uganda |  | Not achieved | Not achieved yet |
| Ukraine |  | Achieved | Achieved |
| United Arab Emirates |  | Achieved | Achieved |
| United Republic of Tanzania |  | Not achieved | Not achieved yet |
| Uruguay |  | Achieved | Achieved |
| Uzbekistan |  | Achieved | Achieved |
| Vanuatu |  | Not achieved | Not achieved yet |
| Venezuela (Bolivarian Republic of) |  | Not achieved | Not achieved yet |
| Viet Nam |  | Not achieved | Achieved |
| Yemen |  | Not achieved | Not achieved yet |
| Zambia |  | Not achieved | Not achieved yet |
| Zimbabwe |  | Not achieved | Not achieved yet |

Note: BRI countries: the Belt and Road countries.

The achievement of SDGs in 2021 was assessed based on the GBD estimate for 2021, and the future achievement by 2030 was the projected value from the health-related SDGs visualizations in GBD 2021 (<https://vizhub.healthdata.org/sdg/>).

| Supplementary Table 25. Achievement of SDGs for NTDs prevalence rate in 149 BRI countries | | | |
| --- | --- | --- | --- |
| Country |  | 2021 | 2030 |
| Afghanistan |  | Not achieved | Not achieved yet |
| Albania |  | Achieved | Achieved |
| Algeria |  | Not achieved | Not achieved yet |
| Angola |  | Not achieved | Not achieved yet |
| Antigua and Barbuda |  | Not achieved | Not achieved yet |
| Argentina |  | Not achieved | Not achieved yet |
| Armenia |  | Not achieved | Not achieved yet |
| Austria |  | Achieved | Achieved |
| Azerbaijan |  | Not achieved | Not achieved yet |
| Bahrain |  | Not achieved | Not achieved yet |
| Bangladesh |  | Not achieved | Not achieved yet |
| Barbados |  | Not achieved | Not achieved yet |
| Belarus |  | Achieved | Achieved |
| Benin |  | Not achieved | Not achieved yet |
| Bolivia (Plurinational State of) |  | Not achieved | Not achieved yet |
| Bosnia and Herzegovina |  | Achieved | Achieved |
| Botswana |  | Not achieved | Not achieved yet |
| Brunei Darussalam |  | Not achieved | Not achieved yet |
| Bulgaria |  | Achieved | Achieved |
| Burkina Faso |  | Not achieved | Not achieved yet |
| Burundi |  | Not achieved | Not achieved yet |
| Cabo Verde |  | Not achieved | Not achieved yet |
| Cambodia |  | Not achieved | Not achieved yet |
| Cameroon |  | Not achieved | Not achieved yet |
| Central African Republic |  | Not achieved | Not achieved yet |
| Chad |  | Not achieved | Not achieved yet |
| Chile |  | Not achieved | Not achieved yet |
| China |  | Not achieved | Not achieved yet |
| Comoros |  | Not achieved | Not achieved yet |
| Congo |  | Not achieved | Not achieved yet |
| Cook Islands |  | Not achieved | Not achieved yet |
| Costa Rica |  | Not achieved | Not achieved yet |
| Côte d'Ivoire |  | Not achieved | Not achieved yet |
| Croatia |  | Achieved | Achieved |
| Cuba |  | Not achieved | Not achieved yet |
| Cyprus |  | Achieved | Achieved |
| Czechia |  | Achieved | Achieved |
| Democratic Republic of the Congo |  | Not achieved | Not achieved yet |
| Djibouti |  | Not achieved | Not achieved yet |
| Dominica |  | Not achieved | Not achieved yet |
| Dominican Republic |  | Not achieved | Not achieved yet |
| Ecuador |  | Not achieved | Not achieved yet |
| Egypt |  | Not achieved | Not achieved yet |
| El Salvador |  | Not achieved | Not achieved yet |
| Equatorial Guinea |  | Not achieved | Not achieved yet |
| Eritrea |  | Not achieved | Not achieved yet |
| Estonia |  | Achieved | Achieved |
| Ethiopia |  | Not achieved | Not achieved yet |
| Fiji |  | Not achieved | Not achieved yet |
| Gabon |  | Not achieved | Not achieved yet |
| Gambia |  | Not achieved | Not achieved yet |
| Georgia |  | Not achieved | Not achieved yet |
| Ghana |  | Not achieved | Not achieved yet |
| Greece |  | Achieved | Achieved |
| Grenada |  | Not achieved | Not achieved yet |
| Guinea |  | Not achieved | Not achieved yet |
| Guinea-Bissau |  | Not achieved | Not achieved yet |
| Guyana |  | Not achieved | Not achieved yet |
| Hungary |  | Achieved | Achieved |
| Indonesia |  | Not achieved | Not achieved yet |
| Iran (Islamic Republic of) |  | Not achieved | Not achieved yet |
| Iraq |  | Not achieved | Not achieved yet |
| Italy |  | Achieved | Achieved |
| Jamaica |  | Not achieved | Not achieved yet |
| Kazakhstan |  | Not achieved | Not achieved yet |
| Kenya |  | Not achieved | Not achieved yet |
| Kiribati |  | Not achieved | Not achieved yet |
| Kuwait |  | Achieved | Achieved |
| Kyrgyzstan |  | Not achieved | Not achieved yet |
| Lao People's Democratic Republic |  | Not achieved | Not achieved yet |
| Latvia |  | Achieved | Achieved |
| Lebanon |  | Not achieved | Not achieved yet |
| Lesotho |  | Not achieved | Not achieved yet |
| Liberia |  | Not achieved | Not achieved yet |
| Libya |  | Not achieved | Not achieved yet |
| Lithuania |  | Achieved | Achieved |
| Luxembourg |  | Achieved | Achieved |
| Madagascar |  | Not achieved | Not achieved yet |
| Malaysia |  | Not achieved | Not achieved yet |
| Maldives |  | Not achieved | Not achieved yet |
| Mali |  | Not achieved | Not achieved yet |
| Malta |  | Achieved | Achieved |
| Mauritania |  | Not achieved | Not achieved yet |
| Micronesia (Federated States of) |  | Not achieved | Not achieved yet |
| Mongolia |  | Not achieved | Not achieved yet |
| Montenegro |  | Achieved | Achieved |
| Morocco |  | Not achieved | Not achieved yet |
| Mozambique |  | Not achieved | Not achieved yet |
| Myanmar |  | Not achieved | Not achieved yet |
| Namibia |  | Not achieved | Not achieved yet |
| Nepal |  | Not achieved | Not achieved yet |
| New Zealand |  | Achieved | Achieved |
| Nicaragua |  | Not achieved | Not achieved yet |
| Niger |  | Not achieved | Not achieved yet |
| Nigeria |  | Not achieved | Not achieved yet |
| Niue |  | Not achieved | Not achieved yet |
| North Macedonia |  | Achieved | Achieved |
| Oman |  | Not achieved | Not achieved yet |
| Pakistan |  | Not achieved | Not achieved yet |
| Panama |  | Not achieved | Not achieved yet |
| Papua New Guinea |  | Not achieved | Not achieved yet |
| Peru |  | Not achieved | Not achieved yet |
| Philippines |  | Not achieved | Not achieved yet |
| Poland |  | Achieved | Achieved |
| Portugal |  | Not achieved | Not achieved yet |
| Qatar |  | Not achieved | Not achieved yet |
| Republic of Korea |  | Not achieved | Not achieved yet |
| Republic of Moldova |  | Achieved | Achieved |
| Romania |  | Achieved | Achieved |
| Russian Federation |  | Not achieved | Not achieved yet |
| Rwanda |  | Not achieved | Not achieved yet |
| Samoa |  | Not achieved | Not achieved yet |
| Sao Tome and Principe |  | Not achieved | Not achieved yet |
| Saudi Arabia |  | Not achieved | Not achieved yet |
| Senegal |  | Not achieved | Not achieved yet |
| Serbia |  | Achieved | Achieved |
| Seychelles |  | Not achieved | Not achieved yet |
| Sierra Leone |  | Not achieved | Not achieved yet |
| Singapore |  | Not achieved | Achieved |
| Slovakia |  | Achieved | Achieved |
| Slovenia |  | Achieved | Achieved |
| Solomon Islands |  | Not achieved | Not achieved yet |
| Somalia |  | Not achieved | Not achieved yet |
| South Africa |  | Not achieved | Not achieved yet |
| South Sudan |  | Not achieved | Not achieved yet |
| Sri Lanka |  | Not achieved | Not achieved yet |
| Sudan |  | Not achieved | Not achieved yet |
| Suriname |  | Not achieved | Not achieved yet |
| Syrian Arab Republic |  | Not achieved | Not achieved yet |
| Tajikistan |  | Not achieved | Not achieved yet |
| Thailand |  | Not achieved | Not achieved yet |
| Timor-Leste |  | Not achieved | Not achieved yet |
| Togo |  | Not achieved | Not achieved yet |
| Tonga |  | Not achieved | Not achieved yet |
| Trinidad and Tobago |  | Not achieved | Not achieved yet |
| Tunisia |  | Not achieved | Not achieved yet |
| Turkey |  | Not achieved | Not achieved yet |
| Uganda |  | Not achieved | Not achieved yet |
| Ukraine |  | Not achieved | Not achieved yet |
| United Arab Emirates |  | Not achieved | Not achieved yet |
| United Republic of Tanzania |  | Not achieved | Not achieved yet |
| Uruguay |  | Not achieved | Achieved |
| Uzbekistan |  | Not achieved | Not achieved yet |
| Vanuatu |  | Not achieved | Not achieved yet |
| Venezuela (Bolivarian Republic of) |  | Not achieved | Not achieved yet |
| Viet Nam |  | Not achieved | Not achieved yet |
| Yemen |  | Not achieved | Not achieved yet |
| Zambia |  | Not achieved | Not achieved yet |
| Zimbabwe |  | Not achieved | Not achieved yet |

Note: BRI countries: the Belt and Road countries.

The achievement of SDGs in 2021 was assessed based on the GBD estimate for 2021, and the future achievement by 2030 was the projected value from the health-related SDGs visualizations in GBD 2021 (https://vizhub.healthdata.org/sdg/).

| Supplementary Table 26. Achievement of SDGs for NCD mortality rate in 149 BRI countries | | | |
| --- | --- | --- | --- |
| Country |  | 2021 | 2030 |
| Afghanistan |  | Not achieved | Not achieved yet |
| Albania |  | Not achieved | Not achieved yet |
| Algeria |  | Not achieved | Not achieved yet |
| Angola |  | Not achieved | Not achieved yet |
| Antigua and Barbuda |  | Not achieved | Not achieved yet |
| Argentina |  | Not achieved | Not achieved yet |
| Armenia |  | Not achieved | Not achieved yet |
| Austria |  | Not achieved | Achieved |
| Azerbaijan |  | Not achieved | Achieved |
| Bahrain |  | Not achieved | Not achieved yet |
| Bangladesh |  | Not achieved | Not achieved yet |
| Barbados |  | Not achieved | Not achieved yet |
| Belarus |  | Not achieved | Not achieved yet |
| Benin |  | Not achieved | Not achieved yet |
| Bolivia (Plurinational State of) |  | Not achieved | Not achieved yet |
| Bosnia and Herzegovina |  | Not achieved | Not achieved yet |
| Botswana |  | Not achieved | Achieved |
| Brunei Darussalam |  | Not achieved | Not achieved yet |
| Bulgaria |  | Not achieved | Not achieved yet |
| Burkina Faso |  | Not achieved | Not achieved yet |
| Burundi |  | Not achieved | Not achieved yet |
| Cabo Verde |  | Not achieved | Not achieved yet |
| Cambodia |  | Not achieved | Not achieved yet |
| Cameroon |  | Not achieved | Not achieved yet |
| Central African Republic |  | Not achieved | Not achieved yet |
| Chad |  | Not achieved | Not achieved yet |
| Chile |  | Not achieved | Not achieved yet |
| China |  | Not achieved | Not achieved yet |
| Comoros |  | Not achieved | Not achieved yet |
| Congo |  | Not achieved | Not achieved yet |
| Cook Islands |  | Not achieved | Not achieved yet |
| Costa Rica |  | Not achieved | Not achieved yet |
| Côte d'Ivoire |  | Not achieved | Not achieved yet |
| Croatia |  | Not achieved | Not achieved yet |
| Cuba |  | Not achieved | Not achieved yet |
| Cyprus |  | Not achieved | Not achieved yet |
| Czechia |  | Not achieved | Not achieved yet |
| Democratic Republic of the Congo |  | Not achieved | Not achieved yet |
| Djibouti |  | Not achieved | Not achieved yet |
| Dominica |  | Not achieved | Not achieved yet |
| Dominican Republic |  | Not achieved | Not achieved yet |
| Ecuador |  | Not achieved | Not achieved yet |
| Egypt |  | Not achieved | Not achieved yet |
| El Salvador |  | Not achieved | Not achieved yet |
| Equatorial Guinea |  | Not achieved | Not achieved yet |
| Eritrea |  | Not achieved | Not achieved yet |
| Estonia |  | Not achieved | Achieved |
| Ethiopia |  | Not achieved | Not achieved yet |
| Fiji |  | Not achieved | Not achieved yet |
| Gabon |  | Not achieved | Not achieved yet |
| Gambia |  | Not achieved | Not achieved yet |
| Georgia |  | Not achieved | Not achieved yet |
| Ghana |  | Not achieved | Not achieved yet |
| Greece |  | Not achieved | Not achieved yet |
| Grenada |  | Not achieved | Not achieved yet |
| Guinea |  | Not achieved | Not achieved yet |
| Guinea-Bissau |  | Not achieved | Not achieved yet |
| Guyana |  | Not achieved | Not achieved yet |
| Hungary |  | Not achieved | Not achieved yet |
| Indonesia |  | Not achieved | Not achieved yet |
| Iran (Islamic Republic of) |  | Not achieved | Not achieved yet |
| Iraq |  | Not achieved | Not achieved yet |
| Italy |  | Not achieved | Not achieved yet |
| Jamaica |  | Not achieved | Not achieved yet |
| Kazakhstan |  | Not achieved | Achieved |
| Kenya |  | Not achieved | Not achieved yet |
| Kiribati |  | Not achieved | Not achieved yet |
| Kuwait |  | Not achieved | Not achieved yet |
| Kyrgyzstan |  | Not achieved | Achieved |
| Lao People's Democratic Republic |  | Not achieved | Not achieved yet |
| Latvia |  | Not achieved | Not achieved yet |
| Lebanon |  | Not achieved | Not achieved yet |
| Lesotho |  | Not achieved | Not achieved yet |
| Liberia |  | Not achieved | Not achieved yet |
| Libya |  | Not achieved | Not achieved yet |
| Lithuania |  | Not achieved | Not achieved yet |
| Luxembourg |  | Not achieved | Achieved |
| Madagascar |  | Not achieved | Not achieved yet |
| Malaysia |  | Not achieved | Not achieved yet |
| Maldives |  | Not achieved | Achieved |
| Mali |  | Not achieved | Not achieved yet |
| Malta |  | Not achieved | Not achieved yet |
| Mauritania |  | Not achieved | Not achieved yet |
| Micronesia (Federated States of) |  | Not achieved | Not achieved yet |
| Mongolia |  | Not achieved | Not achieved yet |
| Montenegro |  | Not achieved | Not achieved yet |
| Morocco |  | Not achieved | Not achieved yet |
| Mozambique |  | Not achieved | Not achieved yet |
| Myanmar |  | Not achieved | Not achieved yet |
| Namibia |  | Not achieved | Not achieved yet |
| Nepal |  | Not achieved | Not achieved yet |
| New Zealand |  | Not achieved | Not achieved yet |
| Nicaragua |  | Not achieved | Not achieved yet |
| Niger |  | Not achieved | Not achieved yet |
| Nigeria |  | Not achieved | Not achieved yet |
| Niue |  | Not achieved | Not achieved yet |
| North Macedonia |  | Not achieved | Not achieved yet |
| Oman |  | Not achieved | Achieved |
| Pakistan |  | Not achieved | Not achieved yet |
| Panama |  | Not achieved | Not achieved yet |
| Papua New Guinea |  | Not achieved | Not achieved yet |
| Peru |  | Not achieved | Not achieved yet |
| Philippines |  | Not achieved | Not achieved yet |
| Poland |  | Not achieved | Not achieved yet |
| Portugal |  | Not achieved | Not achieved yet |
| Qatar |  | Not achieved | Achieved |
| Republic of Korea |  | Not achieved | Not achieved yet |
| Republic of Moldova |  | Not achieved | Not achieved yet |
| Romania |  | Not achieved | Not achieved yet |
| Russian Federation |  | Not achieved | Not achieved yet |
| Rwanda |  | Not achieved | Not achieved yet |
| Samoa |  | Not achieved | Not achieved yet |
| Sao Tome and Principe |  | Not achieved | Not achieved yet |
| Saudi Arabia |  | Not achieved | Not achieved yet |
| Senegal |  | Not achieved | Not achieved yet |
| Serbia |  | Not achieved | Not achieved yet |
| Seychelles |  | Not achieved | Not achieved yet |
| Sierra Leone |  | Not achieved | Not achieved yet |
| Singapore |  | Not achieved | Achieved |
| Slovakia |  | Not achieved | Not achieved yet |
| Slovenia |  | Not achieved | Achieved |
| Solomon Islands |  | Not achieved | Not achieved yet |
| Somalia |  | Not achieved | Not achieved yet |
| South Africa |  | Not achieved | Not achieved yet |
| South Sudan |  | Not achieved | Not achieved yet |
| Sri Lanka |  | Not achieved | Not achieved yet |
| Sudan |  | Not achieved | Not achieved yet |
| Suriname |  | Not achieved | Not achieved yet |
| Syrian Arab Republic |  | Not achieved | Not achieved yet |
| Tajikistan |  | Not achieved | Not achieved yet |
| Thailand |  | Not achieved | Not achieved yet |
| Timor-Leste |  | Not achieved | Not achieved yet |
| Togo |  | Not achieved | Not achieved yet |
| Tonga |  | Not achieved | Not achieved yet |
| Trinidad and Tobago |  | Not achieved | Not achieved yet |
| Tunisia |  | Not achieved | Not achieved yet |
| Turkey |  | Not achieved | Not achieved yet |
| Uganda |  | Not achieved | Not achieved yet |
| Ukraine |  | Not achieved | Not achieved yet |
| United Arab Emirates |  | Achieved | Achieved |
| United Republic of Tanzania |  | Not achieved | Not achieved yet |
| Uruguay |  | Not achieved | Not achieved yet |
| Uzbekistan |  | Not achieved | Not achieved yet |
| Vanuatu |  | Not achieved | Not achieved yet |
| Venezuela (Bolivarian Republic of) |  | Not achieved | Not achieved yet |
| Viet Nam |  | Not achieved | Not achieved yet |
| Yemen |  | Not achieved | Not achieved yet |
| Zambia |  | Not achieved | Not achieved yet |
| Zimbabwe |  | Not achieved | Not achieved yet |

Note: NCD: Non-communicable diseases; BRI countries: the Belt and Road countries.

The achievement of SDGs in 2021 was assessed based on the GBD estimate for 2021, while the future achievement by 2030 was projected based on the weighted mean annual rate of change.

| Supplementary Table 27. Achievement of SDGs for suicide mortality rate in 149 BRI countries | | | |
| --- | --- | --- | --- |
| Country |  | 2021 | 2030 |
| Afghanistan |  | Not achieved | Not achieved yet |
| Albania |  | Not achieved | Not achieved yet |
| Algeria |  | Not achieved | Not achieved yet |
| Angola |  | Not achieved | Not achieved yet |
| Antigua and Barbuda |  | Not achieved | Not achieved yet |
| Argentina |  | Not achieved | Not achieved yet |
| Armenia |  | Not achieved | Achieved |
| Austria |  | Not achieved | Not achieved yet |
| Azerbaijan |  | Not achieved | Not achieved yet |
| Bahrain |  | Not achieved | Not achieved yet |
| Bangladesh |  | Not achieved | Not achieved yet |
| Barbados |  | Not achieved | Not achieved yet |
| Belarus |  | Not achieved | Not achieved yet |
| Benin |  | Not achieved | Not achieved yet |
| Bolivia (Plurinational State of) |  | Not achieved | Not achieved yet |
| Bosnia and Herzegovina |  | Not achieved | Not achieved yet |
| Botswana |  | Not achieved | Not achieved yet |
| Brunei Darussalam |  | Not achieved | Not achieved yet |
| Bulgaria |  | Not achieved | Not achieved yet |
| Burkina Faso |  | Not achieved | Not achieved yet |
| Burundi |  | Not achieved | Not achieved yet |
| Cabo Verde |  | Not achieved | Not achieved yet |
| Cambodia |  | Not achieved | Not achieved yet |
| Cameroon |  | Not achieved | Not achieved yet |
| Central African Republic |  | Not achieved | Not achieved yet |
| Chad |  | Not achieved | Not achieved yet |
| Chile |  | Not achieved | Not achieved yet |
| China |  | Not achieved | Not achieved yet |
| Comoros |  | Not achieved | Not achieved yet |
| Congo |  | Not achieved | Not achieved yet |
| Cook Islands |  | Not achieved | Not achieved yet |
| Costa Rica |  | Not achieved | Not achieved yet |
| Côte d'Ivoire |  | Not achieved | Not achieved yet |
| Croatia |  | Not achieved | Not achieved yet |
| Cuba |  | Not achieved | Not achieved yet |
| Cyprus |  | Not achieved | Not achieved yet |
| Czechia |  | Not achieved | Not achieved yet |
| Democratic Republic of the Congo |  | Not achieved | Not achieved yet |
| Djibouti |  | Not achieved | Not achieved yet |
| Dominica |  | Not achieved | Not achieved yet |
| Dominican Republic |  | Not achieved | Not achieved yet |
| Ecuador |  | Not achieved | Not achieved yet |
| Egypt |  | Not achieved | Not achieved yet |
| El Salvador |  | Not achieved | Not achieved yet |
| Equatorial Guinea |  | Not achieved | Not achieved yet |
| Eritrea |  | Not achieved | Not achieved yet |
| Estonia |  | Not achieved | Not achieved yet |
| Ethiopia |  | Not achieved | Not achieved yet |
| Fiji |  | Not achieved | Not achieved yet |
| Gabon |  | Not achieved | Not achieved yet |
| Gambia |  | Not achieved | Not achieved yet |
| Georgia |  | Not achieved | Not achieved yet |
| Ghana |  | Not achieved | Not achieved yet |
| Greece |  | Not achieved | Not achieved yet |
| Grenada |  | Not achieved | Not achieved yet |
| Guinea |  | Not achieved | Not achieved yet |
| Guinea-Bissau |  | Not achieved | Not achieved yet |
| Guyana |  | Not achieved | Not achieved yet |
| Hungary |  | Not achieved | Not achieved yet |
| Indonesia |  | Not achieved | Not achieved yet |
| Iran (Islamic Republic of) |  | Not achieved | Not achieved yet |
| Iraq |  | Not achieved | Not achieved yet |
| Italy |  | Not achieved | Not achieved yet |
| Jamaica |  | Not achieved | Not achieved yet |
| Kazakhstan |  | Not achieved | Not achieved yet |
| Kenya |  | Not achieved | Not achieved yet |
| Kiribati |  | Not achieved | Not achieved yet |
| Kuwait |  | Not achieved | Not achieved yet |
| Kyrgyzstan |  | Not achieved | Not achieved yet |
| Lao People's Democratic Republic |  | Not achieved | Not achieved yet |
| Latvia |  | Not achieved | Not achieved yet |
| Lebanon |  | Not achieved | Not achieved yet |
| Lesotho |  | Not achieved | Not achieved yet |
| Liberia |  | Not achieved | Not achieved yet |
| Libya |  | Not achieved | Not achieved yet |
| Lithuania |  | Not achieved | Not achieved yet |
| Luxembourg |  | Not achieved | Not achieved yet |
| Madagascar |  | Not achieved | Not achieved yet |
| Malaysia |  | Not achieved | Not achieved yet |
| Maldives |  | Not achieved | Not achieved yet |
| Mali |  | Not achieved | Not achieved yet |
| Malta |  | Not achieved | Not achieved yet |
| Mauritania |  | Not achieved | Not achieved yet |
| Micronesia (Federated States of) |  | Not achieved | Not achieved yet |
| Mongolia |  | Not achieved | Not achieved yet |
| Montenegro |  | Not achieved | Not achieved yet |
| Morocco |  | Not achieved | Not achieved yet |
| Mozambique |  | Not achieved | Not achieved yet |
| Myanmar |  | Not achieved | Not achieved yet |
| Namibia |  | Not achieved | Not achieved yet |
| Nepal |  | Not achieved | Not achieved yet |
| New Zealand |  | Not achieved | Not achieved yet |
| Nicaragua |  | Not achieved | Not achieved yet |
| Niger |  | Not achieved | Not achieved yet |
| Nigeria |  | Not achieved | Not achieved yet |
| Niue |  | Not achieved | Not achieved yet |
| North Macedonia |  | Not achieved | Achieved |
| Oman |  | Not achieved | Not achieved yet |
| Pakistan |  | Not achieved | Not achieved yet |
| Panama |  | Not achieved | Not achieved yet |
| Papua New Guinea |  | Not achieved | Not achieved yet |
| Peru |  | Not achieved | Not achieved yet |
| Philippines |  | Not achieved | Not achieved yet |
| Poland |  | Not achieved | Not achieved yet |
| Portugal |  | Not achieved | Not achieved yet |
| Qatar |  | Not achieved | Achieved |
| Republic of Korea |  | Not achieved | Not achieved yet |
| Republic of Moldova |  | Not achieved | Not achieved yet |
| Romania |  | Not achieved | Not achieved yet |
| Russian Federation |  | Not achieved | Not achieved yet |
| Rwanda |  | Not achieved | Not achieved yet |
| Samoa |  | Not achieved | Not achieved yet |
| Sao Tome and Principe |  | Not achieved | Not achieved yet |
| Saudi Arabia |  | Not achieved | Not achieved yet |
| Senegal |  | Not achieved | Not achieved yet |
| Serbia |  | Not achieved | Not achieved yet |
| Seychelles |  | Not achieved | Not achieved yet |
| Sierra Leone |  | Not achieved | Not achieved yet |
| Singapore |  | Not achieved | Not achieved yet |
| Slovakia |  | Not achieved | Not achieved yet |
| Slovenia |  | Not achieved | Not achieved yet |
| Solomon Islands |  | Not achieved | Not achieved yet |
| Somalia |  | Not achieved | Not achieved yet |
| South Africa |  | Not achieved | Not achieved yet |
| South Sudan |  | Not achieved | Not achieved yet |
| Sri Lanka |  | Not achieved | Not achieved yet |
| Sudan |  | Not achieved | Not achieved yet |
| Suriname |  | Not achieved | Not achieved yet |
| Syrian Arab Republic |  | Not achieved | Not achieved yet |
| Tajikistan |  | Not achieved | Not achieved yet |
| Thailand |  | Not achieved | Not achieved yet |
| Timor-Leste |  | Not achieved | Not achieved yet |
| Togo |  | Not achieved | Not achieved yet |
| Tonga |  | Not achieved | Not achieved yet |
| Trinidad and Tobago |  | Not achieved | Not achieved yet |
| Tunisia |  | Not achieved | Not achieved yet |
| Turkey |  | Not achieved | Not achieved yet |
| Uganda |  | Not achieved | Not achieved yet |
| Ukraine |  | Not achieved | Not achieved yet |
| United Arab Emirates |  | Not achieved | Not achieved yet |
| United Republic of Tanzania |  | Not achieved | Not achieved yet |
| Uruguay |  | Not achieved | Not achieved yet |
| Uzbekistan |  | Not achieved | Not achieved yet |
| Vanuatu |  | Not achieved | Not achieved yet |
| Venezuela (Bolivarian Republic of) |  | Not achieved | Not achieved yet |
| Viet Nam |  | Not achieved | Not achieved yet |
| Yemen |  | Not achieved | Not achieved yet |
| Zambia |  | Not achieved | Not achieved yet |
| Zimbabwe |  | Not achieved | Not achieved yet |

Note: BRI countries: the Belt and Road countries.

The achievement of SDGs in 2021 was assessed based on the GBD estimate for 2021, and the future achievement by 2030 was the projected value from the GBD foresight visualization in GBD 2021 (https://vizhub.healthdata.org/gbd-foresight/).

| Supplementary Table 28. Achievement of SDGs for road traffic mortality rate in 149 BRI countries | | | |
| --- | --- | --- | --- |
| Country |  | 2021 | 2030 |
| Afghanistan |  | Not achieved | Not achieved yet |
| Albania |  | Not achieved | Not achieved yet |
| Algeria |  | Not achieved | Not achieved yet |
| Angola |  | Not achieved | Not achieved yet |
| Antigua and Barbuda |  | Not achieved | Not achieved yet |
| Argentina |  | Not achieved | Not achieved yet |
| Armenia |  | Not achieved | Not achieved yet |
| Austria |  | Not achieved | Not achieved yet |
| Azerbaijan |  | Not achieved | Not achieved yet |
| Bahrain |  | Not achieved | Not achieved yet |
| Bangladesh |  | Not achieved | Not achieved yet |
| Barbados |  | Not achieved | Not achieved yet |
| Belarus |  | Not achieved | Not achieved yet |
| Benin |  | Not achieved | Not achieved yet |
| Bolivia (Plurinational State of) |  | Not achieved | Not achieved yet |
| Bosnia and Herzegovina |  | Not achieved | Not achieved yet |
| Botswana |  | Not achieved | Not achieved yet |
| Brunei Darussalam |  | Not achieved | Not achieved yet |
| Bulgaria |  | Not achieved | Not achieved yet |
| Burkina Faso |  | Not achieved | Not achieved yet |
| Burundi |  | Not achieved | Not achieved yet |
| Cabo Verde |  | Not achieved | Not achieved yet |
| Cambodia |  | Not achieved | Not achieved yet |
| Cameroon |  | Not achieved | Not achieved yet |
| Central African Republic |  | Not achieved | Not achieved yet |
| Chad |  | Not achieved | Not achieved yet |
| Chile |  | Not achieved | Not achieved yet |
| China |  | Not achieved | Not achieved yet |
| Comoros |  | Not achieved | Not achieved yet |
| Congo |  | Not achieved | Not achieved yet |
| Cook Islands |  | Not achieved | Not achieved yet |
| Costa Rica |  | Not achieved | Not achieved yet |
| Côte d'Ivoire |  | Not achieved | Not achieved yet |
| Croatia |  | Not achieved | Not achieved yet |
| Cuba |  | Not achieved | Not achieved yet |
| Cyprus |  | Not achieved | Not achieved yet |
| Czechia |  | Not achieved | Not achieved yet |
| Democratic Republic of the Congo |  | Not achieved | Not achieved yet |
| Djibouti |  | Not achieved | Not achieved yet |
| Dominica |  | Not achieved | Not achieved yet |
| Dominican Republic |  | Not achieved | Not achieved yet |
| Ecuador |  | Not achieved | Not achieved yet |
| Egypt |  | Not achieved | Not achieved yet |
| El Salvador |  | Not achieved | Not achieved yet |
| Equatorial Guinea |  | Not achieved | Not achieved yet |
| Eritrea |  | Not achieved | Not achieved yet |
| Estonia |  | Not achieved | Not achieved yet |
| Ethiopia |  | Not achieved | Not achieved yet |
| Fiji |  | Not achieved | Not achieved yet |
| Gabon |  | Not achieved | Not achieved yet |
| Gambia |  | Not achieved | Not achieved yet |
| Georgia |  | Not achieved | Not achieved yet |
| Ghana |  | Not achieved | Not achieved yet |
| Greece |  | Not achieved | Not achieved yet |
| Grenada |  | Not achieved | Not achieved yet |
| Guinea |  | Not achieved | Not achieved yet |
| Guinea-Bissau |  | Not achieved | Not achieved yet |
| Guyana |  | Not achieved | Not achieved yet |
| Hungary |  | Not achieved | Not achieved yet |
| Indonesia |  | Not achieved | Not achieved yet |
| Iran (Islamic Republic of) |  | Not achieved | Not achieved yet |
| Iraq |  | Not achieved | Not achieved yet |
| Italy |  | Not achieved | Not achieved yet |
| Jamaica |  | Not achieved | Not achieved yet |
| Kazakhstan |  | Not achieved | Not achieved yet |
| Kenya |  | Not achieved | Not achieved yet |
| Kiribati |  | Not achieved | Not achieved yet |
| Kuwait |  | Not achieved | Not achieved yet |
| Kyrgyzstan |  | Not achieved | Not achieved yet |
| Lao People's Democratic Republic |  | Not achieved | Not achieved yet |
| Latvia |  | Not achieved | Not achieved yet |
| Lebanon |  | Not achieved | Not achieved yet |
| Lesotho |  | Not achieved | Not achieved yet |
| Liberia |  | Not achieved | Not achieved yet |
| Libya |  | Not achieved | Not achieved yet |
| Lithuania |  | Not achieved | Not achieved yet |
| Luxembourg |  | Not achieved | Not achieved yet |
| Madagascar |  | Not achieved | Not achieved yet |
| Malaysia |  | Not achieved | Not achieved yet |
| Maldives |  | Not achieved | Not achieved yet |
| Mali |  | Not achieved | Not achieved yet |
| Malta |  | Not achieved | Not achieved yet |
| Mauritania |  | Not achieved | Not achieved yet |
| Micronesia (Federated States of) |  | Not achieved | Not achieved yet |
| Mongolia |  | Not achieved | Not achieved yet |
| Montenegro |  | Not achieved | Not achieved yet |
| Morocco |  | Not achieved | Not achieved yet |
| Mozambique |  | Not achieved | Not achieved yet |
| Myanmar |  | Not achieved | Not achieved yet |
| Namibia |  | Not achieved | Not achieved yet |
| Nepal |  | Not achieved | Not achieved yet |
| New Zealand |  | Not achieved | Not achieved yet |
| Nicaragua |  | Not achieved | Not achieved yet |
| Niger |  | Not achieved | Not achieved yet |
| Nigeria |  | Not achieved | Not achieved yet |
| Niue |  | Not achieved | Not achieved yet |
| North Macedonia |  | Not achieved | Not achieved yet |
| Oman |  | Not achieved | Not achieved yet |
| Pakistan |  | Not achieved | Not achieved yet |
| Panama |  | Not achieved | Not achieved yet |
| Papua New Guinea |  | Not achieved | Not achieved yet |
| Peru |  | Not achieved | Not achieved yet |
| Philippines |  | Not achieved | Not achieved yet |
| Poland |  | Not achieved | Not achieved yet |
| Portugal |  | Not achieved | Not achieved yet |
| Qatar |  | Not achieved | Not achieved yet |
| Republic of Korea |  | Not achieved | Not achieved yet |
| Republic of Moldova |  | Not achieved | Not achieved yet |
| Romania |  | Not achieved | Not achieved yet |
| Russian Federation |  | Not achieved | Not achieved yet |
| Rwanda |  | Not achieved | Not achieved yet |
| Samoa |  | Not achieved | Not achieved yet |
| Sao Tome and Principe |  | Not achieved | Not achieved yet |
| Saudi Arabia |  | Not achieved | Not achieved yet |
| Senegal |  | Not achieved | Not achieved yet |
| Serbia |  | Not achieved | Not achieved yet |
| Seychelles |  | Not achieved | Not achieved yet |
| Sierra Leone |  | Not achieved | Not achieved yet |
| Singapore |  | Not achieved | Not achieved yet |
| Slovakia |  | Not achieved | Not achieved yet |
| Slovenia |  | Not achieved | Not achieved yet |
| Solomon Islands |  | Not achieved | Not achieved yet |
| Somalia |  | Not achieved | Not achieved yet |
| South Africa |  | Not achieved | Not achieved yet |
| South Sudan |  | Not achieved | Not achieved yet |
| Sri Lanka |  | Not achieved | Not achieved yet |
| Sudan |  | Not achieved | Not achieved yet |
| Suriname |  | Not achieved | Not achieved yet |
| Syrian Arab Republic |  | Not achieved | Not achieved yet |
| Tajikistan |  | Not achieved | Not achieved yet |
| Thailand |  | Not achieved | Not achieved yet |
| Timor-Leste |  | Not achieved | Not achieved yet |
| Togo |  | Not achieved | Not achieved yet |
| Tonga |  | Not achieved | Not achieved yet |
| Trinidad and Tobago |  | Not achieved | Not achieved yet |
| Tunisia |  | Not achieved | Not achieved yet |
| Turkey |  | Not achieved | Not achieved yet |
| Uganda |  | Not achieved | Not achieved yet |
| Ukraine |  | Not achieved | Not achieved yet |
| United Arab Emirates |  | Not achieved | Not achieved yet |
| United Republic of Tanzania |  | Not achieved | Not achieved yet |
| Uruguay |  | Not achieved | Not achieved yet |
| Uzbekistan |  | Not achieved | Not achieved yet |
| Vanuatu |  | Not achieved | Not achieved yet |
| Venezuela (Bolivarian Republic of) |  | Not achieved | Not achieved yet |
| Viet Nam |  | Not achieved | Not achieved yet |
| Yemen |  | Not achieved | Not achieved yet |
| Zambia |  | Not achieved | Not achieved yet |
| Zimbabwe |  | Not achieved | Not achieved yet |

Note: BRI countries: the Belt and Road countries.

The achievement of SDGs in 2021 was assessed based on the GBD estimate for 2021, and the future achievement by 2030 was the projected value from the GBD foresight visualization in GBD 2021 (https://vizhub.healthdata.org/gbd-foresight/).
